# *Salvia chinensis* Benth Inhibits Triple-Negative Breast Cancer Progression by Inducing the DNA Damage Pathway

**DOI:** 10.3389/fonc.2022.882784

**Published:** 2022-08-10

**Authors:** Kai-nan Wang, Ye Hu, Lin-lin Han, Shan-shan Zhao, Chen Song, Si-wen Sun, Hui-yun Lv, Ni-na Jiang, Ling-zhi Xv, Zuo-wei Zhao, Man Li

**Affiliations:** ^1^ Department of Oncology, The Second Hospital of Dalian Medical University, Dalian, China; ^2^ Health Management Center, The Second Hospital of Dalian Medical University, Dalian, China; ^3^ Department of Breast Surgery, The Second Hospital of Dalian Medical University, Dalian, China

**Keywords:** *Salvia chinensia* Benth, triple negative breast cancer, DNA damage and repair, Quercetin, β-Sitosterol

## Abstract

**Objective:**

Triple-negative breast cancer (TNBC) is distinguished by early recurrence and metastases, a high proclivity for treatment resistance, and a lack of targeted medicines, highlighting the importance of developing innovative therapeutic techniques. *Salvia chinensis* Benth (SCH) has been widely studied for its anticancer properties in a variety of cancers. However, its significance in TNBC treatment is rarely discussed. Our study investigated the anticancer effect of SCH on TNBC and the underlying mechanisms.

**Methods:**

First, we used clonogenic, cell viability, flow cytometry, and Transwell assays to assess the effect of SCH on TNBC. Bioinformatic studies, especially network pharmacology-based analysis and RNA sequencing analysis, were performed to investigate the constituents of SCH and its molecular mechanisms in the suppression of TNBC. High-performance liquid chromatography and thin-layer chromatography were used to identify two major components, quercetin and β-sitosterol. Then, we discovered the synergistic cytotoxicity of quercetin and β-sitosterol and assessed their synergistic prevention of cell migration and invasion. Breast cancer xenografts were also created using MDA-MB-231 cells to test the synergistic therapeutic impact of quercetin and β-sitosterol on TNBC *in vivo*. The impact on the DNA damage and repair pathways was investigated using the comet assay and Western blot analysis.

**Results:**

Our findings showed that SCH decreased TNBC cell growth, migration, and invasion while also inducing cell death. We identified quercetin and β-sitosterol as the core active components of SCH based on a network pharmacology study. According to RNA sequencing research, the p53 signaling pathway is also regarded as a critical biological mechanism of SCH treatment. The comet assay consistently showed that SCH significantly increased DNA damage in TNBC cells. Our *in vivo* and *in vitro* data revealed that the combination of quercetin and β-sitosterol induced synergistic cytotoxicity and DNA damage in TNBC cells. In particular, SCH particularly blocked the inter-strand cross-link repair mechanism and the double-strand breach repair caused by the homologous recombination pathway, in addition to inducing DNA damage. Treatment with quercetin and β-sitosterol produced similar outcomes.

**Conclusion:**

The current study provides novel insight into the previously unknown therapeutic potential of SCH as a DNA-damaging agent in TNBC.

## Introduction

Despite significant advances in diagnostic and treatment strategies, breast cancer remains the leading cause of cancer-related death in women (1). Triple-negative breast cancer (TNBC) is detected in 15% to 20% of women with breast cancer and is characterized by the lack of ER, PR, and HER2 (2). In the first 3 to 5 years following the initial diagnosis, TNBC patients have an increased risk of recurrence and metastasis (2, 3). The main obstacles in the treatment of TNBC are primary and acquired resistance to treatments (4). When one signaling node is suppressed, cancer cells prefer to rely on alternate signaling routes, which is recognized as the underlying mechanism of acquired resistance (5). As a result, multitarget tactics may be more effective than single-node strategies.

Herbs have long been used as adjuvant therapies for TNBC, particularly in patients with advanced metastatic cancers (6). Previous research found that combining herbal medication with chemotherapy or radiotherapy might boost antitumor effectiveness while decreasing side effects (7, 8). *Salvia chinensis* Benth (SCH), also known as *Salvia chinensis* herbal or Chinese Sage (Shijianchuan), is extensively documented in the Compendium of Materia Medica (Ming Dynasty, A.D. 1590). SCH is often used to treat ostealgia and swelling carbuncles (9). Previous studies have suggested that SCH has anticancer efficacy in breast, gastric, nasopharynx, lung, colon, liver, and pancreatic cancer (10, 11). However, there are few studies on the effects and underlying mechanisms of SCH in TNBC.

We aimed to investigate the role of SCH in TNBC therapy in this study. To the best of our knowledge, this is the first *in vitro* and *in vivo* study indicating that SCH promotes TNBC cell death by generating DNA damage while simultaneously decreasing DNA damage repair. Furthermore, subsequent findings show that quercetin and β-sitosterol, two essential constituents of SCH, synergistically suppress TNBC cells by increasing DNA damage. Overall, our data suggest that SCH has strong potential as an adjuvant treatment option for TNBC patients.

## Materials and methods

### Cells and Reagents

The human TNBC cell lines MDA-MB-231 and HCC1187, the human mammary epithelial cell line MCF-10A, the mouse TNBC cell line 4T1, the human luminal breast cancer cell lines MCF-7 and T47D, and the human HER2-positive breast cancer cell lines HCC1954 and SKBR3 were procured from ATCC (Manassas, VA, USA) and were sustained in culture medium (MDA-MB-231 in DMEM (Gibco, Grand Island, NY, USA, C11995500BT), other cell lines in RPMI-1640 (Gibco, C11875500BT)) supplemented with 10% fetal bovine serum (FBS, Biological Industries, Cromwell, CT, USA, 04-001-1ACS) and 100 units per ml penicillin/streptomycin (HyClone, Logan, UT, USA, SC30010) at 5% CO_2_ and a moderate temperature of 37°C in an incubator. We prepared the culture medium for MCF-10A cells as described previously (12). The cell lines were free of mycoplasma contamination and were verified by short tandem repeat (STR) profiling. Quercetin and β-sitosterol were purchased from MedChemExpress (MCE, Princeton, NJ, USA, HY-18085 and HY-N0171A). To procure the aqueous extracts of SCH, the shredded herb was boiled with a 10× volume of water for 2 h (in duplicate), followed by freeze-drying of the concentrate. The resultant dry powder was preserved at -20°C. The doses used in the current investigation were aliquoted as an equivalent weight of raw herb per ml.

### Clonogenic Assay

The cells (1 × 10^4^ cells/well) were incubated in 12-well plates and cultured continuously, and every third day, the medium was renewed. After culturing, the cells that were stained with 0.5% crystal violet were subsequently washed with PBS, dried, and finally dissolved in 10% acetic acid. The OD was measured at 590 nm with the aid of a SpectraMax 190 (Molecular Devices, San Jose, CA, USA).

### Migration and Invasion Assay

Cell migration and invasion were assessed according to the instructions of the manufacturer (Corning, Tewksbury, MA, USA) of the Transwell assay kits. In brief, the chambers were filled with BD Biosciences Matrigel (San Jose, CA, USA) for the invasion assay or without Matrigel for the migration assay. Serum-restricted medium (5 × 10^3^ cells/200 μl of cell suspension) was added to the upper chamber, with SCH (100 mg/ml) medium containing 10% FBS in the lower chamber. After 2 days of incubation, the non-invading cells were carefully removed, and the invading cells stained with 0.5% crystal violet were imaged *via* inverted phase-contrast microscopy (Olympus Corporation, Tokyo, Japan). Images were captured with a microscope (Leica, Wetzlar, Germany, DMI1) at ×100 magnification. Cell counts were assessed from three random microscope fields for every group.

### Flow Cytometry

A flow cytometric evaluation of cell apoptosis was performed by Annexin V and PI staining *via* the APC Annexin V Apoptosis Detection Kit (KeyGen Biotech, Nanjing, China, KGA1030). The cells for cell-cycle analysis were prepared and stained as previously described (13). All examinations were performed on a BD FACSCanto™ II (BD Biosciences, USA).

### Western Blot Analysis

RIPA buffer augmented with protease and phosphatase inhibitors was administered for cell lysis (14). The proteins were separated by SDS-PAGE and then transferred to nitrocellulose blotting membranes (GE Healthcare Life Science, Germany). The spots were examined with antibodies against PARP (Cell Signaling Technology, CST, Danvers, MA, USA, 9542), vinculin (Abcam, Cambridge, MA, USA, ab129002), GAPDH (HUABIO, Woburn, MA, USA, M1310-2), ATM (Santa Cruz Biotechnology, Dallas, TX, USA, sc135663), p-ATM (Ser1981) (CST, 5883), H2AX (Santa Cruz Biotechnology, sc517336), p-H2AX (Ser139) (Santa Cruz Biotechnology, sc517348), CHK1 (Santa Cruz Biotechnology, sc8408), p-CHK1 (Ser345) (CST, 2348), P53 (Santa Cruz Biotechnology, sc126), p-P53 (Ser15) (CST, 9286), ATR (Santa Cruz Biotechnology, sc515173), p-ATR (Ser428) (CST, 2853), FANCD2 (Santa Cruz Biotechnology, sc20022), and RAD51 (Santa Cruz Biotechnology, sc398587).

### Cell Viability

The cells (10^3^ cells/well) were incubated in 96-well plates. Then, the viability of the cells was evaluated with the aid of CCK-8 (Bimake, B34304). A SpectraMax 190 (Molecular Devices, USA) was used to assay the optical density (OD) at 450 nm. The IC50s were calculated from sigmoidal dose–response curves utilizing Prism.

### Network Pharmacology Analysis

The chemical ingredients and target genes of SCH were investigated based on the Traditional Chinese Medicine Systems Pharmacology (TCMSP) database. Parameters such as oral bioavailability (OB) ≥30% and drug likeness (DL) ≥0.18 were evaluated according to the thresholds recommended in the TCMSP. TNBC-related genes were collected from Gene Cards (www.genecards.org) and Online Mendelian Inheritance in Man (OMIM). After collecting the data for genes of interest pertaining to SCH and TNBC, the shared genes between SCH-related genes in TNBC were identified by Kyoto Encyclopedia of Genes and Genomes (KEGG) pathway analysis (15).

### High-Performance Liquid Chromatography

The quercetin standard was dissolved in methanol, generating concentrations of 0.1, 0.05, 0.02, 0.01, and 0.005 mg/ml, which were used to prepare the standard curves for quercetin quantification in the tested sample. Aqueous extracts of SCH (100 mg) were extracted *via* ultrasonication in methanol (1 ml) for 20 min. The extracted solutions were subjected to a 0.22-μm filter membrane, yielding a clear solution for high-performance liquid chromatography (HPLC) analysis. Analytical HPLC experiments were conducted on a Dionex UltiMate 3000 instrument (Thermo Scientific, Waltham, MA, USA) equipped with a diode array detector and a Waters XSelect HSS T3 C18 column (250 × 4.6 mm, 5 μm). The chromatographic conditions for HPLC analysis were as follows: acetonitrile-H2O (0.1% H_3_PO_4_) (30:70, v/v), flow rate of 1 ml per min, column temperature of 30°C, run time of 20 min, and a detected wavelength of 360 nm.

### Thin-Layer Chromatography

Using β-sitosterol as the standard compound, SCH was analyzed by thin-layer chromatography (TLC). In general, TLC was performed using precoated silica gel GF254 plates (2.5 × 7.5 cm, Qingdao Marine Chemical Inc.) with petroleum ether-ethyl acetate (9:1, v/v) as the developing solvents. Then, the spots were visualized by spraying the plates with 20% sulfuric acid in EtOH, followed by heating at 90°C.

### RNA Sequencing and Data Analysis

RNA sequencing of MDA-MB-231 cells (vehicle vs. SCH treatment) was carried out by Novogene Corporation (Beijing, China). The sequencing libraries were created using the NEBNext^®^ Ultra™ RNA Library Prep Kit for Illumina^®^ (NEB, Ipswich, MA, USA) according to the manufacturer’s instructions. FPKM (expected number of Fragments Per Kilobase of transcript sequence per Million base pairs sequenced) was used to estimate gene expression levels. The ClusterProfiler R package was used to test the statistical enrichment of differentially expressed genes in KEGG pathways. We have submitted our RNA-seq dataset to the Gene Expression Omnibus (GEO) under accession number GSE189547.

### Comet Assay

DNA damage was assayed by a comet assay (16). Using alkaline lysis buffer, the cells were lysed and electrophoresed (buffer counteracted with PBS), stained, and assessed with Gold View (Coolaber, SL2140). Ultimately, cells were documented with a Leica fluorescence microscope (Leica Microsystems, Wetzlar, Germany), and the occurrence of the DNA in the tail (percentage) was evaluated on a quantitative scale of DNA damage using CASP (Comet Assay Software Project Lab) software.

### Animals

The protocol for animal experiments was approved by the Animal Research Committee of Dalian Medical University. To develop breast cancer xenografts, MDA-MB-231 cells (6 × 10^6^) were subcutaneously inoculated into female BALB/c nude mice. At the culmination of the 7th day of implantation, mice were administered vehicle, quercetin (75 mg kg^-1^), β-sitosterol (75 mg kg^-1^), or both quercetin and β-sitosterol each day *via* oral gavage for 3 weeks. The tumor volumes were calculated every 5 days with the aid of calipers and were quantified using the following formula: tumor volume = (length × width^2^)/2. At the end of the experiment, mice were subjected to carbon dioxide asphyxiation for euthanasia: the mice were put into a clean container with carbon dioxide at a slowly increasing concentration. After approximately 10 min, the mice died slowly and painlessly.

### Immunohistochemistry

Immunohistochemical (IHC) staining was performed using the streptavidin–peroxidase method. Formalin-fixed paraffin-embedded tumor tissues were cut into 4-µm-thick sections that were dewaxed and rehydrated using standard conditions. Sections were pretreated with 10 mM Tris–Na citrate (pH 6) for 20 min at 95°C and washed. Then, the sections were incubated for 10 min in 3% H_2_O_2_ in PBS to inhibit endogenous peroxidase. The slides were blocked in goat serum for 30 min, followed by incubation with antibodies (the same antibodies used for immunoblotting) overnight at 4°C. After washing with PBS, the biotinylated secondary antibody–HRP conjugate was applied for 60 min at room temperature. The sections were counterstained with hematoxylin. Additionally, some slides were stained with hematoxylin and eosin (H&E). After dehydration and mounting, histological samples were quantified using a microscope (Lecia, DMI1) at ×200 magnification. Tumor necrosis was determined according to the percentage of the overall necrosis area. The proteins involved in DNA damage and repair were quantified by the percentage of the overall area that was stained. For each sample, the percentages of positive staining were assessed on the basis of three random microscope fields, and each group was tested with three independent samples.

### Statistical Analysis

Data are presented as the mean ± standard deviation (SD). GraphPad Prism software was employed to perform unpaired two-sided t tests, one-way ANOVA, and Tukey’s multiple-comparison tests (for both *in vitro* and *in vivo* animal studies). CalcuSyn 2.0 software (Biosoft) was used for the drug interaction assessment (Chou–Talay method). An additive effect was indicated by combination index (CI) =1, whereas synergism was indicated by CI <1 and antagonism was indicated by CI >1. A P value <0.05 was considered to indicate statistical significance.

## Results

### SCH Suppresses TNBC Cell Growth and Induces TNBC Cell Apoptosis

**Figure 1:**
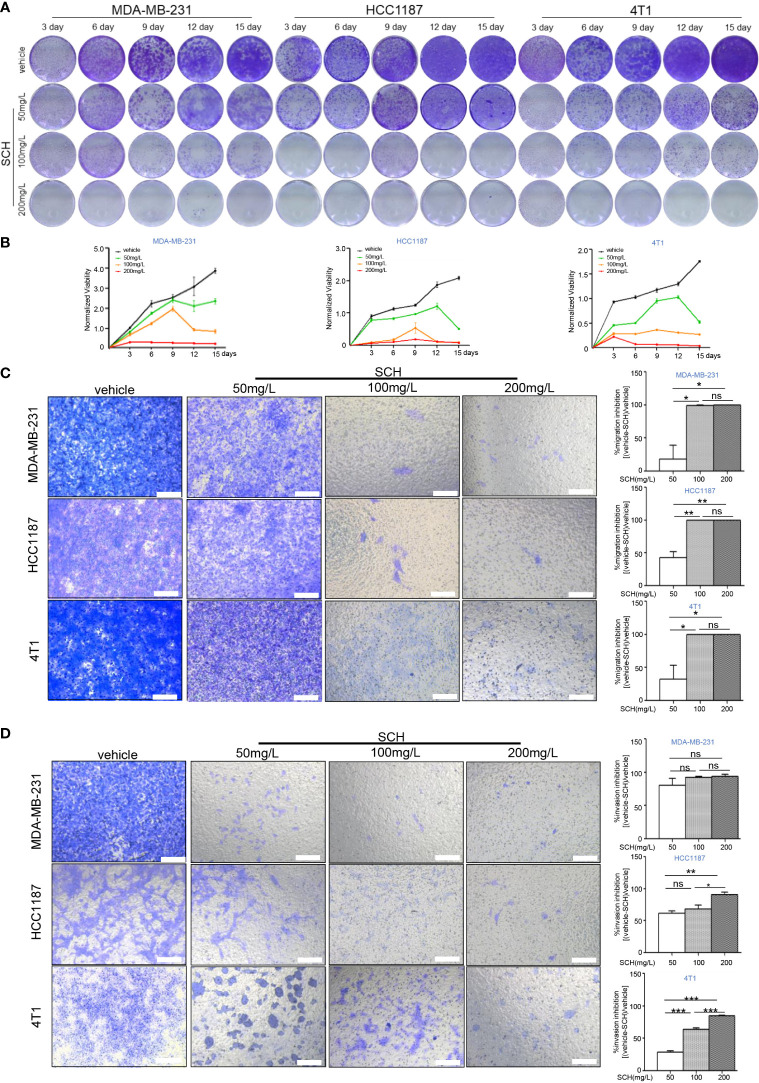
SCH inhibits TNBC cell growth, migration, and invasion. **(A)** TNBC cells were cultured with varying dosages of SCH for 3, 6, 9, 12, and 15 days and stained *via* crystal violet (media were replenished every third day). **(B)** The quantification of the clonogenic survival assay is shown as mean ± S.D. from three independent experiments. Representative images of the migration **(C)** and invasion **(D)** after SCH treatment. Scale bar, 100 µm. Results exhibited as mean ± S.D. from three separate trials. “ns”, not significant, *P < 0.05, **P < 0.01, ***P < 0.001 (Student’s *t*-test).

**Figure 2:**
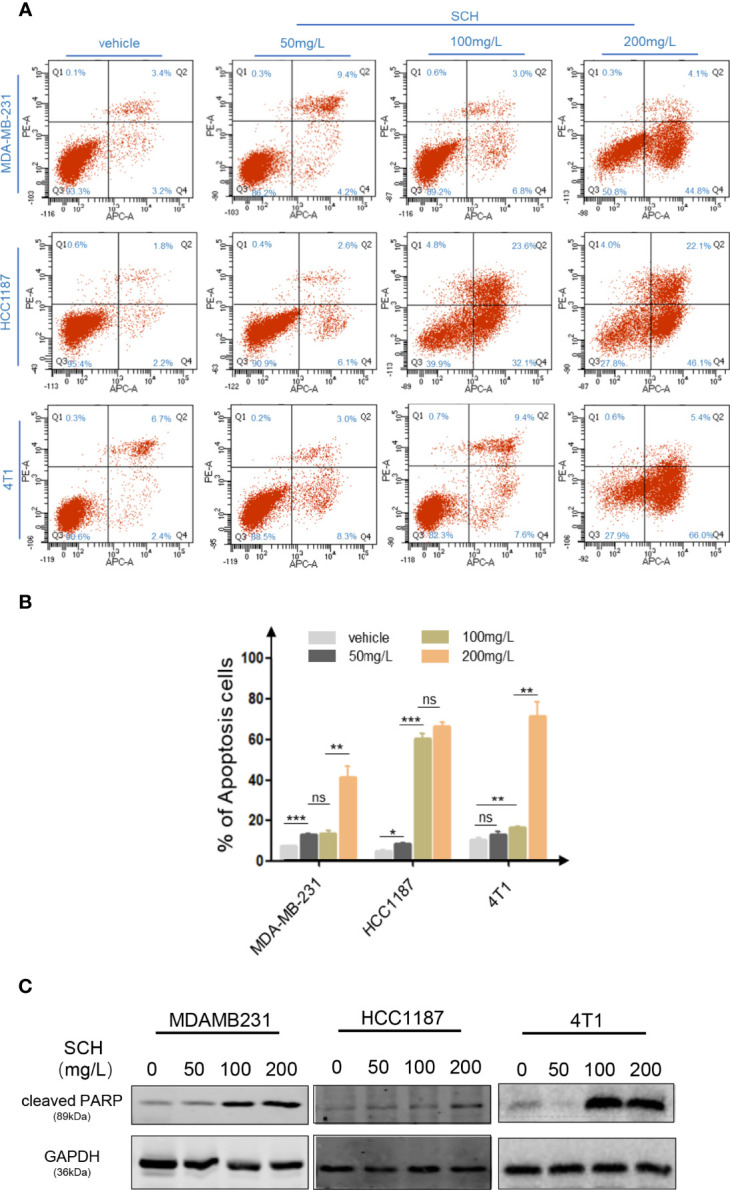
SCH activates TNBC cell apoptosis. **(A)** Apoptotic cell death was evaluated *via* flow cytometric analysis subsequently DAPI and FITC-Annexin V staining in cells treated with varying dosages of SCH. **(B)** The % of Annexin V+/PI- (early apoptotic cells, lower right), Annexin V+/PI+ (late apoptotic cells, upper right), Annexin V-/PI- (viable cells, lower left), and Annexin V+/PI+ (necrotic cells, upper left) cells are displayed. Columns epitomize the % of Annexin V-positive TNBC cells. “ns,” not significant, *P < 0.05, **P < 0.01, ***P < 0.001 (Student’s *t*-test). **(C)** TNBC cells were treated with varying dosages of SCH for 24 h and then subjected to an immunoblotting analysis.

We investigated the *in vitro* cytotoxicity of SCH against human TNBC cells (MDA-MB-231, HCC1187) and mouse TNBC 4T1 cells using a clonogenic survival experiment. **Figures 1A, B** shows the time-dependent and dose-dependent suppressive effect of SCH on TNBC cells. A high dose of SCH (200 mg/l) reduced the clonogenic potential to a level comparable to that achieved with a traditional chemical medication (cisplatin 5 µM, paclitaxel 10 µM) (**Figure S1A**). We also examined the drug sensitivity of various subtypes of breast cancer cells to SCH (**Figure S1B**). According to the data, the appropriate therapeutic dosage of SCH for TNBC is 50–200 mg/l. In addition, we performed a Transwell assay and found that SCH suppressed cellular migration and invasion 48 h after treatment (**Figures 1C, D**). The effect of SCH on cell survival was also investigated, and the results demonstrated that SCH treatment promoted G0/G1-phase cell-cycle arrest (**Figure S2**), followed by apoptotic cell death (**Figures 2A, B**), with increasing levels of cleaved PARP (**Figure 2C**), an indicator of apoptosis. Altogether, these results indicate that SCH has antitumor activity against TNBC cells.

### Quercetin and β-Sitosterol Synergistically Inhibit TNBC Cells

**Figure 3:**
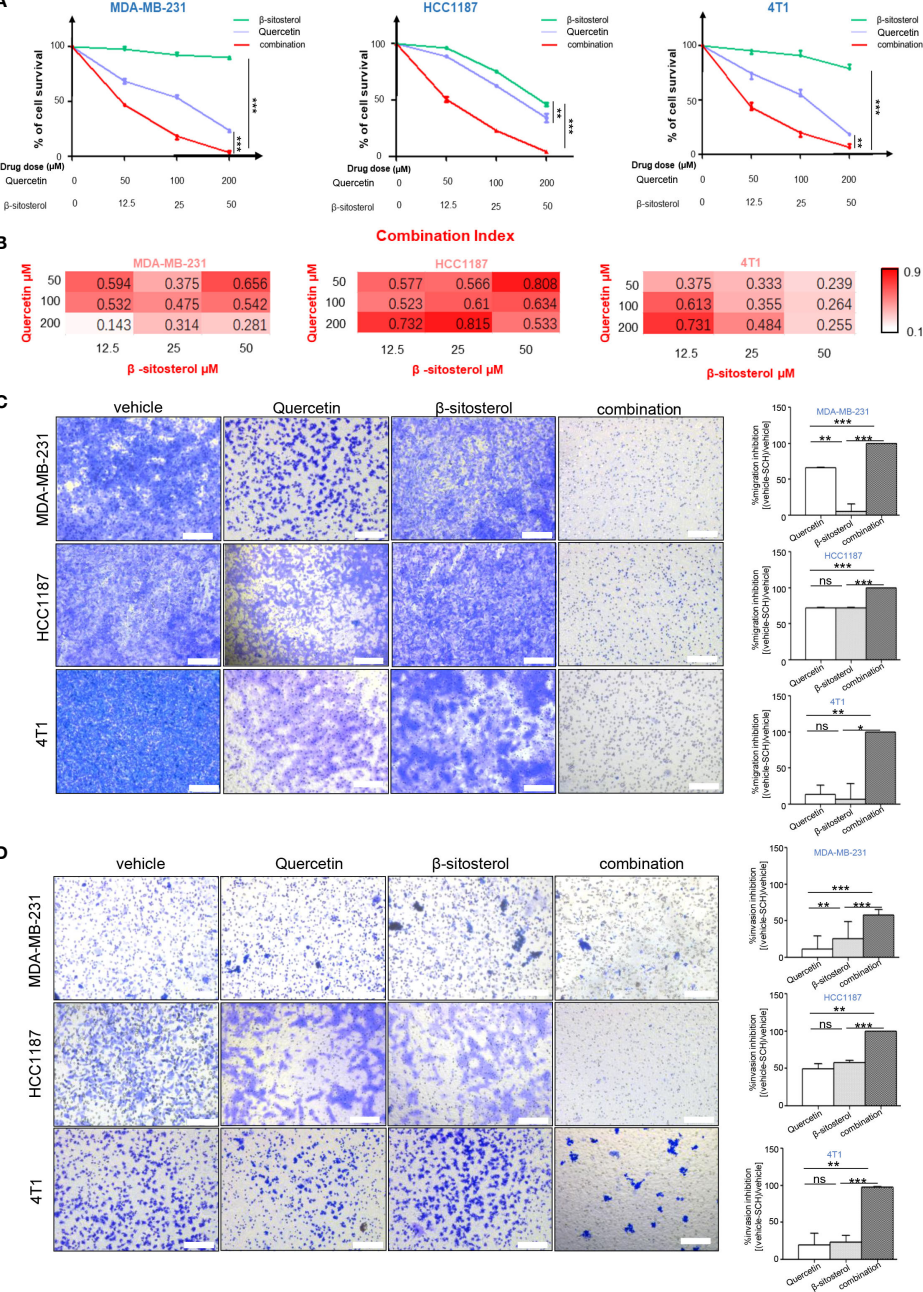
The combination of quercetin and β-sitosterol induces synergistic growth inhibitory effects on TNBC cells. **(A)** TNBC cells were simultaneously treated with quercetin and/or β-sitosterol for 48 h. Cell viability was measured *via* CCK-8 assay and depicted as the percentage of viable cells in the experimental group relative to than in the control group. Results are presented as mean ± S.D. from three separate trials. **(B)** The combined effect of quercetin and beta-sitosterol was gauged with the aid of CalcuSyn software. Heatmaps depict the combination index (CI). Representative images of the migration **(C)** and invasion **(D)** after quercetin and/or β-sitosterol treatment with Transwell assay. Scale bar, 100 µm. Results exhibited as mean ± S.D. from three separate trials. “ns,” not significant, *P < 0.05, **P < 0.01, ***P < 0.001 (Student’s *t*-test).

**Figure 4:**
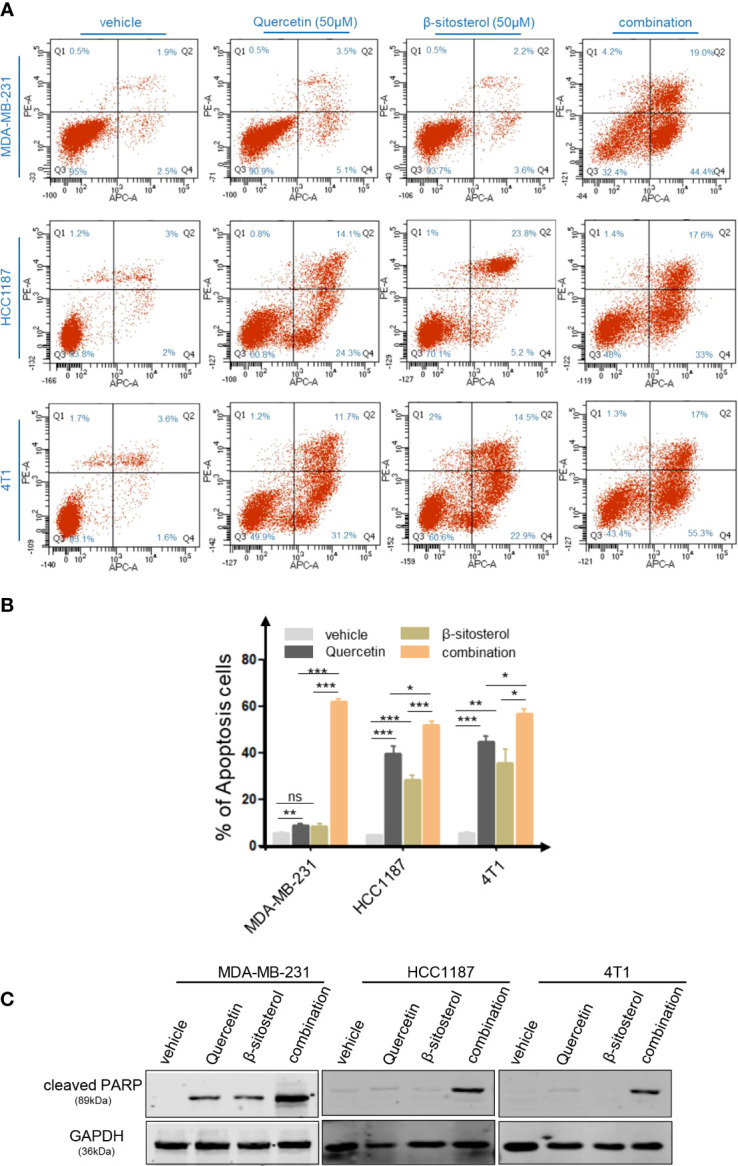
The combo treatment of quercetin and β-sitosterol synergistically increases TNBC cell apoptosis. **(A)** Apoptotic cell death was assessed by flow cytometric analysis following DAPI and FITC-Annexin V staining in cells treated with quercetin and/or β-sitosterol for 48 h. **(B)** The % of Annexin V+/PI- (early apoptotic cells, lower right), Annexin V+/PI+ (late apoptotic cells, upper right), Annexin V-/PI- (viable cells, lower left), and Annexin V+/PI+ (necrotic cells, upper left) cells are shown. Columns represent the percentages of Annexin V-positive TNBC cells. “ns,” not significant, *P < 0.05, **P < 0.01, ***P < 0.001 (Student’s *t*-test). **(C)** TNBC cells were treated with quercetin and/or β-sitosterol for 24 h and then subjected to an immunoblotting analysis.

Based on the TCMSP database, the main therapeutic constituents of SCH were identified as quercetin and β-sitosterol (**Table 1**). The ingredients with low OB (≤ 30%) or low DL (≤ 0.18) are not listed in the table. Using HPLC and TLC, we verified the presence of quercetin and β-sitosterol in SCH (**Figure S3**). The effects of quercetin and/or β-sitosterol on TNBC cells were then assessed using cell viability tests. Interestingly, both quercetin and β-sitosterol inhibited TNBC cell growth, and the combination treatment resulted in a more dramatic dose-susceptible suppression of cell viability than monotherapy (**Figure 3A**). The CI values revealed a strong synergism of the combination treatment, with CI <1.0 for all dosages tested (**Figure 3B**). Furthermore, the combination treatment’s synergism was verified by a 3-day clonogenic survival experiment (**Figures S4A, B**). We assessed the synergistic inhibition of the combination treatment on cell migration and invasion using a Transwell assay (**Figures 3C, D**). In addition to the synergistic inhibition of TNBC viability, the synergistic induction of G0/G1-phase cell-cycle arrest (**Figure S4C**), cell apoptosis (**Figures 4A, B**), and activation of cleaved PARP were observed after the combination treatment (**Figure 4C**). These findings confirm that the major components of SCH are effective in inhibiting TNBC growth and that the combination of these substances generates significant synergistic cytotoxicity.

**Table 1:** Bioactive Compounds of SCH.

Molecule ID	Molecule Name	OB (%)	DL
MOL000358	beta-sitosterol	36.91	0.75
MOL000098	quercetin	46.43	0.28

OB, oral bioavailability. DL drug likeness.

### SCH Exerts Its Antitumor Effect Through the P53 Signaling Pathway in TNBC

**Figure 5:**
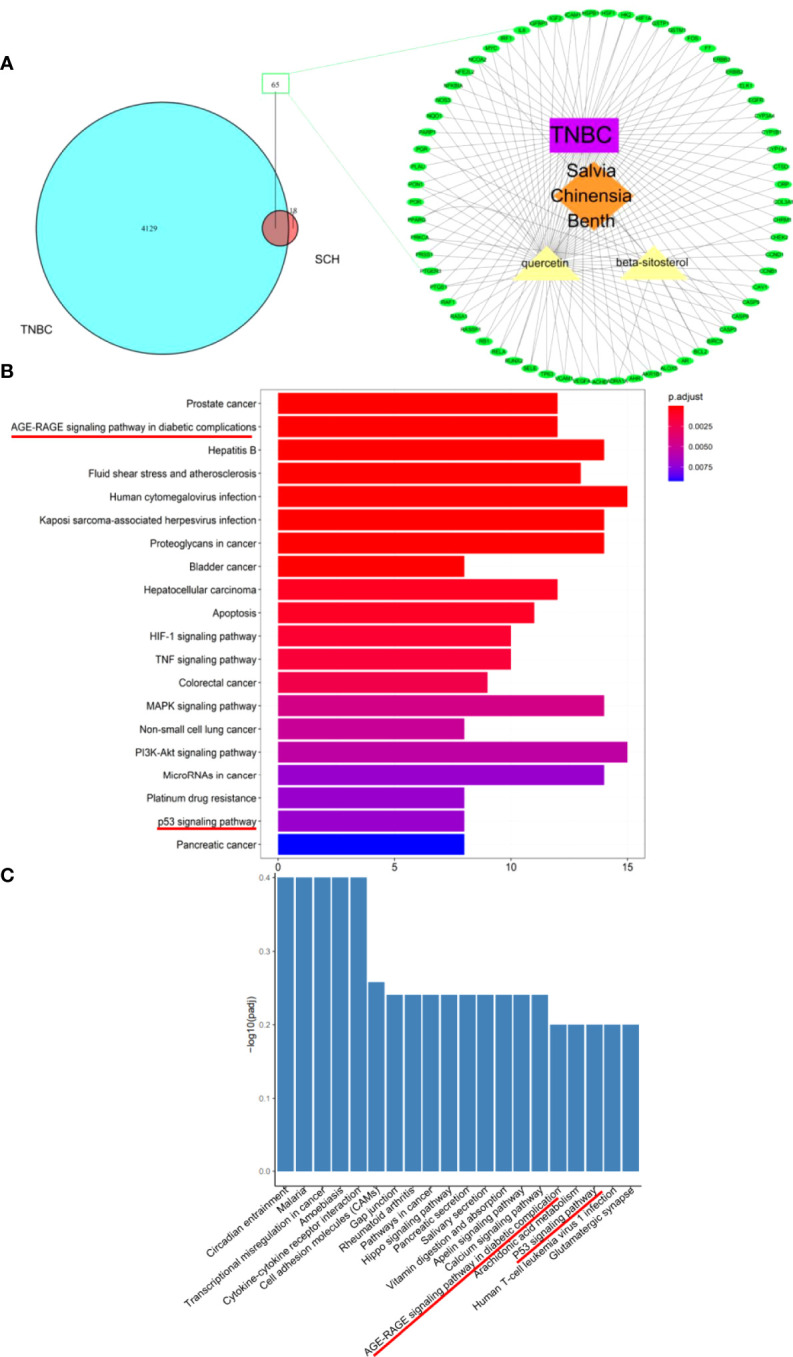
Bioinformatic analysis and transcriptome analysis divulge latent targets of SCH. **(A)** C–T network of SCH. The green ellipse represents target genes. The purple rectangle, orange diamond, and yellow triangle stand for compounds. KEGG pathway enrichment scrutiny of target genes of SCH from online database **(B)** and transcriptome analysis **(C)**. Pathways with attuned *P*-value <0.05 are shown.

Using network pharmacology analysis, 83 genes were identified as SCH- candidate genes, while 4,129 TNBC-related genes were acquired from Gene Cards and the OMIM database. Next, we located 65 shared genes between SCH-targeted genes and TNBC-related genes (**Figure 5A**). Then, to identify SCH-related pathways in TNBC, KEGG analysis of shared genes was performed. As shown in **Figure 5B**, many classical pathways, such as the p53 signaling pathway, apoptosis pathway, and TNF signaling pathway, were represented. We performed RNA sequencing to detect transcriptome alterations in MDA-MB-231 cells generated by SCH treatment for 24 h to learn more about the underlying anticancer mechanism of SCH. RNA sequencing analysis revealed a substantial link between the p53 signaling pathway and SCH, confirming the findings of bioinformatic research (**Figure 5C**). Recent research showed that quercetin and β-sitosterol therapy triggered the p53 signaling pathway (17–19). Taken together, the current findings reveal a link between SCH therapy and the regulation of the p53 signaling pathway in TNBC cells.

### SCH Activates DNA Damage and Impairs DNA Damage Repair in TNBC Cells

**Figure 6:**
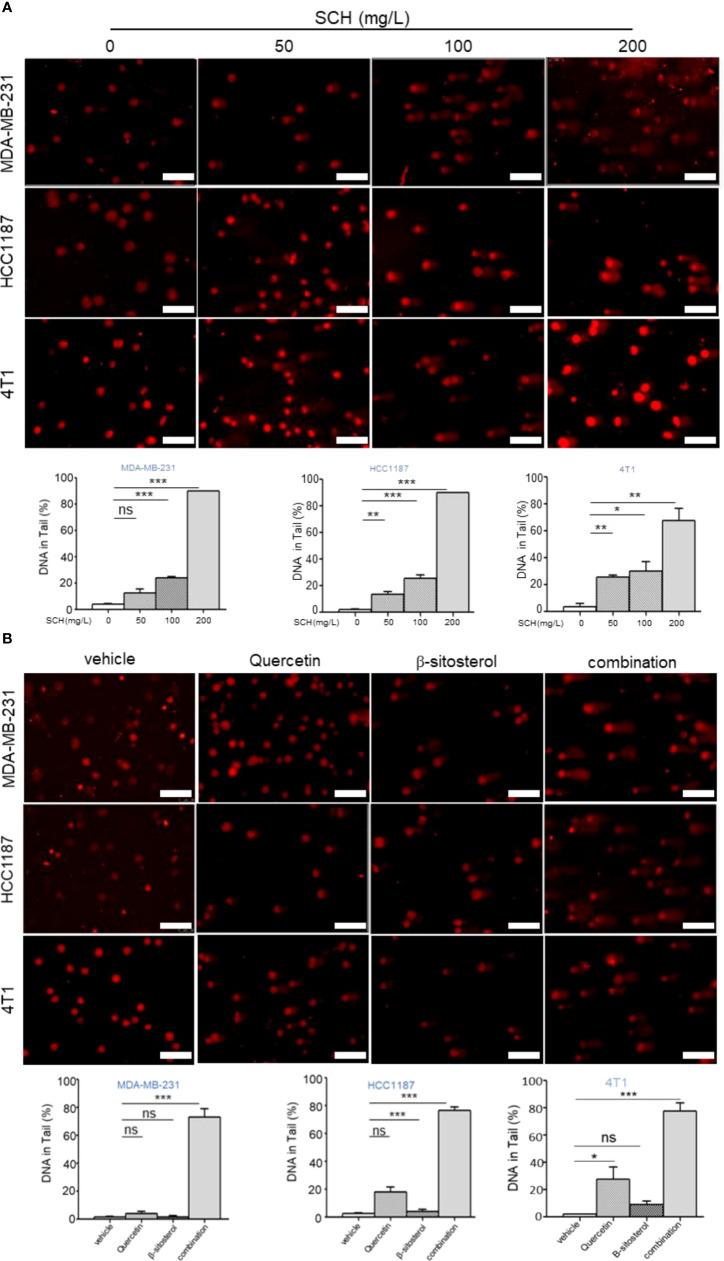
SCH treatment induces DNA damage *via* the synergistic effect of quercetin and β-sitosterol. **(A)** Comet assay (scale bar, 100 μm) was executed to appraise DNA damage in TNBC cells administered with varying dosages of SCH for 48 h. **(B)** Comet assay (scale bar, 100 μm) was performed to evaluate DNA damage in TNBC cells treated with quercetin and/or β-sitosterol for 48 h. The DNA quantification in the tail from three separate trials is revealed as mean ± S.D., ns-not significant, *P < 0.05, **P < 0.01, ***P < 0.001 (Student’s *t*-test).

**Figure 7:**
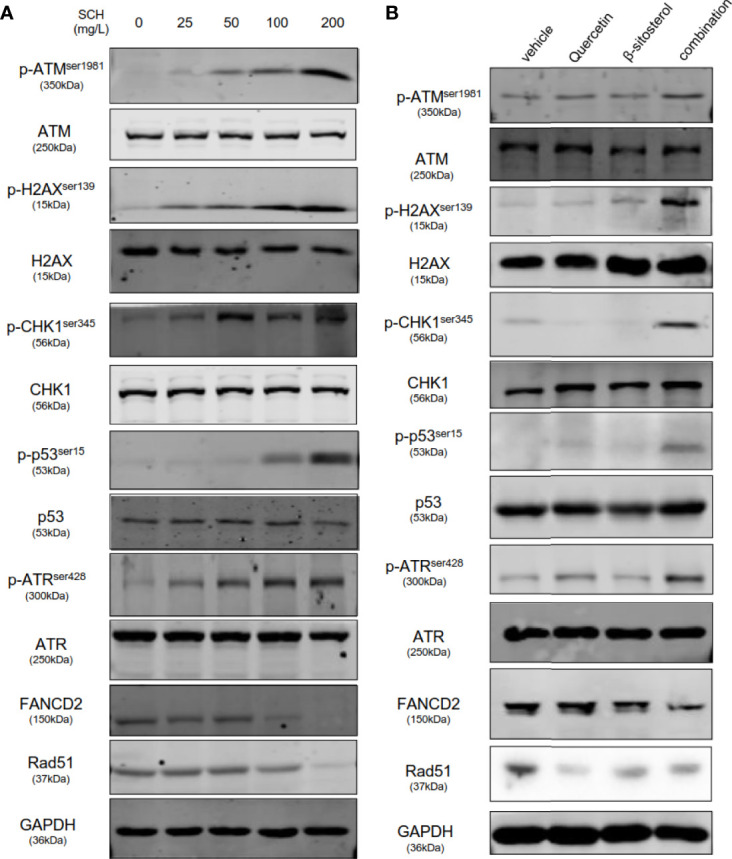
SCH and its key ingredients, quercetin and β-sitosterol, activate DNA damage response pathways and suppress DNA repair pathways. MDA-MB-231-whole-cell lysates administrated with varying concentrations of SCH **(A)**, quercetin and/or β-sitosterol **(B)** for 24 h were obtained and subsequently subjected to immunoblotting analysis and explored with the designated antibodies.

Stimulation of p53-mediated transcription is a crucial cellular reaction to DNA damage, culminating in cell-cycle arrest and reduced cell proliferation (20). Thus, we assessed the impact of SCH on DNA damage by the comet assay. SCH treatment significantly increased DNA damage in a dose-dependent manner, as measured by the percentage of DNA in the tails (**Figure 6A**). Next, the effects of quercetin and β-sitosterol on DNA damage in TNBC cells were determined. In MDA-MB-231 cells, compared to a single drug, the combination of quercetin and β-sitosterol induced a strong synergistic induction of DNA damage (**Figure 6B**). At the molecular level, we conducted immunoblot analysis to evaluate the effects of SCH and its active ingredients quercetin and β-sitosterol on the expression of DNA damage response and repair proteins. In MDA-MB-231 cells treated with SCH for 24 h, DNA damage response proteins (p-H2AX^ser139^, p-ATM^ser1981^, p-CHK1^ser345^, p-ATR^ser428^, and p-p53^ser15^) were activated, while FANCD2 (involved in DNA inter-strand cross-link repair) and RAD51 (involved in DNA double-strand break repair) were downregulated (**Figure 7A**). To further investigate the suppression of DNA double-strand damage repair by SCH, we also evaluated the efficacy of the combination of SCH and olaparib (a PARP inhibitor) against TNBC (**Figure S5**). The combination of olaparib and SCH caused “synthetic lethality” by inducing the accumulation of single- and double-strand breaks simultaneously. Furthermore, compared to a single drug, quercetin and β-sitosterol induced substantial synergistic activation of DNA damage response proteins and inhibition of DNA damage repair proteins in MDA-MB-231 cells (**Figure 7B**). Together, these findings suggest that SCH suppresses TNBC progression *via* the synergistic ability of quercetin and β-sitosterol to induce DNA damage and restrain DNA damage repair.

### Quercetin and β-Sitosterol Synergize to Suppress TNBC Tumor Growth *In Vivo*

**Figure 8:**
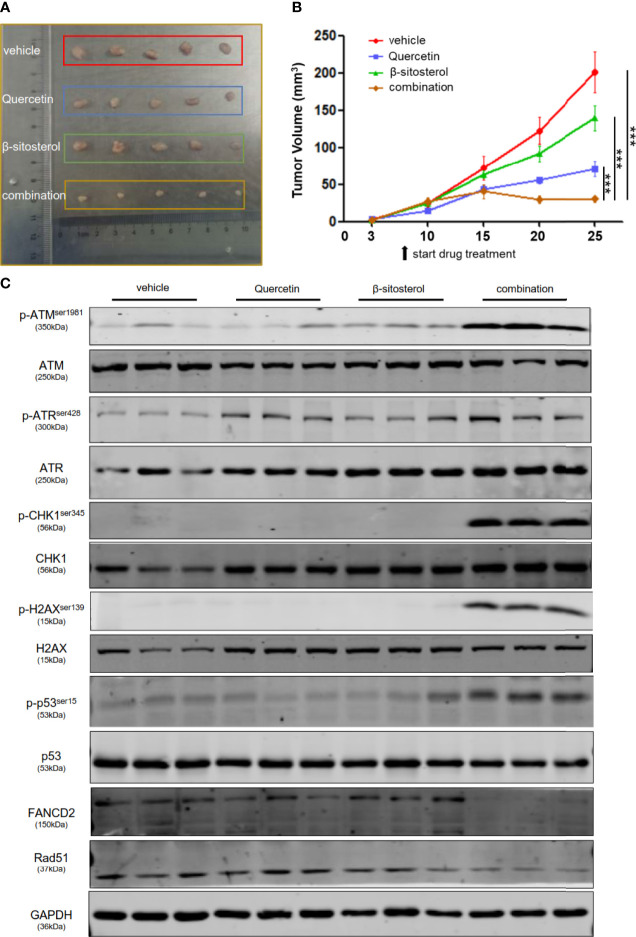
Combination therapy leads to significant tumor regression *in vivo*. **(A)** Nude mice were subcutaneously injected with MDA-MB-231 cells. Representative images show the dissected tumors in distinct treatment groups as specified at the endpoint. **(B)** The tumor growth curve of xenografts treated with vehicle or quercetin or β-sitosterol or combination. The data presented as mean ± S.E.M. n = 5 for each group. ***P < 0.001 (one-way ANOVA, with Turkey’s multiple-comparison tests). **(C)** The tumor lysates were collected, and the protein profusion was established by immunoblotting analysis.

After establishing that quercetin and β-sitosterol synergize to inhibit TNBC cell proliferation and survival *in vitro*, we evaluated their *in vivo* efficacy in a human breast cancer xenograft mouse model. After tumor establishment, the mice were treated with vehicle, quercetin, β-sitosterol, or a combination of quercetin and β-sitosterol. We found that the tumors in mice treated with a combination of quercetin and β-sitosterol were much smaller than the tumors in animals treated with a single agent (quercetin or β-sitosterol) (**Figures 8A, B**). All of the treatments were well tolerated (**Figure S6**). Furthermore, immunoblotting of tumor lysates confirmed the *in vivo* impacts of quercetin and β-sitosterol on DNA damage and repair pathways (**Figure 8C**). H&E and IHC analysis demonstrated that a combination of quercetin and β-sitosterol treatment induced more tumor necrosis and DNA damage than a single treatment (**Figure S7**). These findings suggest that quercetin and β-sitosterol inhibited TNBC cell proliferation *in vivo* by inducing DNA damage in a synergistic way.

## Discussion

TNBC patients tend to have worse morbidity than other subtypes due to significant molecular heterogeneity and metastatic potential, a lack of effective targeted treatments, and a strong predisposition to multidrug resistance (21). DNA-damaging agents, such as PARP inhibitors, cisplatin, and carboplatin, have long been used to treat TNBC and have been shown to dramatically improve patient survival. Our results revealed that SCH inhibited TNBC tumor growth *in vivo* and *in vitro* by causing DNA damage and suppressing DNA repair. This finding is useful as it provides a novel multitarget DNA-damaging agent for patients with advanced TNBC who have developed multidrug resistance.

SCH has been shown to have anticancer effects on hepatocellular carcinoma, gastric cancer, nasopharyngeal carcinoma, luminal breast cancer, and other cancers (9, 11, 22–25). We first observed the suppressive effect of SCH on TNBC cell proliferation, migration, and invasion, as well as the induction of cell death, in the current study. Moreover, SCH dramatically decreased tumor growth in TNBC xenograft mouse models, which was consistent with the *in vitro* data. Through network pharmacology and RNA sequencing analyses, two important constituents of SCH, quercetin and β-sitosterol, were found, and a putative relationship between SCH and the p53 signaling pathway was identified. The activation of the tumor-suppressor p53 is regarded as the major mechanism of apoptosis induced by DNA damage (26). We thus focused on the association between SCH and DNA damage and found that SCH treatment caused substantial DNA damage. Furthermore, immunoblotting revealed that after SCH therapy, DNA damage response proteins were activated, whereas DNA repair proteins were inhibited. These findings suggest that SCH could be used as a novel DNA-damaging agent to treat TNBC.

SCH has a variety of components, and we used HPLC and TLC to identify quercetin and β-sitosterol. Our data showed that the combination treatment of quercetin and β-sitosterol led to the synergistic suppression of cell viability, along with induction of apoptosis and caspase activation. Quercetin suppresses tumor progression through a variety of mechanisms, including antioxidative activity, growth inhibition, apoptosis induction, reduced inflammation, and angiogenesis inhibition (27–34). β-Sitosterol is a plant-derived compound that has anticancer properties in the context of breast, prostate, colon, and lung cancer. According to recent research, β-sitosterol disrupts the cell cycle, regulates multiple signaling pathways, and has effects on apoptosis, proliferation, survival, invasion, angiogenesis, metastasis, and inflammation (35–39). However, the effects of quercetin and β-sitosterol on DNA damage have yet to be fully explored. In the current study, we found that when quercetin and β-sitosterol were administered together, there was a larger increase in DNA damage and a larger reduction in DNA repair than when they were given separately.

Previously, only a few studies have examined the underlying mechanism of SCH for oncotherapy. Our findings reveal that SCH appears to target DNA damage mechanisms. The proteins transmitting signals from DNA damage and cell-cycle checkpoints to DNA repair pathways consist of ataxia-telangiectasia mutated (ATM), ATM- and Rad3-related (ATR), CHK1, H2AX, RAD51, and FANCD2. ATM is recruited to double-strand breaks (DSBs) and executes checkpoint signaling. ATR is activated by replication stress, during which it facilitates fork stabilization and restart. CHK1 and H2AX are effector kinases that function downstream of ATR and ATM. RAD51 is directly involved in the DSB repair processes of homologous recombination. The activation of FANCD2 marks the major activation switch for the FA pathway in the process of inter-strand cross-link repair (40, 41). In cells treated with SCH, there was a considerable increase in the phosphorylation of CHK1, H2AX, ATM, and ATR, culminating in the activation of p53, which supports our theory. Moreover, SCH downregulated the expression of FANCD2 and RAD51 at the same time, causing further DNA damage.

Approximately 50%–60% of TNBCs will exhibit homologous recombination deficiency (HRD) due to the genetic or epigenetic inactivation of one or more HR pathway genes (42). HRD is associated with vulnerability to DNA-damaging agents like anthracyclines and platinum chemotherapeutic agents or PARP inhibitors (43). Our study found that SCH caused apoptosis in TNBC cells by inducing double-strand DNA damage and blocking DNA damage repair. Simultaneously, additional research demonstrated that SCH and olaparib had a synergistic impact. In the future, we could administer SCH as a sensitizer combined with platinum-based chemotherapeutic agents or PARP inhibitors to treat advanced refractory TNBC. The ability of SCH to sensitize cancer cells to anticancer agents or defeat drug resistance is yet to be verified by clinical trials.

**Figure 9:**
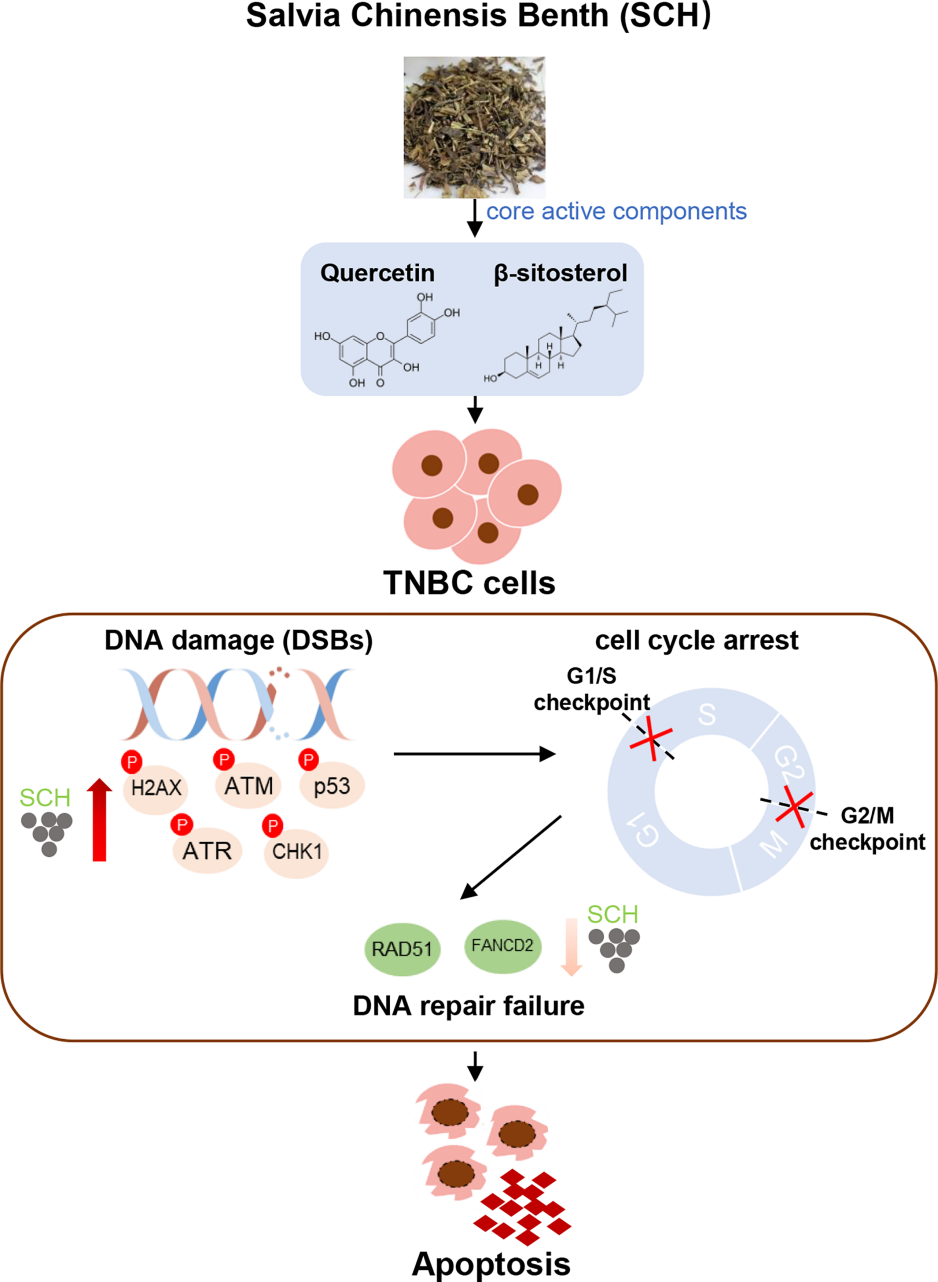
Schematics illustrate the identified signaling cascade triggered by SCH treatment. In TNBC cells, SCH treatment causes DNA damage (increased phosphorylation levels of H2AX, ATM, p53, ATR, and CHK1), which leads to cell-cycle arrest. At the same time, SCH treatment suppresses DNA repair (reduced RAD51 and FANCD2), and DNA repair failure finally leads to apoptosis.

In summary, our findings highlight the antitumor effect of SCH therapy in TNBC, which is mediated by improvement of the DNA damage response and impairment of the repair pathway, and revealed the strong synergistic effect of the combination of quercetin and β-sitosterol (**Figure 9**). The current study provides a rationale for the future application of SCH in TNBC patients. Furthermore, the finding that SCH sensitizes TNBC cells to olaparib suggests that there may be value in combining SCH with other DNA-damaging agents to treat TNBC, which requires extensive research.

## Data Availability Statement

The datasets presented in this study can be found in online repositories. The names of the repository/repositories and accession number(s) can be found below: National Center for Biotechnology Information (NCBI) BioProject database under accession number GSE189547.

## Ethics Statement

The animal study was reviewed and approved by the Animal Research Committee of Dalian Medical University.

## Author Contributions

Research design, ML, Z-wZ, and L-zX; Experiment operation, K-nW, YH, and L-lH; Data analysis, K-nW, L-zX, CS, H-yL and N-nJ; Manuscript preparation, K-nW, S-sZ, and S-wS; Thorough reading and final approval of the version, ML and Z-wZ to be published. All authors contributed to the article and approved the submitted version.

## Funding

This work was supported by the National Natural Science Foundation of China (81872156 and 82173361) and LiaoNing Revitalization Talents Program (XLYC2002057).

## Conflict of Interest

The authors declare that the research was conducted in the absence of any commercial or financial relationships that could be construed as a potential conflict of interest.

## Publisher’s Note

All claims expressed in this article are solely those of the authors and do not necessarily represent those of their affiliated organizations, or those of the publisher, the editors and the reviewers. Any product that may be evaluated in this article, or claim that may be made by its manufacturer, is not guaranteed or endorsed by the publisher.

## Abbreviations

SCH, *Salvia chinensis* Benth; TNBC, triple-negative breast cancer; ER, estrogen receptor; PR, progesterone receptor; HER2, human epidermal growth factor receptor 2; STR, short-tandem repeat; OD, optical density; TCMSP, Traditional Chinese Medicine Systems Pharmacology database; OB, oral bioavailability; DL, drug likeness; OMIM, Online Mendelian Inheritance in Man; GO, Gene Ontology; KEGG, Kyoto Encyclopedia of Genes and Genomes; PPI, protein–protein interaction; SD, standard deviation; CI, combination index; DDR, DNA damage repair; ATM, ataxia-telangiectasia mutated; ATR, ATM- and Rad3-related; DSB, double-strand break; PARP, poly ADP-ribose polymerase.
