# Supplementary figures and images for: Salvia chinensis Benth Inhibits Triple-Negative Breast Cancer Progression by Inducing the DNA Damage Pathway (part 1 of 2)
Source: Front Oncol. 2022 Aug 10;12:882784. doi: 10.3389/fonc.2022.882784 (PMC9404549; doi:10.3389/fonc.2022.882784)

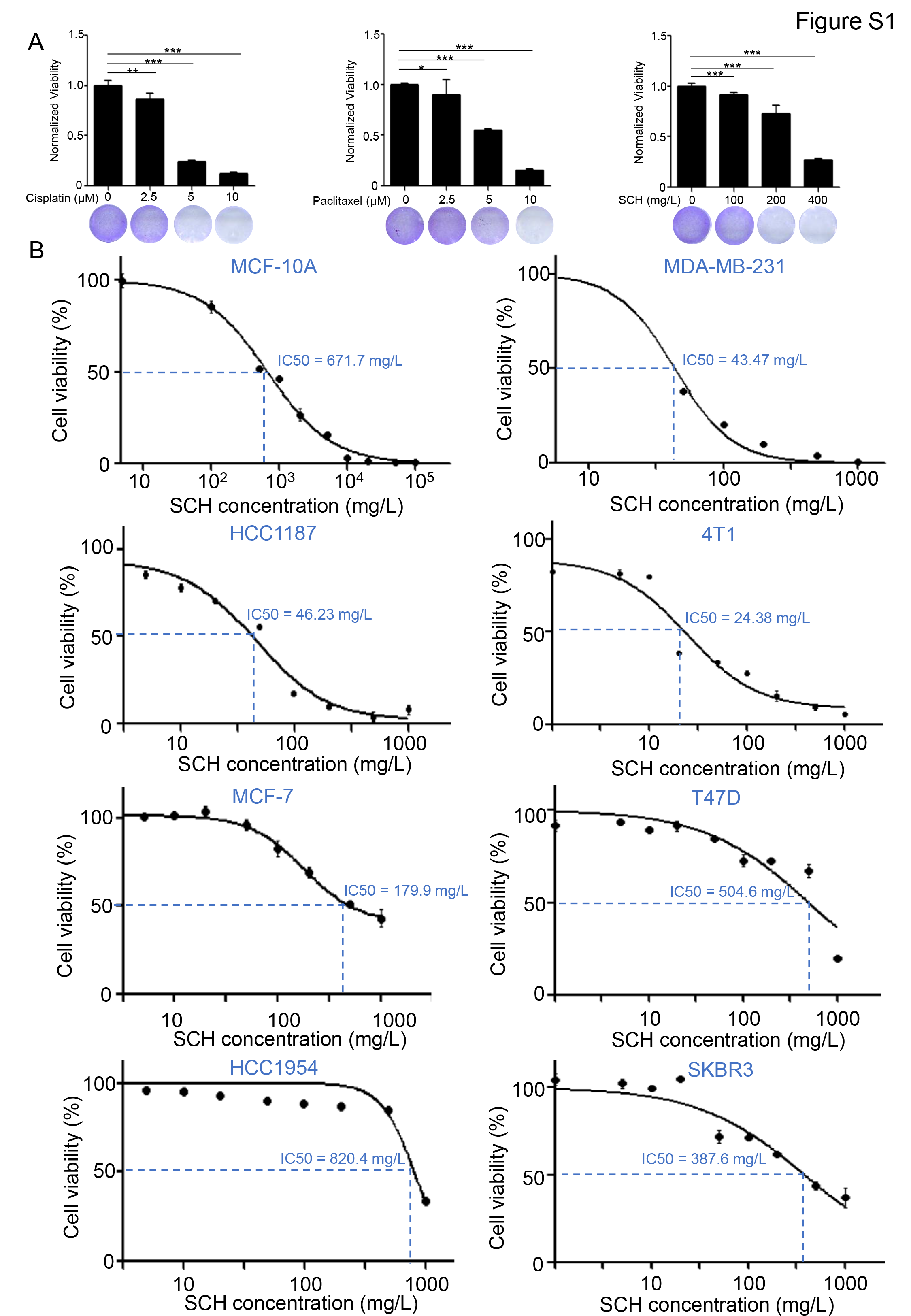

Supplement: Supplementary file 1 [file Image_1.tif]

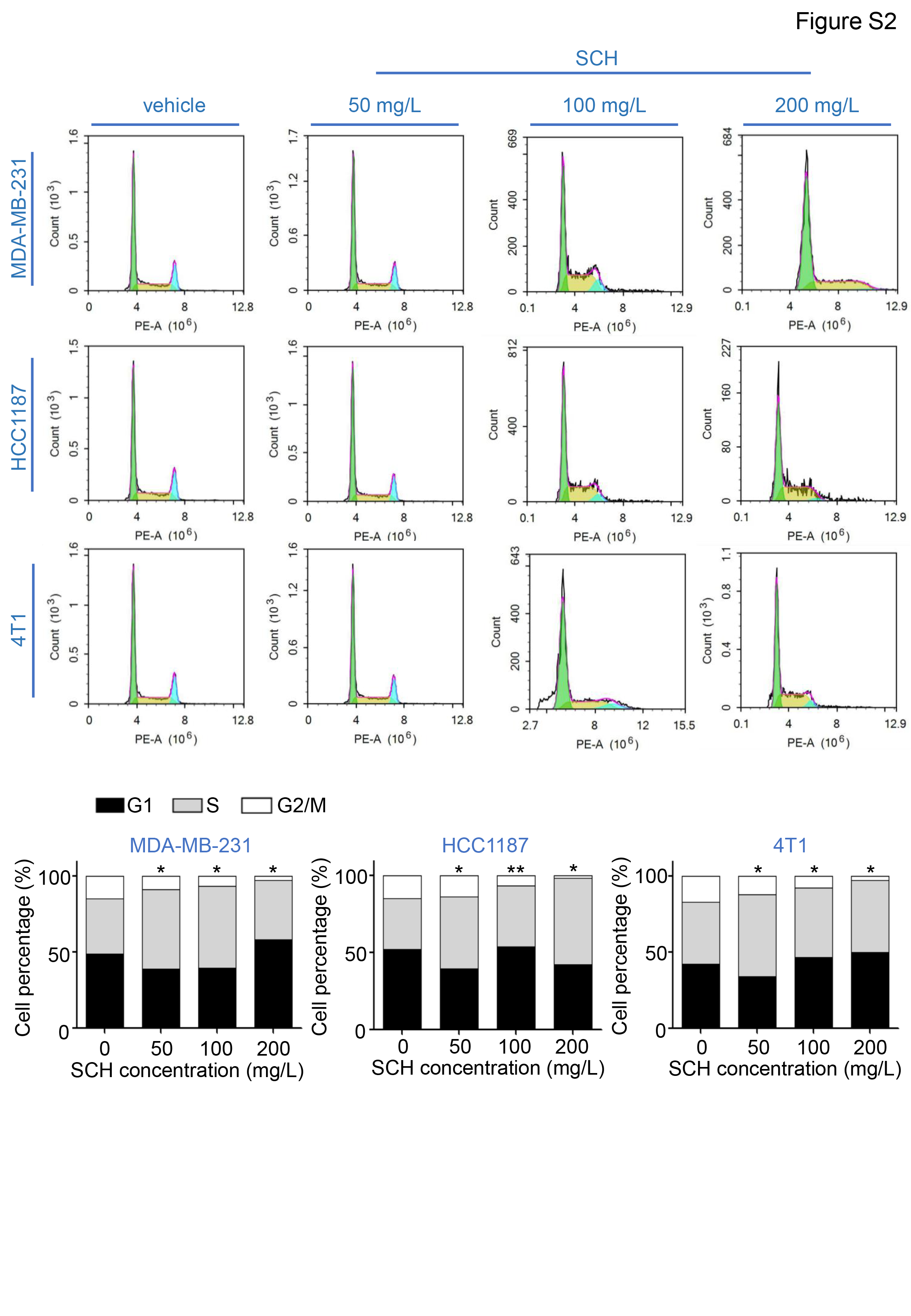

Supplement: Supplementary file 2 [file Image_2.tif]

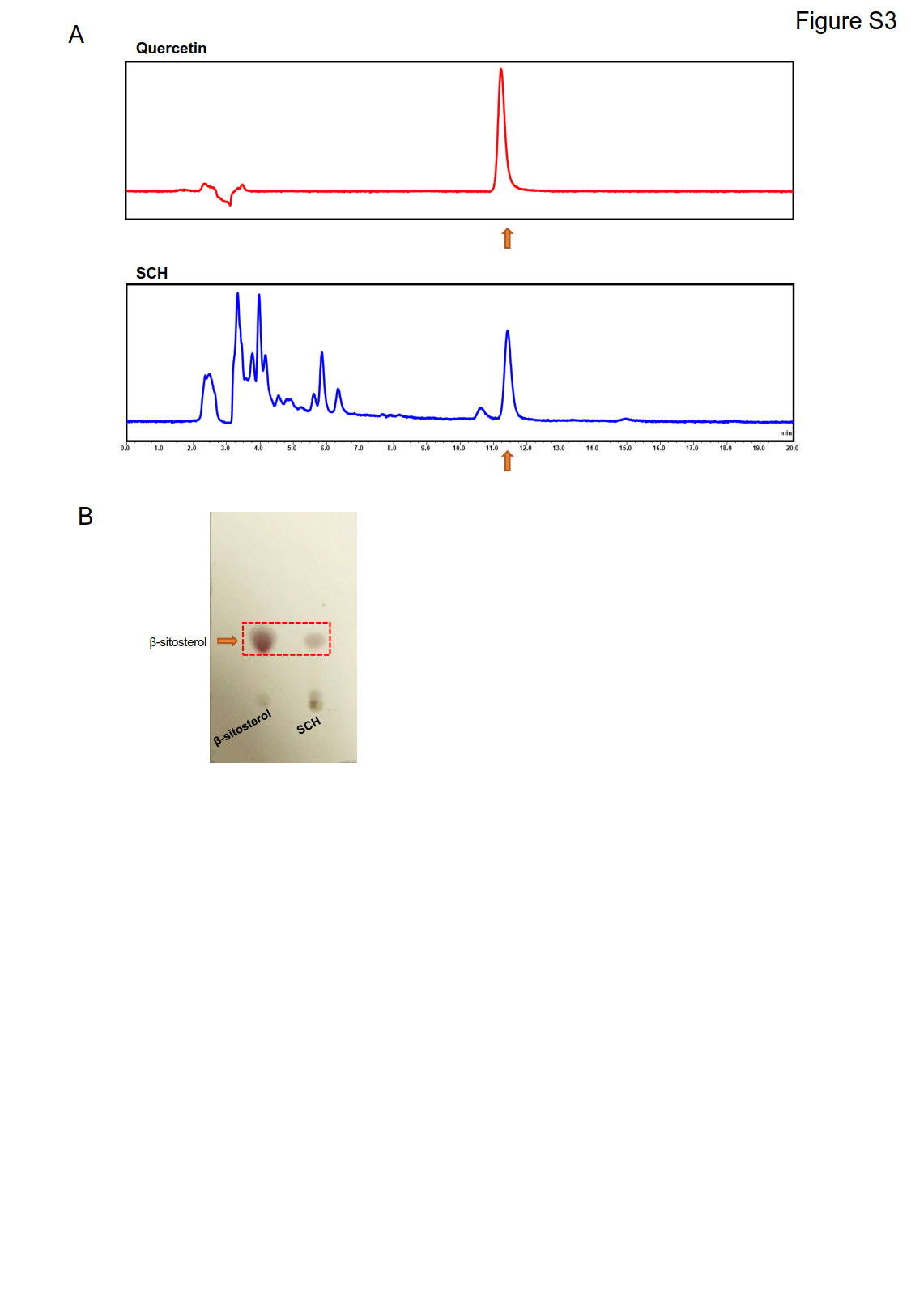

Supplement: Supplementary file 3 [file Image_3.tif]

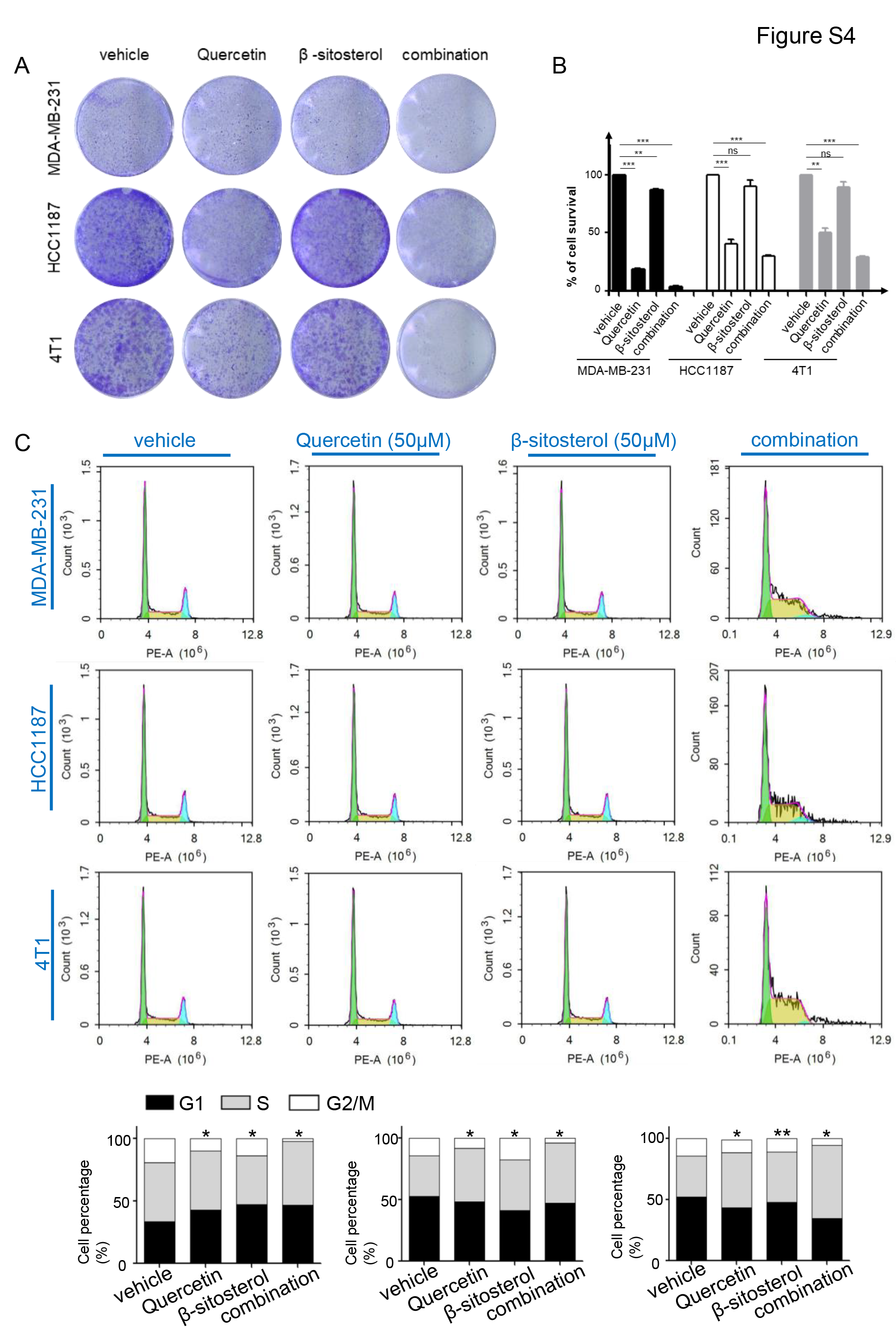

Supplement: Supplementary file 4 [file Image_4.tif]

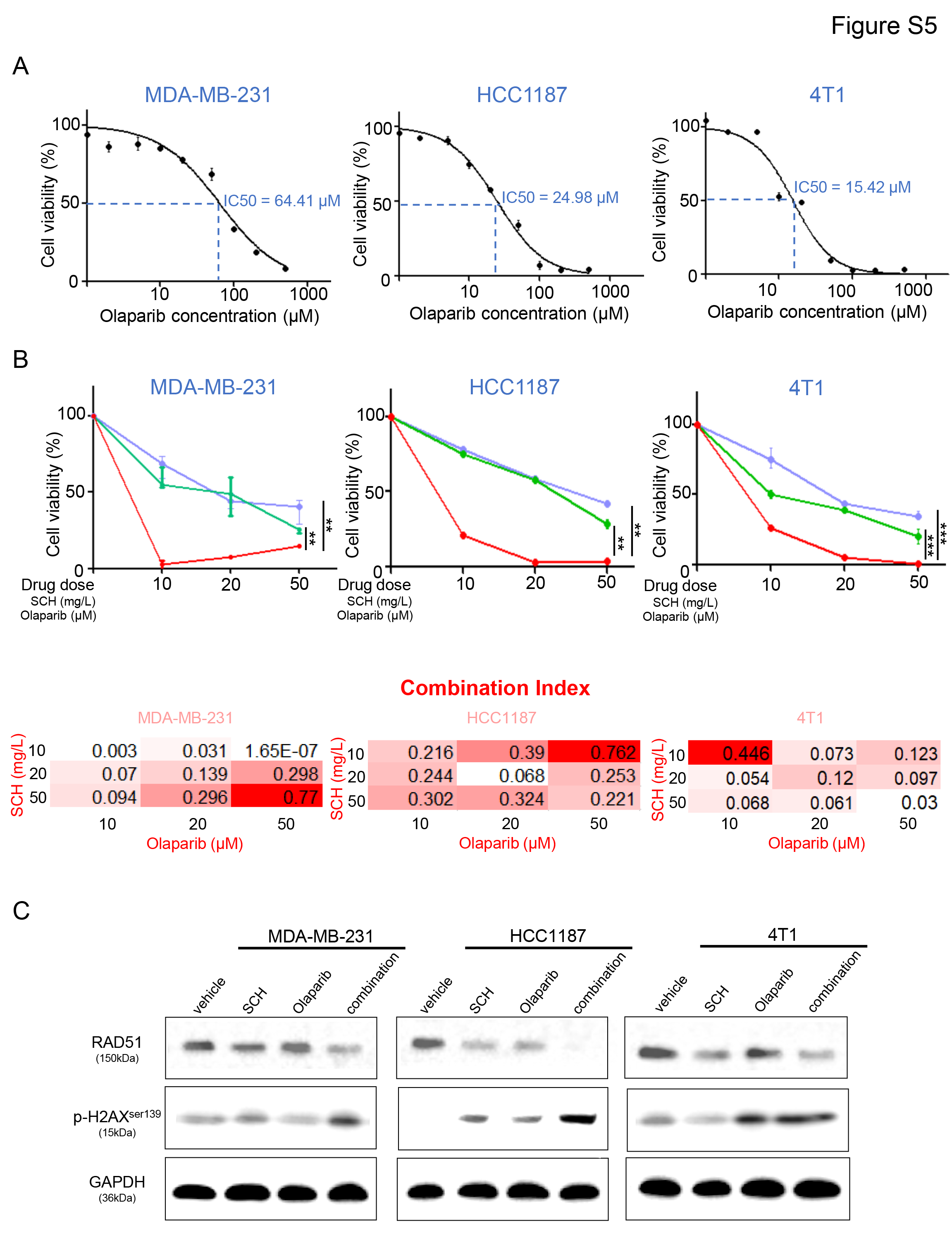

Supplement: Supplementary file 5 [file Image_5.tif]

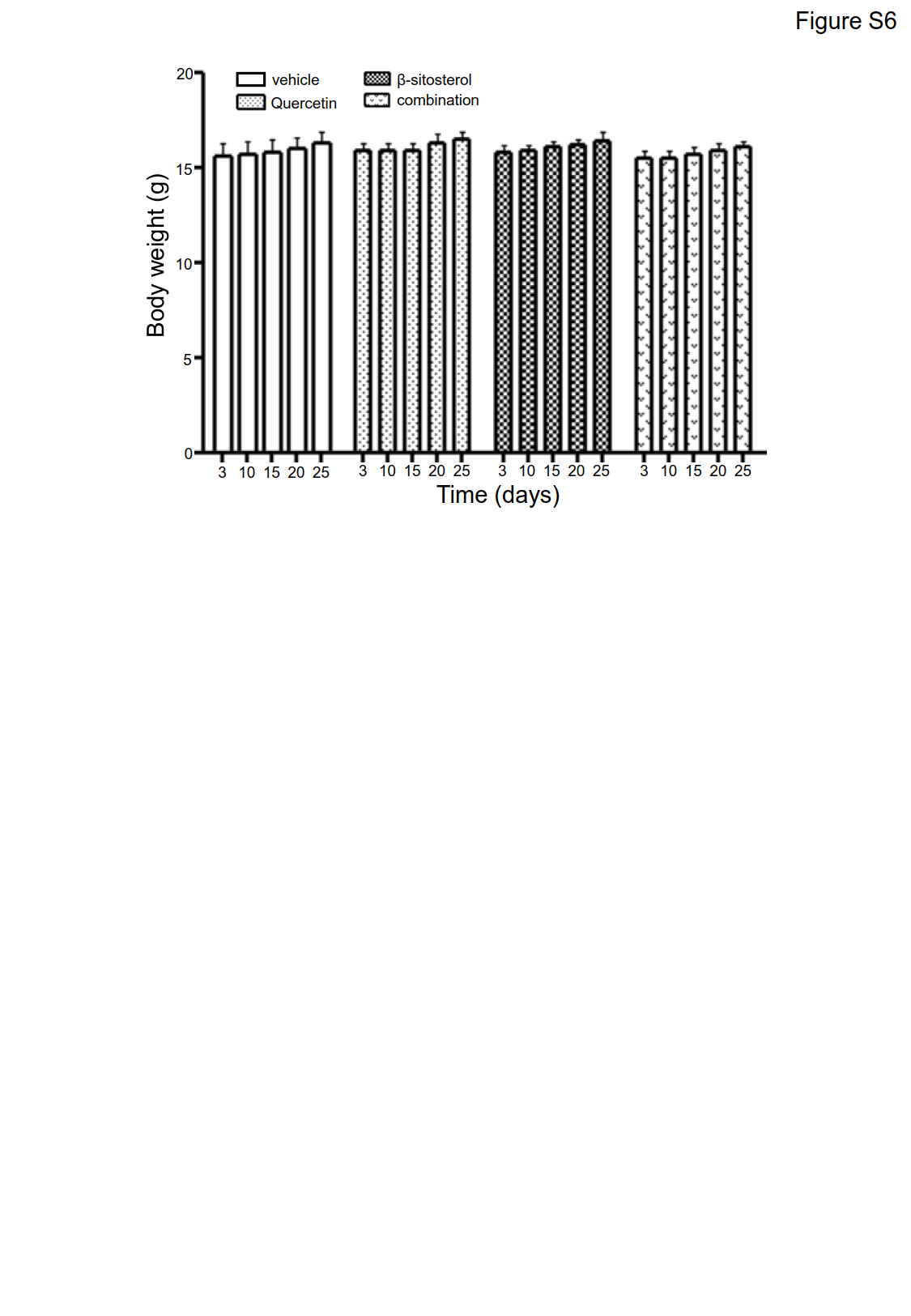

Supplement: Supplementary file 6 [file Image_6.tif]

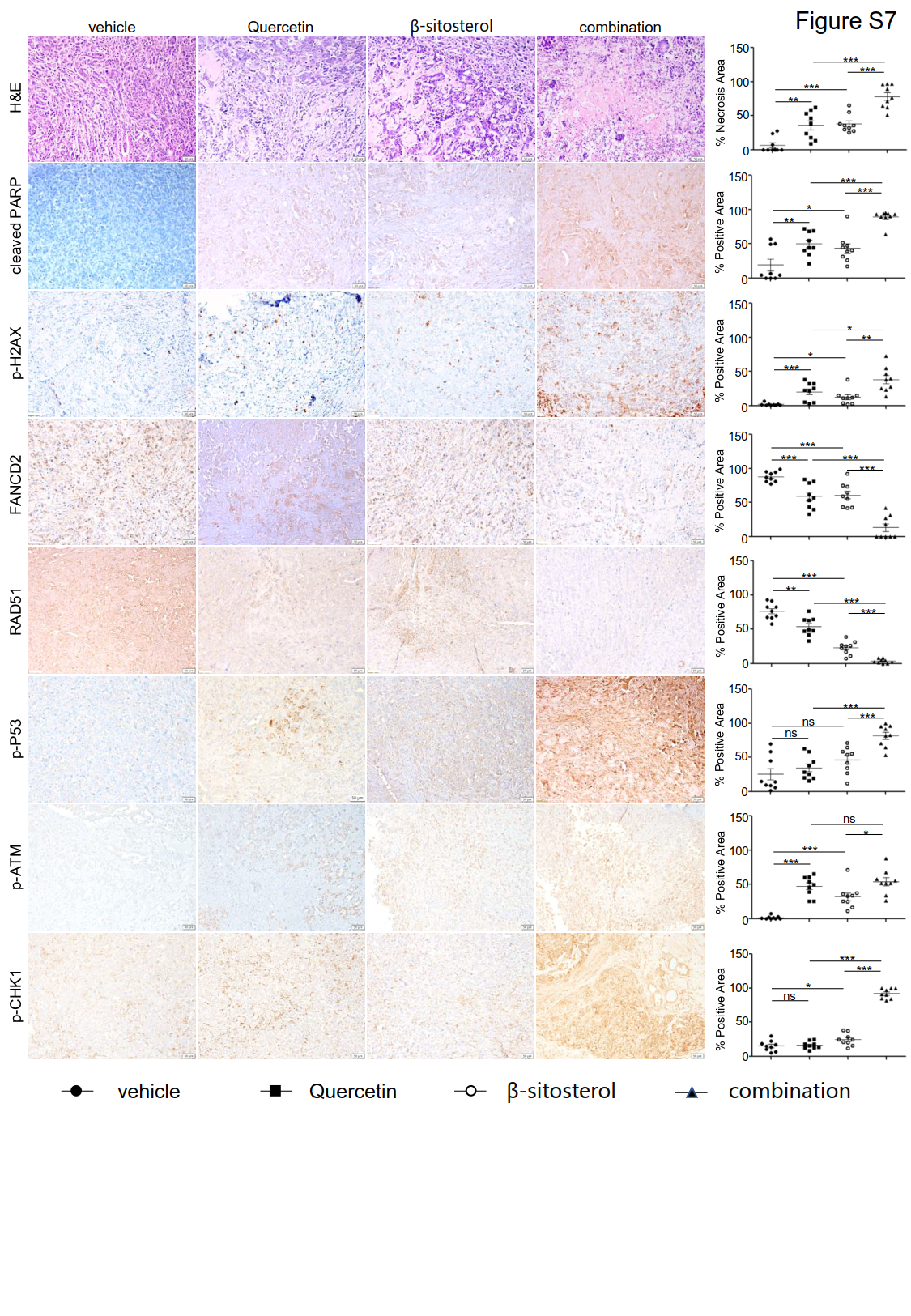

Supplement: Supplementary file 7 [file Image_7.tif]

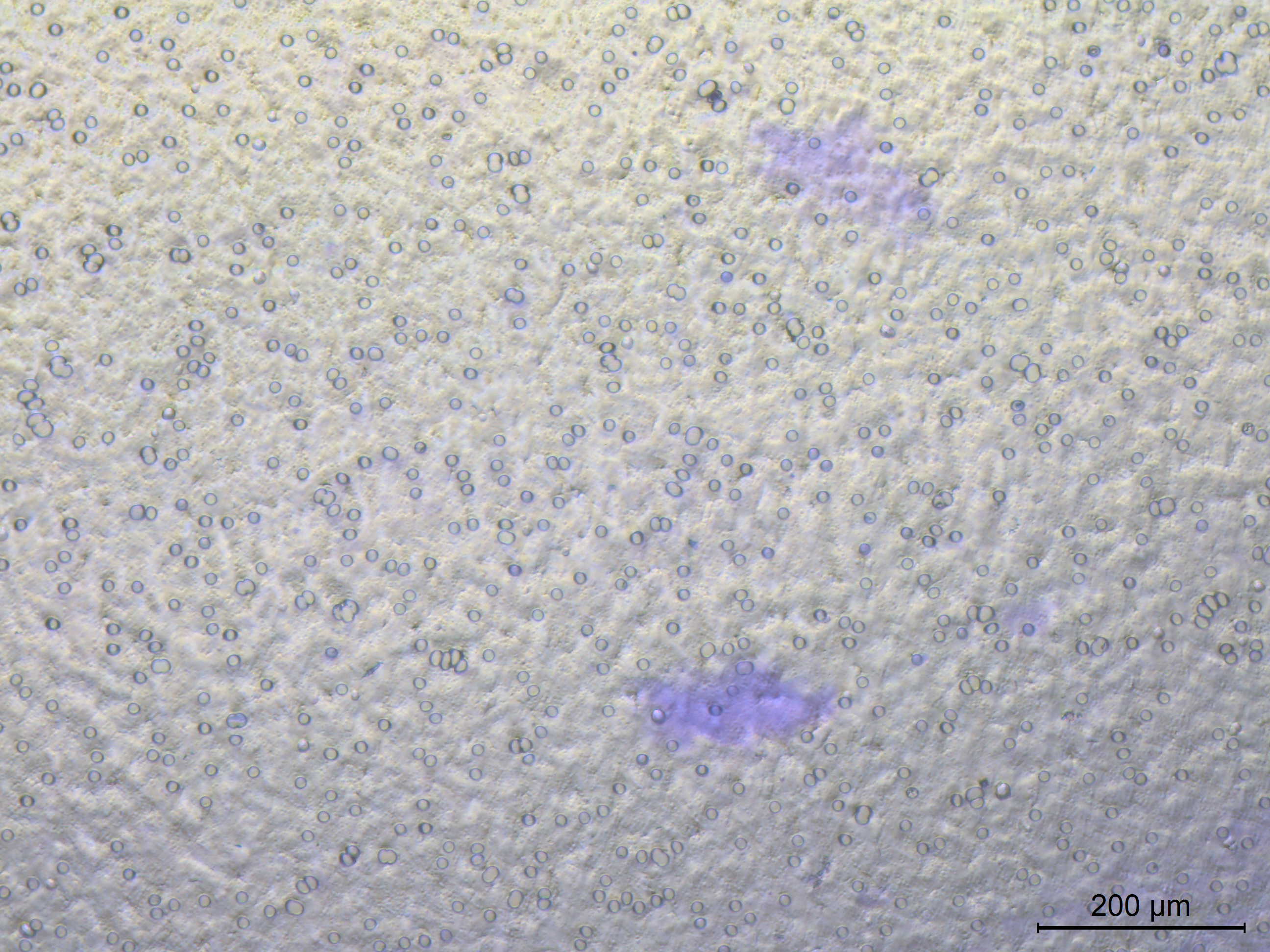

Supplement: Supplementary file 8 [file DataSheet_1.zip › Data Sheet 1/raw data-figure 1c-MDAMB231/fig.1c.MDAMB231_100.jpg]

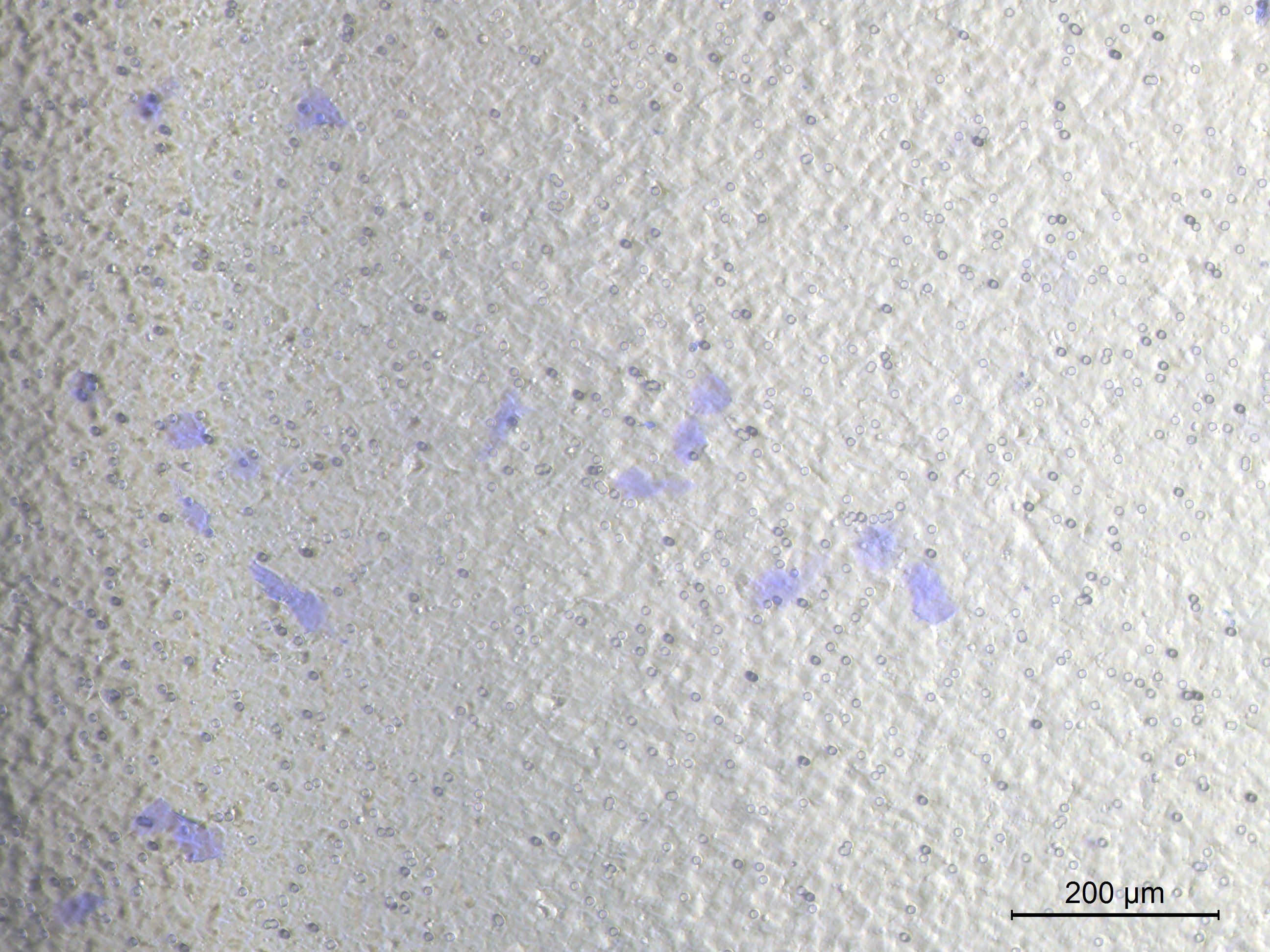

Supplement: Supplementary file 8 [file DataSheet_1.zip › Data Sheet 1/raw data-figure 1c-MDAMB231/fig.1c.MDAMB231_200.jpg]

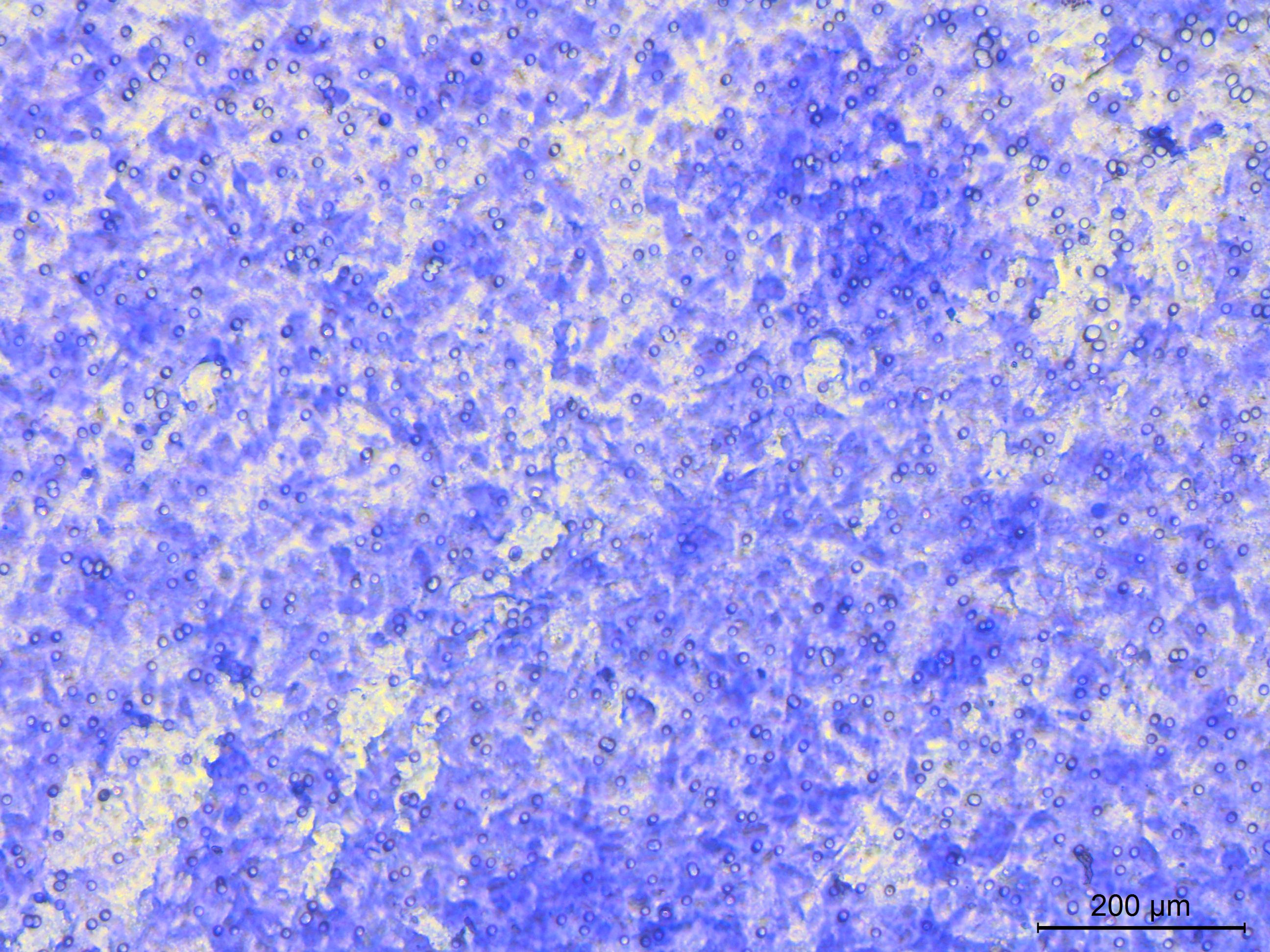

Supplement: Supplementary file 8 [file DataSheet_1.zip › Data Sheet 1/raw data-figure 1c-MDAMB231/fig.1c.MDAMB231_50.jpg]

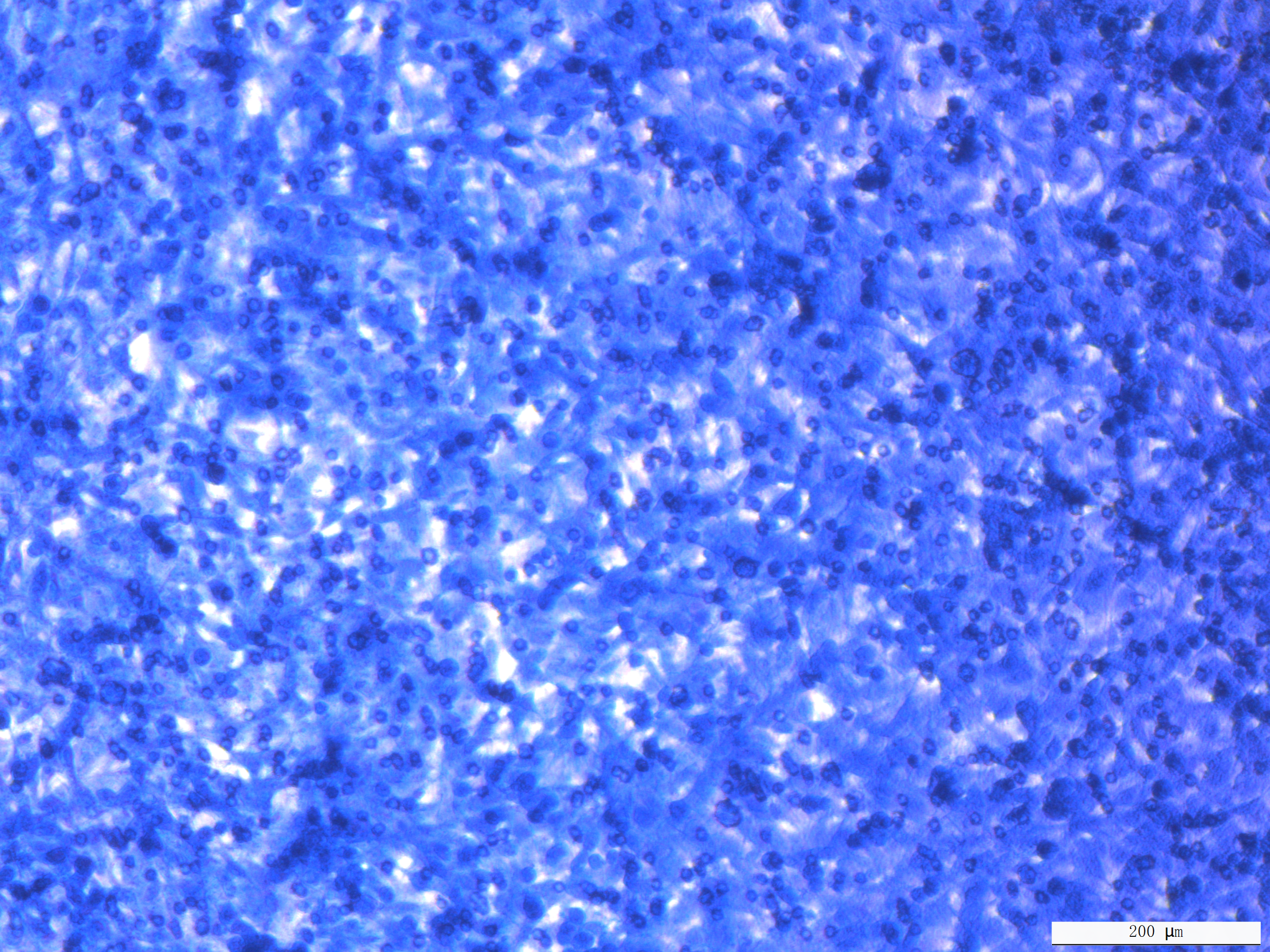

Supplement: Supplementary file 8 [file DataSheet_1.zip › Data Sheet 1/raw data-figure 1c-MDAMB231/fig.1c.MDAMB231_vehicle.tif]

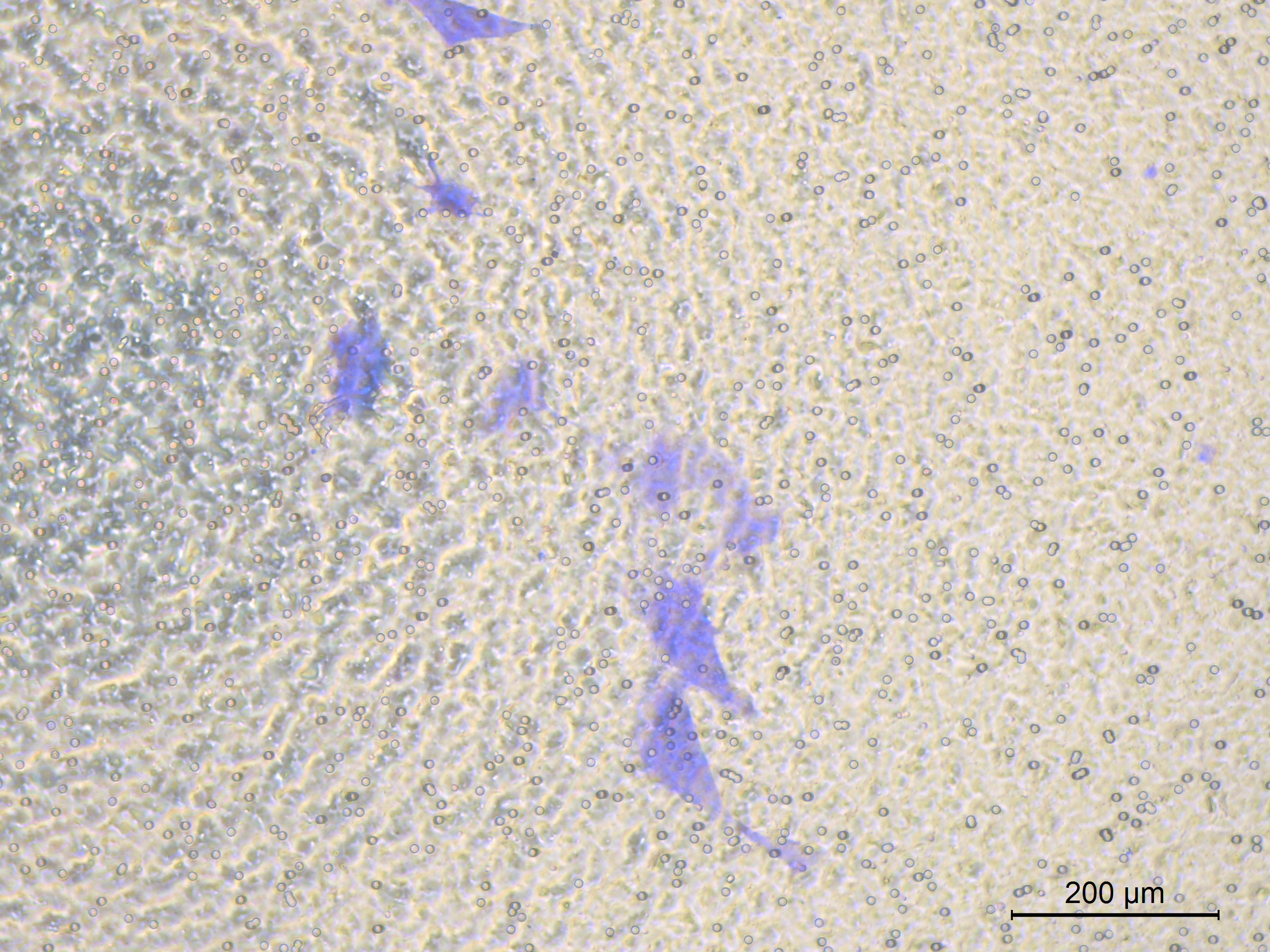

Supplement: Supplementary file 9 [file DataSheet_2.zip › raw data-figure 1c-HCC1187/raw data-fig.1c.HCC1187_100.jpg]

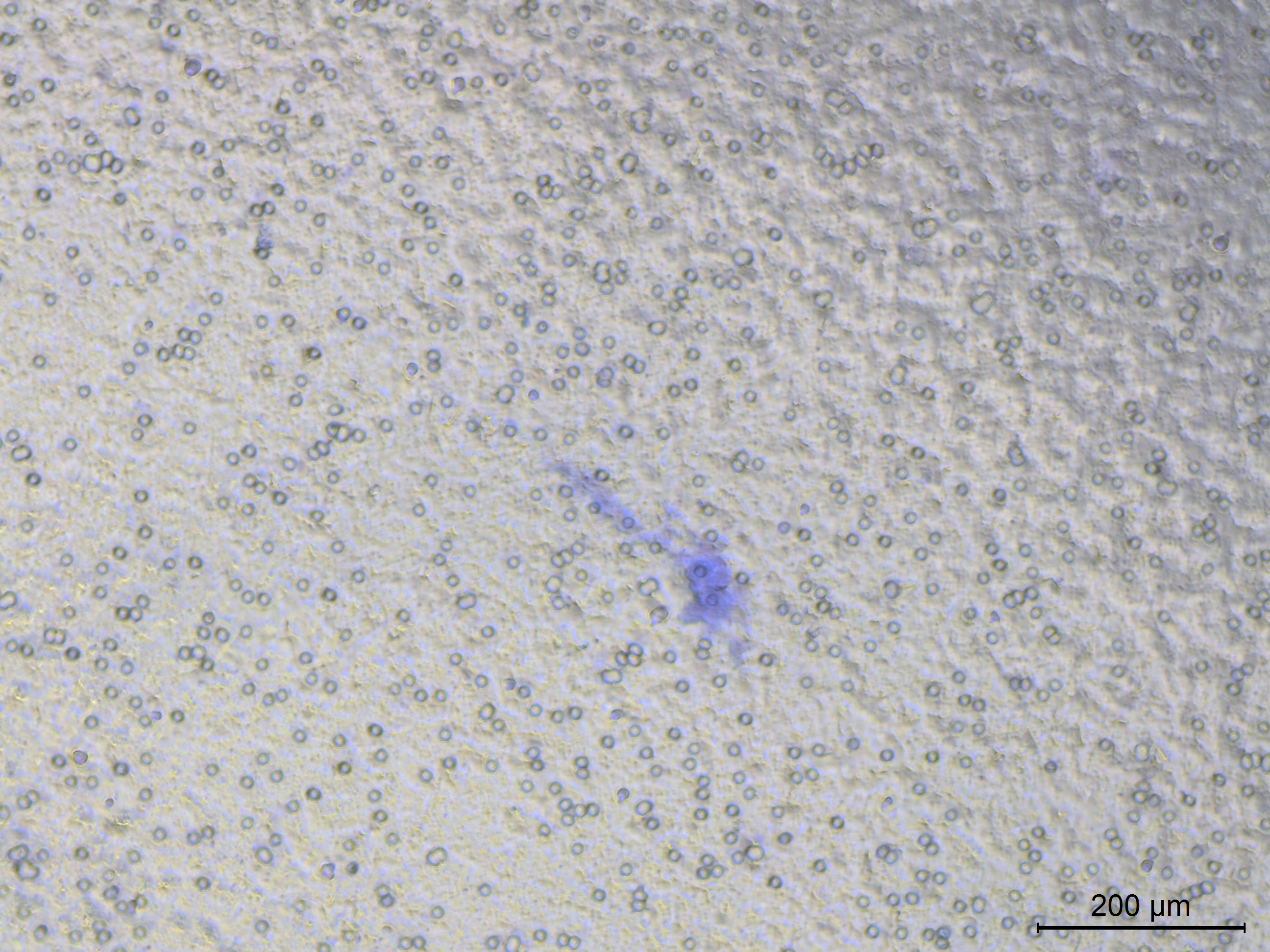

Supplement: Supplementary file 9 [file DataSheet_2.zip › raw data-figure 1c-HCC1187/raw data-fig.1c.HCC1187_200.jpg]

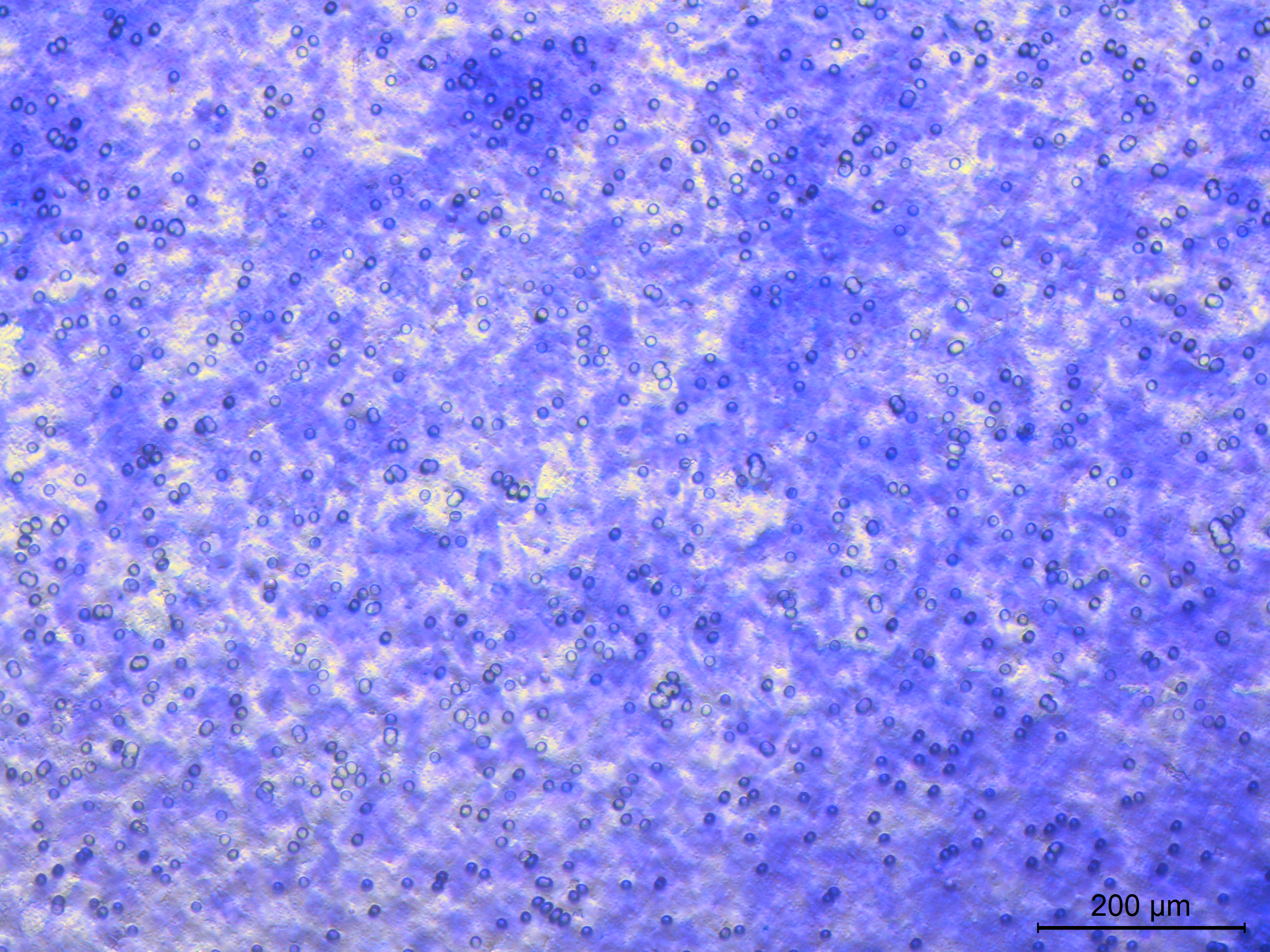

Supplement: Supplementary file 9 [file DataSheet_2.zip › raw data-figure 1c-HCC1187/raw data-fig.1c.HCC1187_50.jpg]

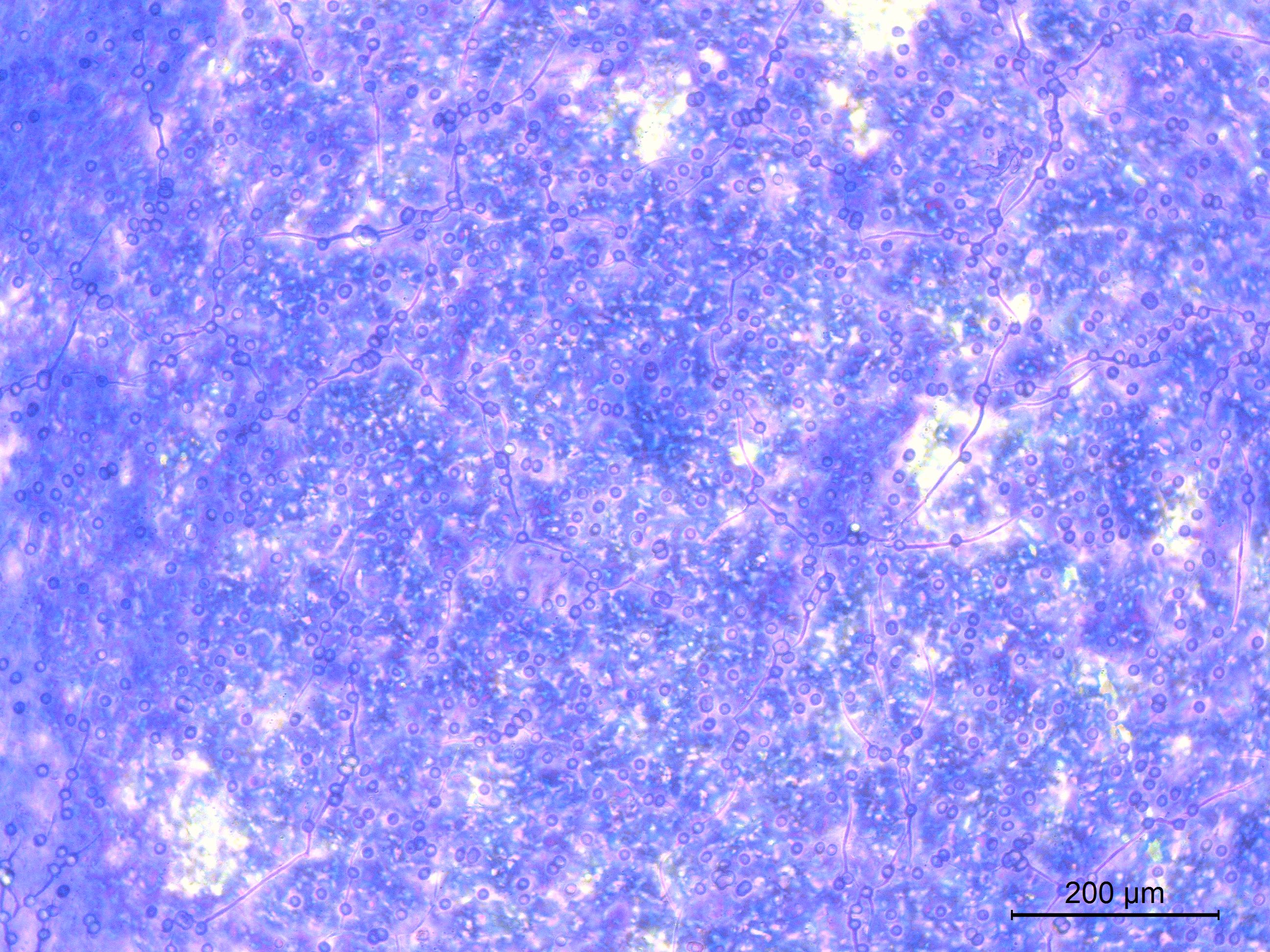

Supplement: Supplementary file 9 [file DataSheet_2.zip › raw data-figure 1c-HCC1187/raw data-fig.1c.HCC1187_vehicle.jpg]

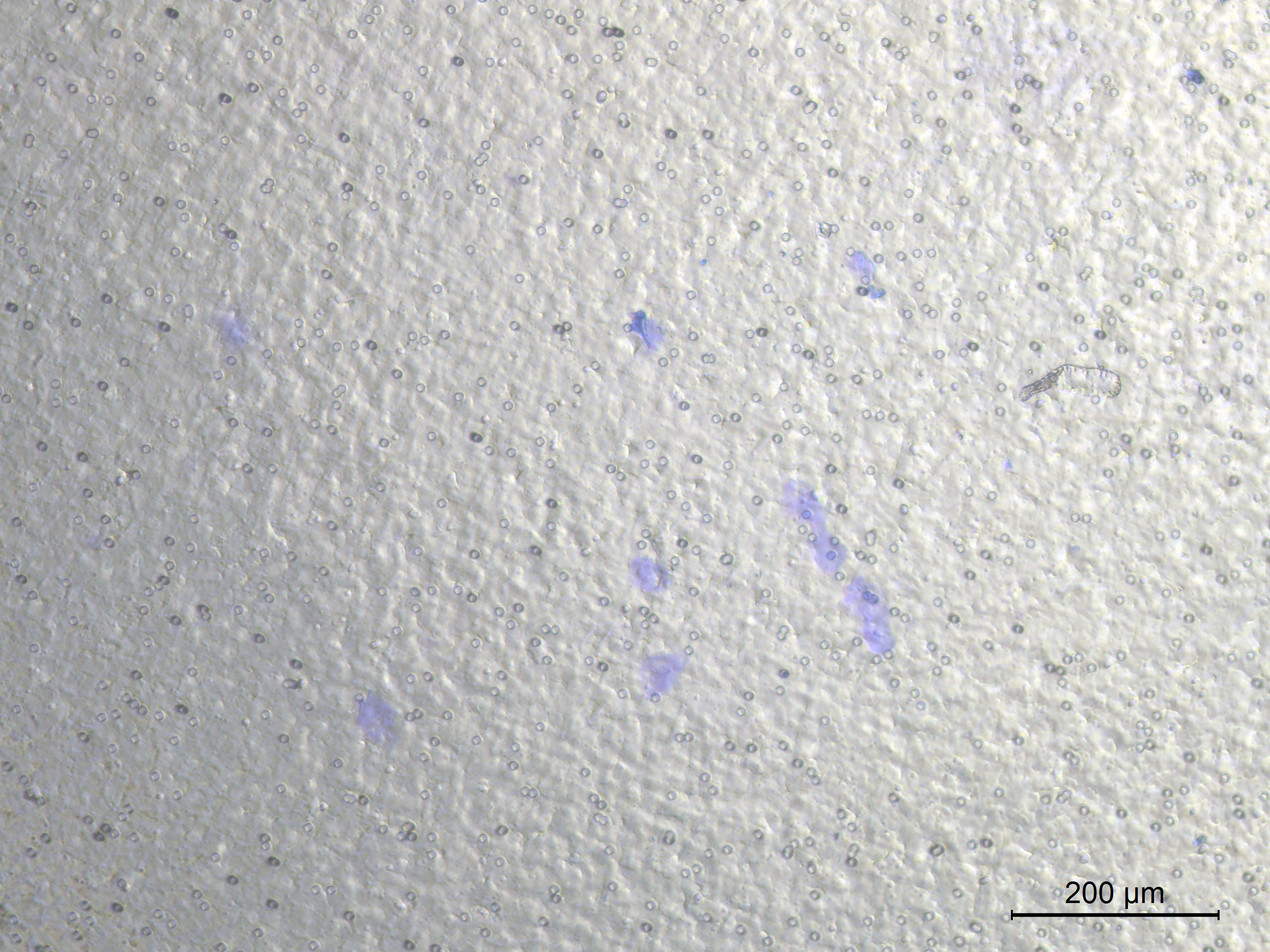

Supplement: Supplementary file 11 [file DataSheet_4.zip › Data Sheet 4/raw data-figure 1d-MDAMB231/fig.1d.MDAMB231_100.tif]

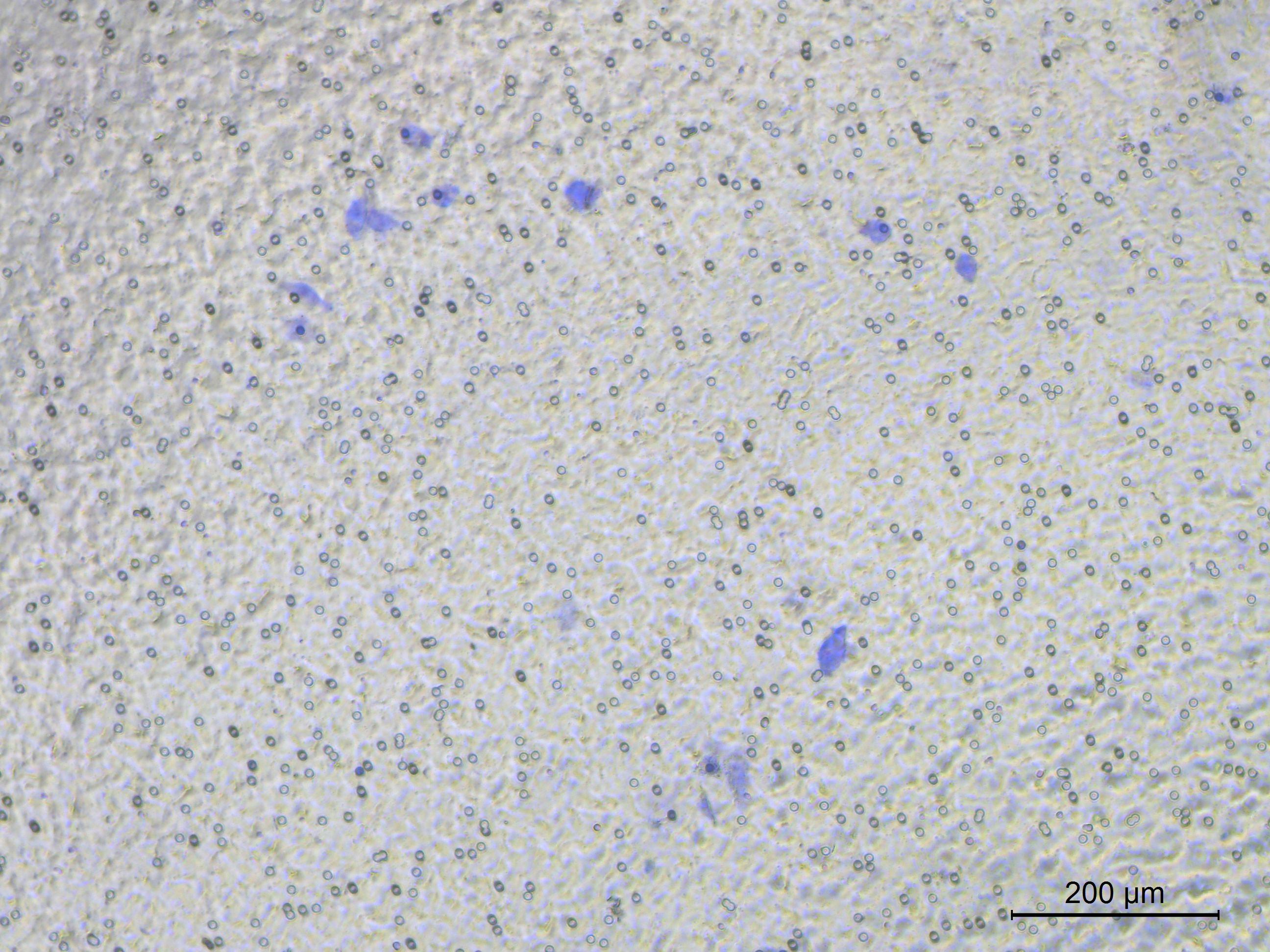

Supplement: Supplementary file 11 [file DataSheet_4.zip › Data Sheet 4/raw data-figure 1d-MDAMB231/fig.1d.MDAMB231_200.jpg]

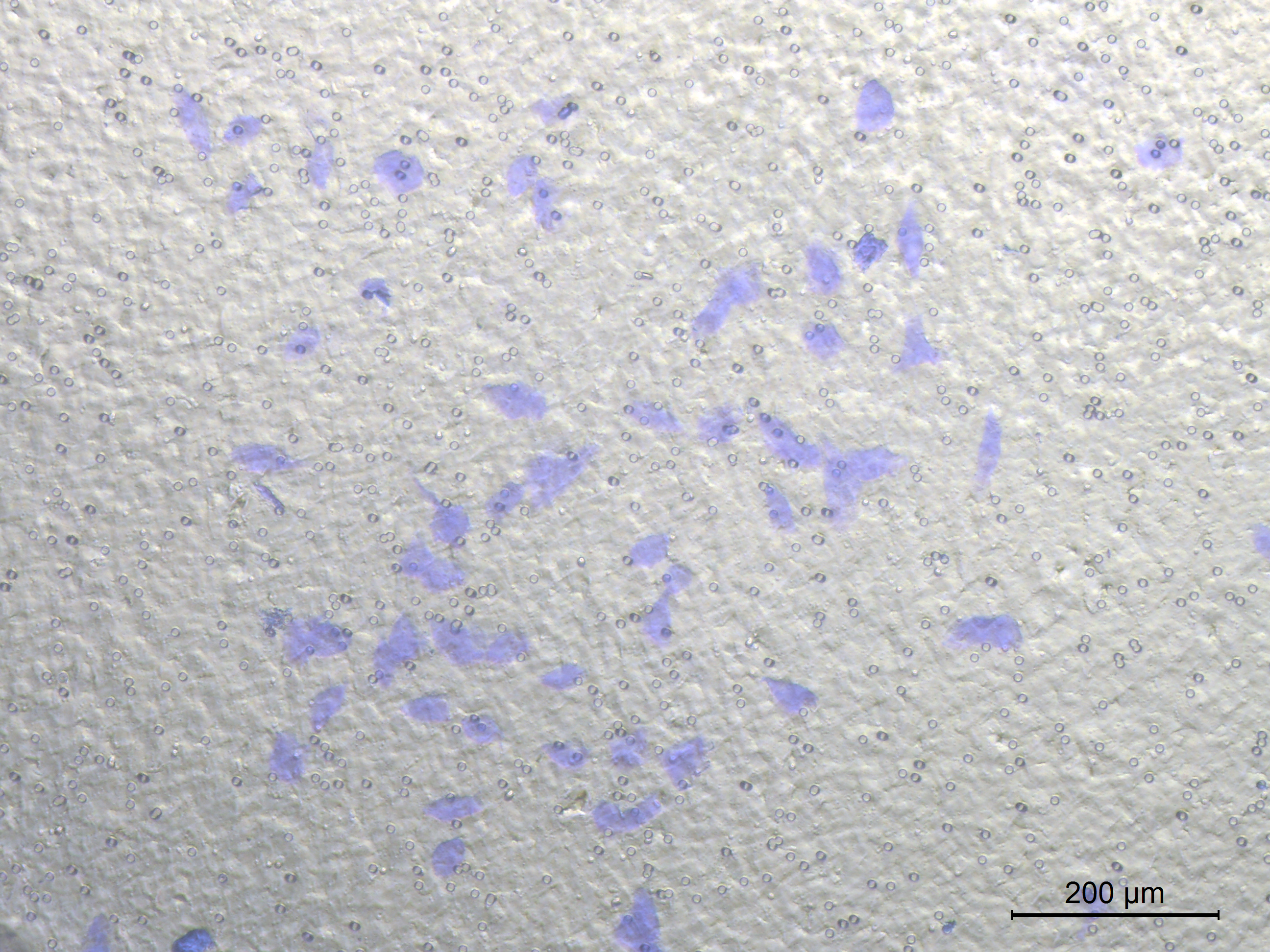

Supplement: Supplementary file 11 [file DataSheet_4.zip › Data Sheet 4/raw data-figure 1d-MDAMB231/fig.1d.MDAMB231_50.tif]

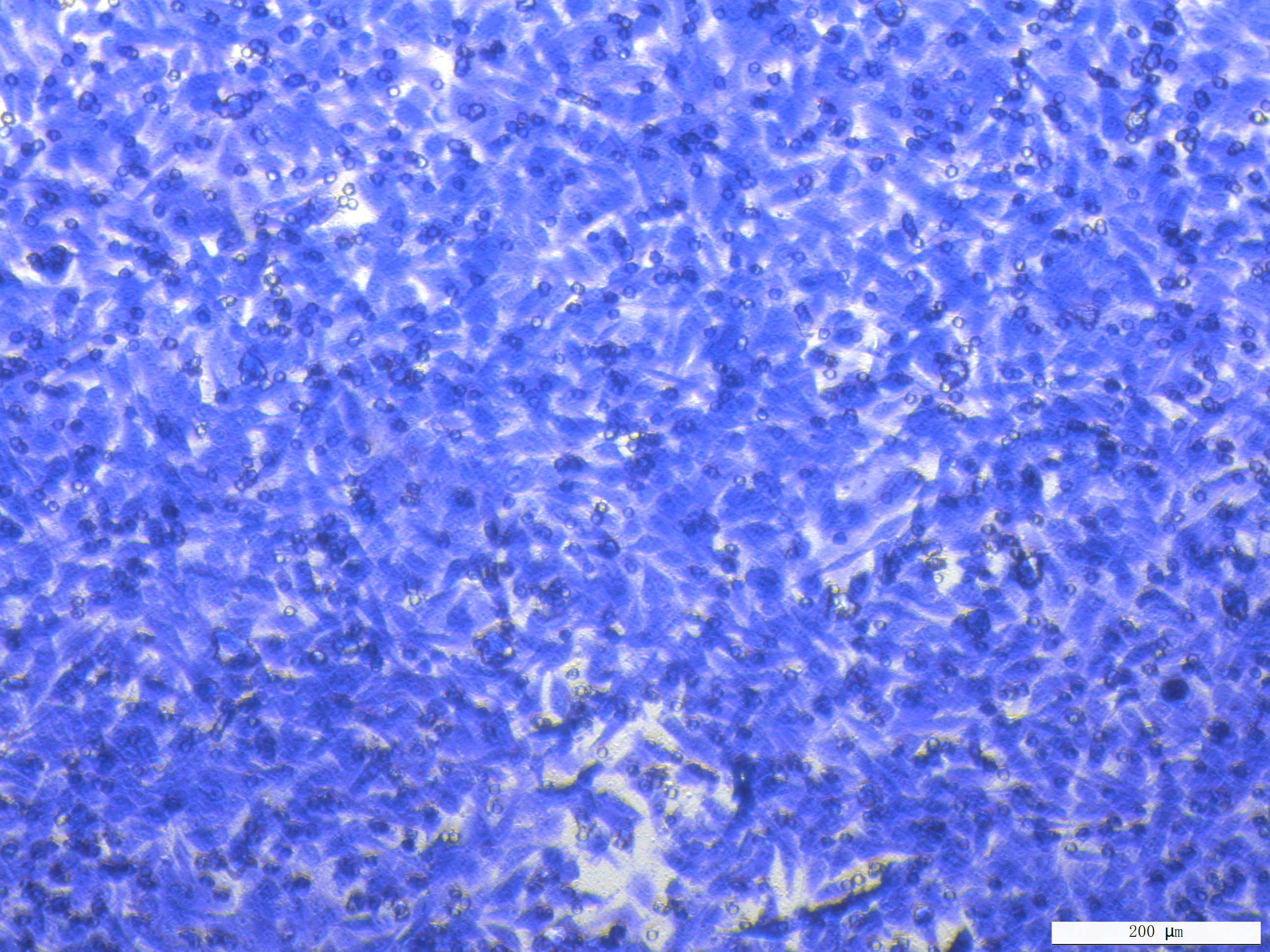

Supplement: Supplementary file 11 [file DataSheet_4.zip › Data Sheet 4/raw data-figure 1d-MDAMB231/fig.1d.MDAMB231_vehicle.tif]

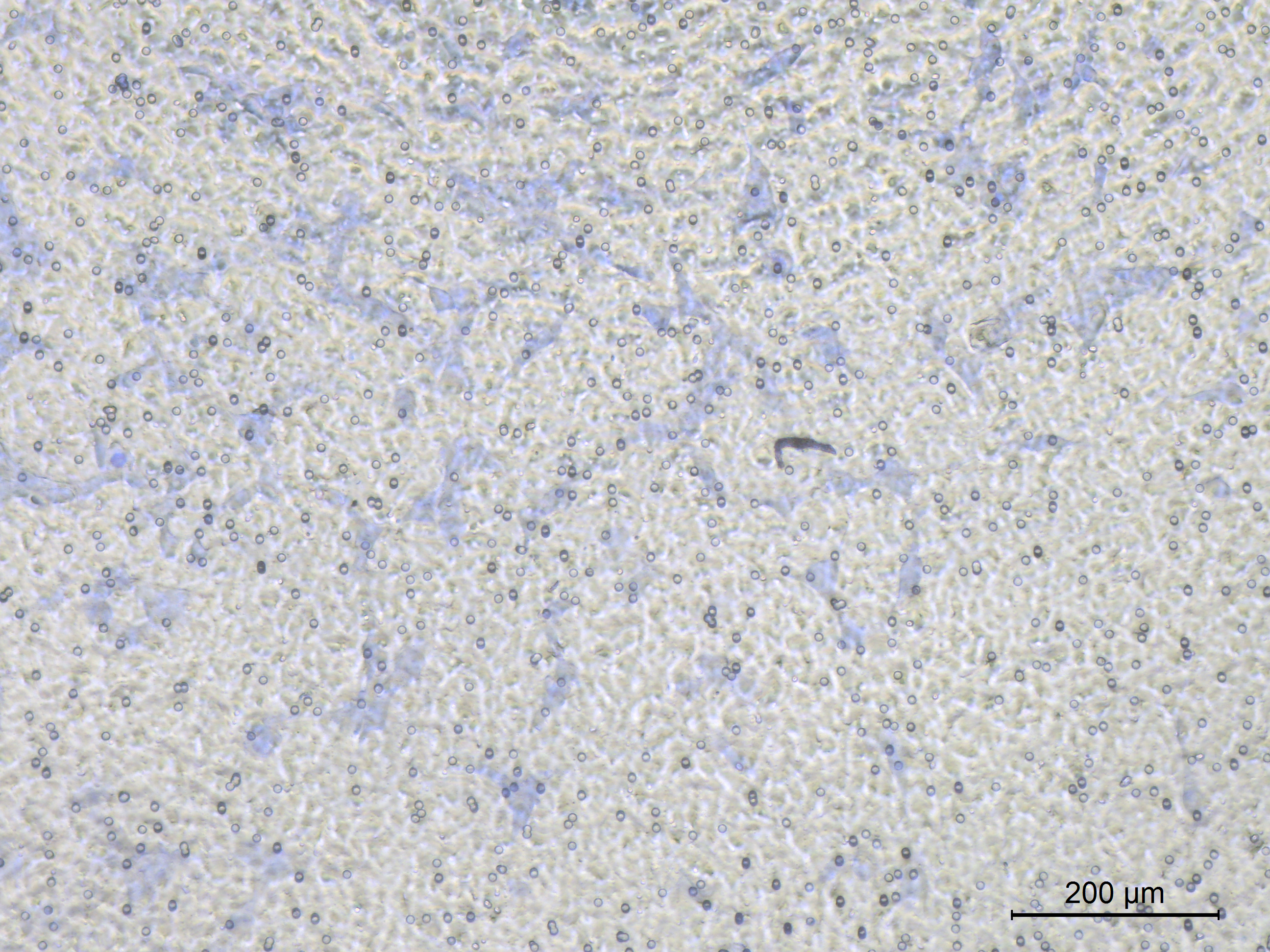

Supplement: Supplementary file 12 [file DataSheet_5.zip › raw data-figure 1d-HCC1187/fig.1d.HCC1187_100.tif]

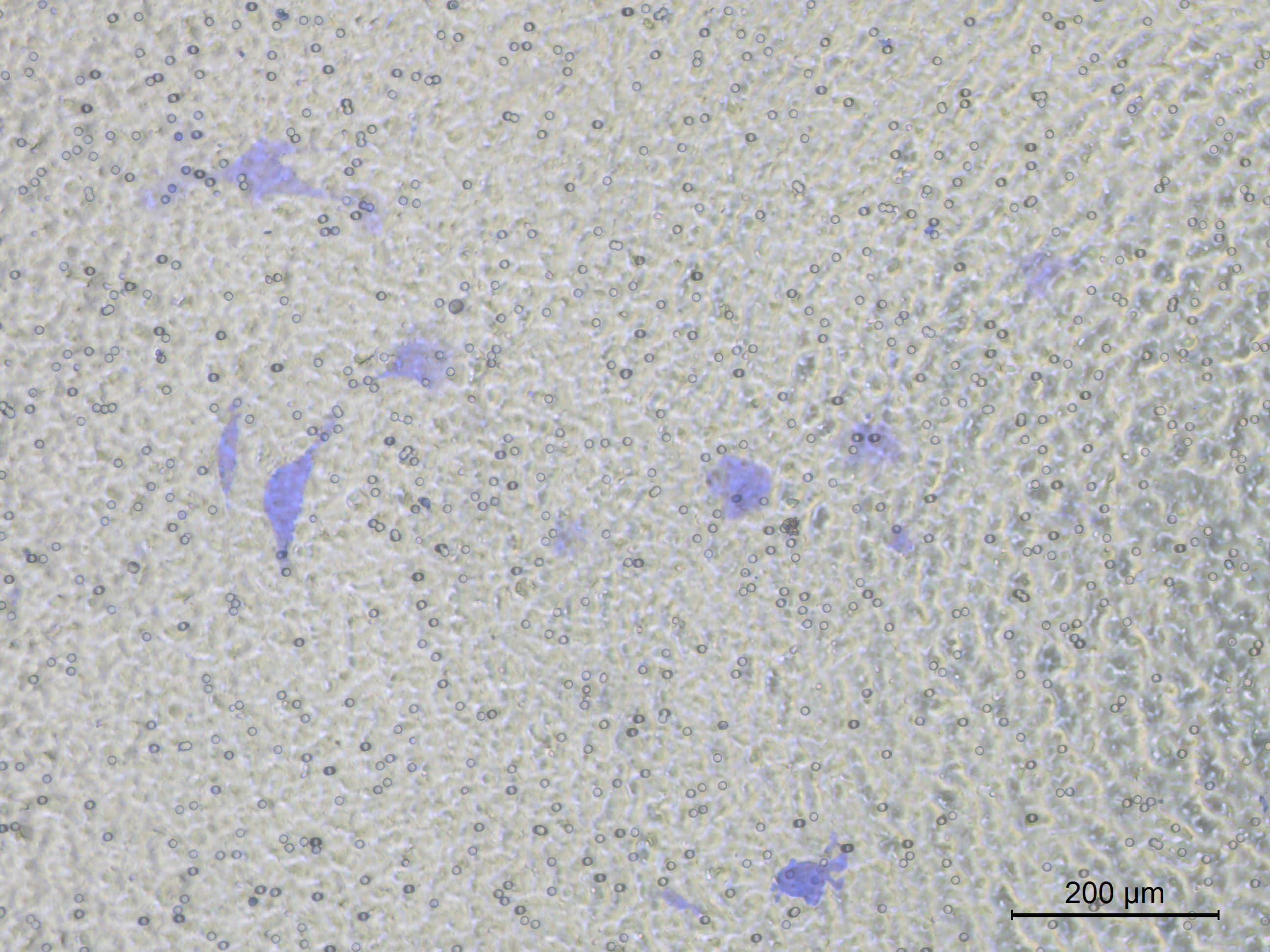

Supplement: Supplementary file 12 [file DataSheet_5.zip › raw data-figure 1d-HCC1187/fig.1d.HCC1187_200.tif]

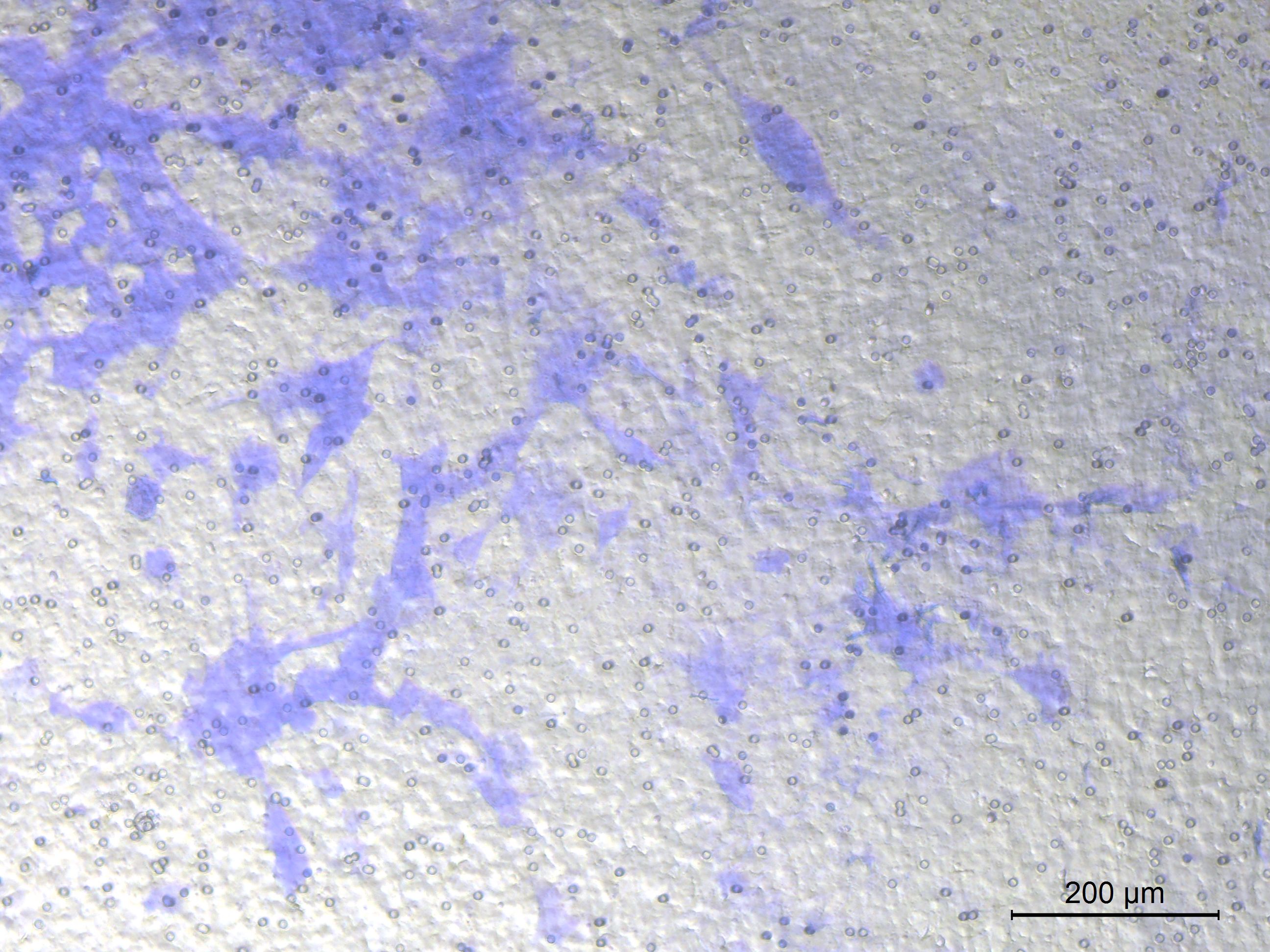

Supplement: Supplementary file 12 [file DataSheet_5.zip › raw data-figure 1d-HCC1187/fig.1d.HCC1187_50.jpg]

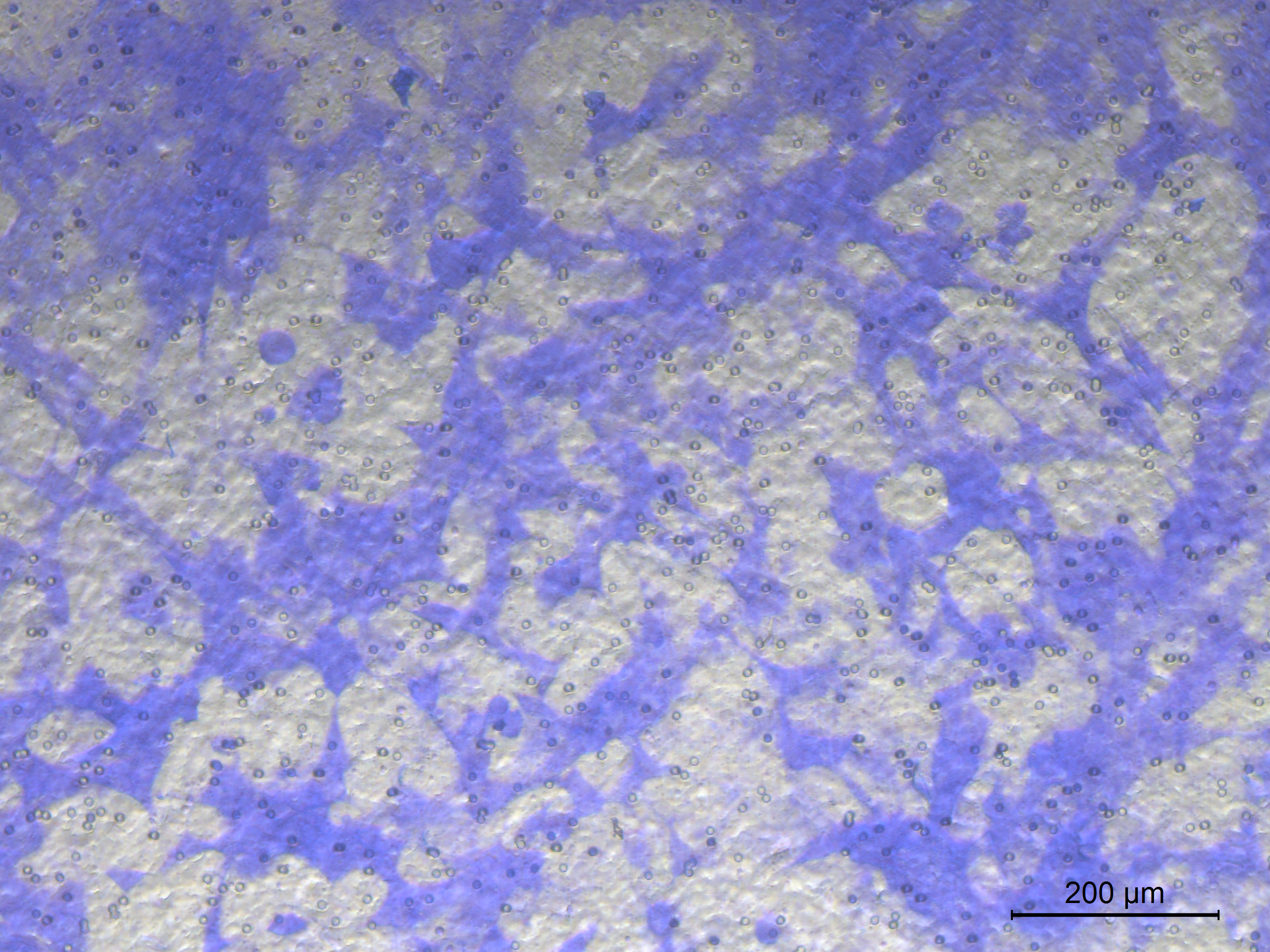

Supplement: Supplementary file 12 [file DataSheet_5.zip › raw data-figure 1d-HCC1187/fig.1d.HCC1187_vehicle.tif]

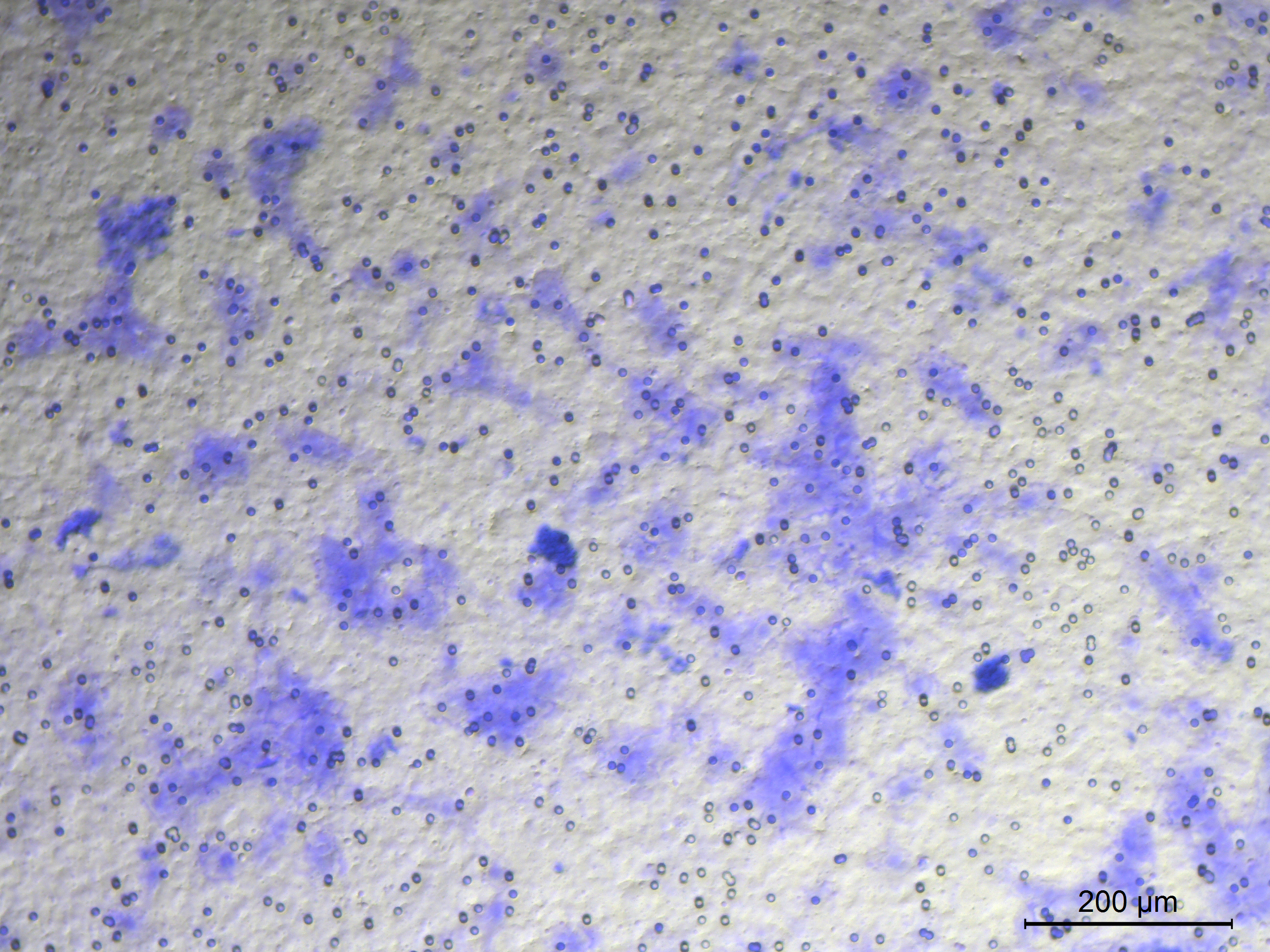

Supplement: Supplementary file 13 [file DataSheet_6.zip › raw data-figure 1d-4T1/fig.1d.4T1_100.jpg]

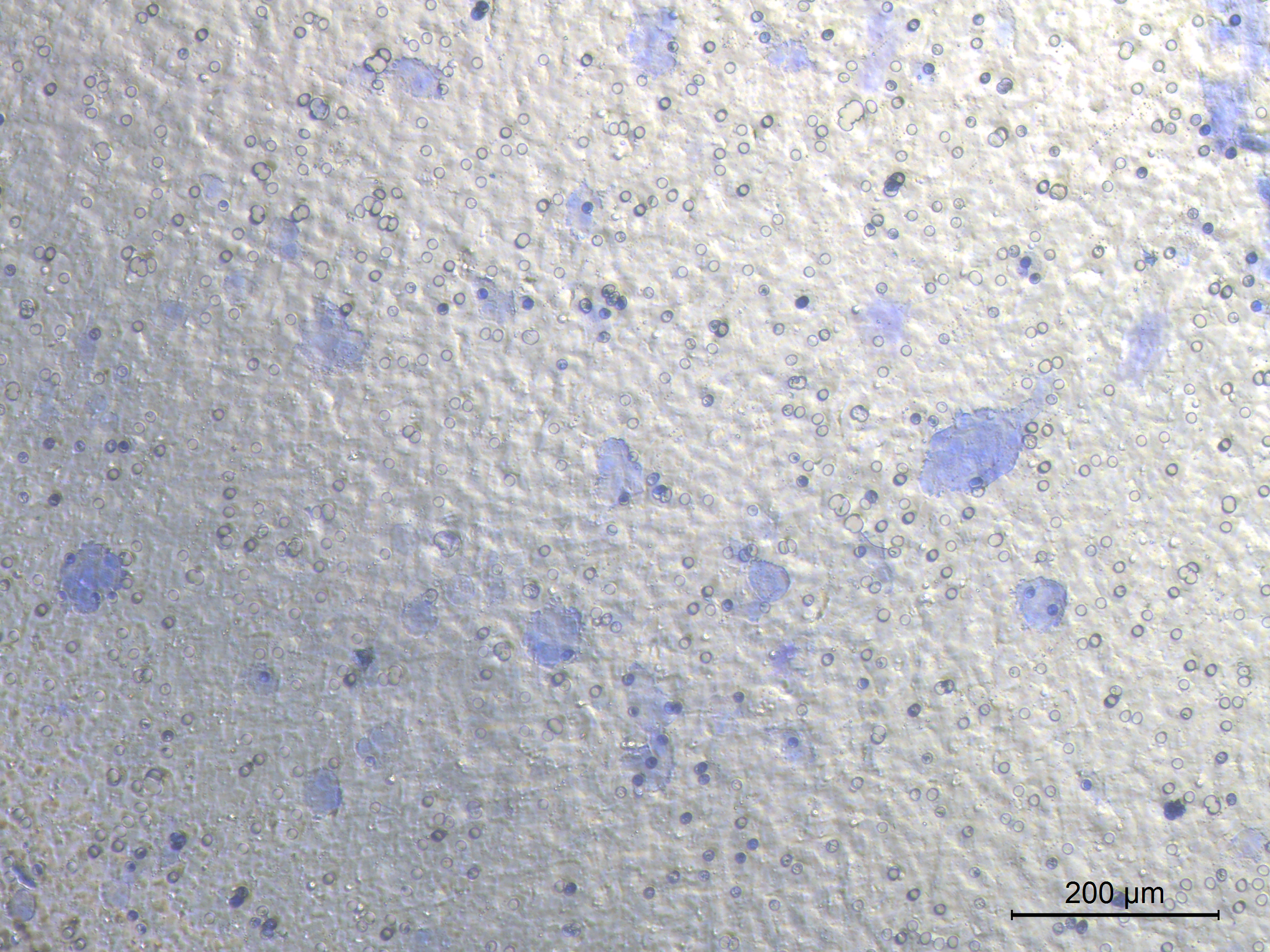

Supplement: Supplementary file 13 [file DataSheet_6.zip › raw data-figure 1d-4T1/fig.1d.4T1_200.jpg]

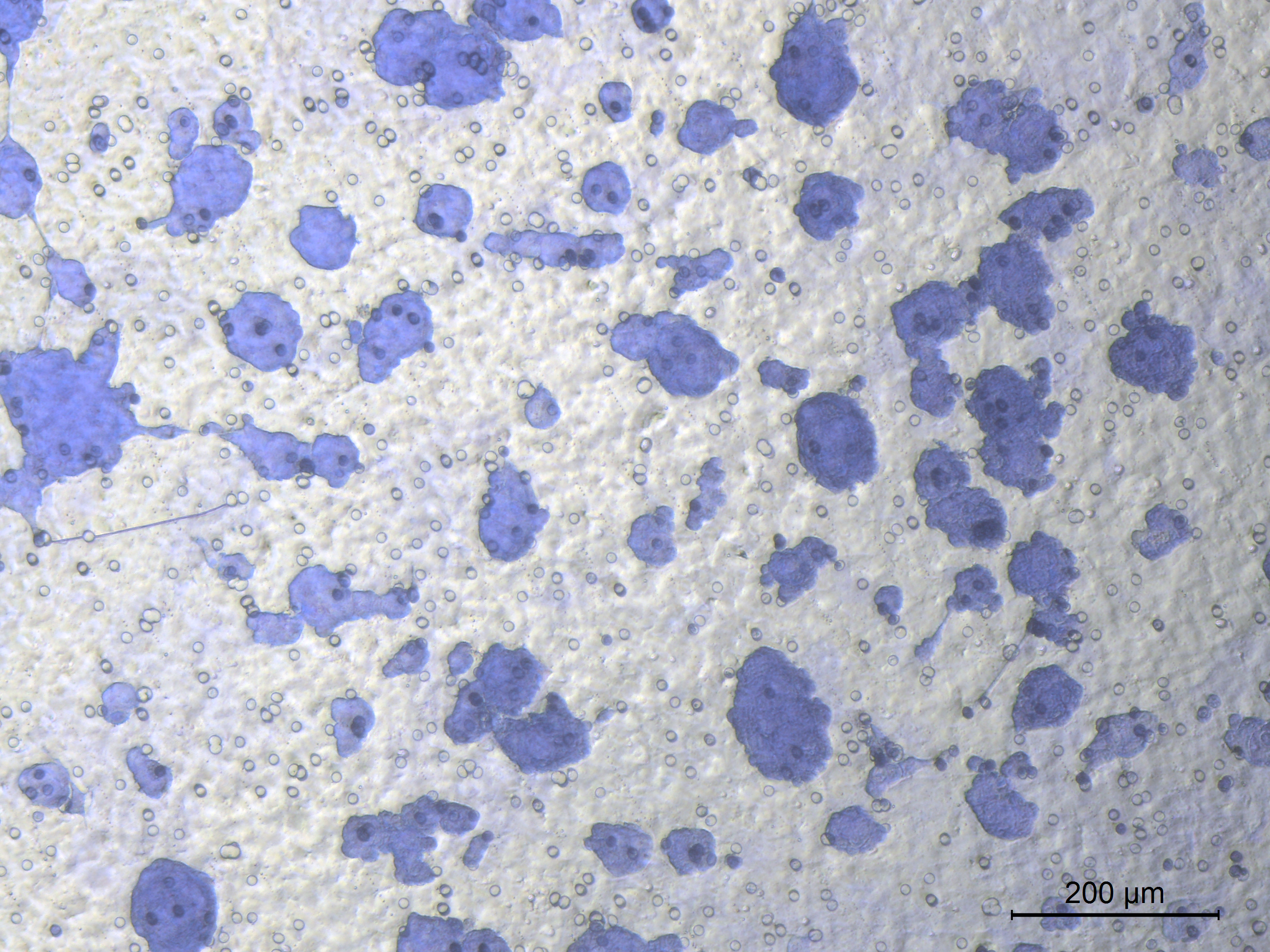

Supplement: Supplementary file 13 [file DataSheet_6.zip › raw data-figure 1d-4T1/fig.1d.4T1_50.jpg]

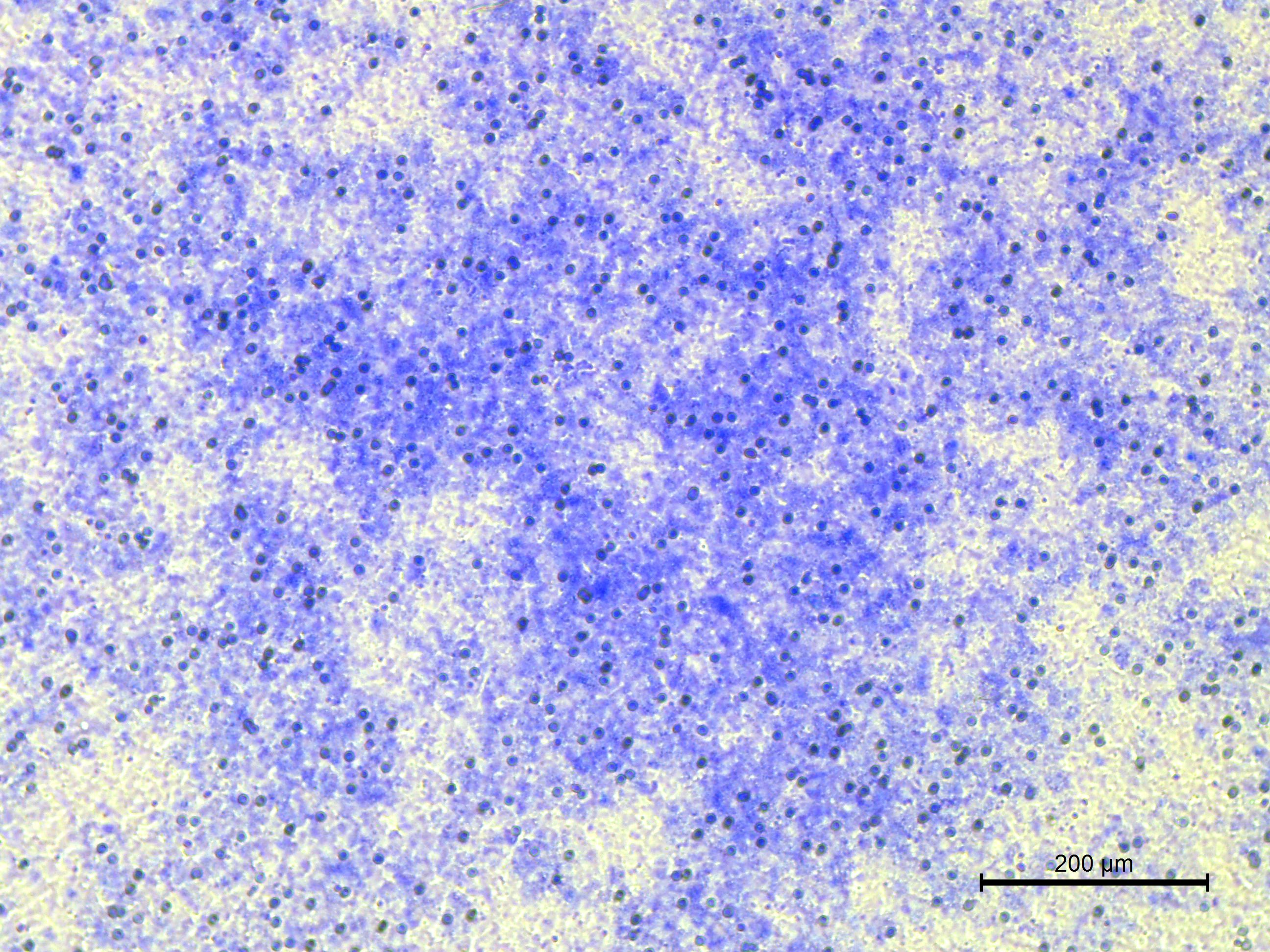

Supplement: Supplementary file 13 [file DataSheet_6.zip › raw data-figure 1d-4T1/fig.1d.4T1_vehicle.jpg]

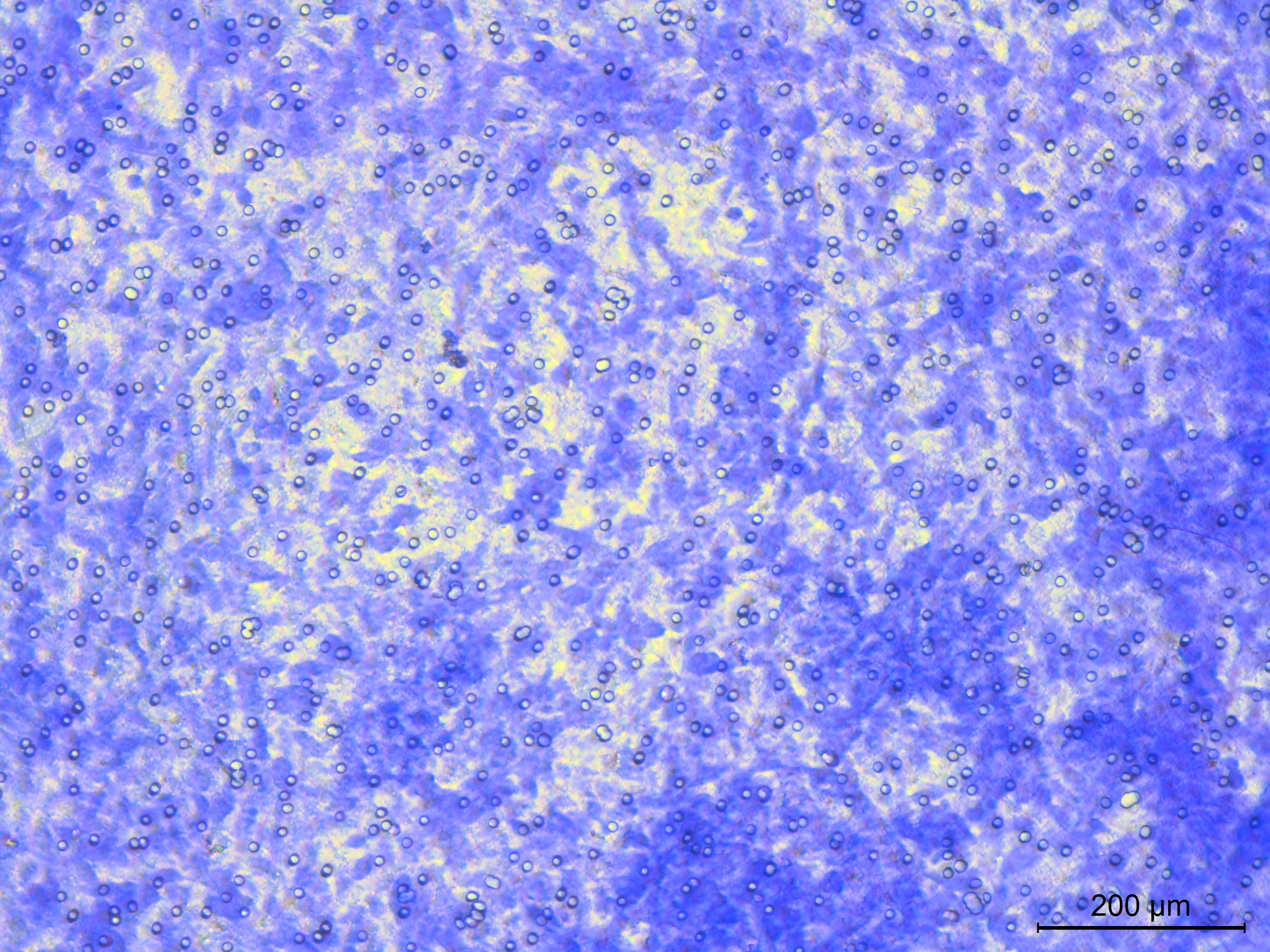

Supplement: Supplementary file 14 [file DataSheet_7.zip › raw data-figure 3c-MDAMB231/raw data-fig.3c.MDAMB231_beta-sitoterol.tif]

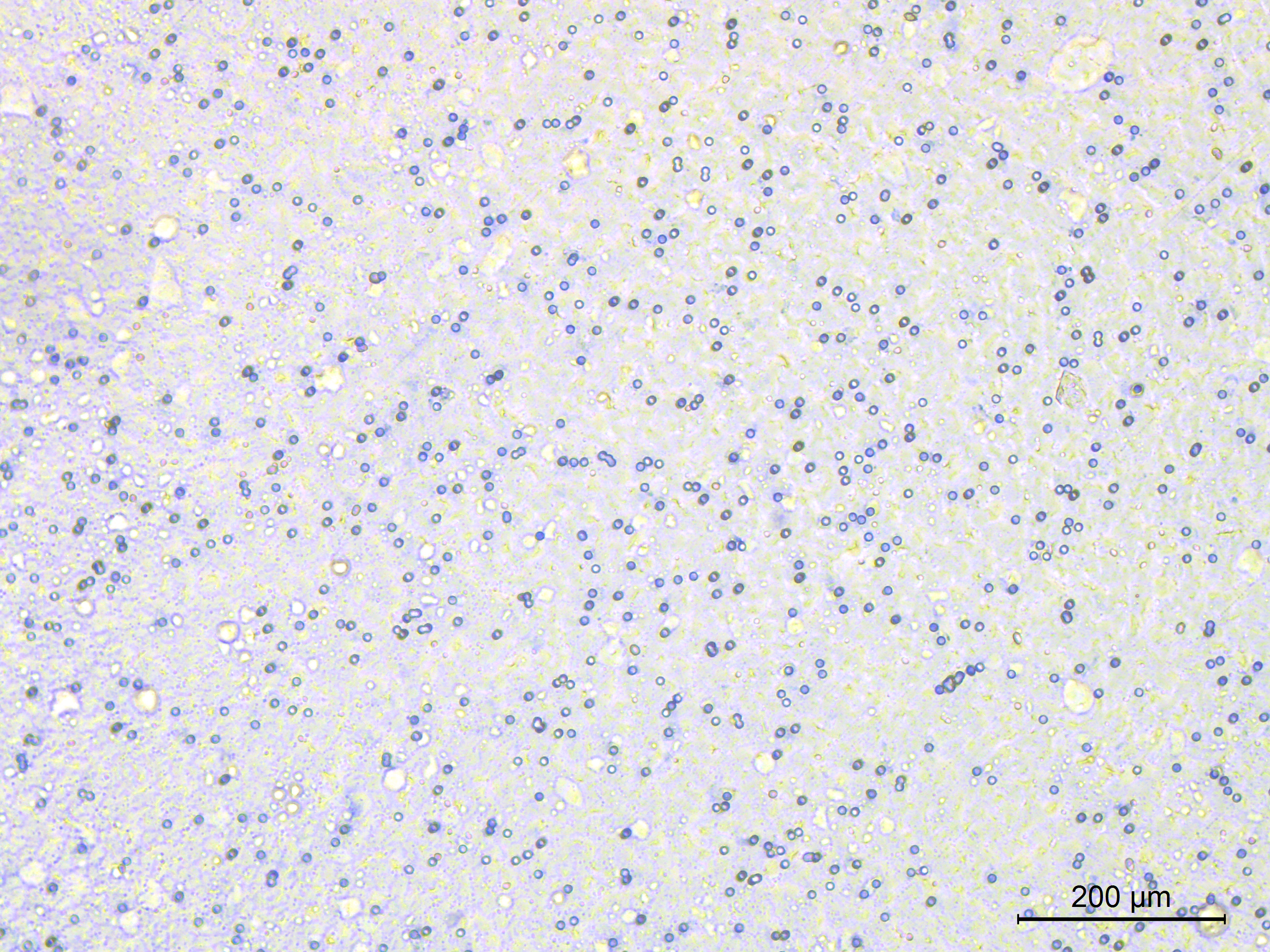

Supplement: Supplementary file 14 [file DataSheet_7.zip › raw data-figure 3c-MDAMB231/raw data-fig.3c.MDAMB231_combination.tif]

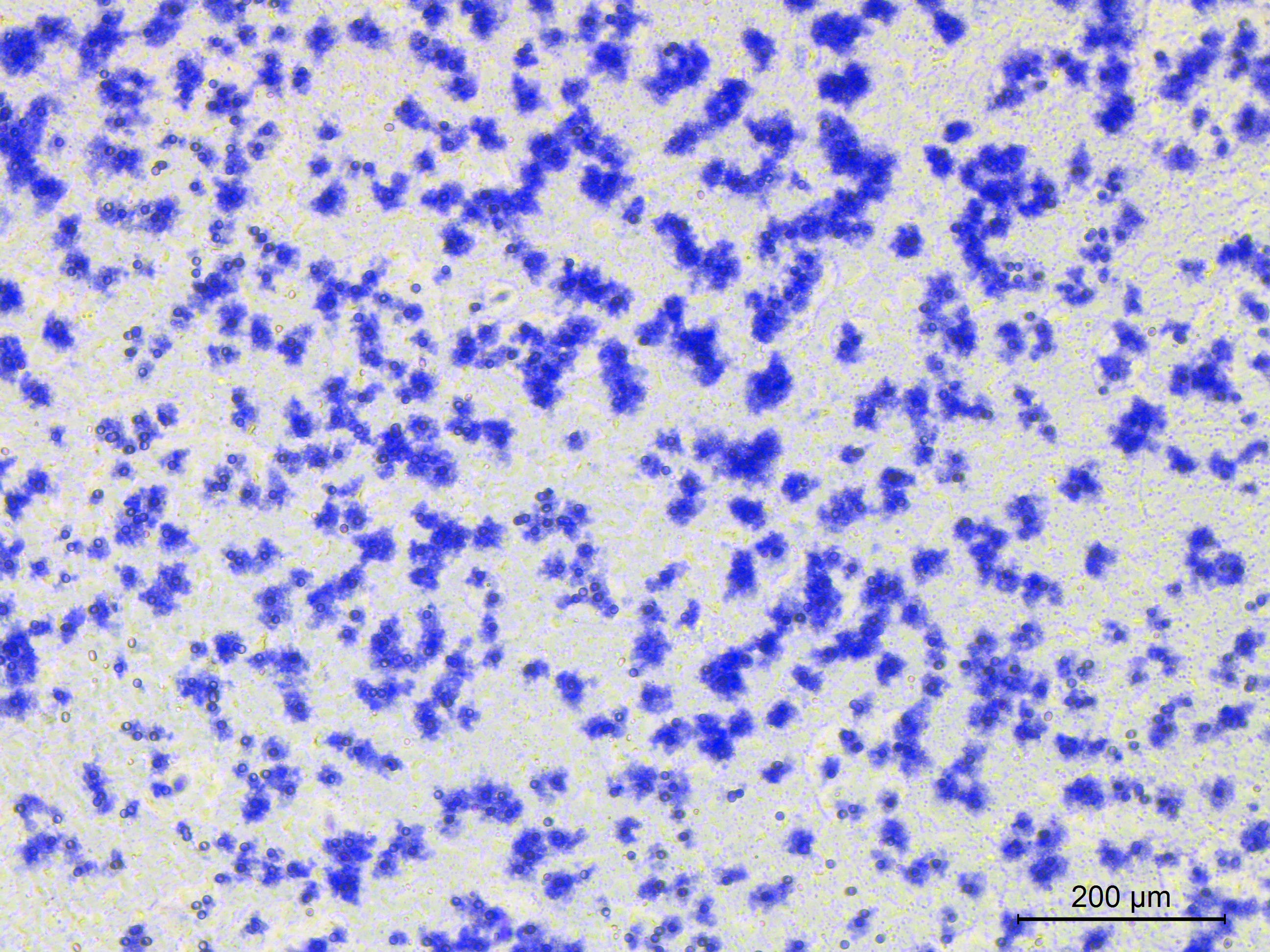

Supplement: Supplementary file 14 [file DataSheet_7.zip › raw data-figure 3c-MDAMB231/raw data-fig.3c.MDAMB231_quercetin.tif]

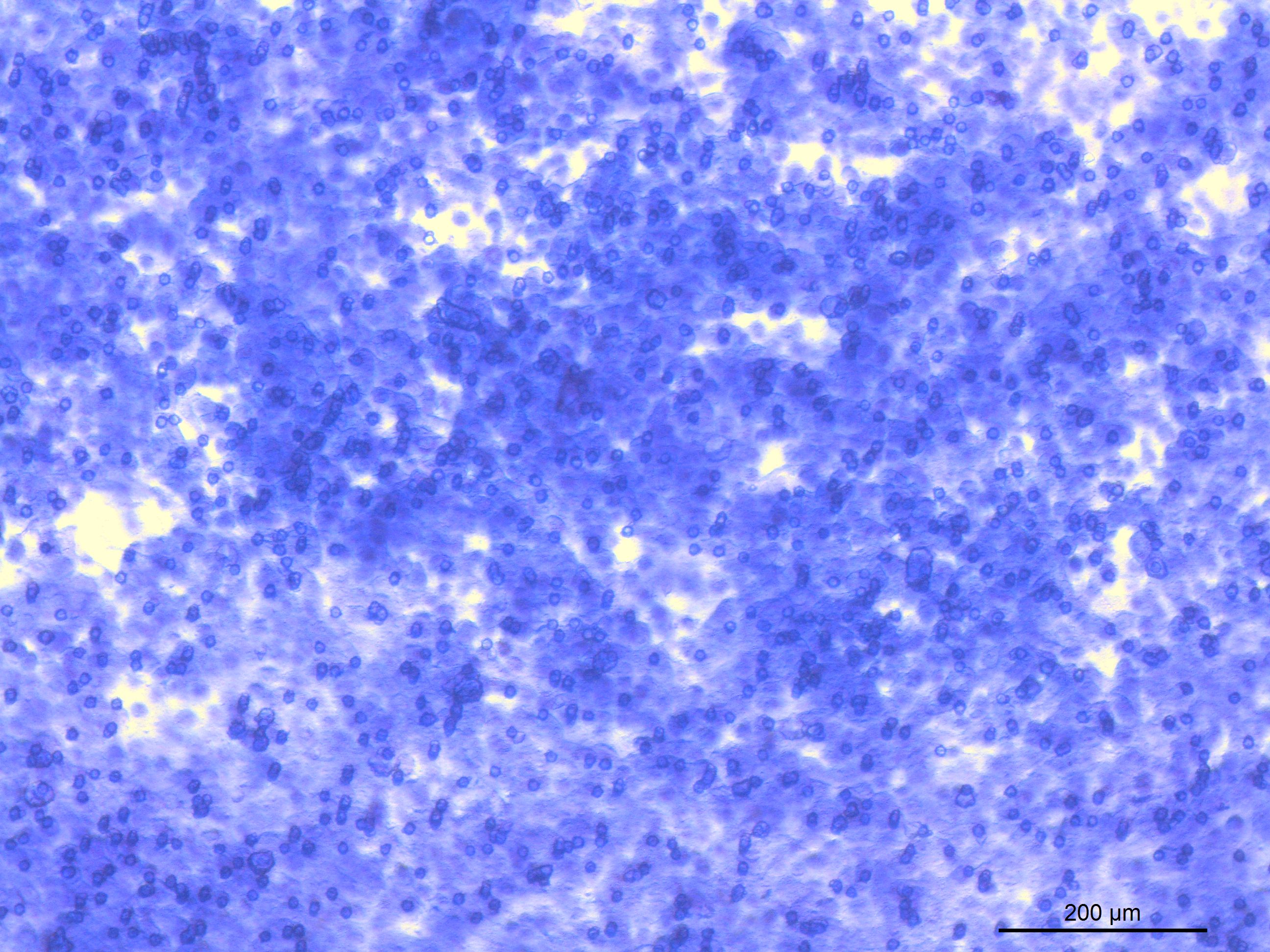

Supplement: Supplementary file 14 [file DataSheet_7.zip › raw data-figure 3c-MDAMB231/raw data-fig.3c.MDAMB231_vehicle.jpg]

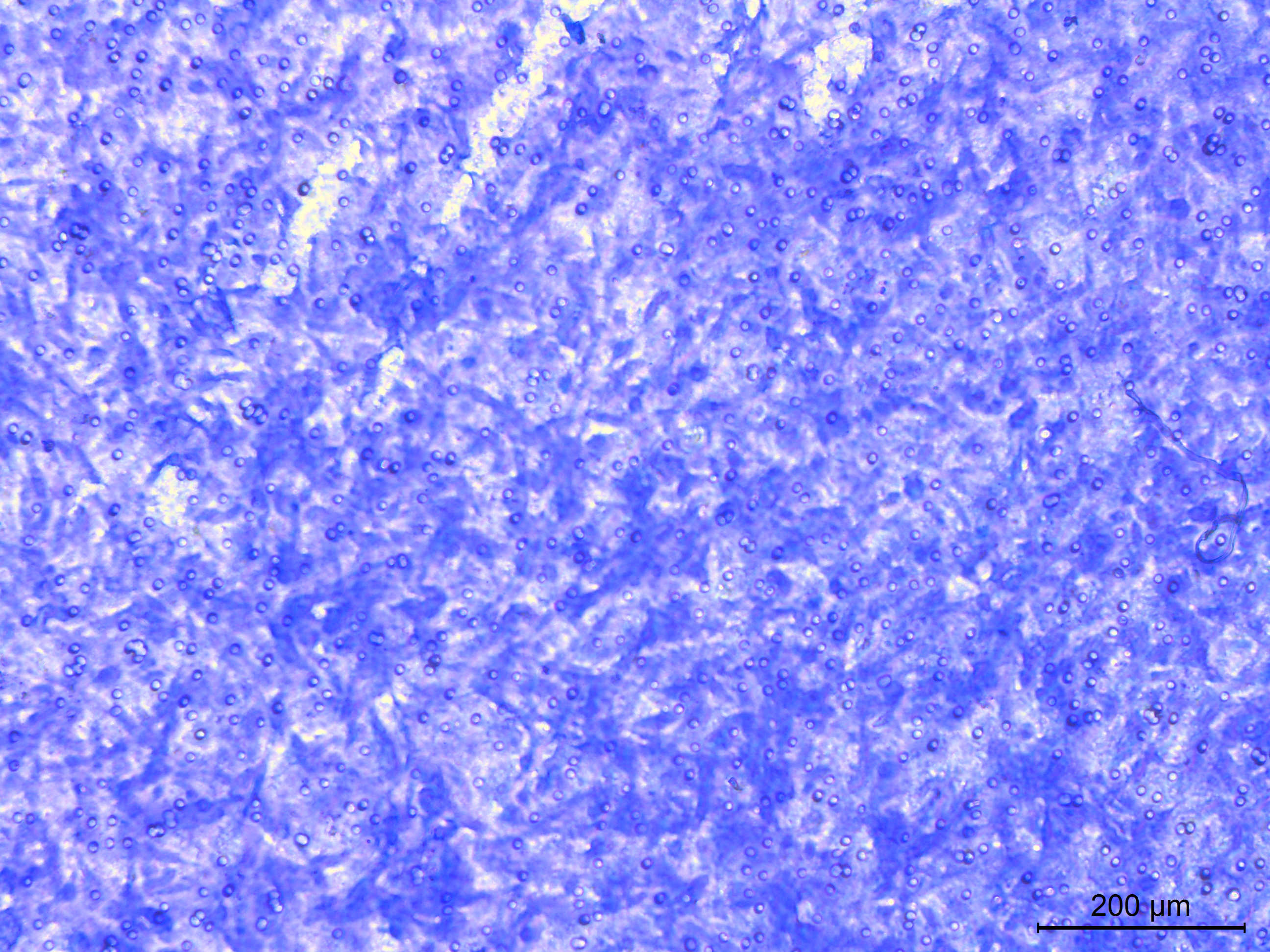

Supplement: Supplementary file 15 [file DataSheet_8.zip › raw data-migration assay-HCC1187/raw data-fig.3c.HCC1187_beta-sitosterol.tif]

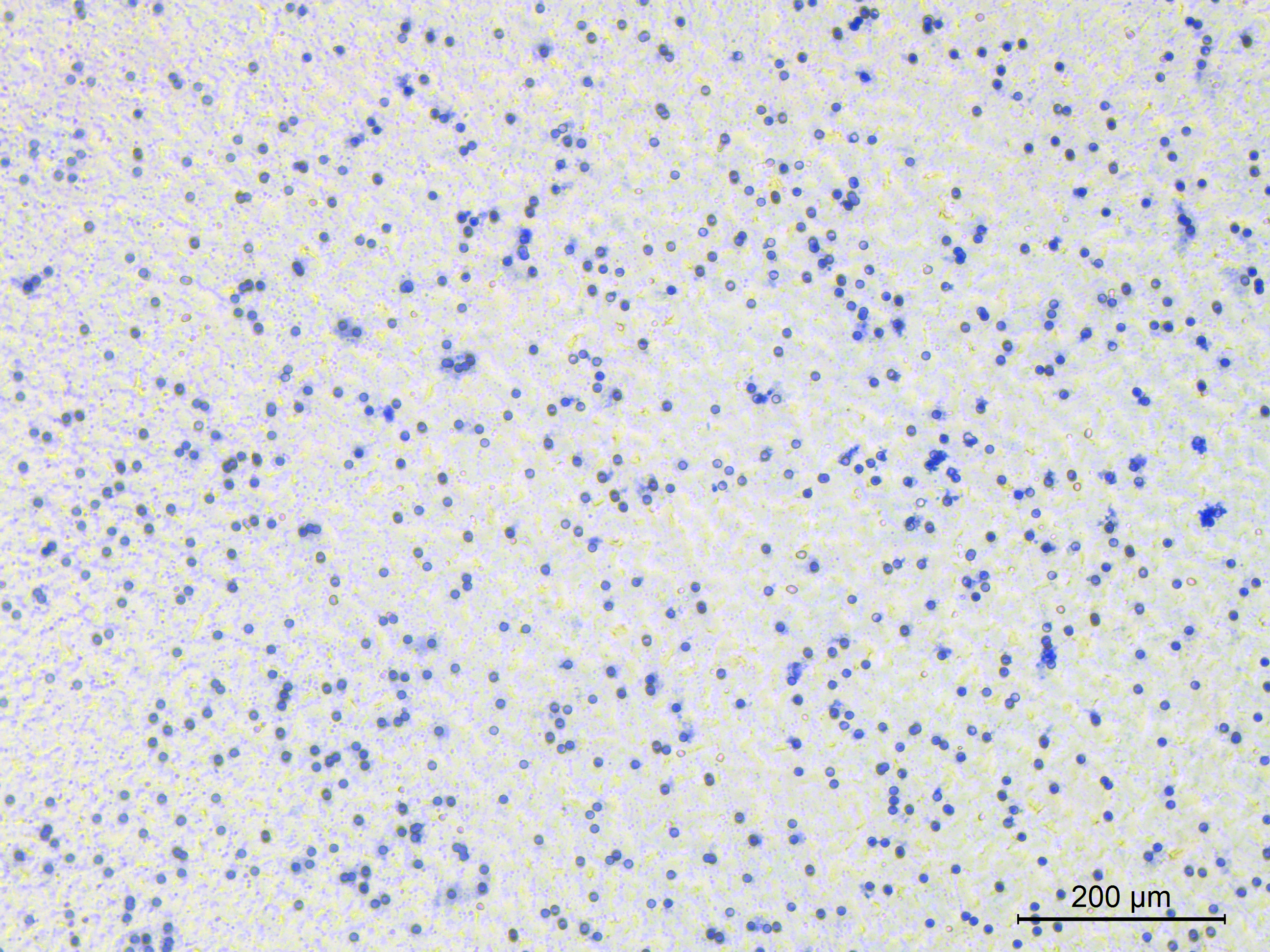

Supplement: Supplementary file 15 [file DataSheet_8.zip › raw data-migration assay-HCC1187/raw data-fig.3c.HCC1187_combination.tif]

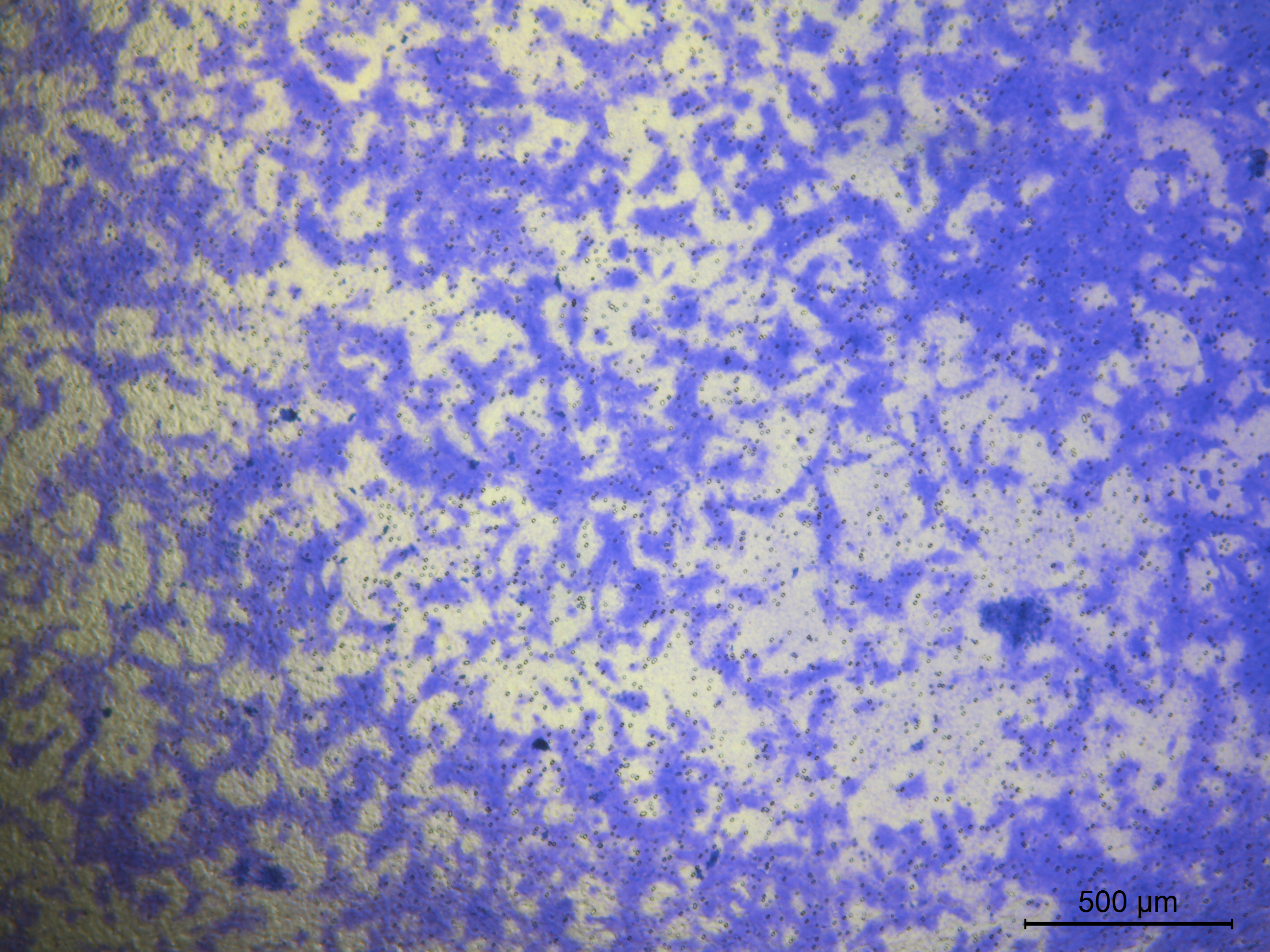

Supplement: Supplementary file 15 [file DataSheet_8.zip › raw data-migration assay-HCC1187/raw data-fig.3c.HCC1187_quercetin.tif]

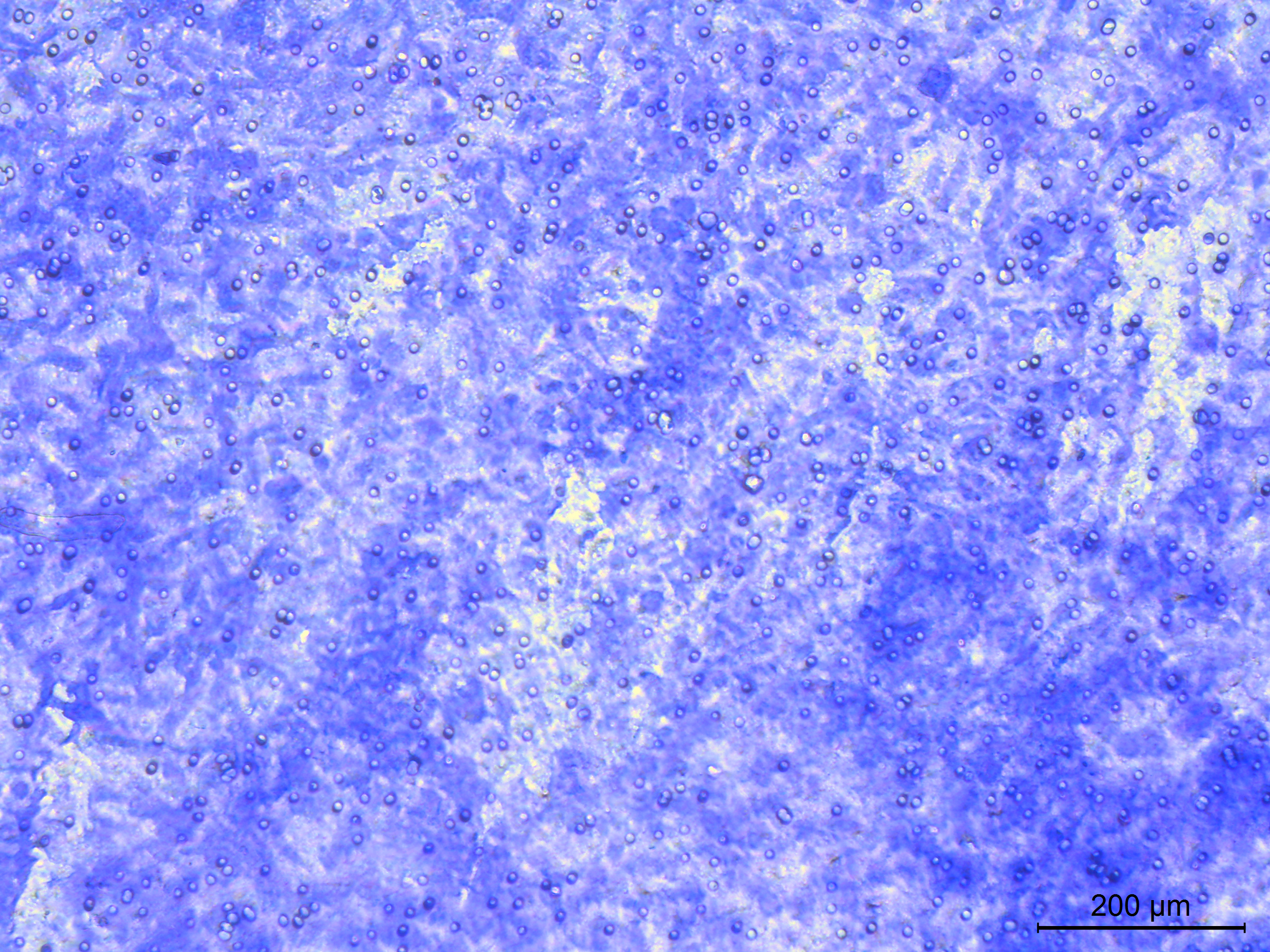

Supplement: Supplementary file 15 [file DataSheet_8.zip › raw data-migration assay-HCC1187/raw data-fig.3c.HCC1187_vehicle.tif]

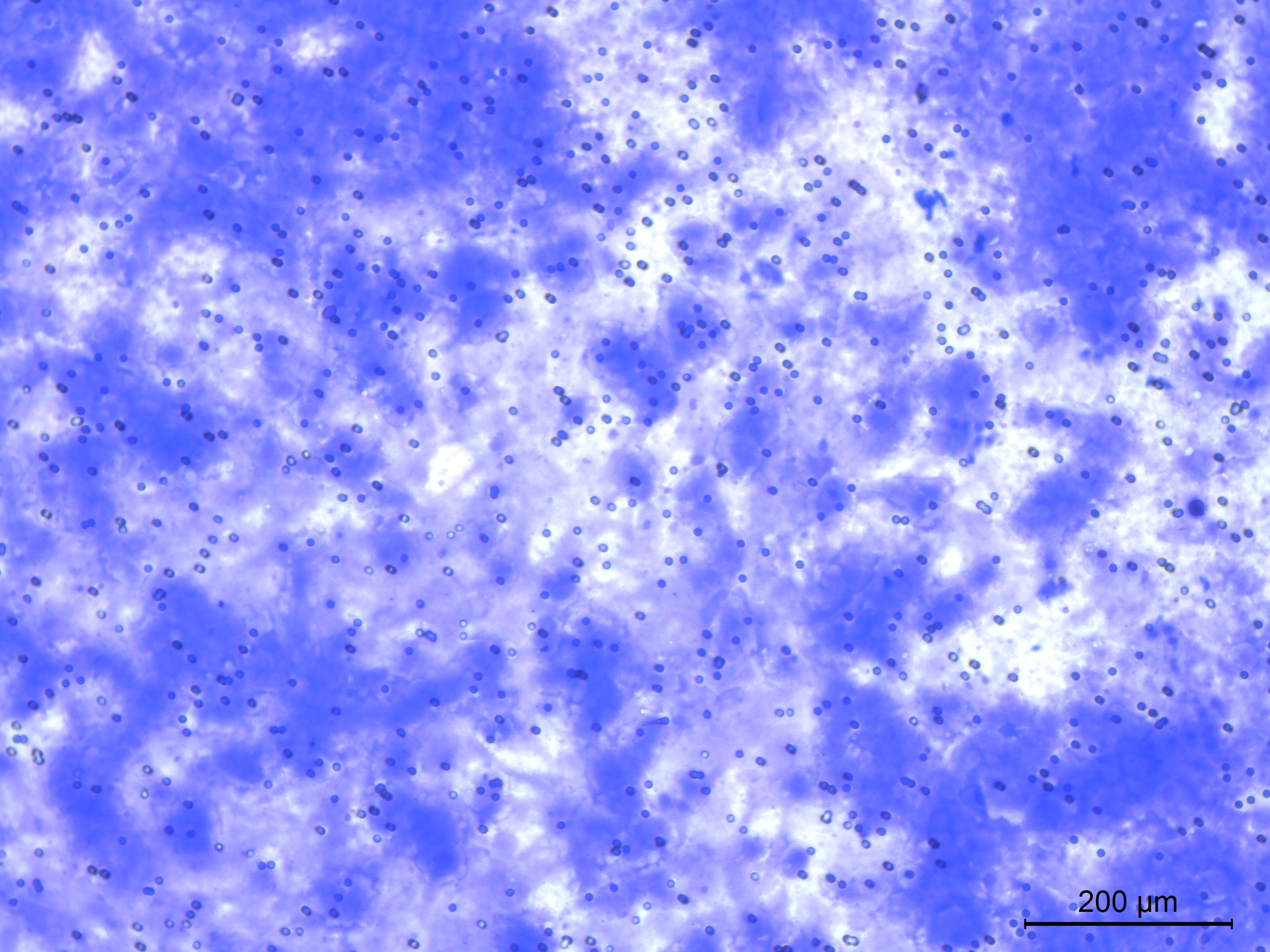

Supplement: Supplementary file 16 [file DataSheet_9.zip › Data Sheet 9/raw data-migration assay-4T1/fig.3c.4T1_beta-sitosterol.tif]

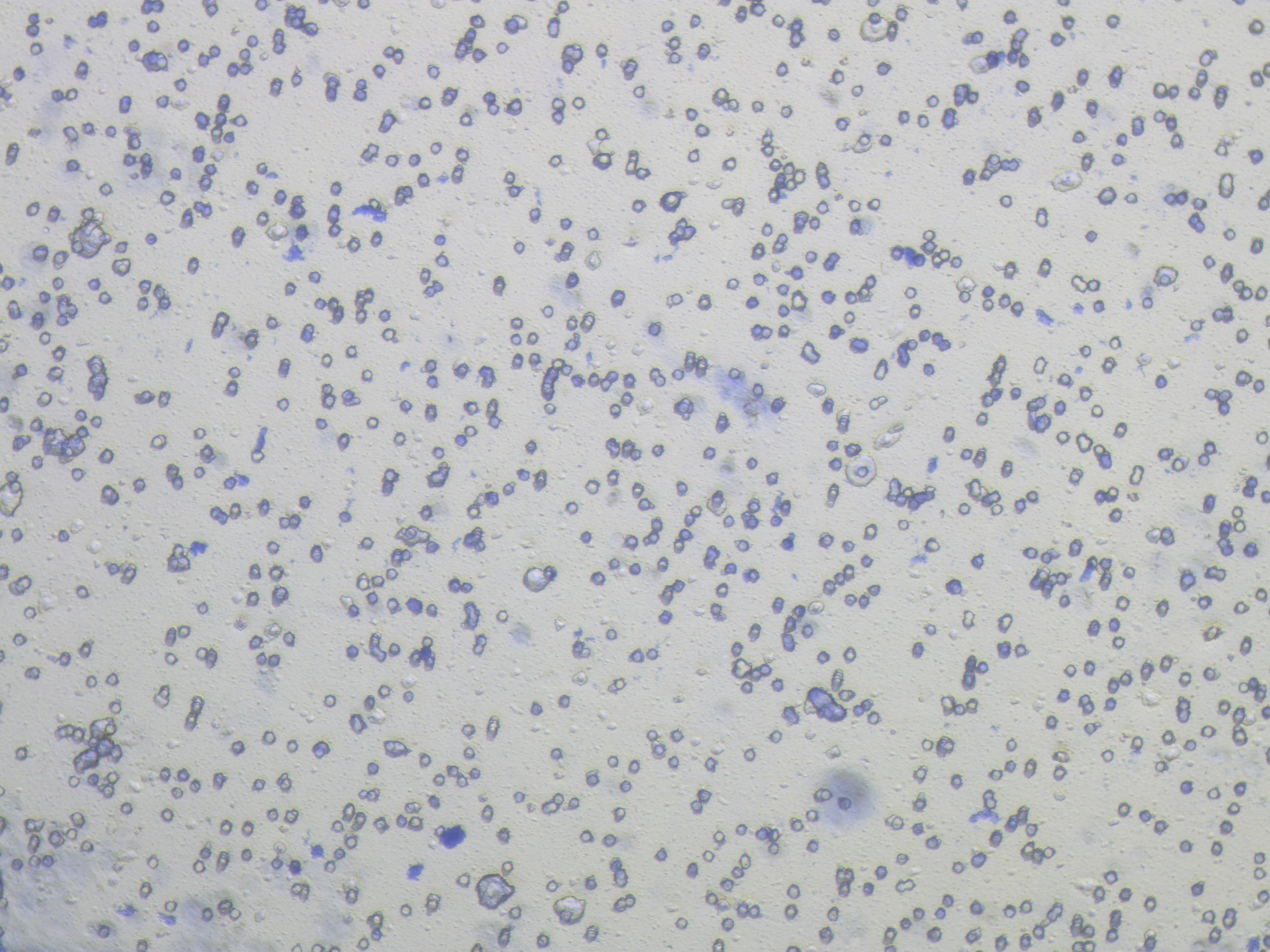

Supplement: Supplementary file 16 [file DataSheet_9.zip › Data Sheet 9/raw data-migration assay-4T1/fig.3c.4T1_combination.tif]

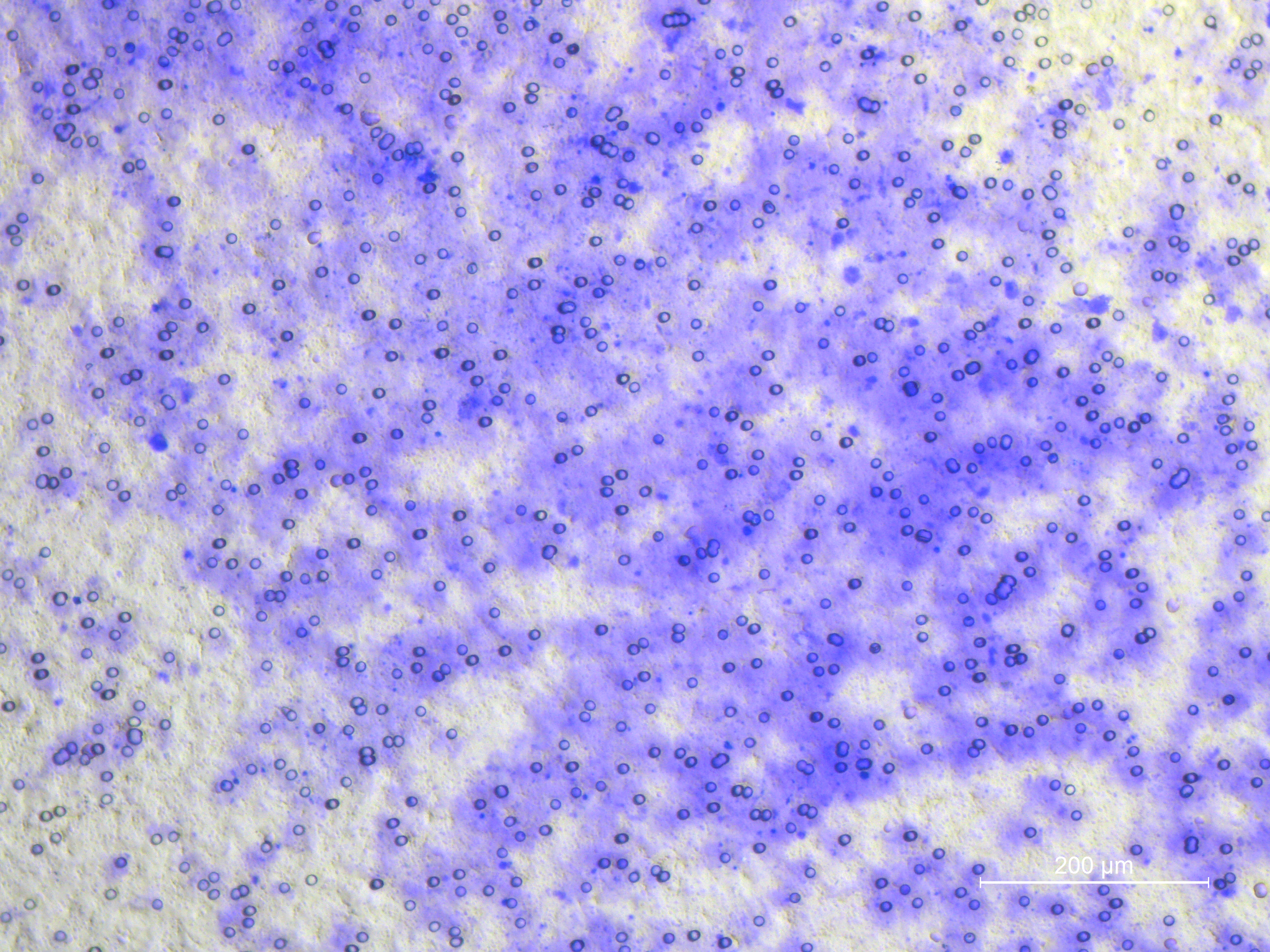

Supplement: Supplementary file 16 [file DataSheet_9.zip › Data Sheet 9/raw data-migration assay-4T1/fig.3c.4T1_quercetin.tif]

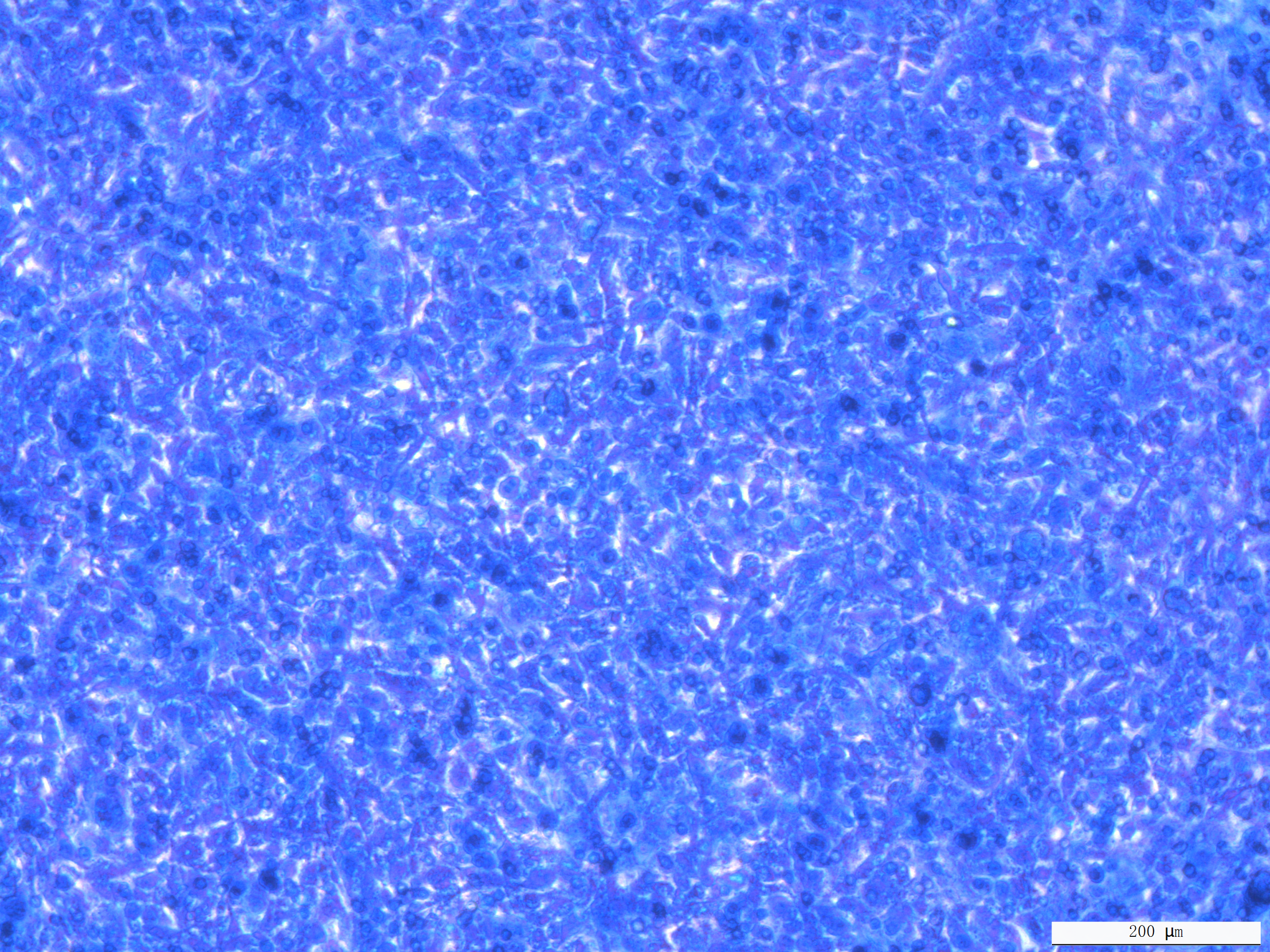

Supplement: Supplementary file 16 [file DataSheet_9.zip › Data Sheet 9/raw data-migration assay-4T1/fig.3c_4T1_vehicle.tif]

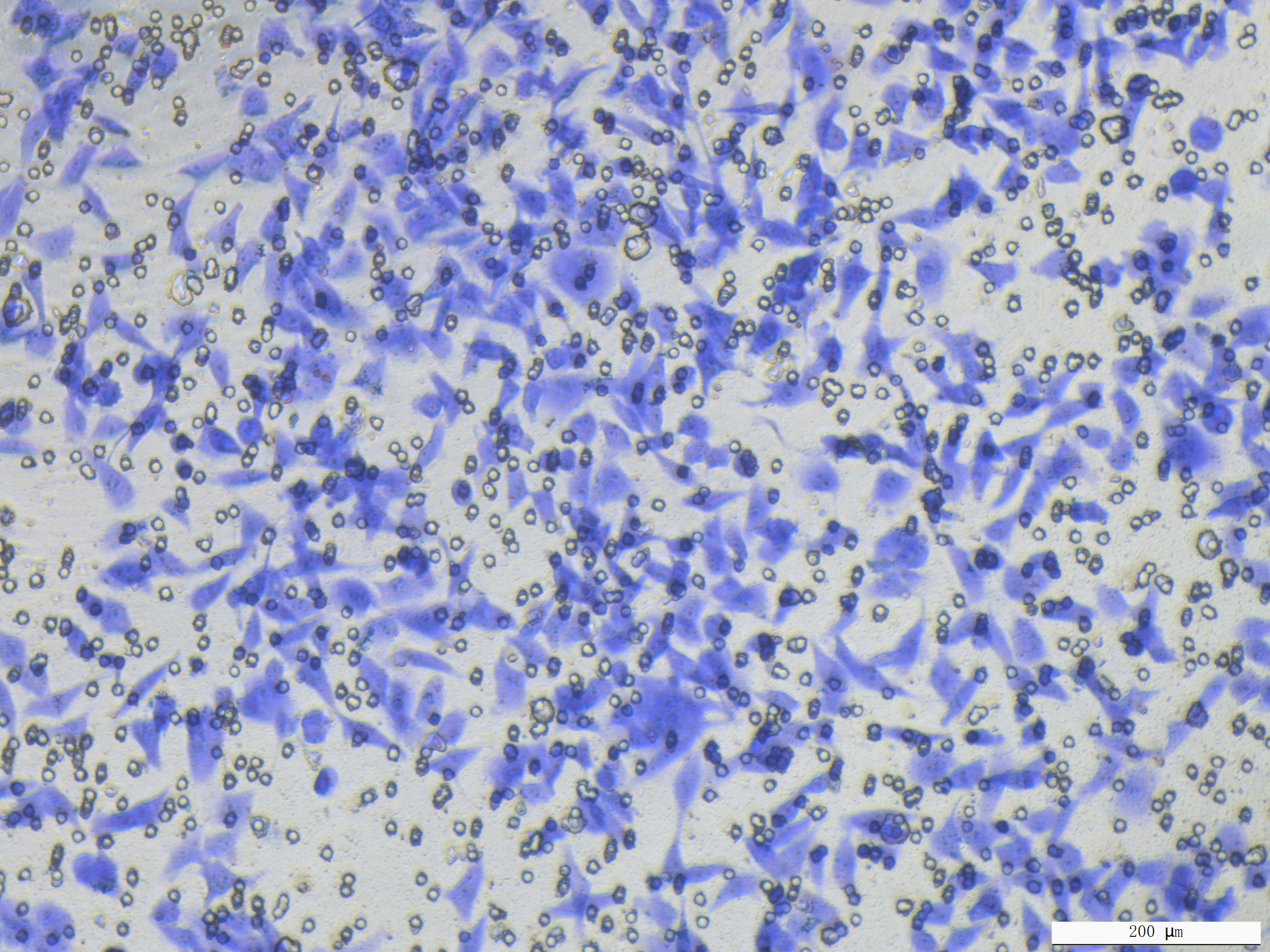

Supplement: Supplementary file 17 [file DataSheet_10.zip › Data Sheet 10/raw data-figure 3d/fig. 3d.HCC1187_vehicle.tif]

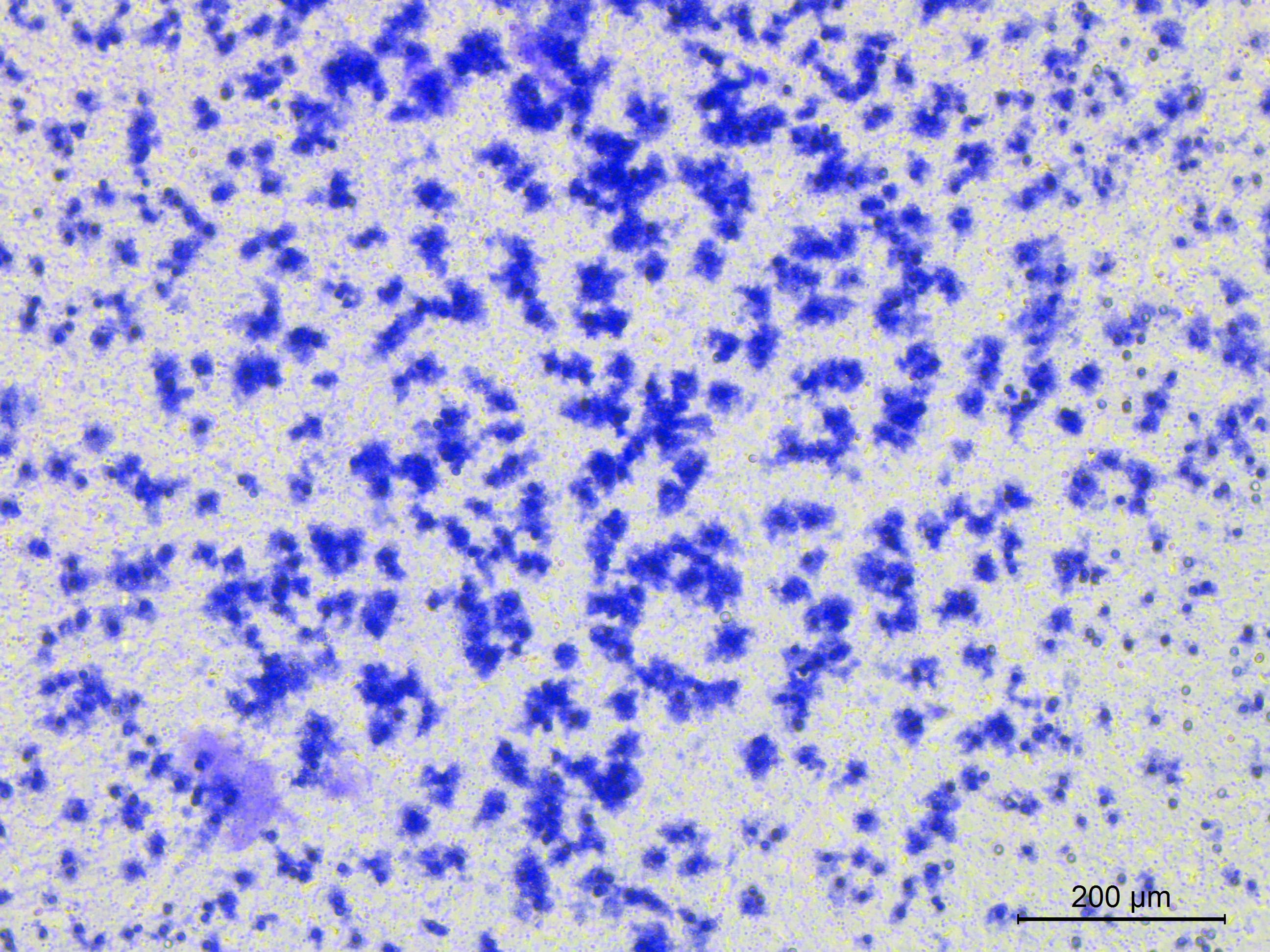

Supplement: Supplementary file 17 [file DataSheet_10.zip › Data Sheet 10/raw data-figure 3d/fig.3d.4T1_beta-sitosterol.jpg]

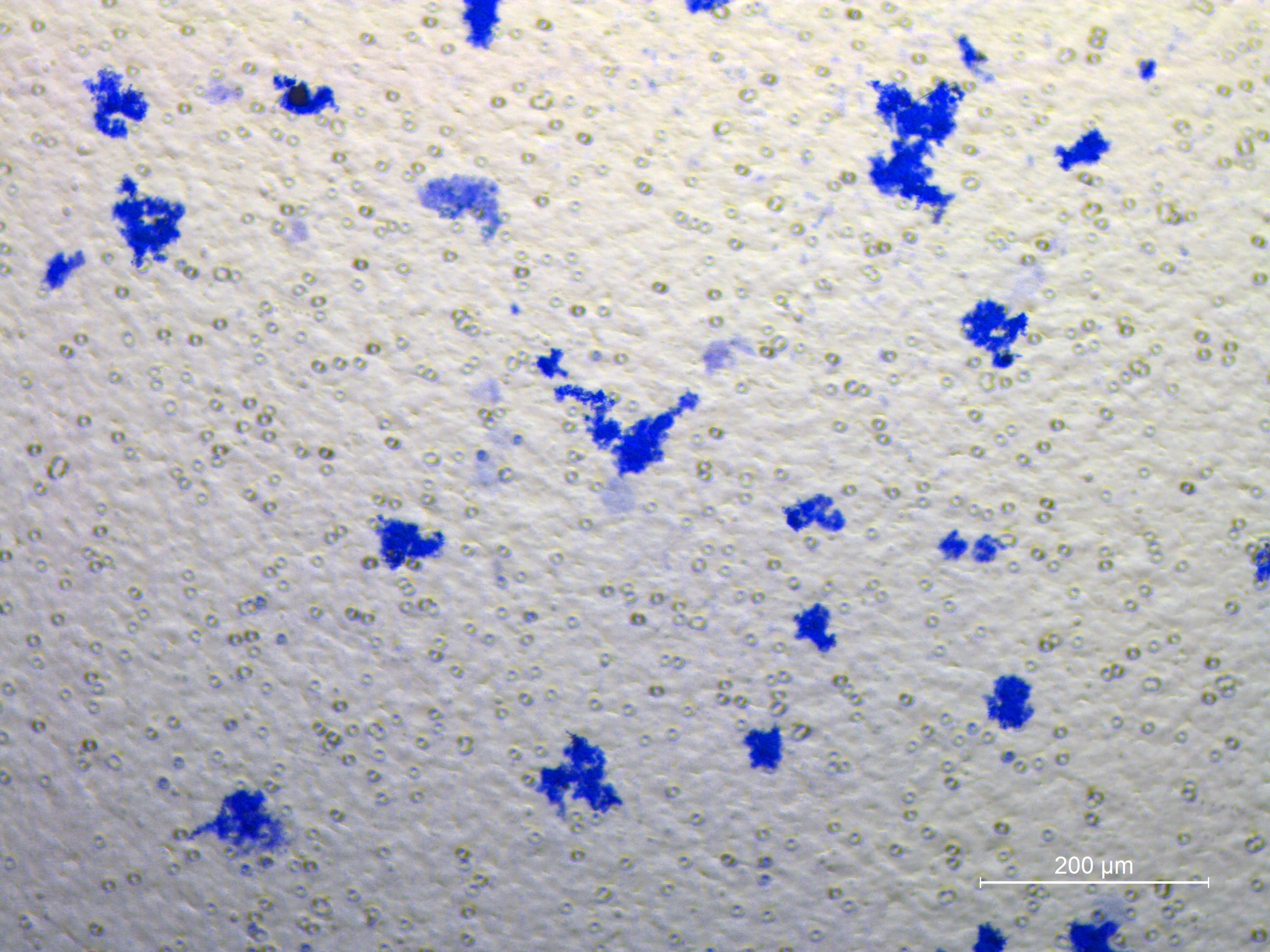

Supplement: Supplementary file 17 [file DataSheet_10.zip › Data Sheet 10/raw data-figure 3d/fig.3d.4T1_combination.jpg]

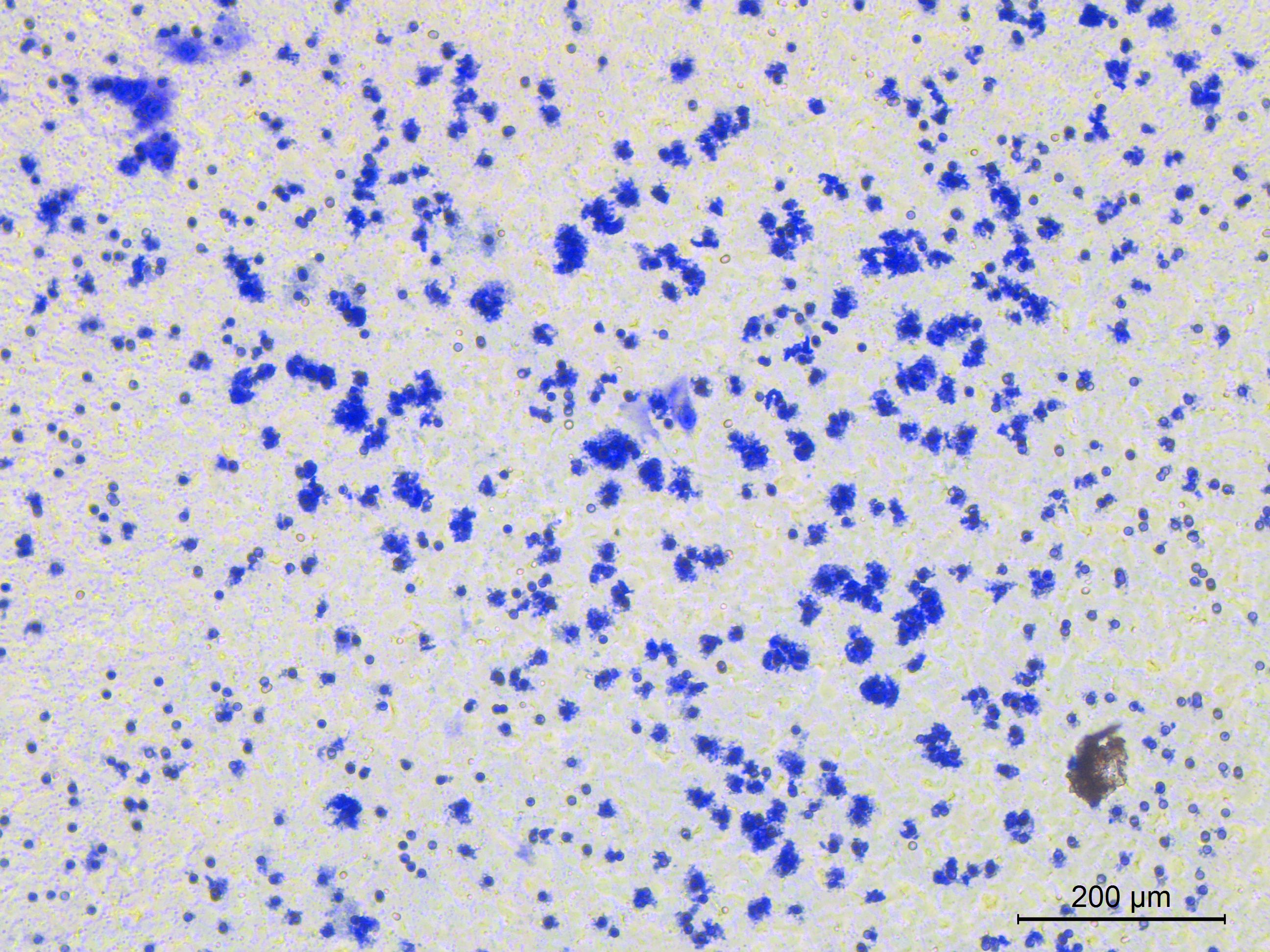

Supplement: Supplementary file 17 [file DataSheet_10.zip › Data Sheet 10/raw data-figure 3d/fig.3d.4T1_quercetin.jpg]

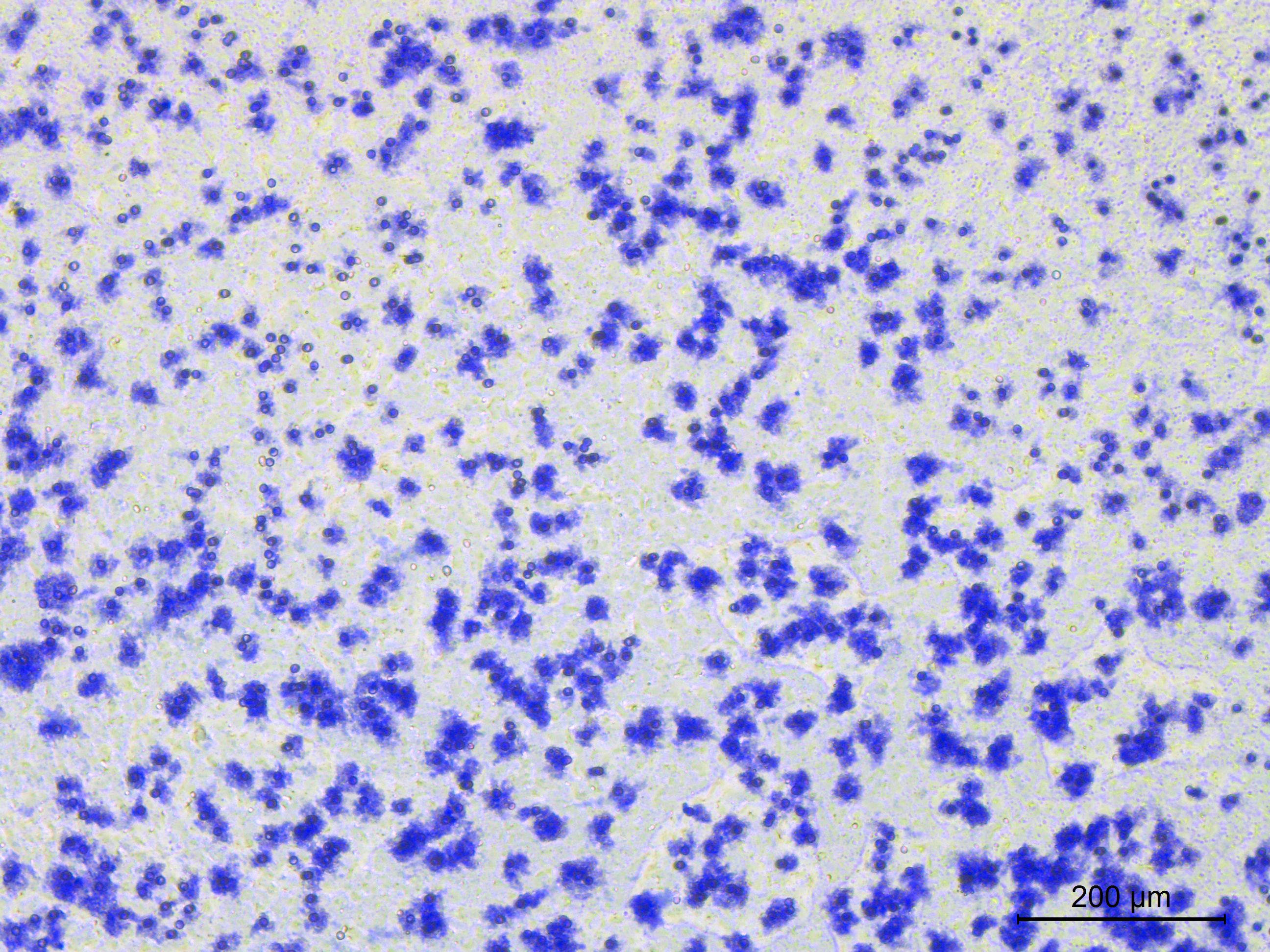

Supplement: Supplementary file 17 [file DataSheet_10.zip › Data Sheet 10/raw data-figure 3d/fig.3d.4T1_vehicle.jpg]

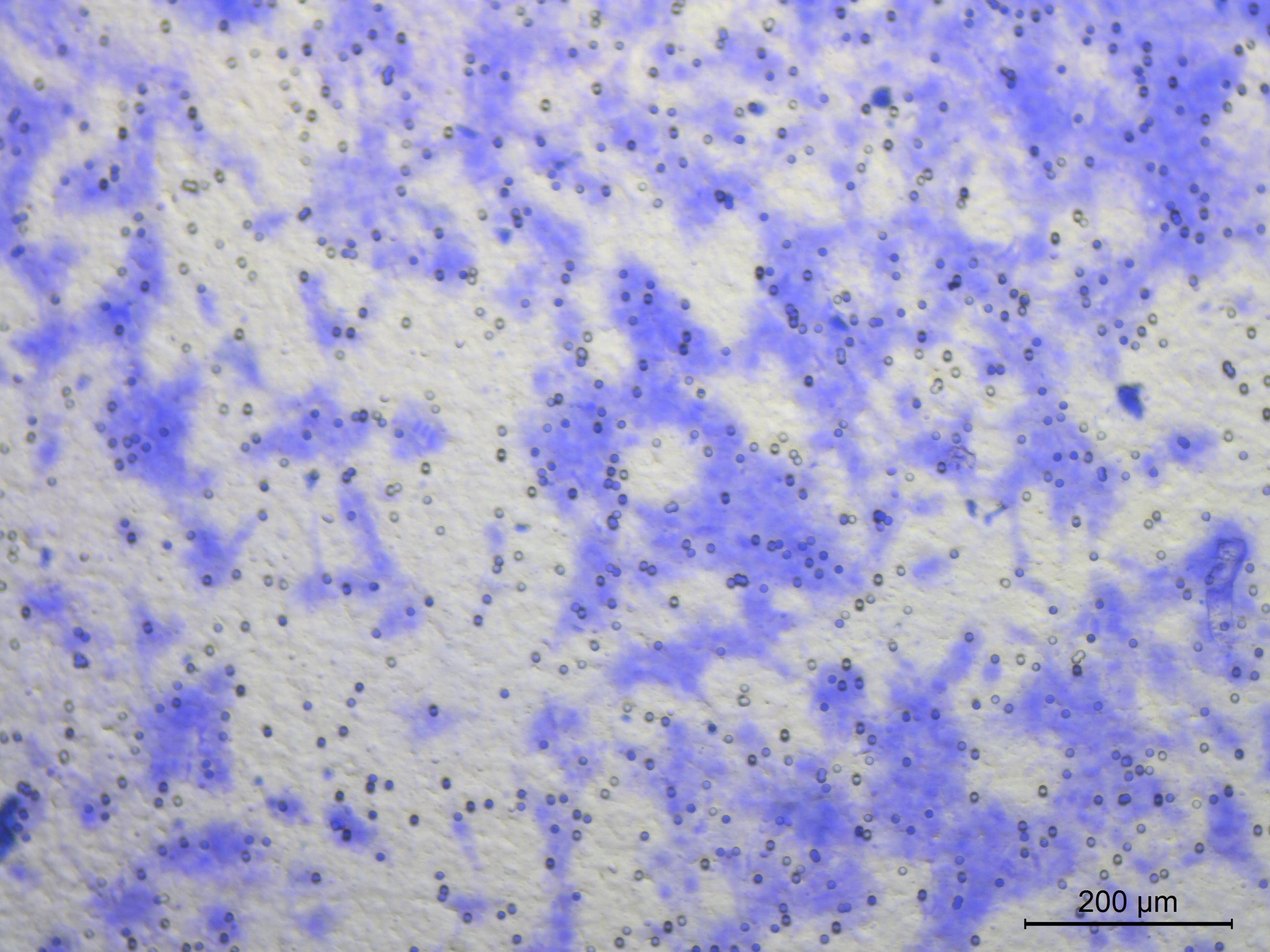

Supplement: Supplementary file 17 [file DataSheet_10.zip › Data Sheet 10/raw data-figure 3d/fig.3d.HCC1187_beta-sitosterol.jpg]

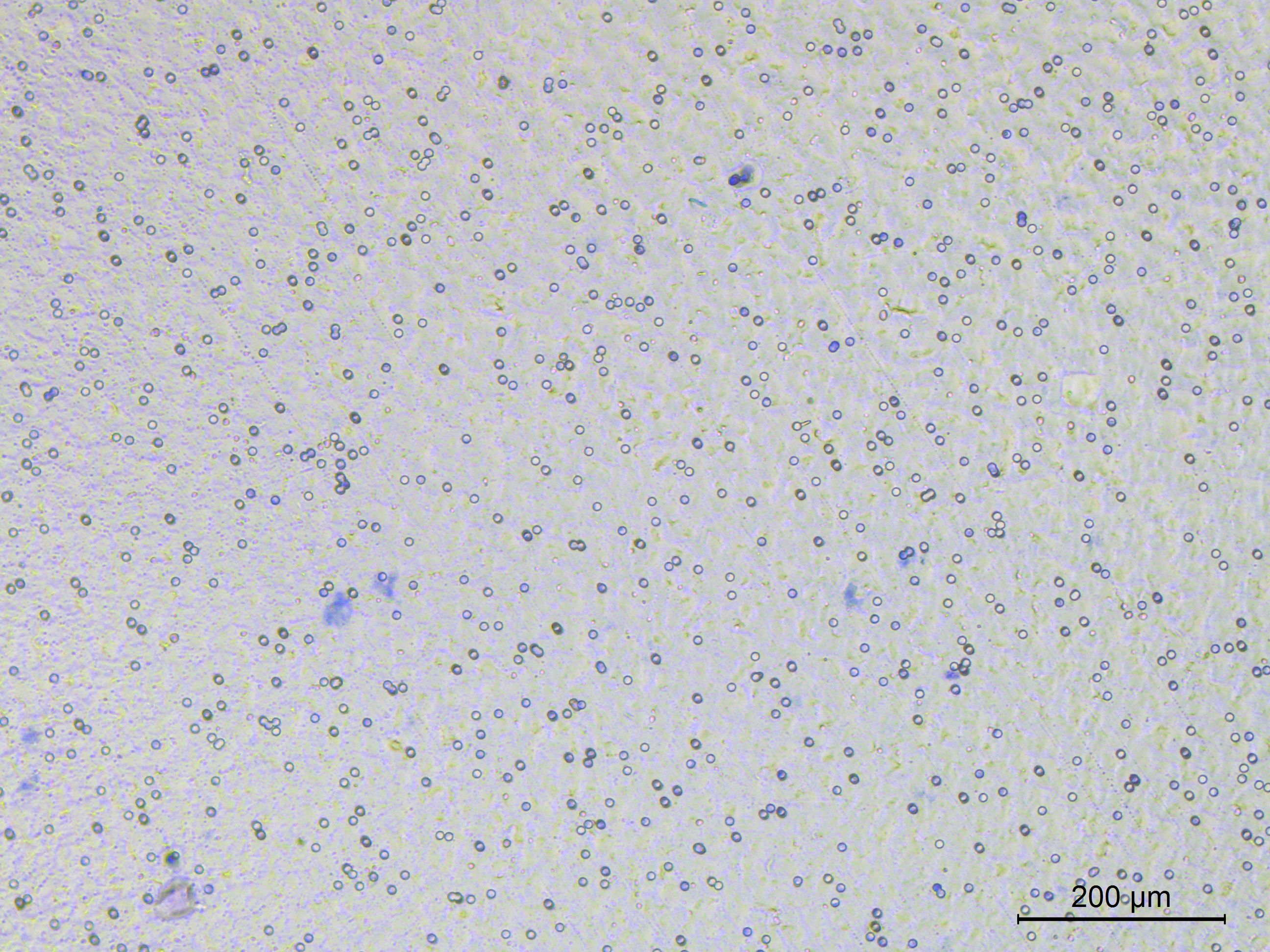

Supplement: Supplementary file 17 [file DataSheet_10.zip › Data Sheet 10/raw data-figure 3d/fig.3d.HCC1187_combination.jpg]

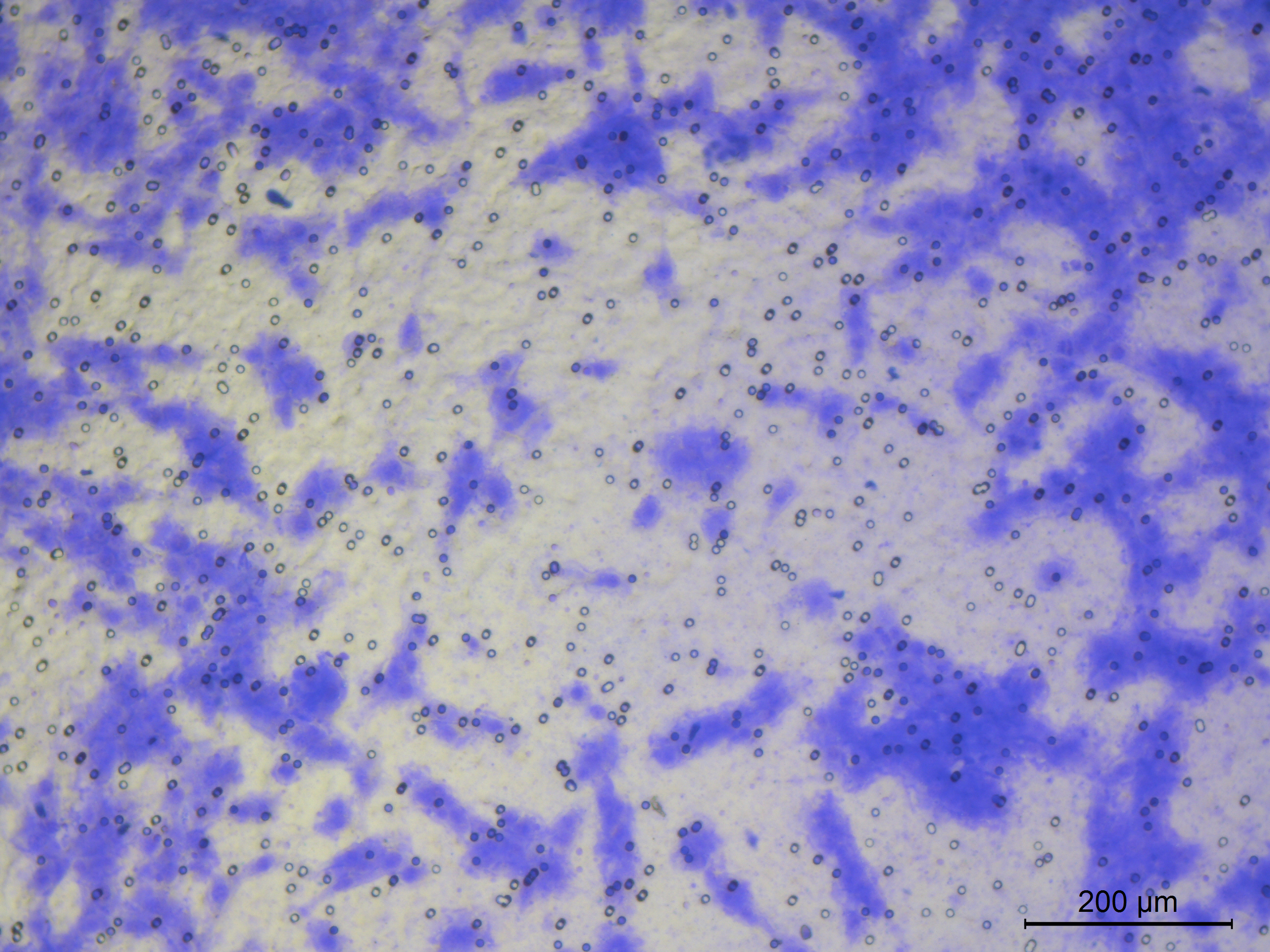

Supplement: Supplementary file 17 [file DataSheet_10.zip › Data Sheet 10/raw data-figure 3d/fig.3d.HCC1187_quercetin.jpg]

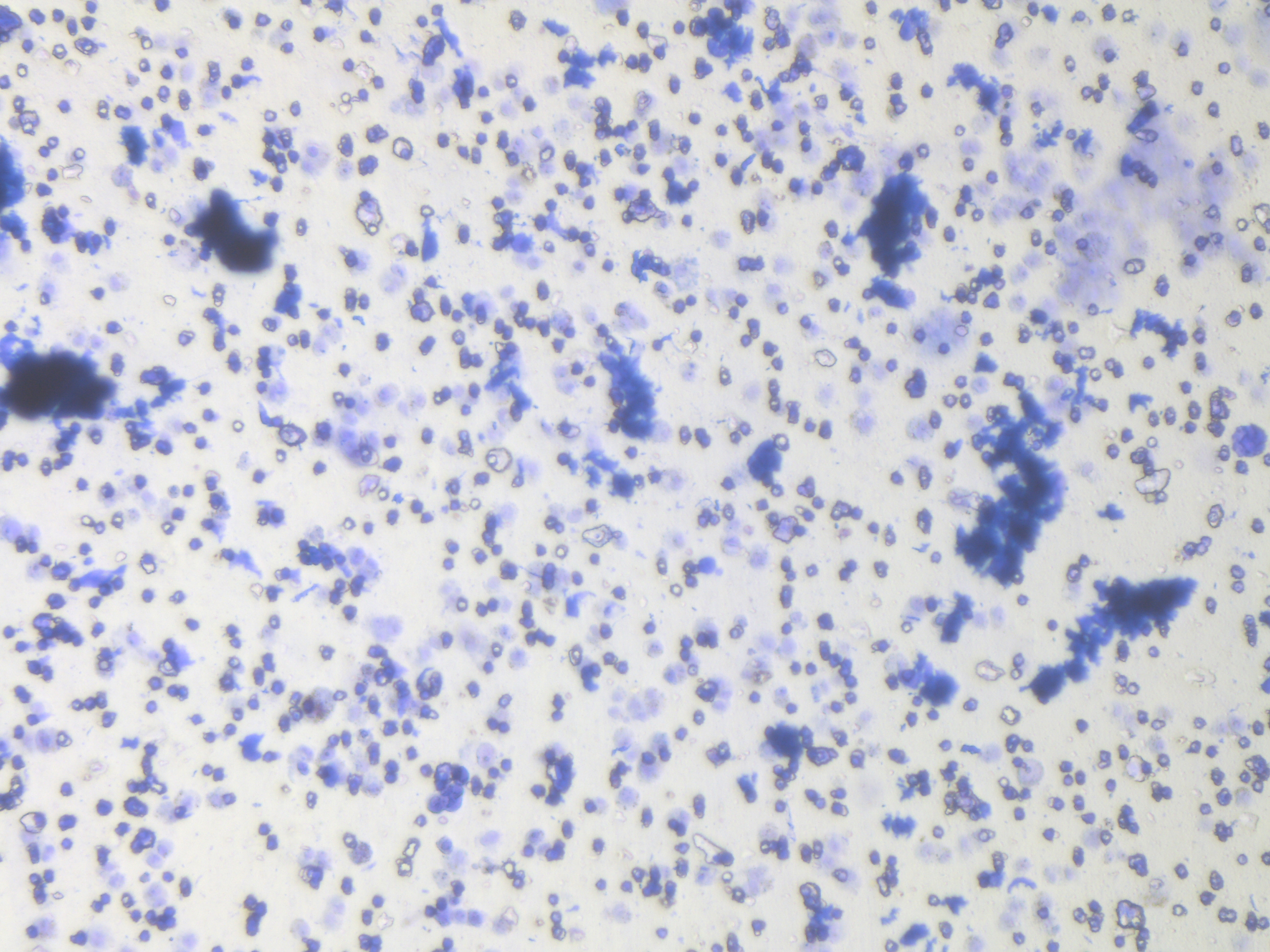

Supplement: Supplementary file 17 [file DataSheet_10.zip › Data Sheet 10/raw data-figure 3d/fig.3d.MDAMB231_beta-sitosterol.jpg]

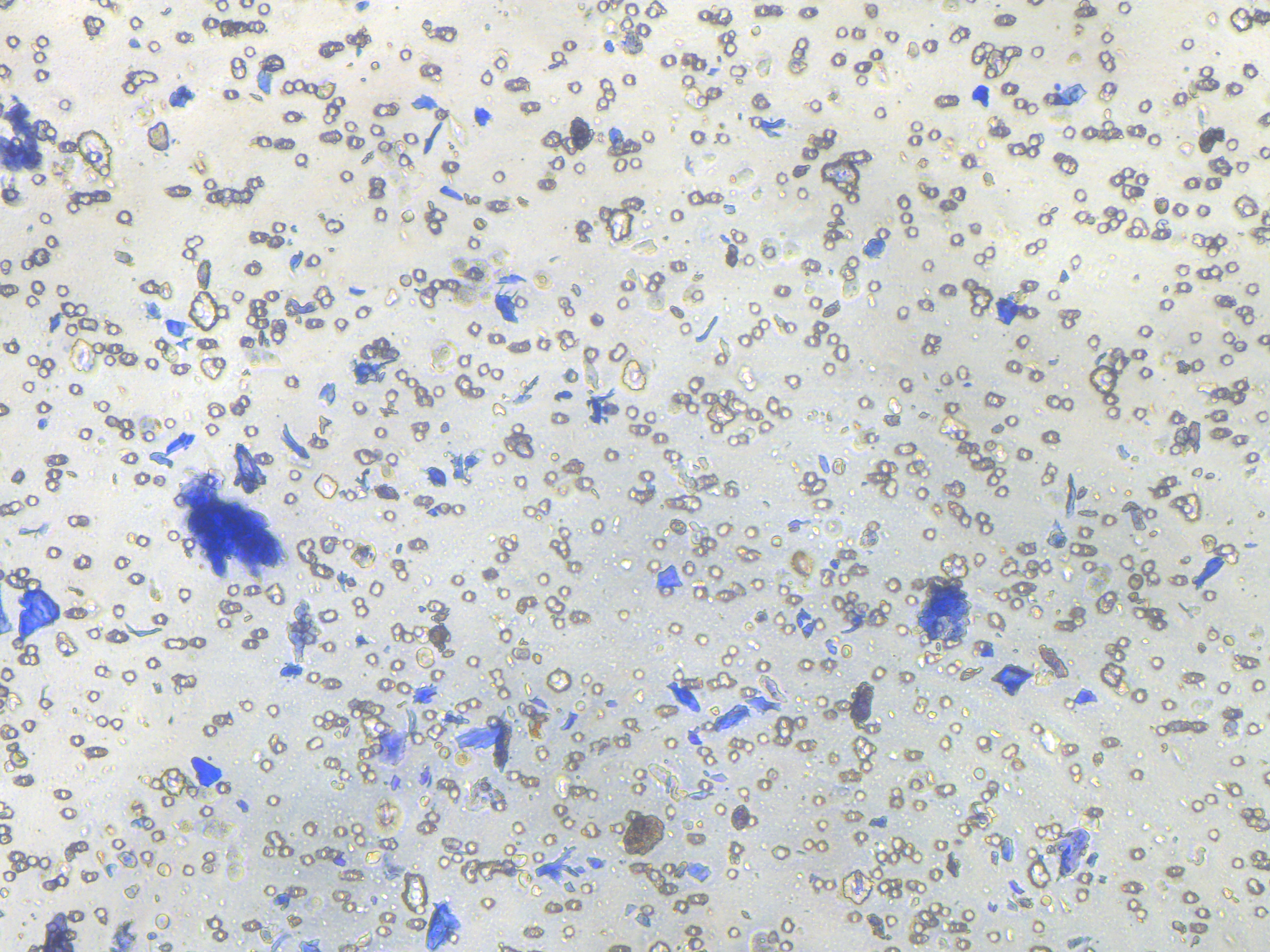

Supplement: Supplementary file 17 [file DataSheet_10.zip › Data Sheet 10/raw data-figure 3d/fig.3d.MDAMB231_combination.jpg]

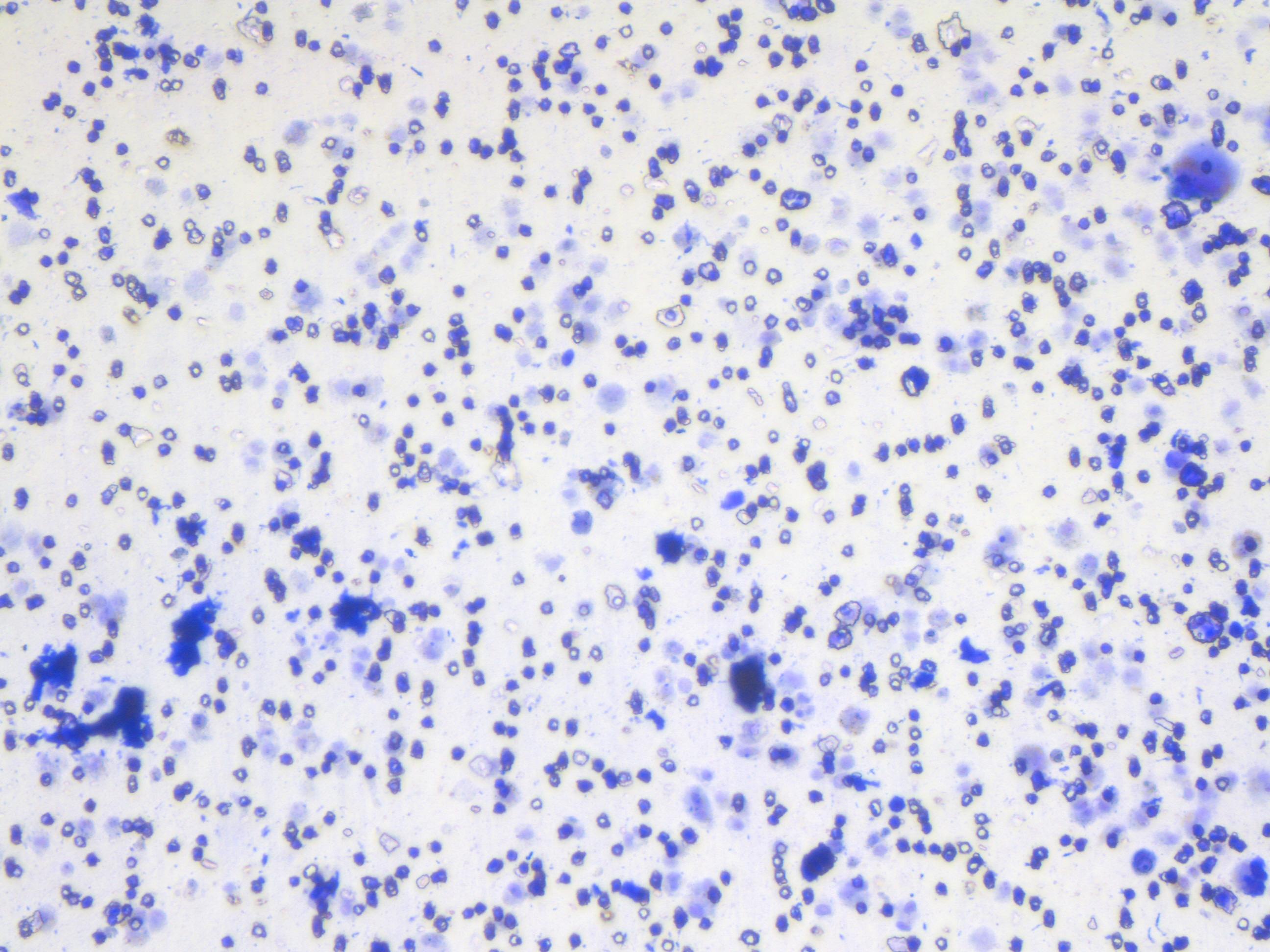

Supplement: Supplementary file 17 [file DataSheet_10.zip › Data Sheet 10/raw data-figure 3d/fig.3d.MDAMB231_quercetin.jpg]

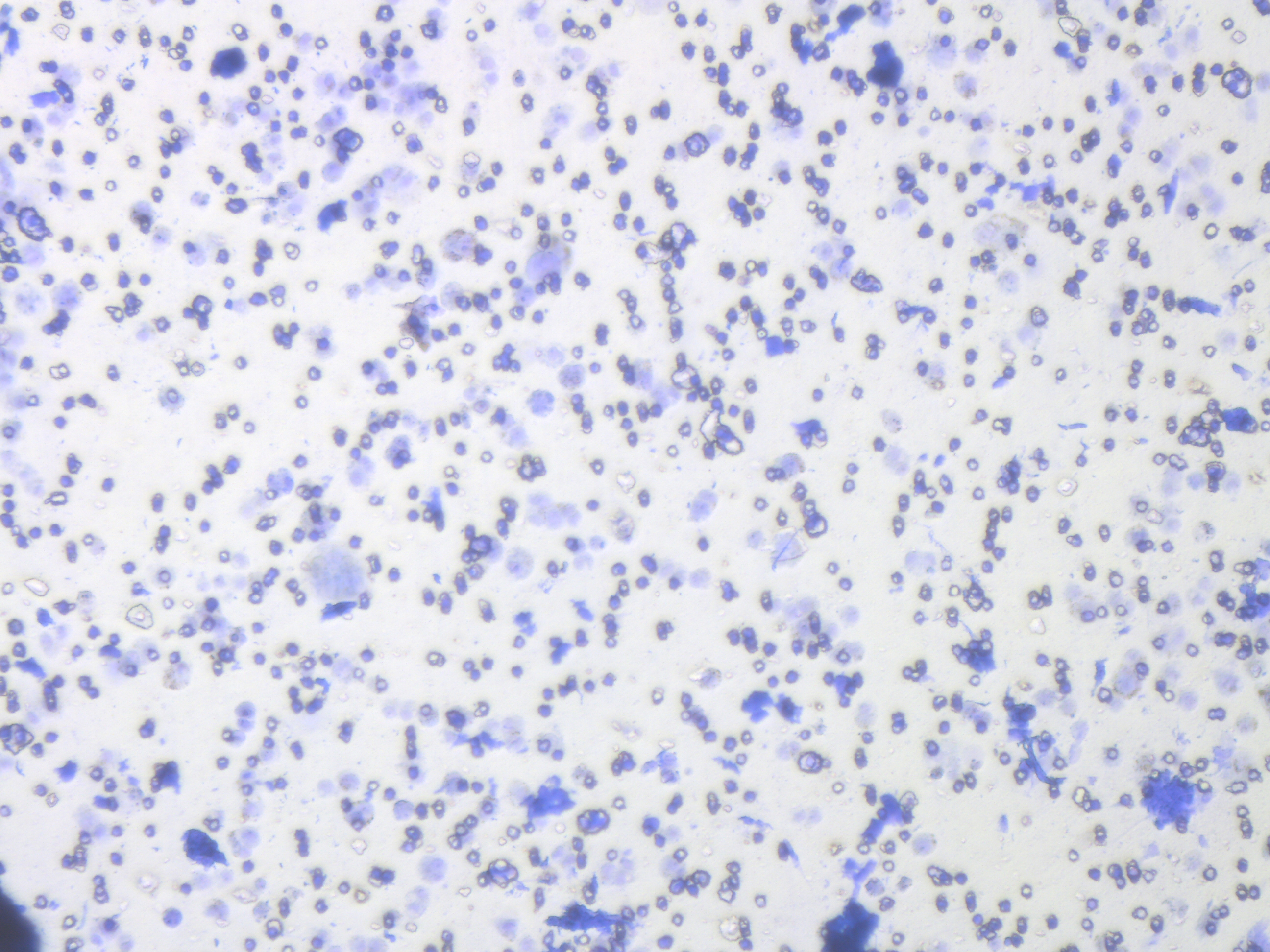

Supplement: Supplementary file 17 [file DataSheet_10.zip › Data Sheet 10/raw data-figure 3d/fig.3d.MDAMB231_vehicle.jpg]

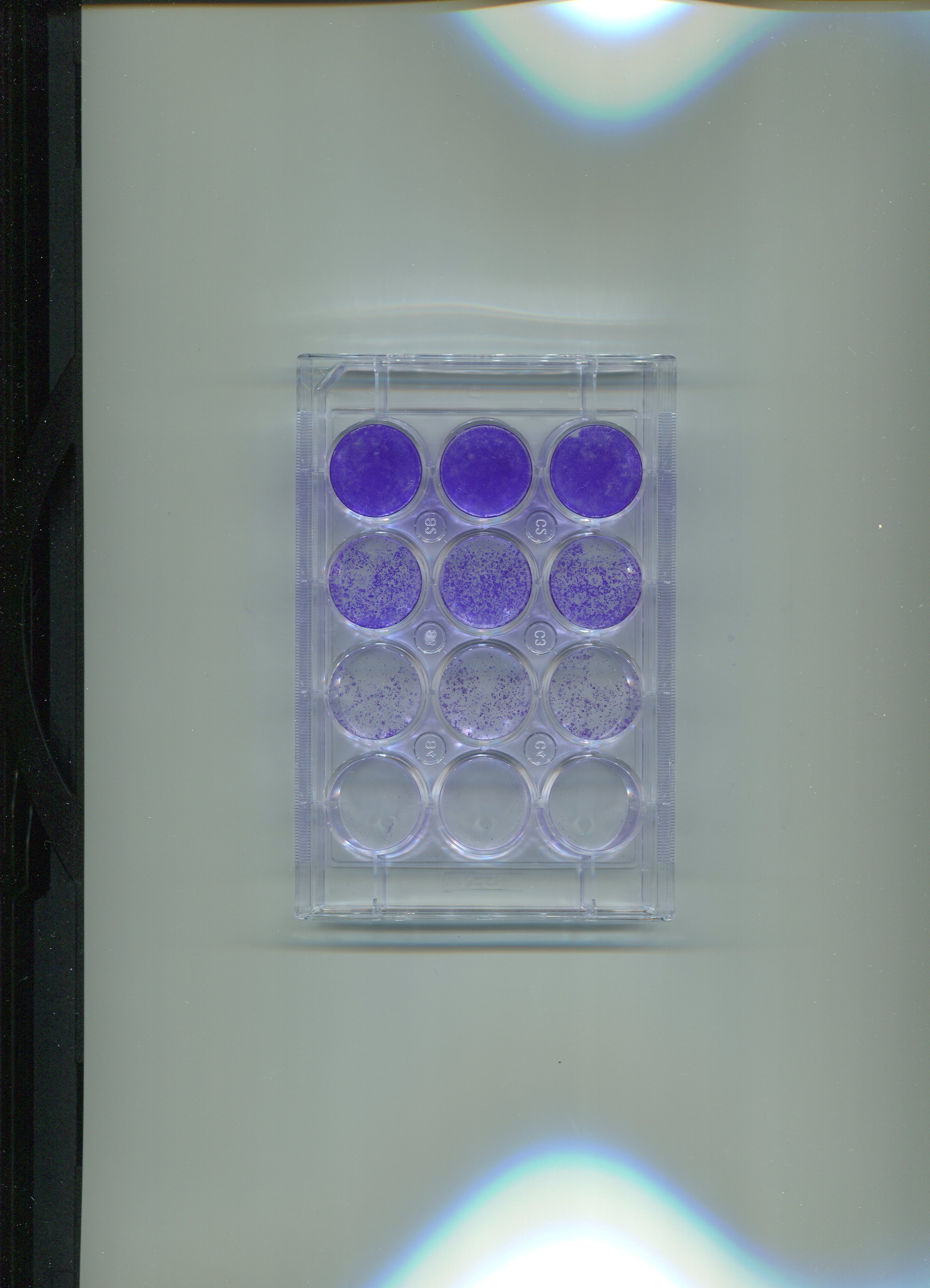

Supplement: Supplementary file 18 [file DataSheet_11.zip › other raw data/figure 1a/fig.1a.4T1-12day.tif]

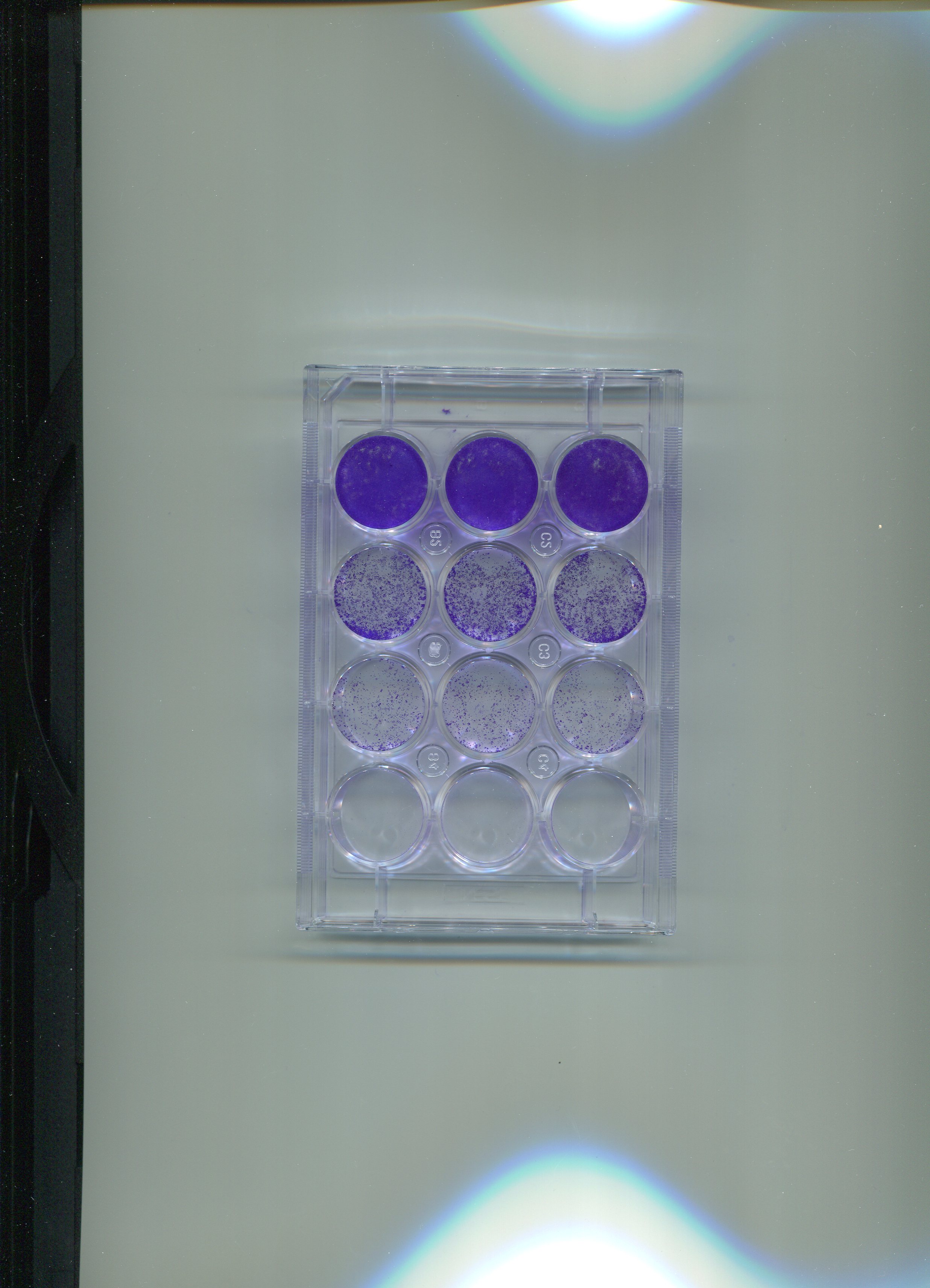

Supplement: Supplementary file 18 [file DataSheet_11.zip › other raw data/figure 1a/fig.1a.4T1-15day.tif]

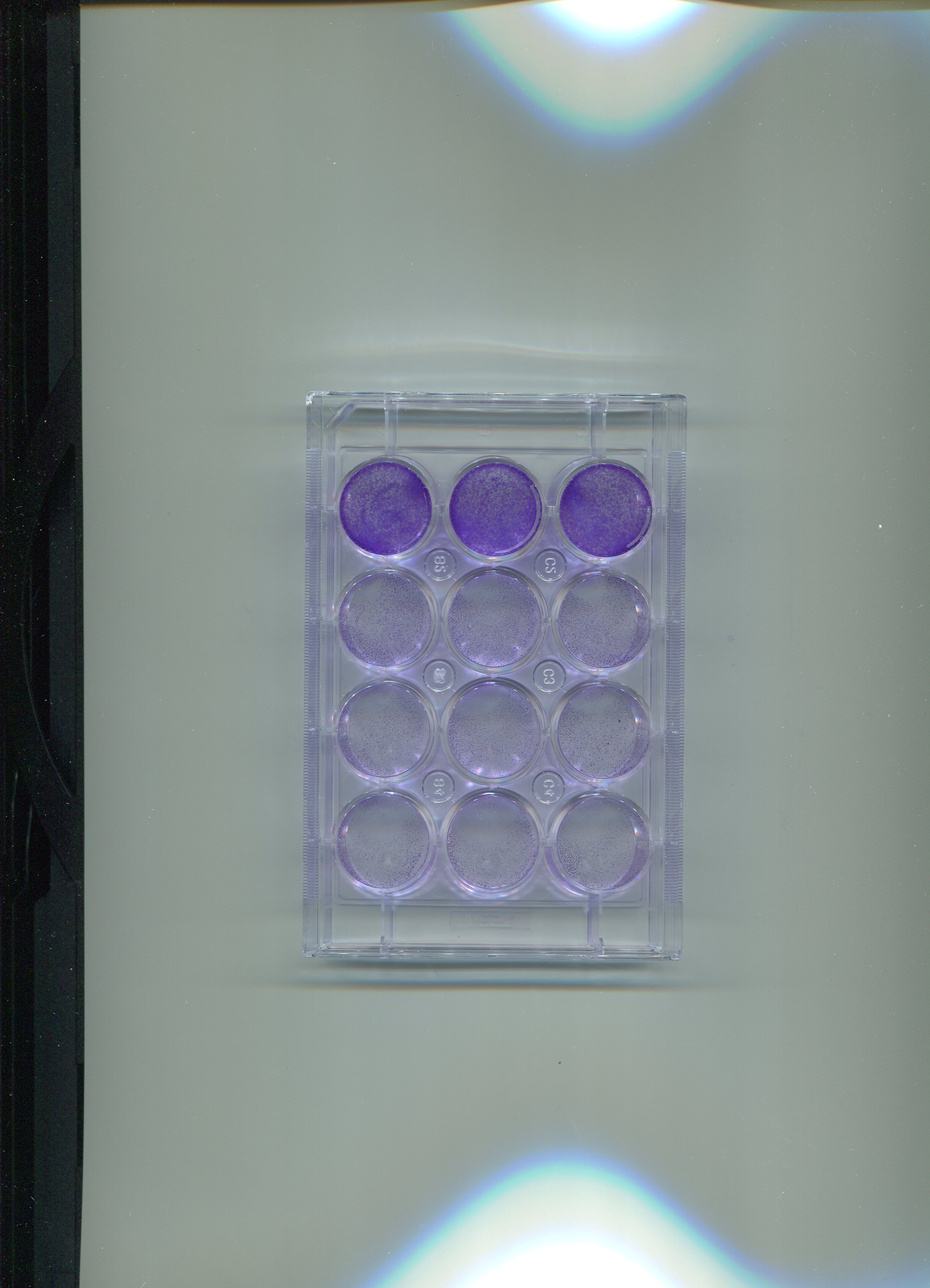

Supplement: Supplementary file 18 [file DataSheet_11.zip › other raw data/figure 1a/fig.1a.4T1-3day.tif]

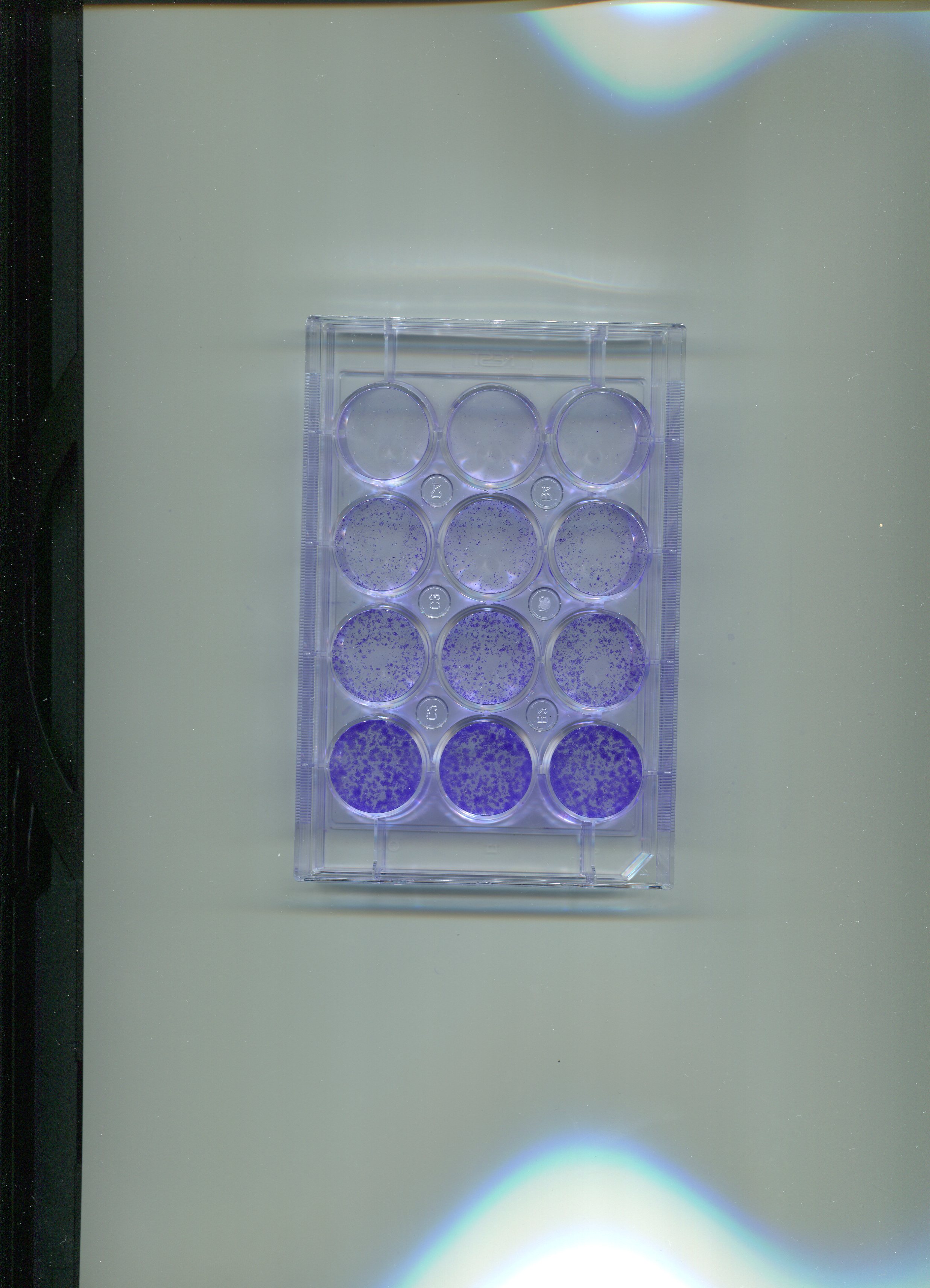

Supplement: Supplementary file 18 [file DataSheet_11.zip › other raw data/figure 1a/fig.1a.4T1-6day.tif]

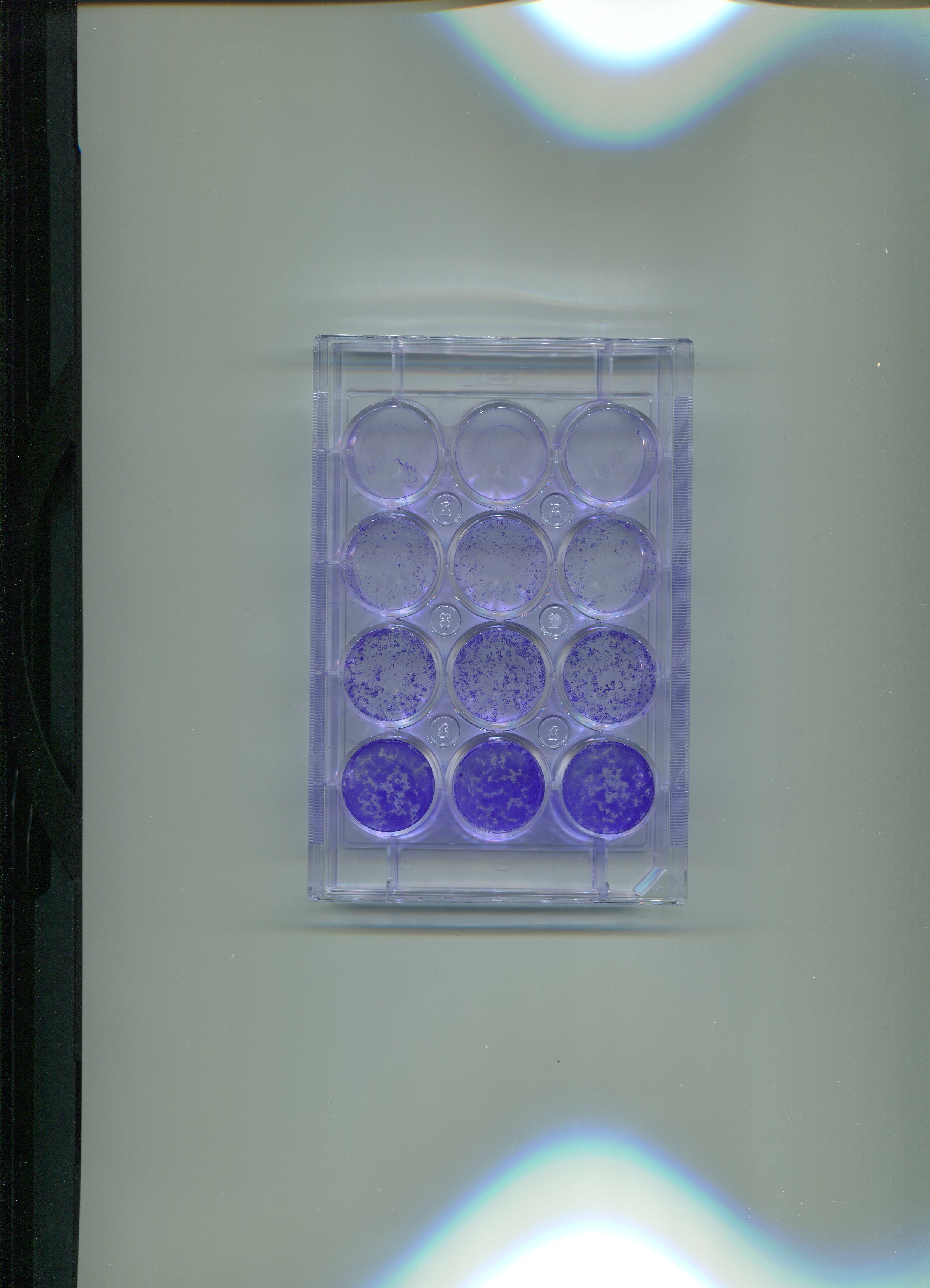

Supplement: Supplementary file 18 [file DataSheet_11.zip › other raw data/figure 1a/fig.1a.4T1-9day.tif]

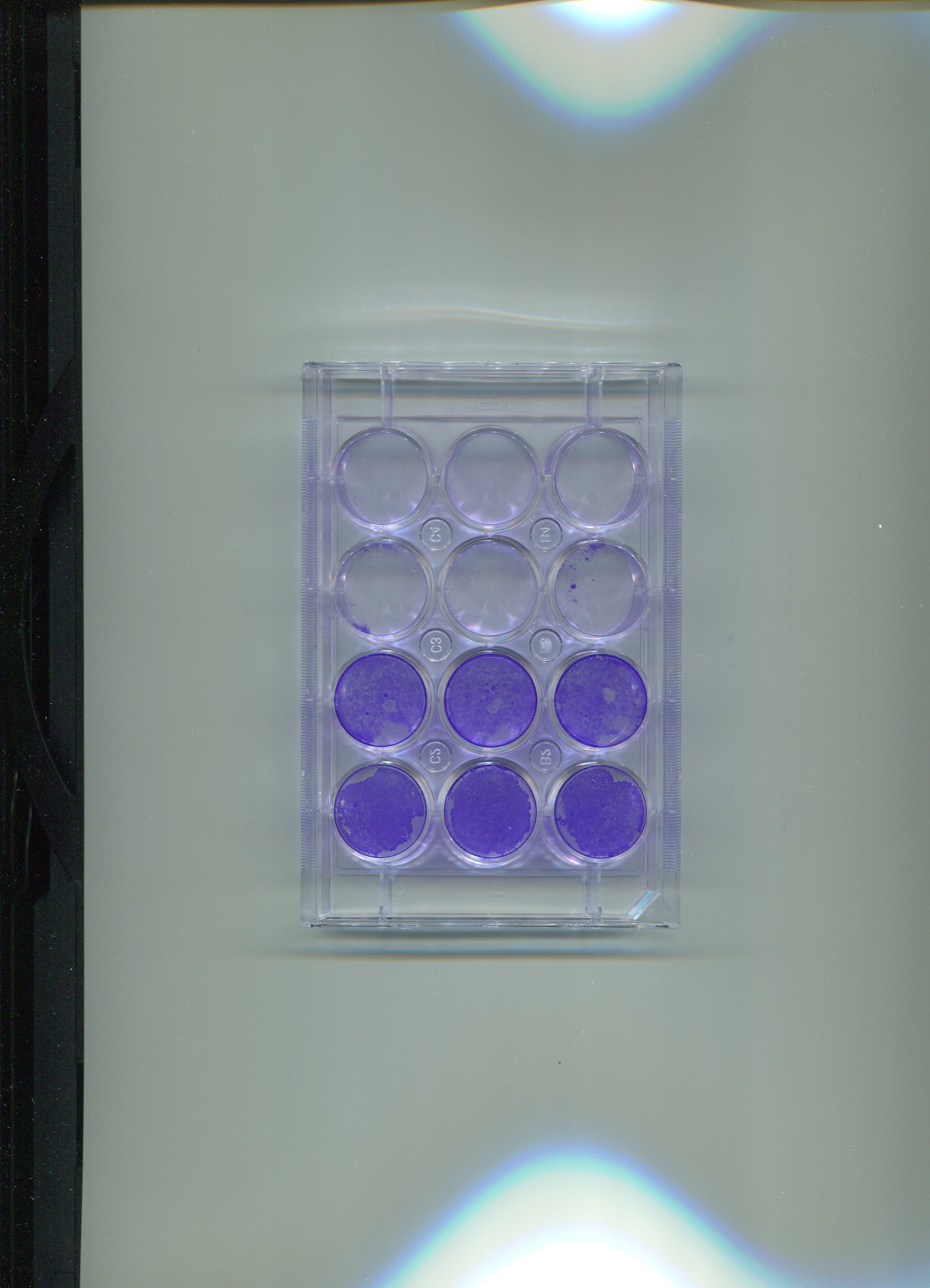

Supplement: Supplementary file 18 [file DataSheet_11.zip › other raw data/figure 1a/fig.1a.HCC1187-12day.tif]

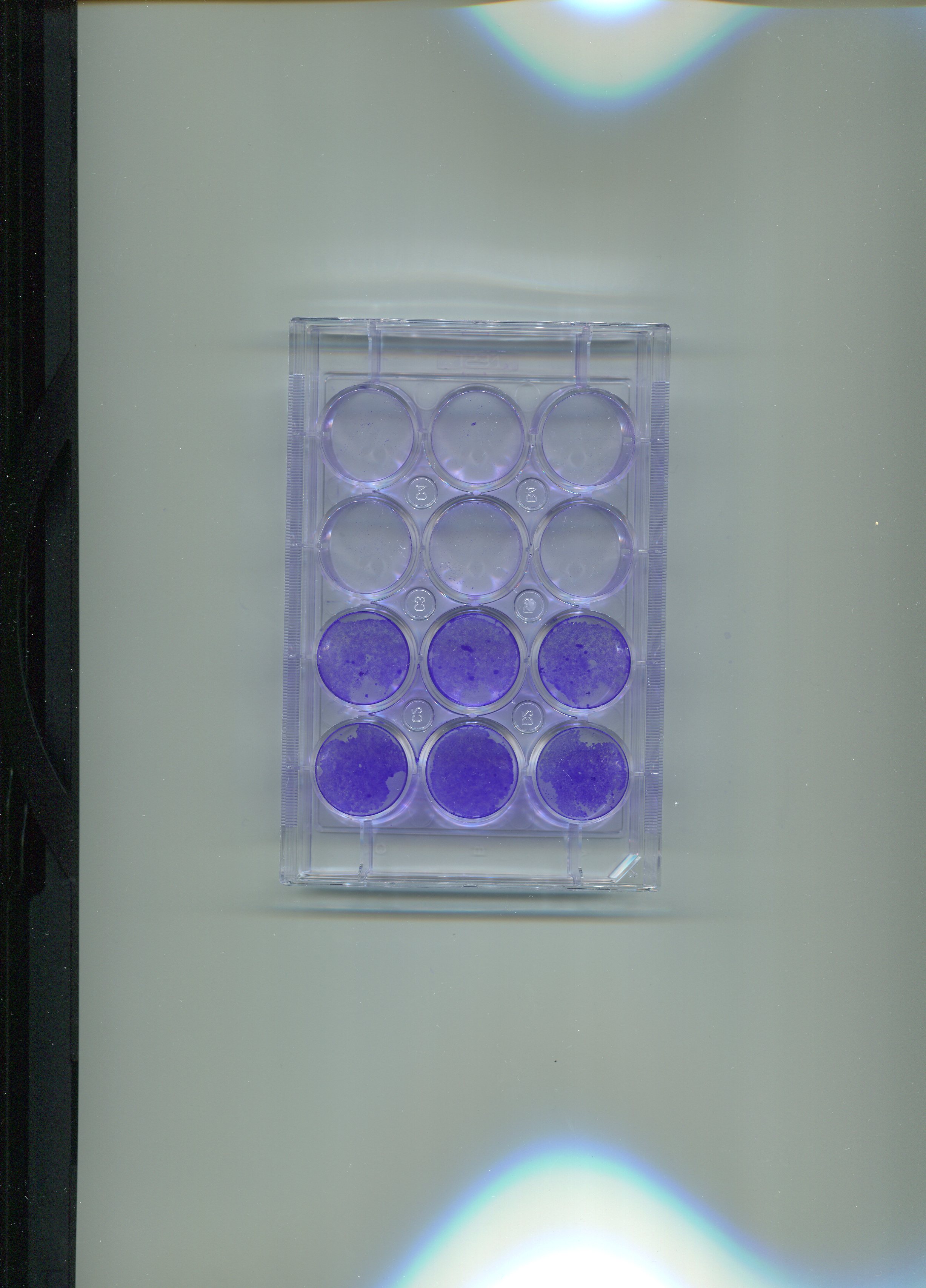

Supplement: Supplementary file 18 [file DataSheet_11.zip › other raw data/figure 1a/fig.1a.HCC1187-15day.tif]

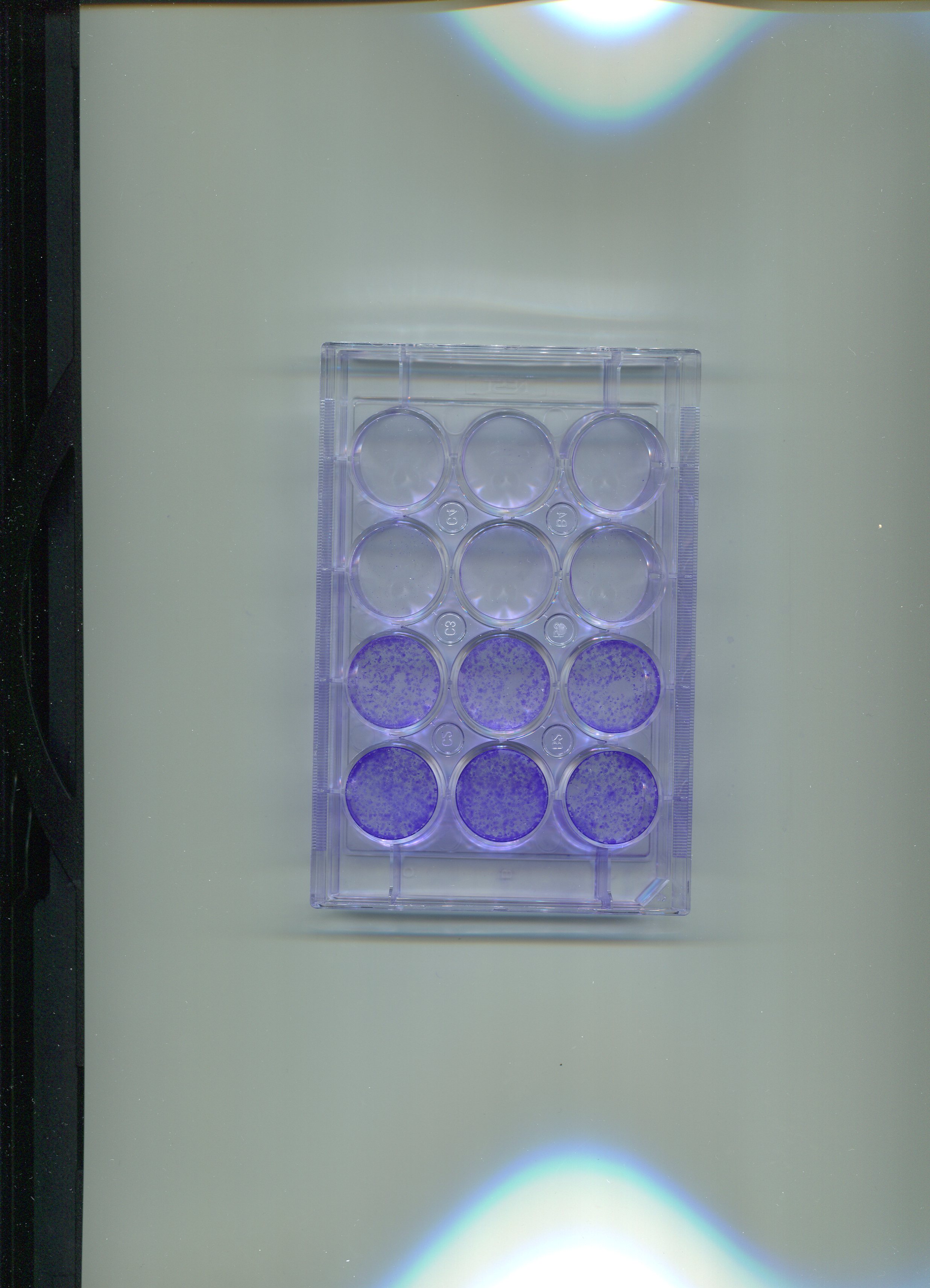

Supplement: Supplementary file 18 [file DataSheet_11.zip › other raw data/figure 1a/fig.1a.HCC1187-3day.tif]

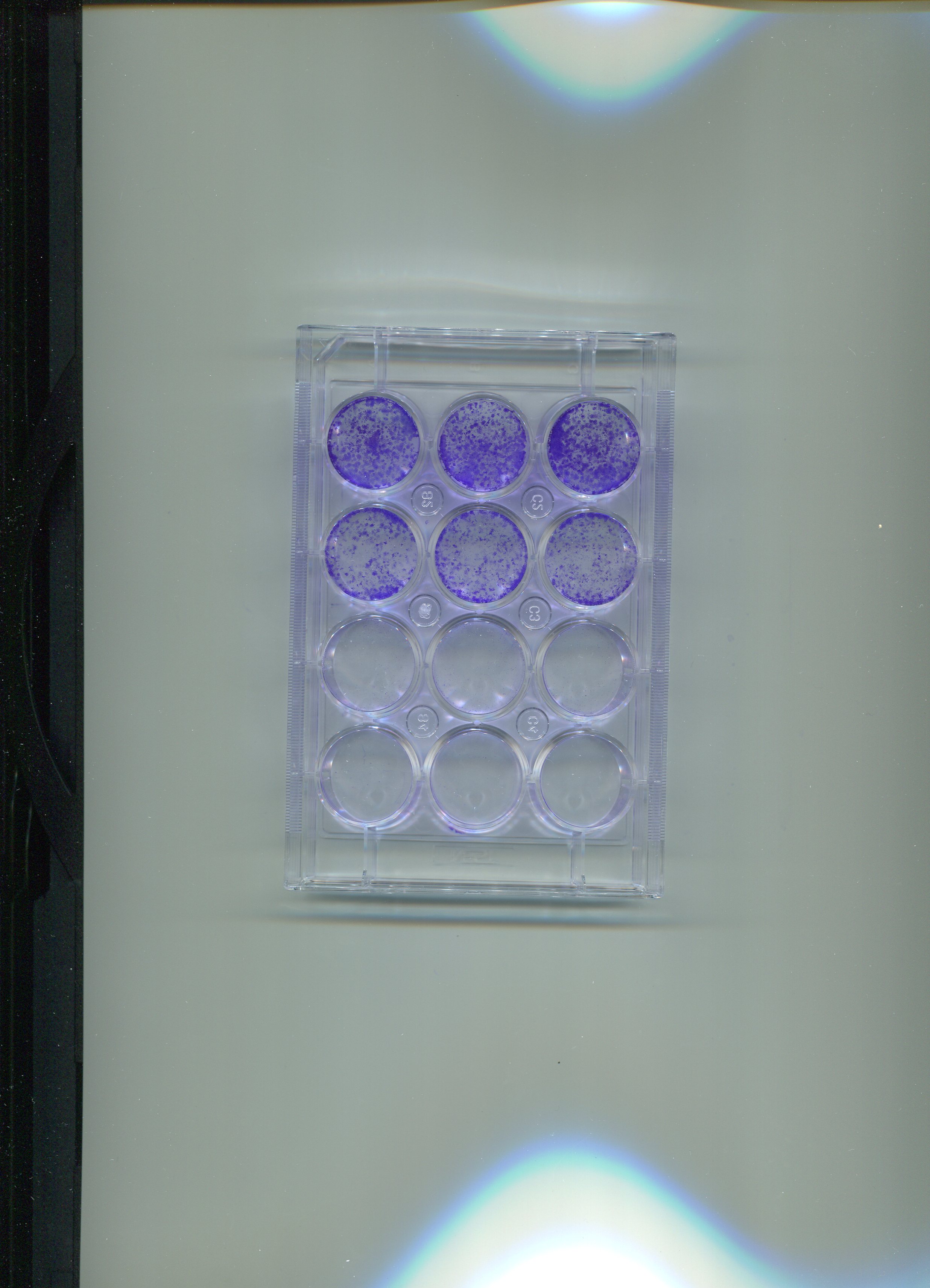

Supplement: Supplementary file 18 [file DataSheet_11.zip › other raw data/figure 1a/fig.1a.HCC1187-6day.tif]

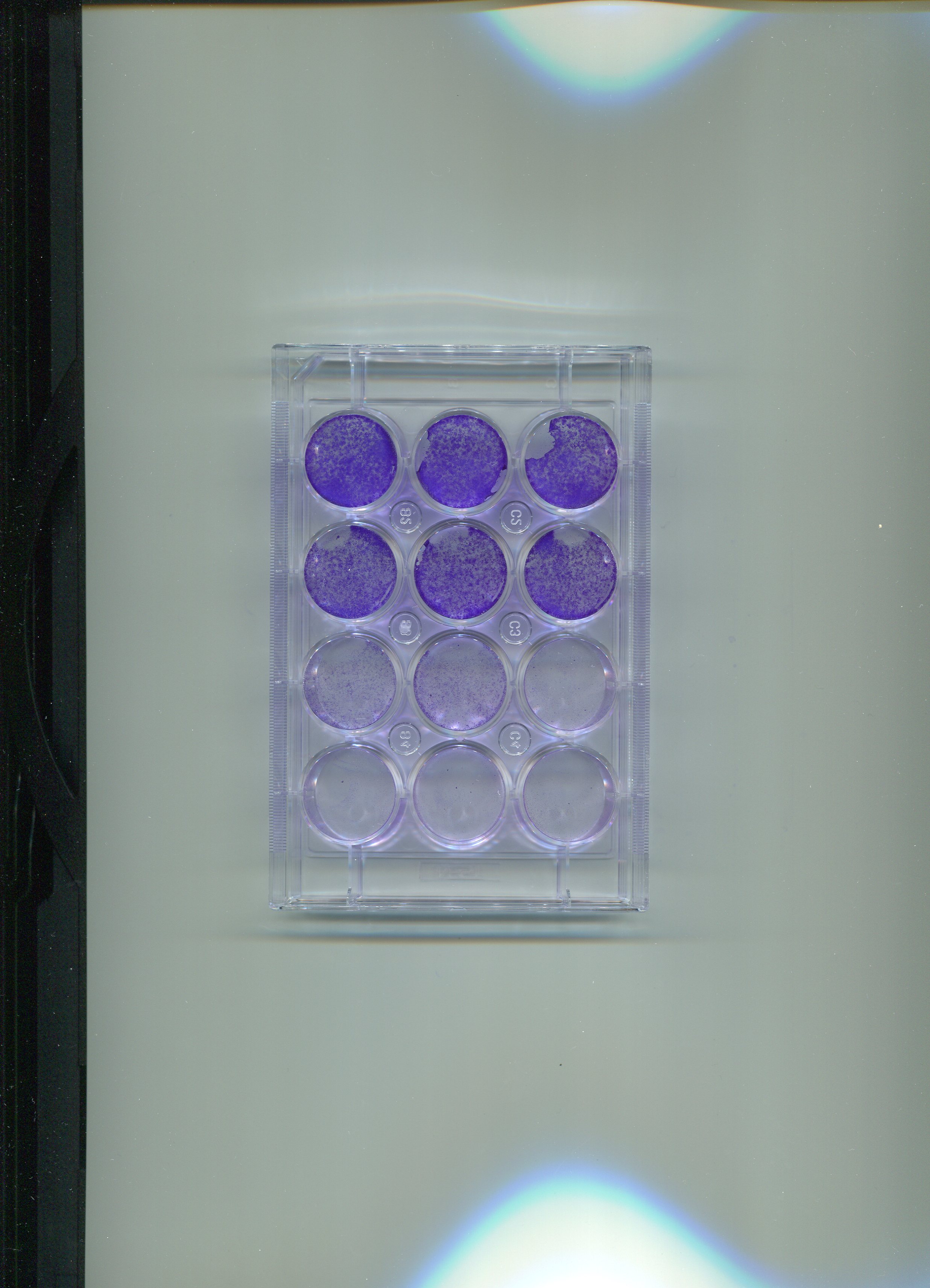

Supplement: Supplementary file 18 [file DataSheet_11.zip › other raw data/figure 1a/fig.1a.HCC1187-9day.tif]

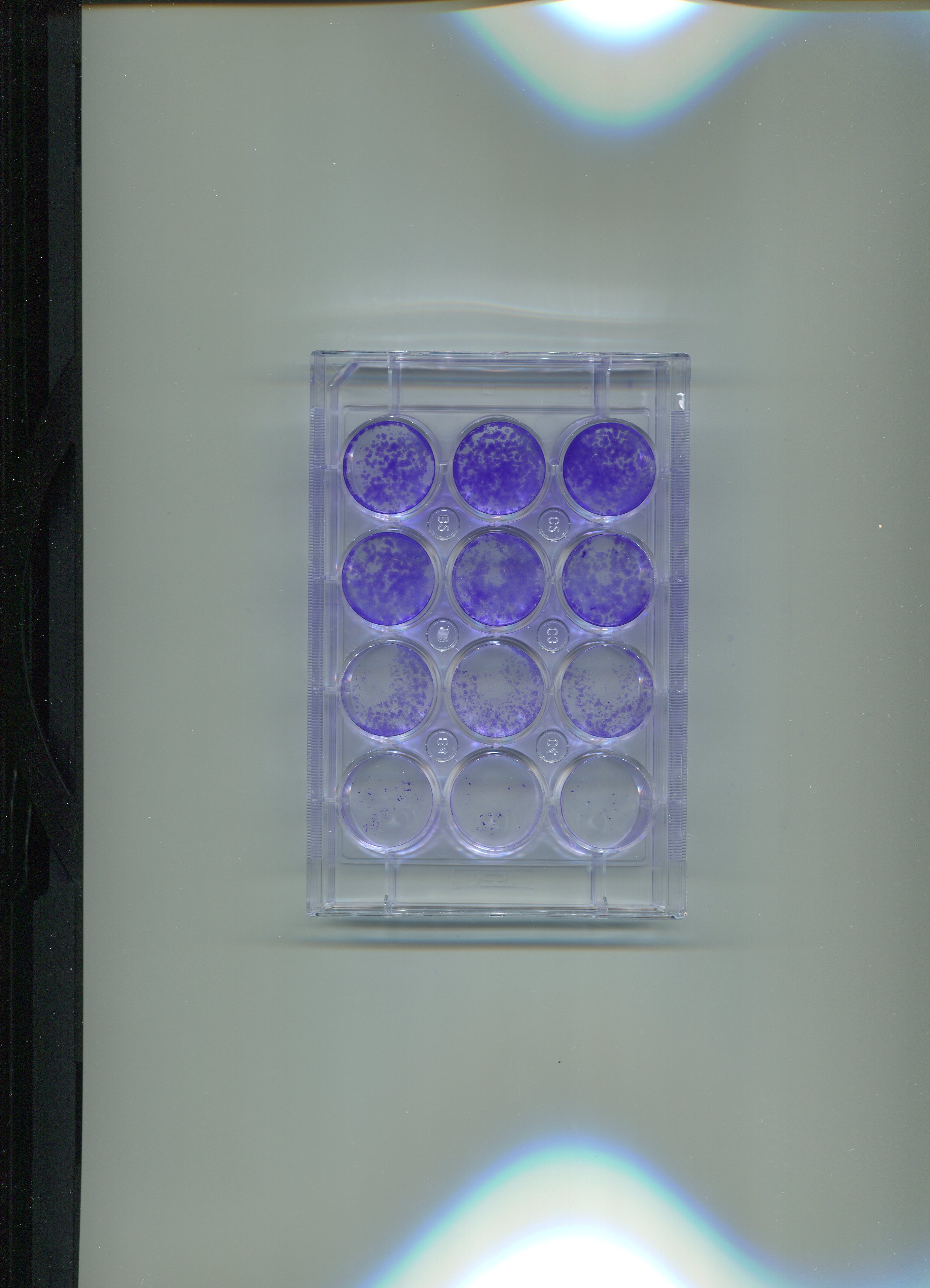

Supplement: Supplementary file 18 [file DataSheet_11.zip › other raw data/figure 1a/fig.1a.MDAMB231-12day.tif]

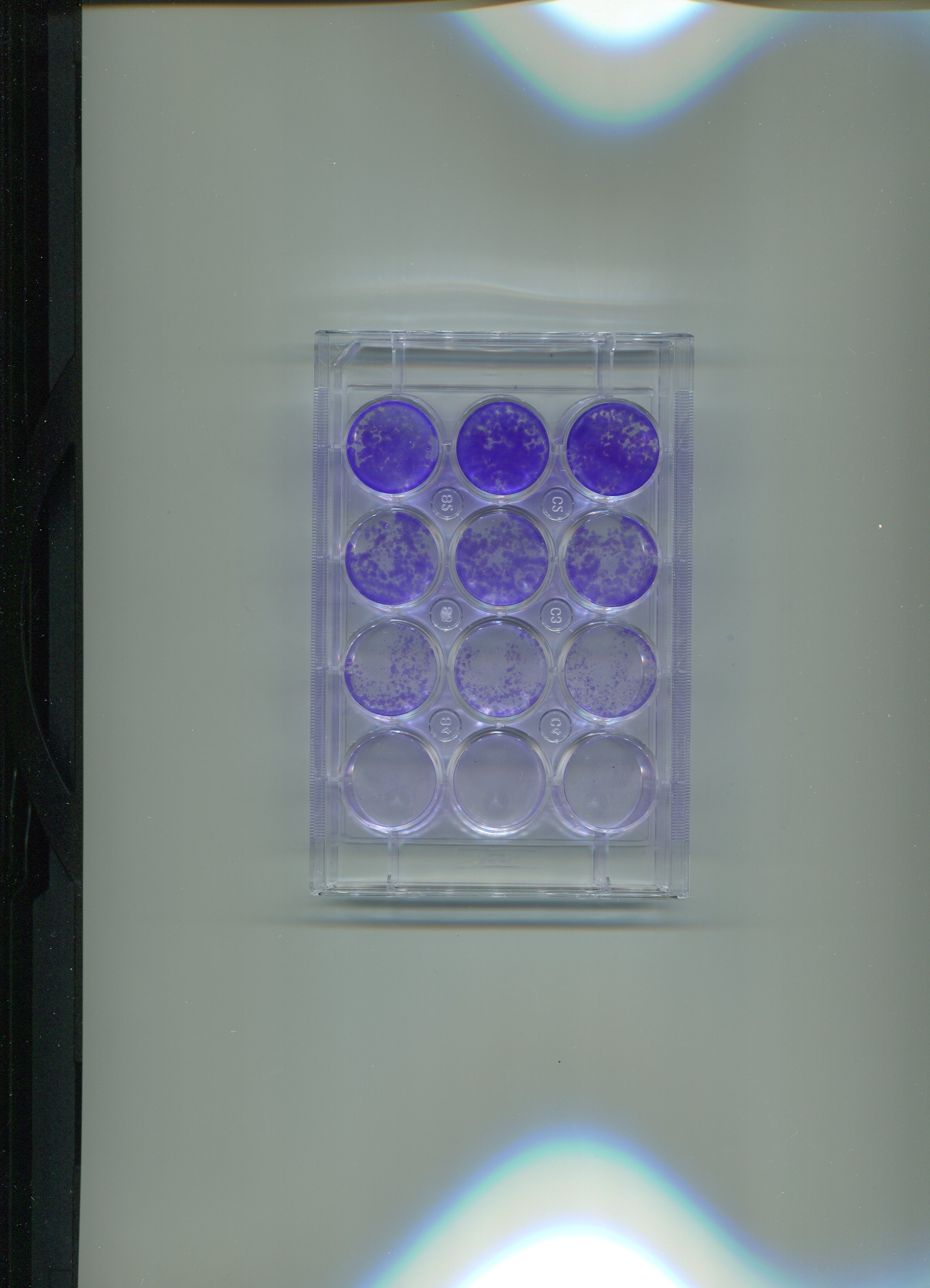

Supplement: Supplementary file 18 [file DataSheet_11.zip › other raw data/figure 1a/fig.1a.MDAMB231-15day.tif]

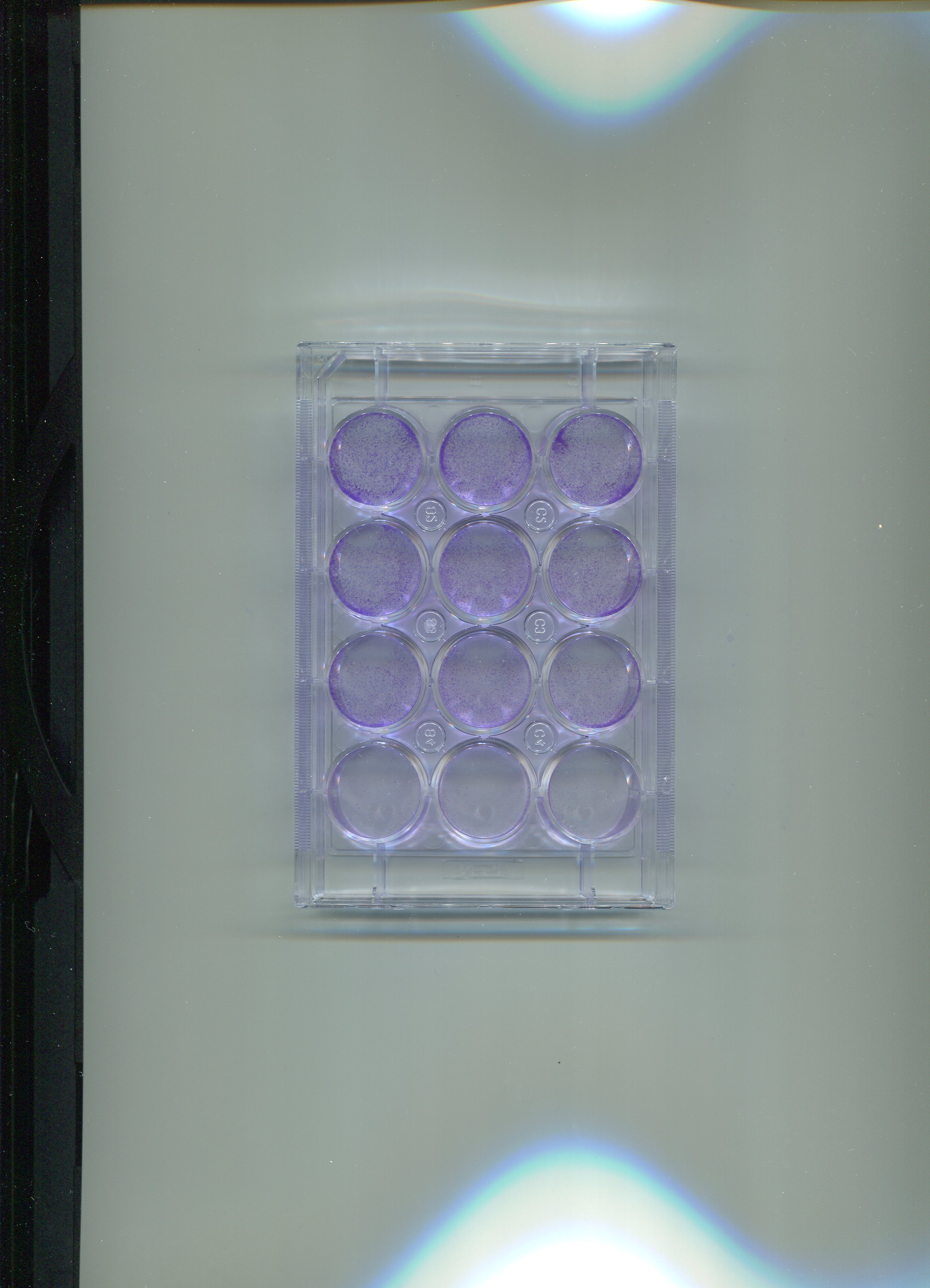

Supplement: Supplementary file 18 [file DataSheet_11.zip › other raw data/figure 1a/fig.1a.MDAMB231-3day.tif]

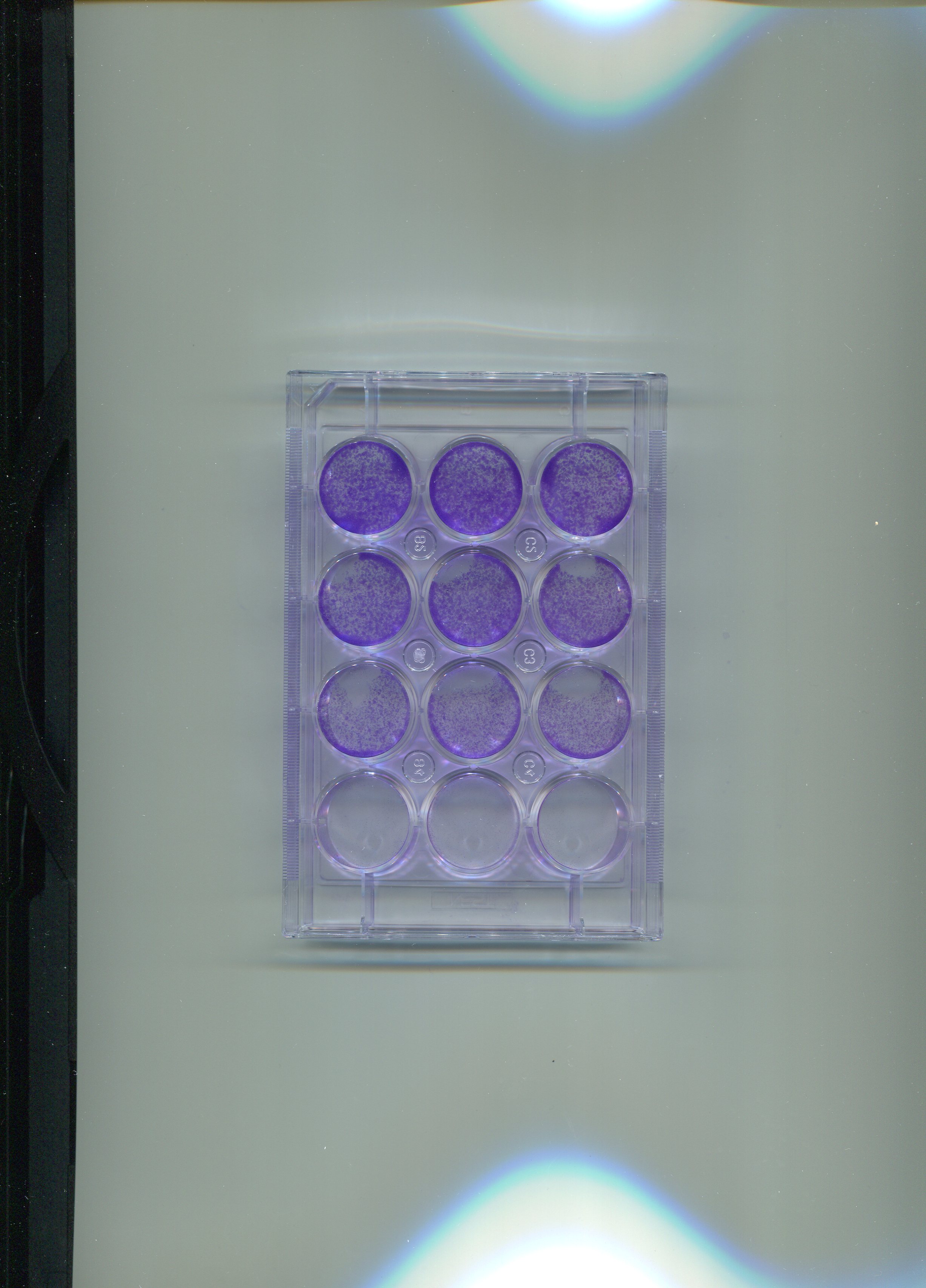

Supplement: Supplementary file 18 [file DataSheet_11.zip › other raw data/figure 1a/fig.1a.MDAMB231-6day.tif]

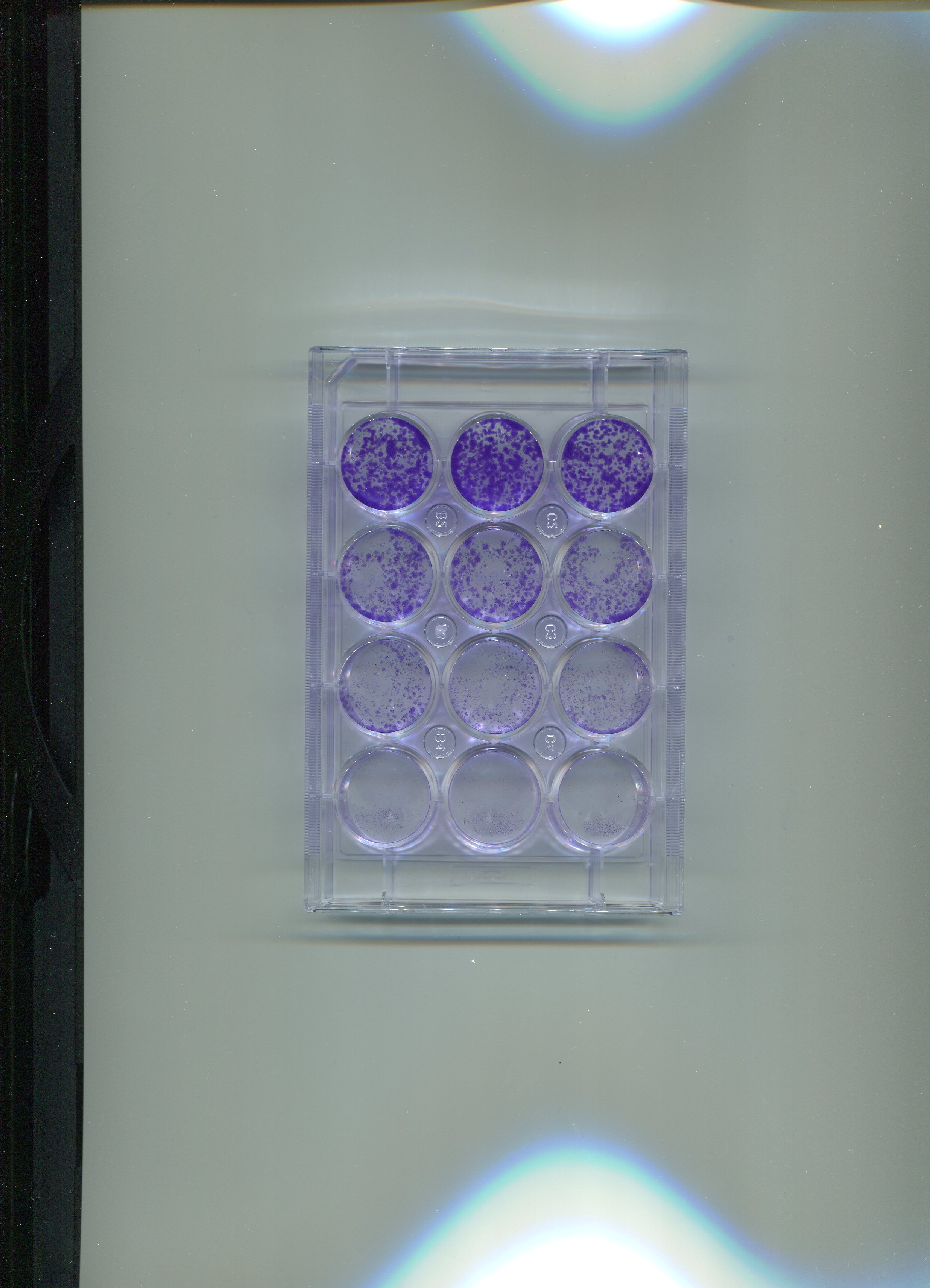

Supplement: Supplementary file 18 [file DataSheet_11.zip › other raw data/figure 1a/fig.1a.MDAMB231-9day.tif]

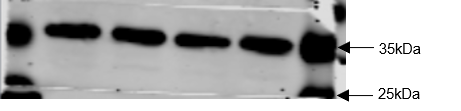

Supplement: Supplementary file 18 [file DataSheet_11.zip › other raw data/figure 2c/fig.2c.4T1_GAPDH.tif]

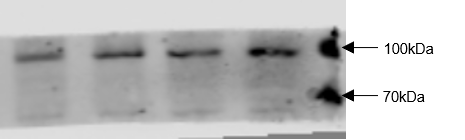

Supplement: Supplementary file 18 [file DataSheet_11.zip › other raw data/figure 2c/fig.2c.4T1_cleaved PARP.tif]

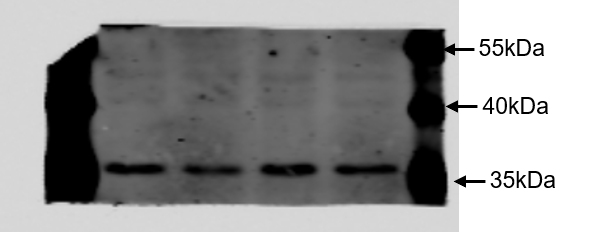

Supplement: Supplementary file 18 [file DataSheet_11.zip › other raw data/figure 2c/fig.2c.HCC1187_GAPDH.tif]

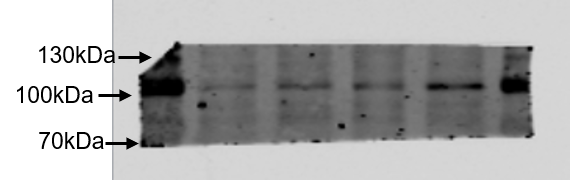

Supplement: Supplementary file 18 [file DataSheet_11.zip › other raw data/figure 2c/fig.2c.HCC1187_cleaved PARP.tif]

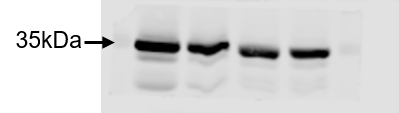

Supplement: Supplementary file 18 [file DataSheet_11.zip › other raw data/figure 2c/fig.2c.MDAMB231_GAPDH.tif]

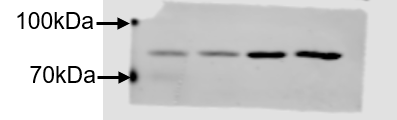

Supplement: Supplementary file 18 [file DataSheet_11.zip › other raw data/figure 2c/fig.2c.MDAMB231_cleaved PARP.tif]

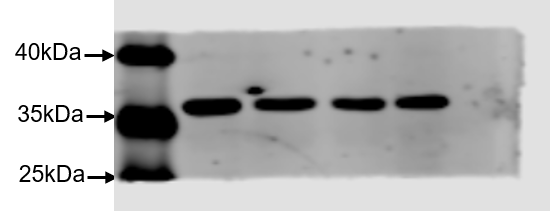

Supplement: Supplementary file 18 [file DataSheet_11.zip › other raw data/figure 4c/fig.4c.4T1_GAPDH.tif]

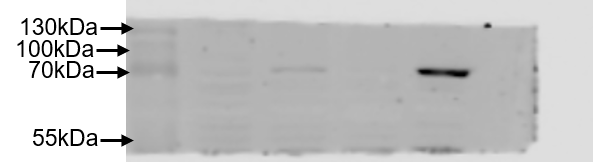

Supplement: Supplementary file 18 [file DataSheet_11.zip › other raw data/figure 4c/fig.4c.4T1_cleaved PARP.tif]

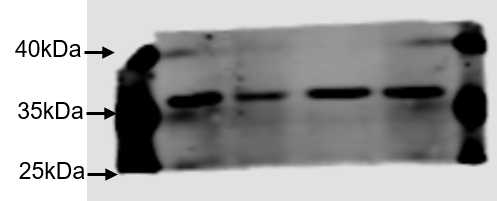

Supplement: Supplementary file 18 [file DataSheet_11.zip › other raw data/figure 4c/fig.4c.HCC1187_GAPDH.tif]

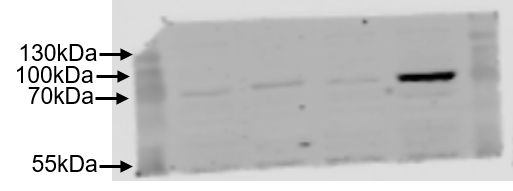

Supplement: Supplementary file 18 [file DataSheet_11.zip › other raw data/figure 4c/fig.4c.HCC1187_cleaved PARP.tif]

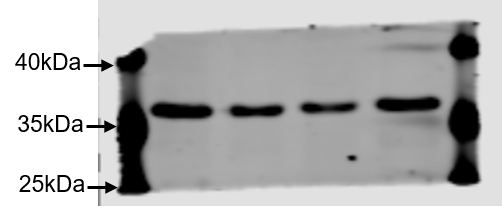

Supplement: Supplementary file 18 [file DataSheet_11.zip › other raw data/figure 4c/fig.4c.MDAMB231_GAPDH.tif]

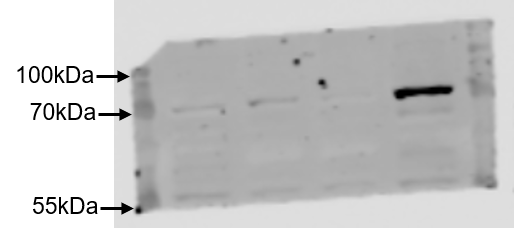

Supplement: Supplementary file 18 [file DataSheet_11.zip › other raw data/figure 4c/fig.4c.MDAMB231_cleaved PARP.tif]

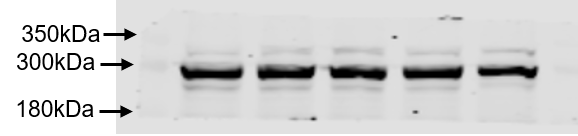

Supplement: Supplementary file 18 [file DataSheet_11.zip › other raw data/figure 7a/fig.7a.ATM.tif]

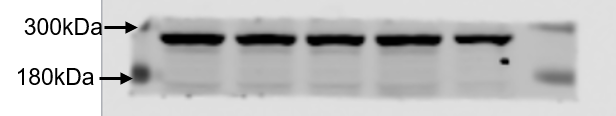

Supplement: Supplementary file 18 [file DataSheet_11.zip › other raw data/figure 7a/fig.7a.ATR.tif]

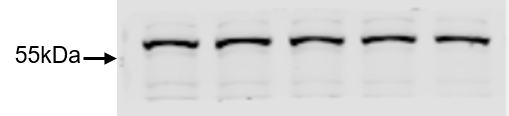

Supplement: Supplementary file 18 [file DataSheet_11.zip › other raw data/figure 7a/fig.7a.CHK1.tif]

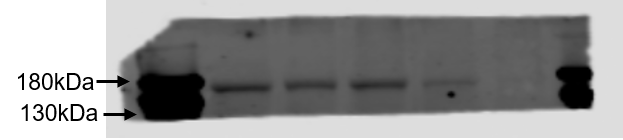

Supplement: Supplementary file 18 [file DataSheet_11.zip › other raw data/figure 7a/fig.7a.FANCD2.tif]

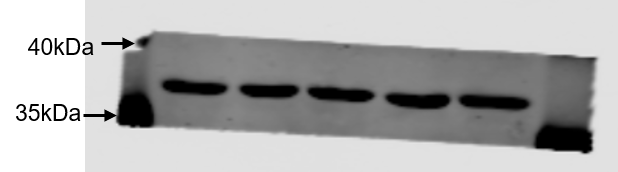

Supplement: Supplementary file 18 [file DataSheet_11.zip › other raw data/figure 7a/fig.7a.GAPDH.tif]

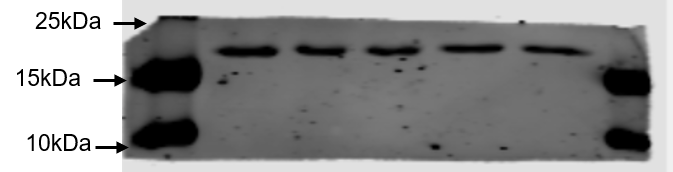

Supplement: Supplementary file 18 [file DataSheet_11.zip › other raw data/figure 7a/fig.7a.H2AX.tif]

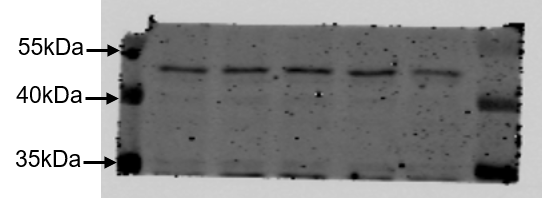

Supplement: Supplementary file 18 [file DataSheet_11.zip › other raw data/figure 7a/fig.7a.P53.tif]

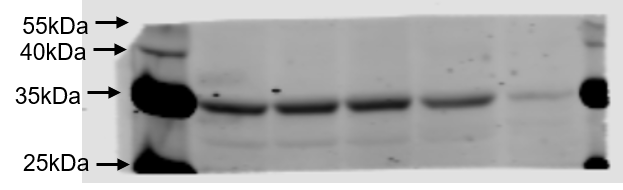

Supplement: Supplementary file 18 [file DataSheet_11.zip › other raw data/figure 7a/fig.7a.RAD51.tif]

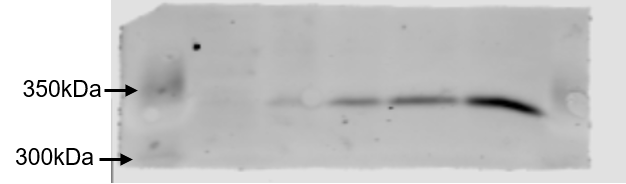

Supplement: Supplementary file 18 [file DataSheet_11.zip › other raw data/figure 7a/fig.7a.p-ATM.tif]

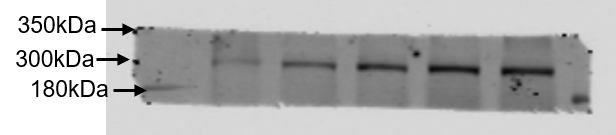

Supplement: Supplementary file 18 [file DataSheet_11.zip › other raw data/figure 7a/fig.7a.p-ATR.tif]

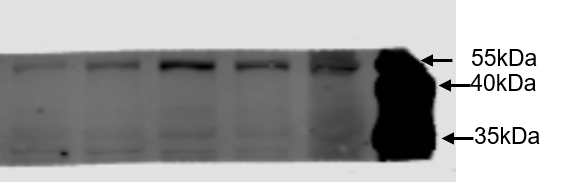

Supplement: Supplementary file 18 [file DataSheet_11.zip › other raw data/figure 7a/fig.7a.p-CHK1.tif]

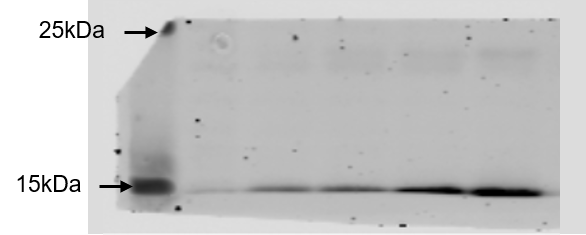

Supplement: Supplementary file 18 [file DataSheet_11.zip › other raw data/figure 7a/fig.7a.p-H2AX.tif]

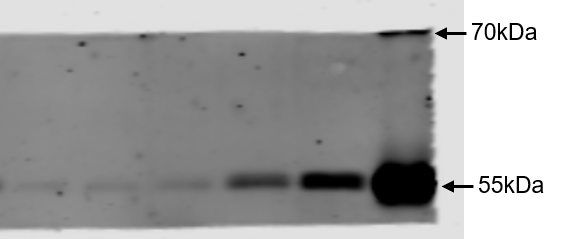

Supplement: Supplementary file 18 [file DataSheet_11.zip › other raw data/figure 7a/fig.7a.p-P53.tif]

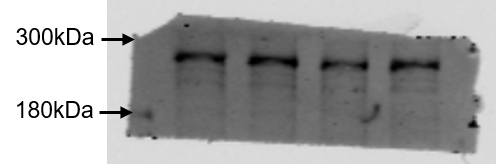

Supplement: Supplementary file 18 [file DataSheet_11.zip › other raw data/figure 7b/fig.7b.ATM.tif]

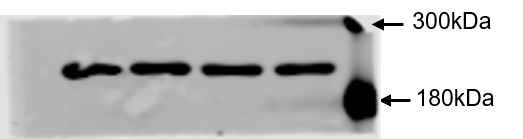

Supplement: Supplementary file 18 [file DataSheet_11.zip › other raw data/figure 7b/fig.7b.ATR.tif]

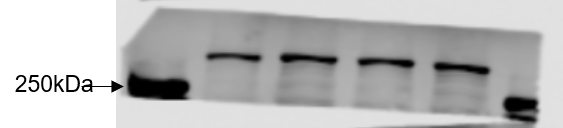

Supplement: Supplementary file 18 [file DataSheet_11.zip › other raw data/figure 7b/fig.7b.CHK1.tif]

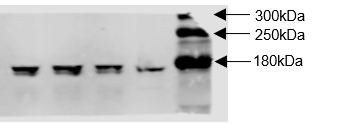

Supplement: Supplementary file 18 [file DataSheet_11.zip › other raw data/figure 7b/fig.7b.FANCD2.tif]

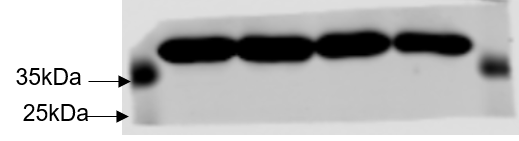

Supplement: Supplementary file 18 [file DataSheet_11.zip › other raw data/figure 7b/fig.7b.GAPDH.tif]

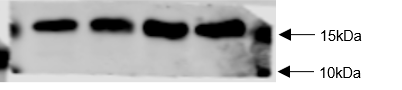

Supplement: Supplementary file 18 [file DataSheet_11.zip › other raw data/figure 7b/fig.7b.H2AX.tif]

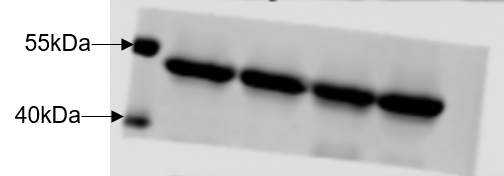

Supplement: Supplementary file 18 [file DataSheet_11.zip › other raw data/figure 7b/fig.7b.P53.tif]

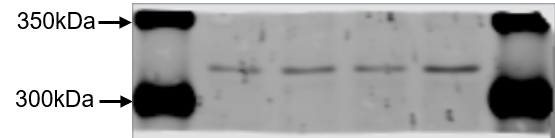

Supplement: Supplementary file 18 [file DataSheet_11.zip › other raw data/figure 7b/fig.7b.p-ATM.tif]

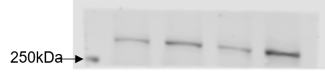

Supplement: Supplementary file 18 [file DataSheet_11.zip › other raw data/figure 7b/fig.7b.p-ATR.tif]
