# Supplementary material for: Salvia chinensis Benth Inhibits Triple-Negative Breast Cancer Progression by Inducing the DNA Damage Pathway
Source: Front Oncol. 2022 Aug 10;12:882784. doi: 10.3389/fonc.2022.882784 (PMC9404549; doi:10.3389/fonc.2022.882784)
Supplement: Supplementary file 18 [file DataSheet_11.zip › other raw data/figure 2a/1.MDAMB231-V1.pdf]

BD FACSDiva 8.0.1

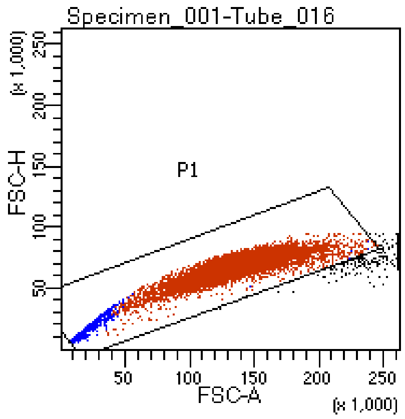

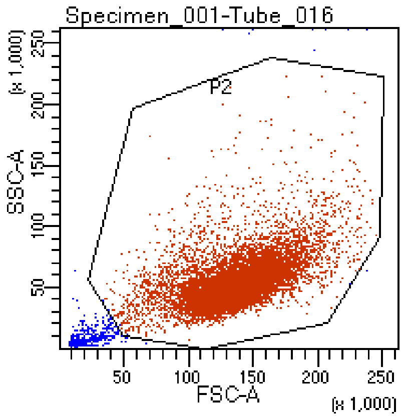

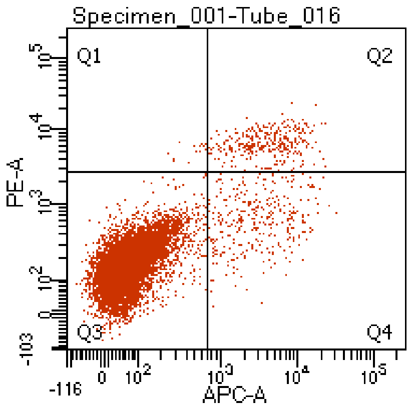

Tube: Tube_016

Population	#Events	%Parent	%Total
All Events	11,203	####	100.0
P1	10,671	95.3	95.3
P2	10,036	94.0	89.6
Q1	14	0.1	0.1
Q2	338	3.4	3.0
Q3	9,363	93.3	83.6
Q4	321	3.2	2.9

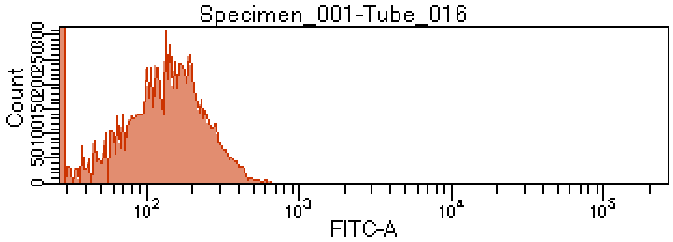

Tube Name: Tube_016
GUID: ffb3fff1-c91f-443a-8d8b-458494e6fd76

Population	#Events	%Parent	PE-A Mean	PE-A %CV	APC-A Mean	APC-A %CV	APC-Cy7-A Mean	APC-Cy7-A %CV
All Events	11,203	####	486	363.2	429	418.9	254	440.2
P1	10,671	95.3	449	312.5	411	406.0	242	425.6
P2	10,036	94.0	454	306.4	394	415.0	232	436.0
Q1	14	0.1	5,169	24.4	469	31.3	279	39.1
Q2	338	3.4	7,141	41.7	5,356	77.7	3,259	81.4
Q3	9,363	93.3	194	77.3	75	108.7	39	131.9
Q4	321	3.2	781	75.9	4,464	99.3	2,676	104.0
