# Supplementary material for: Salvia chinensis Benth Inhibits Triple-Negative Breast Cancer Progression by Inducing the DNA Damage Pathway
Source: Front Oncol. 2022 Aug 10;12:882784. doi: 10.3389/fonc.2022.882784 (PMC9404549; doi:10.3389/fonc.2022.882784)
Supplement: Supplementary file 18 [file DataSheet_11.zip › other raw data/figure 2a/10.MDAMB231-200mg-1.pdf]

BD FACSDiva 8.0.1

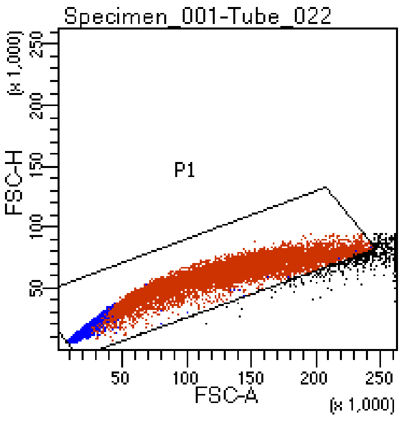

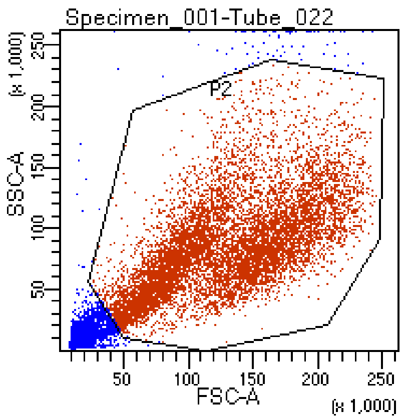

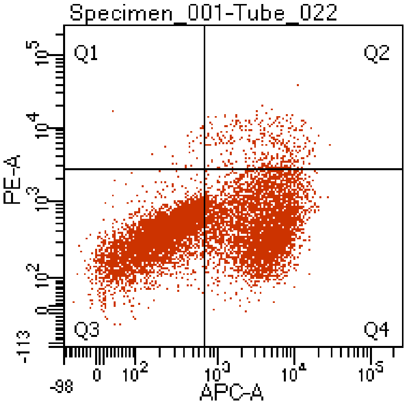

Tube: Tube_022

Population	#Events	%Parent	%Total
All Events	14,359	####	100.0
P1	13,065	91.0	91.0
P2	9,741	74.6	67.8
Q1	28	0.3	0.2
Q2	400	4.1	2.8
Q3	4,952	50.8	34.5
Q4	4,361	44.8	30.4

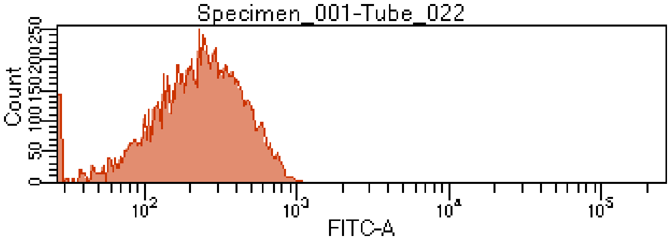

Tube Name: Tube_022
GUID: cbea945f-292c-4502-9069-05b5eb93169b

Population	#Events	%Parent	PE-A Mean	PE-A %CV	APC-A Mean	APC-A %CV	APC-Cy7-A Mean	APC-Cy7-A %CV
All Events	14,359	####	668	262.2	1,948	168.7	1,168	173.8
P1	13,065	91.0	630	219.1	1,947	146.5	1,166	151.0
P2	9,741	74.6	768	193.9	2,318	132.3	1,390	136.6
Q1	28	0.3	5,976	55.1	453	38.8	261	42.5
Q2	400	4.1	6,302	61.6	5,657	70.9	3,544	73.5
Q3	4,952	50.8	389	59.2	230	70.5	127	75.7
Q4	4,361	44.8	658	81.9	4,394	69.0	2,634	72.3
