# Supplementary material for: Salvia chinensis Benth Inhibits Triple-Negative Breast Cancer Progression by Inducing the DNA Damage Pathway
Source: Front Oncol. 2022 Aug 10;12:882784. doi: 10.3389/fonc.2022.882784 (PMC9404549; doi:10.3389/fonc.2022.882784)
Supplement: Supplementary file 18 [file DataSheet_11.zip › other raw data/figure 2a/11.MDAMB231-200mg-2.pdf]

BD FACSDiva 8.0.1

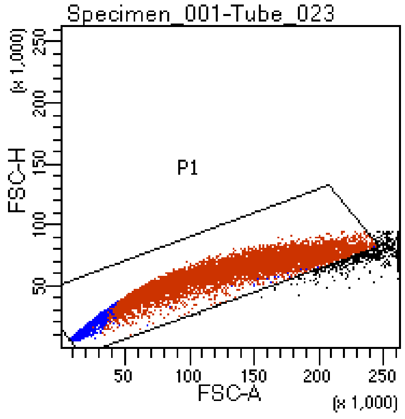

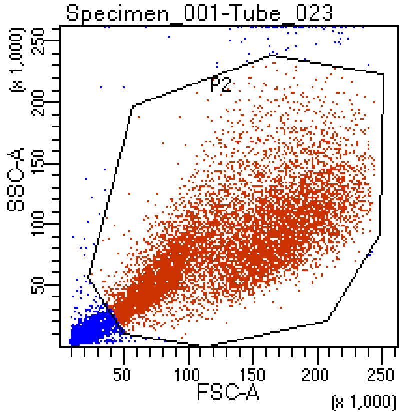

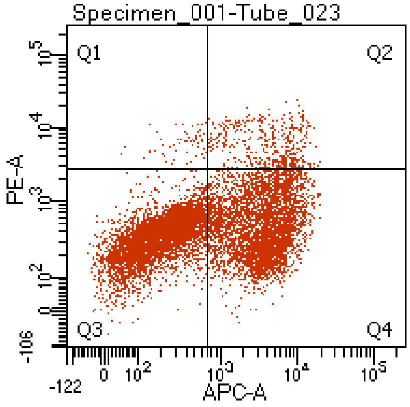

Tube: Tube_023

Population	#Events	%Parent	%Total
All Events	13,574	####	100.0
P1	12,311	90.7	90.7
P2	9,827	79.8	72.4
Q1	61	0.6	0.4
Q2	465	4.7	3.4
Q3	5,394	54.9	39.7
Q4	3,907	39.8	28.8

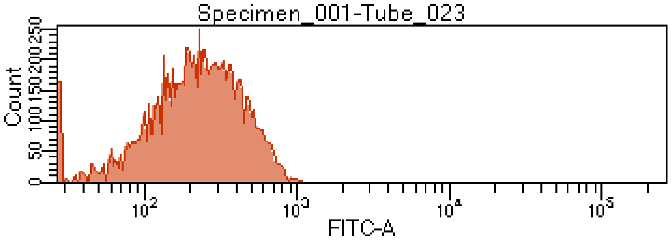

Tube Name: Tube_023
GUID: 33ae7fd2-48c6-4b1f-bdae-85361994ed15

Population	#Events	%Parent	PE-A Mean	PE-A %CV	APC-A Mean	APC-A %CV	APC-Cy7-A Mean	APC-Cy7-A %CV
All Events	13,574	####	750	238.2	1,758	162.3	1,043	167.4
P1	12,311	90.7	730	219.1	1,813	150.5	1,076	155.1
P2	9,827	79.8	830	197.5	2,019	140.6	1,198	145.0
Q1	61	0.6	6,541	35.9	395	43.3	239	45.7
Q2	465	4.7	6,469	58.6	5,484	68.6	3,382	70.4
Q3	5,394	54.9	408	58.1	226	70.5	123	76.0
Q4	3,907	39.8	653	87.1	4,108	70.0	2,438	73.3
