# Supplementary material for: Salvia chinensis Benth Inhibits Triple-Negative Breast Cancer Progression by Inducing the DNA Damage Pathway
Source: Front Oncol. 2022 Aug 10;12:882784. doi: 10.3389/fonc.2022.882784 (PMC9404549; doi:10.3389/fonc.2022.882784)
Supplement: Supplementary file 18 [file DataSheet_11.zip › other raw data/figure 2a/12.MDAMB231-200mg-3.pdf]

BD FACSDiva 8.0.1

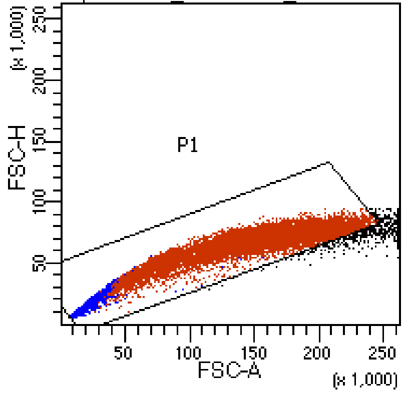

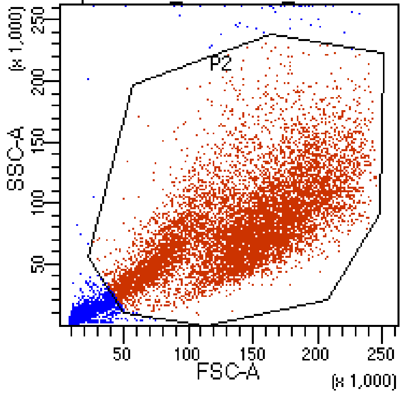

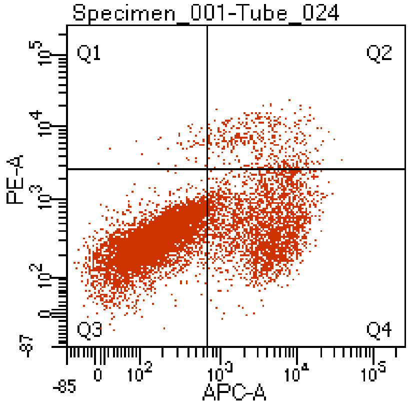

Tube: Tube_024

Population	#Events	%Parent	%Total
All Events	13,291	####	100.0
P1	12,129	91.3	91.3
P2	9,948	82.0	74.8
Q1	45	0.5	0.3
Q2	433	4.4	3.3
Q3	6,876	69.1	51.7
Q4	2,594	26.1	19.5

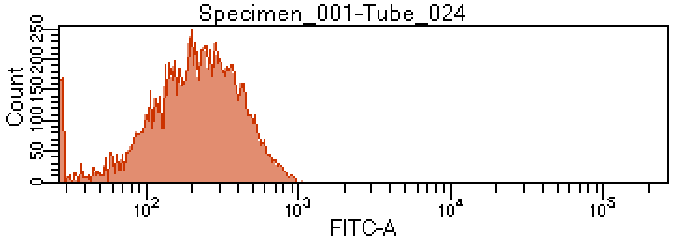

Tube Name: Tube_024
GUID: a62d4905-6e04-4c03-8b3c-09187c9ea9b2

Population	#Events	%Parent	PE-A Mean	PE-A %CV	APC-A Mean	APC-A %CV	APC-Cy7-A Mean	APC-Cy7-A %CV
All Events	13,291	####	710	287.5	1,476	203.9	879	210.9
P1	12,129	91.3	666	239.8	1,491	192.5	887	198.9
P2	9,948	82.0	750	224.4	1,606	186.0	956	192.1
Q1	45	0.5	5,963	33.4	417	38.8	235	41.4
Q2	433	4.4	7,160	55.1	5,176	92.3	3,197	94.3
Q3	6,876	69.1	352	58.7	195	77.1	105	84.0
Q4	2,594	26.1	644	86.6	4,771	75.4	2,848	78.9
