# Supplementary material for: Salvia chinensis Benth Inhibits Triple-Negative Breast Cancer Progression by Inducing the DNA Damage Pathway
Source: Front Oncol. 2022 Aug 10;12:882784. doi: 10.3389/fonc.2022.882784 (PMC9404549; doi:10.3389/fonc.2022.882784)
Supplement: Supplementary file 18 [file DataSheet_11.zip › other raw data/figure 2a/13.HCC187-V1.pdf]

BD FACSDiva 8.0.1

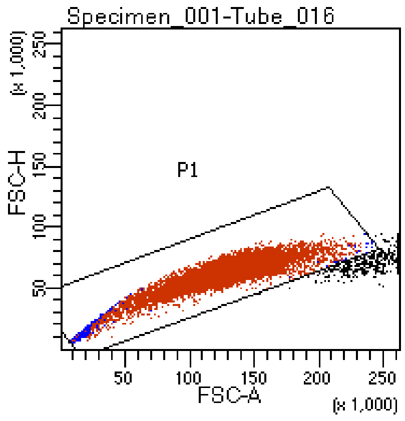

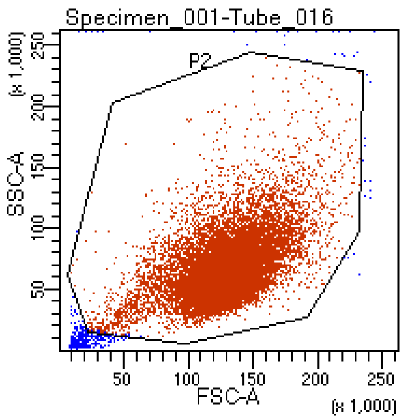

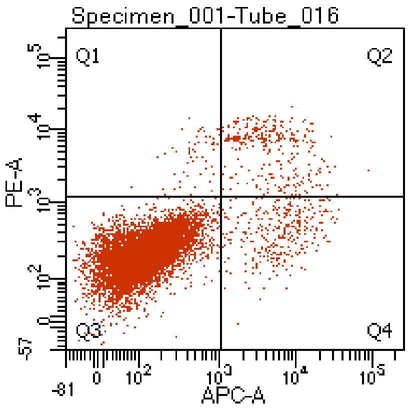

Tube: Tube_016

Population	#Events	%Parent	%Total
All Events	21,391	####	100.0
P1	20,596	96.3	96.3
P2	20,060	97.4	93.8
Q1	116	0.6	0.5
Q2	642	3.2	3.0
Q3	18,664	93.0	87.3
Q4	638	3.2	3.0

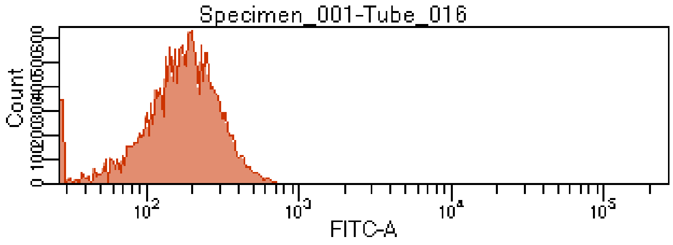

Tube Name: Tube_016
GUID: a19ba964-adcd-4707-b3fc-93f6c0e21033

Population	#Events	%Parent	PE-A Mean	PE-A %CV	APC-A Mean	APC-A %CV	APC-Cy7-A Mean	APC-Cy7-A %CV
All Events	21,391	####	518	303.0	622	400.2	360	427.3
P1	20,596	96.3	501	300.2	615	403.0	357	429.3
P2	20,060	97.4	500	296.9	605	408.5	351	435.1
Q1	116	0.6	5,433	55.6	581	43.7	331	46.6
Q2	642	3.2	7,168	55.8	6,652	102.6	3,945	108.4
Q3	18,664	93.0	241	53.3	141	86.8	72	97.8
Q4	638	3.2	499	58.7	8,077	83.7	4,878	89.2
