# Supplementary material for: Salvia chinensis Benth Inhibits Triple-Negative Breast Cancer Progression by Inducing the DNA Damage Pathway
Source: Front Oncol. 2022 Aug 10;12:882784. doi: 10.3389/fonc.2022.882784 (PMC9404549; doi:10.3389/fonc.2022.882784)
Supplement: Supplementary file 18 [file DataSheet_11.zip › other raw data/figure 2a/14.HCC1187-V2.pdf]

BD FACSDiva 8.0.1

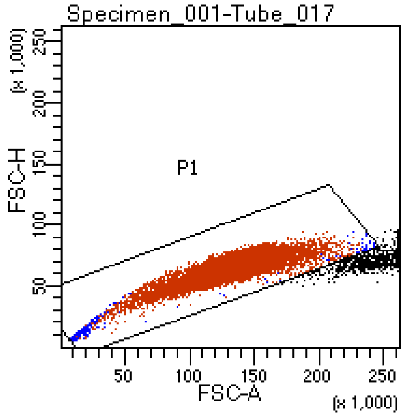

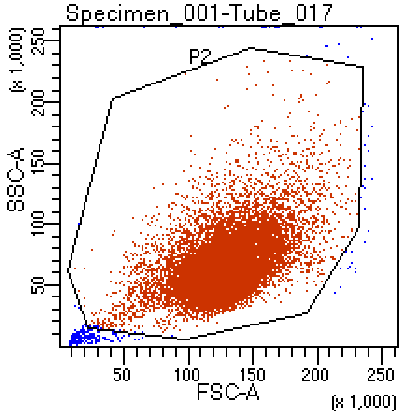

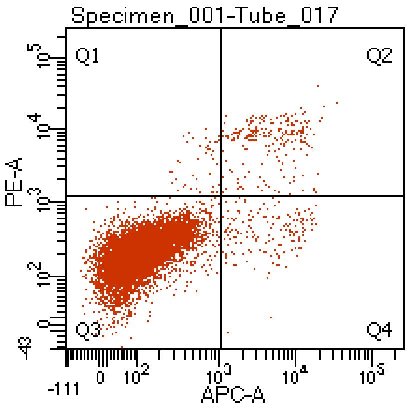

Tube: Tube_017

Population	#Events	%Parent	%Total
All Events	21,645	####	100.0
P1	20,422	94.3	94.3
P2	20,031	98.1	92.5
Q1	90	0.4	0.4
Q2	409	2.0	1.9
Q3	19,197	95.8	88.7
Q4	335	1.7	1.5

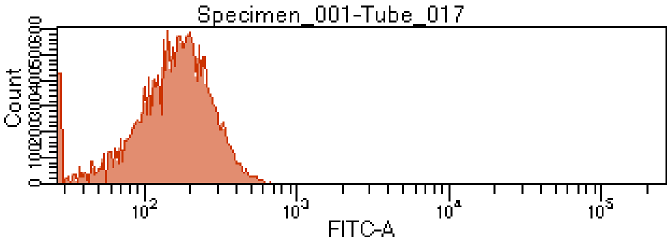

Tube Name: Tube_017
GUID: 7570150a-4bfb-4039-88b3-27f89114c00d

Population	#Events	%Parent	PE-A Mean	PE-A %CV	APC-A Mean	APC-A %CV	APC-Cy7-A Mean	APC-Cy7-A %CV
All Events	21,645	####	455	340.2	388	404.2	217	432.9
P1	20,422	94.3	437	353.5	374	418.3	210	446.2
P2	20,031	98.1	432	335.3	362	424.7	203	453.3
Q1	90	0.4	4,365	64.5	566	46.0	314	47.9
Q2	409	2.0	8,887	54.7	6,851	81.9	4,006	85.9
Q3	19,197	95.8	233	53.1	129	90.4	66	103.8
Q4	335	1.7	442	55.1	5,715	76.0	3,340	80.1
