# Supplementary material for: Salvia chinensis Benth Inhibits Triple-Negative Breast Cancer Progression by Inducing the DNA Damage Pathway
Source: Front Oncol. 2022 Aug 10;12:882784. doi: 10.3389/fonc.2022.882784 (PMC9404549; doi:10.3389/fonc.2022.882784)
Supplement: Supplementary file 18 [file DataSheet_11.zip › other raw data/figure 2a/15.HCC1187-V3.pdf]

BD FACSDiva 8.0.1

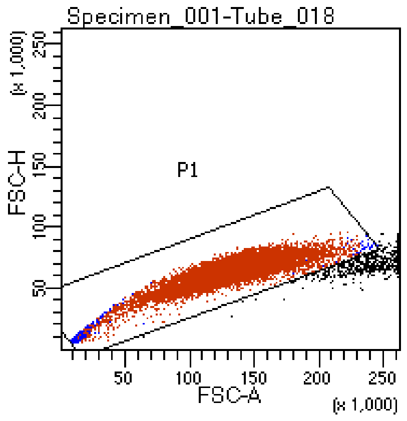

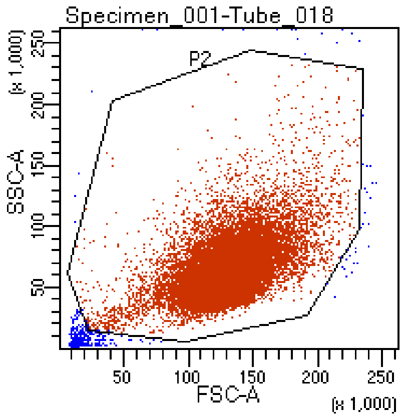

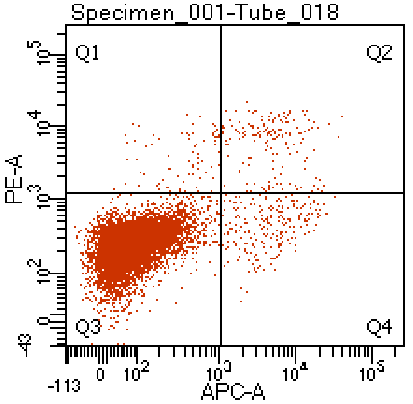

Tube: Tube_018

Population	#Events	%Parent	%Total
All Events	21,747	####	100.0
P1	20,620	94.8	94.8
P2	20,054	97.3	92.2
Q1	130	0.6	0.6
Q2	351	1.8	1.6
Q3	19,133	95.4	88.0
Q4	440	2.2	2.0

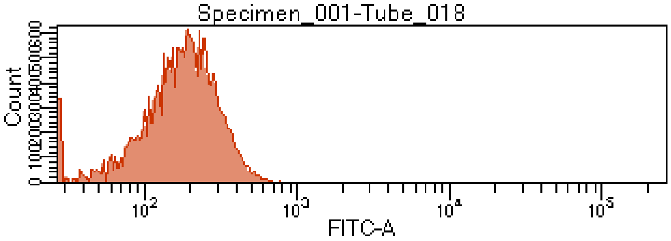

Tube Name: Tube_018

GUID: 8e937a78-0d72-4a73-9dd0-552a5e71bf2c

Population	#Events	%Parent	PE-A Mean	PE-A %CV	APC-A Mean	APC-A %CV	APC-Cy7-A Mean	APC-Cy7-A %CV
All Events	21,747	####	450	300.8	367	474.8	204	505.4
P1	20,620	94.8	428	299.0	353	469.5	196	495.8
P2	20,054	97.3	431	294.6	348	473.2	193	501.3
Q1	130	0.6	5,593	68.1	478	61.2	269	63.0
Q2	351	1.8	7,828	56.7	6,488	99.6	3,702	104.9
Q3	19,133	95.4	259	52.0	100	106.9	51	123.2
Q4	440	2.2	495	56.6	6,171	77.9	3,545	82.8
