# Supplementary material for: Salvia chinensis Benth Inhibits Triple-Negative Breast Cancer Progression by Inducing the DNA Damage Pathway
Source: Front Oncol. 2022 Aug 10;12:882784. doi: 10.3389/fonc.2022.882784 (PMC9404549; doi:10.3389/fonc.2022.882784)
Supplement: Supplementary file 18 [file DataSheet_11.zip › other raw data/figure 2a/16.HCC1187-50mg-1.pdf]

BD FACSDiva 8.0.1

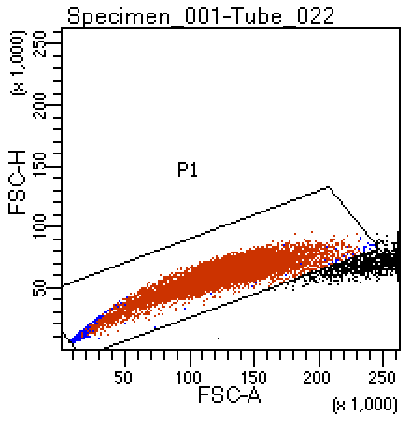

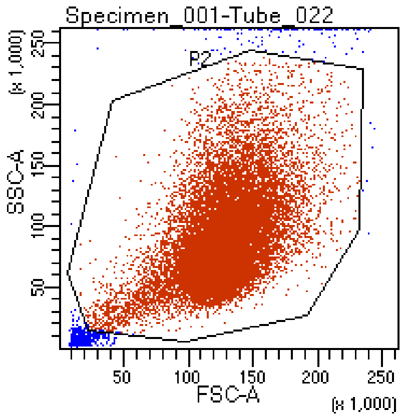

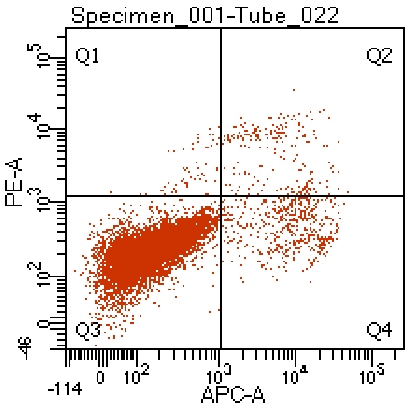

Tube: Tube_022

Population	#Events	%Parent	%Total
All Events	22,724	####	100.0
P1	20,962	92.2	92.2
P2	20,074	95.8	88.3
Q1	110	0.5	0.5
Q2	468	2.3	2.1
Q3	18,503	92.2	81.4
Q4	993	4.9	4.4

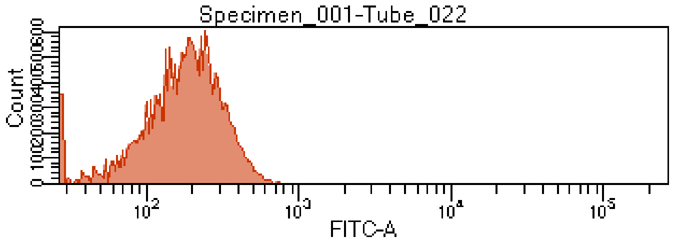

Tube Name: Tube_022

GUID: fae5b4ab-2df8-4b9c-8e63-392d5164a603

Population	#Events	%Parent	PE-A Mean	PE-A %CV	APC-A Mean	APC-A %CV	APC-Cy7-A Mean	APC-Cy7-A %CV
All Events	22,724	####	449	279.4	910	368.8	535	394.9
P1	20,962	92.2	434	292.5	927	369.8	548	394.6
P2	20,074	95.8	434	290.0	928	374.2	548	399.1
Q1	110	0.5	3,893	69.4	546	46.5	299	47.8
Q2	468	2.3	6,761	68.2	8,087	86.8	4,784	91.7
Q3	18,503	92.2	249	53.6	191	85.9	98	93.6
Q4	993	4.9	534	55.4	11,320	77.9	6,969	83.8
