# Supplementary material for: Salvia chinensis Benth Inhibits Triple-Negative Breast Cancer Progression by Inducing the DNA Damage Pathway
Source: Front Oncol. 2022 Aug 10;12:882784. doi: 10.3389/fonc.2022.882784 (PMC9404549; doi:10.3389/fonc.2022.882784)
Supplement: Supplementary file 18 [file DataSheet_11.zip › other raw data/figure 2a/17.HCC1187-50mg-2.pdf]

BD FACSDiva 8.0.1

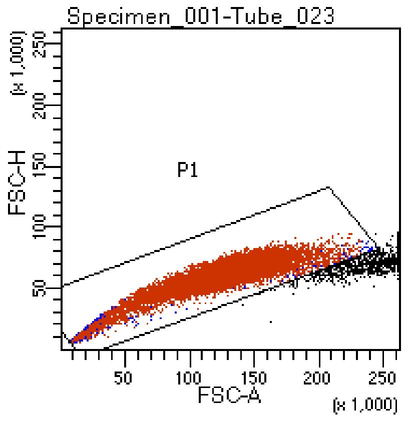

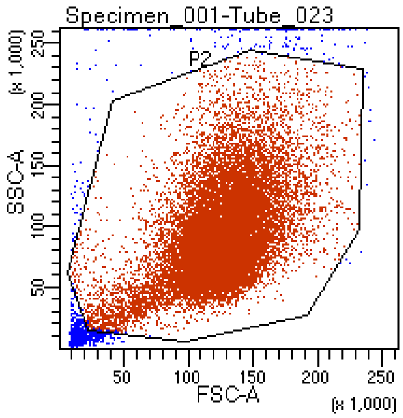

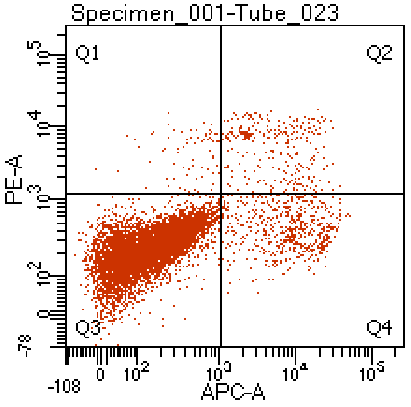

Tube: Tube_023

Population	#Events	%Parent	%Total
All Events	23,024	####	100.0
P1	21,466	93.2	93.2
P2	20,118	93.7	87.4
Q1	146	0.7	0.6
Q2	603	3.0	2.6
Q3	18,187	90.4	79.0
Q4	1,182	5.9	5.1

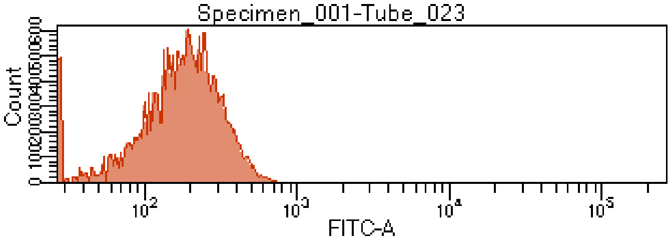

Tube Name: Tube_023
GUID: f32c747d-888e-4985-997d-67d4a55b25a5

Population	#Events	%Parent	PE-A Mean	PE-A %CV	APC-A Mean	APC-A %CV	APC-Cy7-A Mean	APC-Cy7-A %CV
All Events	23,024	####	511	292.5	1,020	363.4	608	386.6
P1	21,466	93.2	492	286.0	1,035	359.2	619	381.9
P2	20,118	93.7	503	281.5	1,051	361.2	630	383.5
Q1	146	0.7	5,227	58.6	527	55.4	293	58.5
Q2	603	3.0	6,757	62.2	7,649	88.1	4,462	93.7
Q3	18,187	90.4	261	56.4	177	95.3	91	103.5
Q4	1,182	5.9	462	59.3	11,201	82.2	7,023	87.3
