# Supplementary material for: Salvia chinensis Benth Inhibits Triple-Negative Breast Cancer Progression by Inducing the DNA Damage Pathway
Source: Front Oncol. 2022 Aug 10;12:882784. doi: 10.3389/fonc.2022.882784 (PMC9404549; doi:10.3389/fonc.2022.882784)
Supplement: Supplementary file 18 [file DataSheet_11.zip › other raw data/figure 2a/18.HCC1187-50mg-3.pdf]

BD FACSDiva 8.0.1

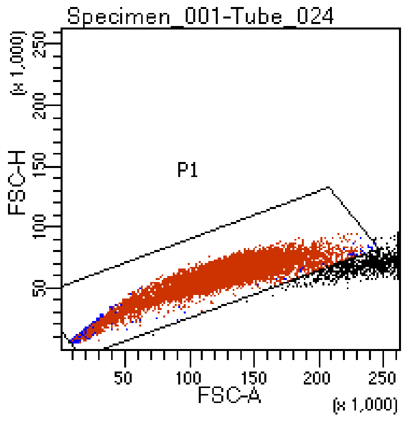

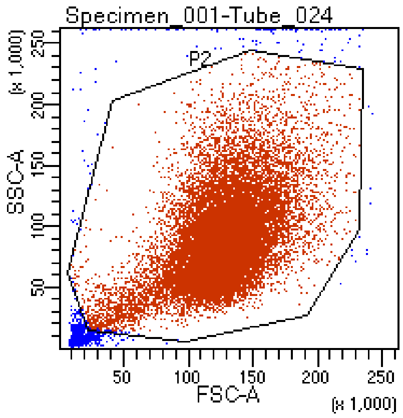

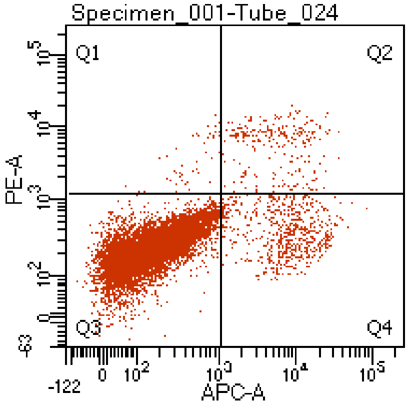

Tube: Tube_024

Population	#Events	%Parent	%Total
All Events	23,106	####	100.0
P1	21,375	92.5	92.5
P2	20,133	94.2	87.1
Q1	88	0.4	0.4
Q2	528	2.6	2.3
Q3	18,298	90.9	79.2
Q4	1,219	6.1	5.3

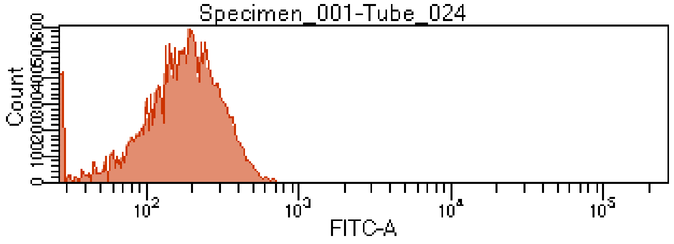

Tube Name: Tube_024
GUID: 2f628358-2880-40a2-b327-e16420e194fd

Population	#Events	%Parent	PE-A Mean	PE-A %CV	APC-A Mean	APC-A %CV	APC-Cy7-A Mean	APC-Cy7-A %CV
All Events	23,106	####	480	269.7	1,027	345.3	607	366.8
P1	21,375	92.5	461	278.6	1,023	344.0	607	364.6
P2	20,133	94.2	469	273.1	1,021	347.5	606	367.5
Q1	88	0.4	4,825	60.3	567	48.3	314	51.1
Q2	528	2.6	6,833	57.0	8,032	88.8	4,656	91.8
Q3	18,298	90.9	266	54.2	215	87.7	111	93.5
Q4	1,219	6.1	448	63.5	10,113	83.8	6,317	87.6
