# Supplementary material for: Salvia chinensis Benth Inhibits Triple-Negative Breast Cancer Progression by Inducing the DNA Damage Pathway
Source: Front Oncol. 2022 Aug 10;12:882784. doi: 10.3389/fonc.2022.882784 (PMC9404549; doi:10.3389/fonc.2022.882784)
Supplement: Supplementary file 18 [file DataSheet_11.zip › other raw data/figure 2a/19.HCC1187-100mg-1.pdf]

BD FACSDiva 8.0.1

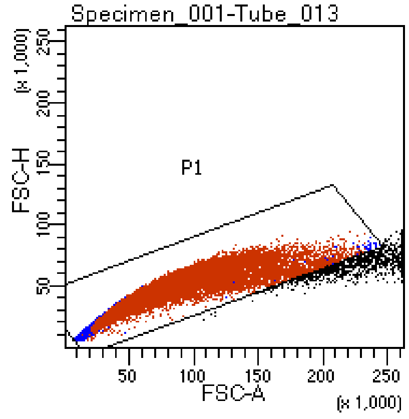

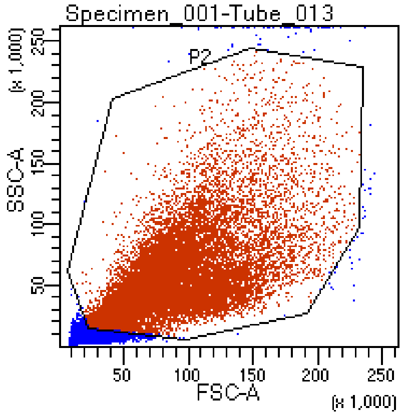

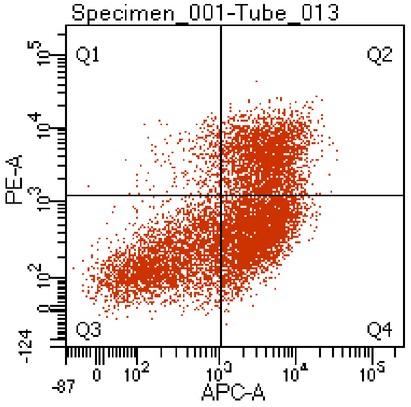

Tube: Tube_013

Population	#Events	%Parent	%Total
All Events	29,760	####	100.0
P1	27,256	91.6	91.6
P2	20,712	76.0	69.6
Q1	601	2.9	2.0
Q2	5,571	26.9	18.7
Q3	6,746	32.6	22.7
Q4	7,794	37.6	26.2

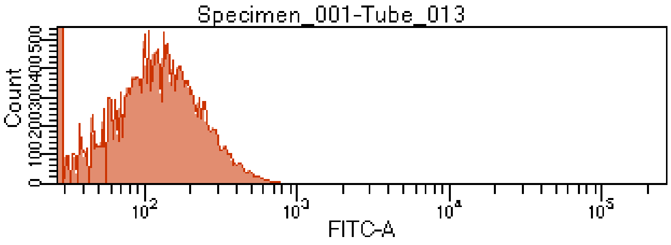

Tube Name: Tube_013
GUID: 981d2476-93ef-4b91-89d7-ba1175423aa8

Population	#Events	%Parent	PE-A Mean	PE-A %CV	APC-A Mean	APC-A %CV	APC-Cy7-A Mean	APC-Cy7-A %CV
All Events	29,760	####	1,437	196.0	2,304	129.0	1,426	135.3
P1	27,256	91.6	1,439	185.4	2,368	121.3	1,469	126.8
P2	20,712	76.0	1,755	165.1	2,884	105.1	1,793	110.2
Q1	601	2.9	5,070	66.7	640	42.6	370	45.1
Q2	5,571	26.9	5,044	70.6	5,083	66.9	3,218	70.6
Q3	6,746	32.6	226	88.3	347	83.3	187	87.2
Q4	7,794	37.6	472	59.9	3,681	66.7	2,274	70.7
