# Supplementary material for: Salvia chinensis Benth Inhibits Triple-Negative Breast Cancer Progression by Inducing the DNA Damage Pathway
Source: Front Oncol. 2022 Aug 10;12:882784. doi: 10.3389/fonc.2022.882784 (PMC9404549; doi:10.3389/fonc.2022.882784)
Supplement: Supplementary file 18 [file DataSheet_11.zip › other raw data/figure 2a/2.MDAMB231-V2.pdf]

BD FACSDiva 8.0.1

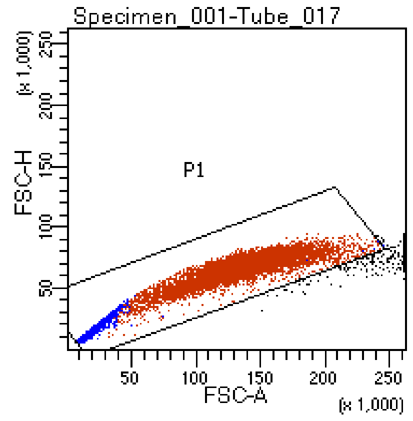

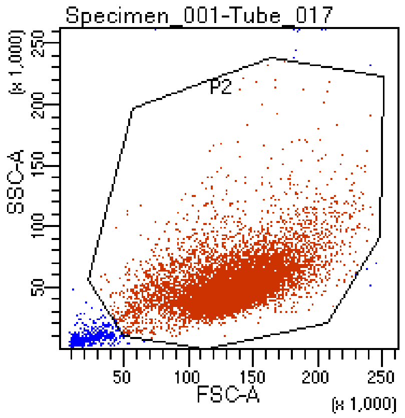

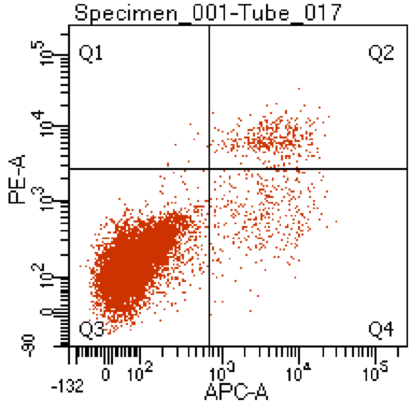

Tube: Tube_017

Population	#Events	%Parent	%Total
All Events	11,230	####	100.0
P1	10,742	95.7	95.7
P2	10,026	93.3	89.3
Q1	8	0.1	0.1
Q2	407	4.1	3.6
Q3	9,267	92.4	82.5
Q4	344	3.4	3.1

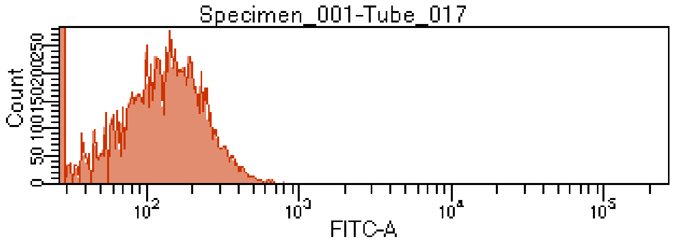

Tube Name: Tube_017
GUID: ffc7bea6-b8e6-4b0d-9c4b-02e446aae27c

Population	#Events	%Parent	PE-A Mean	PE-A %CV	APC-A Mean	APC-A %CV	APC-Cy7-A Mean	APC-Cy7-A %CV
All Events	11,230	####	526	363.5	512	387.7	298	406.9
P1	10,742	95.7	492	325.9	493	374.6	286	392.8
P2	10,026	93.3	505	321.4	474	381.1	274	398.7
Q1	8	0.1	5,870	48.2	390	38.1	221	42.0
Q2	407	4.1	7,505	45.9	5,582	70.7	3,330	73.0
Q3	9,267	92.4	181	80.2	81	105.3	40	129.9
Q4	344	3.4	825	75.2	5,015	91.0	2,962	95.9
