# Supplementary material for: Salvia chinensis Benth Inhibits Triple-Negative Breast Cancer Progression by Inducing the DNA Damage Pathway
Source: Front Oncol. 2022 Aug 10;12:882784. doi: 10.3389/fonc.2022.882784 (PMC9404549; doi:10.3389/fonc.2022.882784)
Supplement: Supplementary file 18 [file DataSheet_11.zip › other raw data/figure 2a/20.HCC1187-100mg-2.pdf]

BD FACSDiva 8.0.1

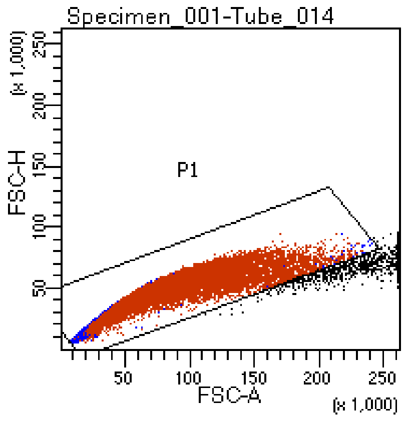

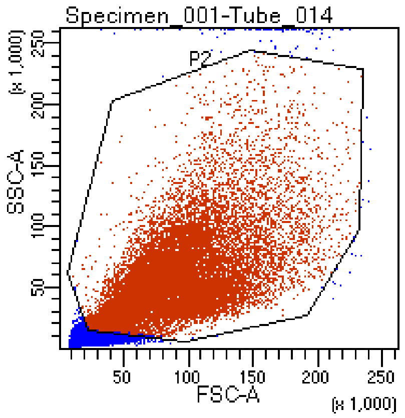

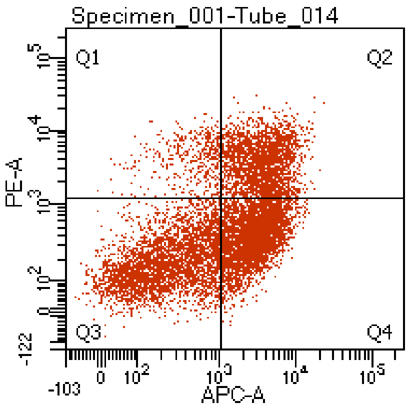

Tube: Tube_014

Population	#Events	%Parent	%Total
All Events	30,670	####	100.0
P1	28,375	92.5	92.5
P2	20,785	73.3	67.8
Q1	874	4.2	2.8
Q2	5,066	24.4	16.5
Q3	7,419	35.7	24.2
Q4	7,426	35.7	24.2

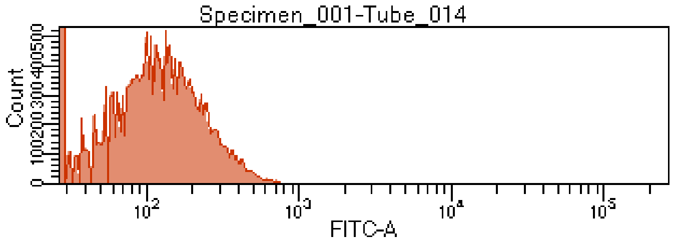

Tube Name: Tube_014
GUID: 7f99298d-28d7-49fb-bd65-3ba577806221

Population	#Events	%Parent	PE-A Mean	PE-A %CV	APC-A Mean	APC-A %CV	APC-Cy7-A Mean	APC-Cy7-A %CV
All Events	30,670	####	1,279	195.2	1,836	125.5	1,140	131.0
P1	28,375	92.5	1,292	188.7	1,891	119.7	1,175	125.1
P2	20,785	73.3	1,606	166.3	2,362	102.1	1,472	106.8
Q1	874	4.2	5,228	62.6	591	48.8	350	51.7
Q2	5,066	24.4	4,657	71.3	4,351	60.0	2,763	63.4
Q3	7,419	35.7	231	88.2	339	87.2	186	91.5
Q4	7,426	35.7	473	60.4	3,235	58.6	2,010	62.2
