# Supplementary material for: Salvia chinensis Benth Inhibits Triple-Negative Breast Cancer Progression by Inducing the DNA Damage Pathway
Source: Front Oncol. 2022 Aug 10;12:882784. doi: 10.3389/fonc.2022.882784 (PMC9404549; doi:10.3389/fonc.2022.882784)
Supplement: Supplementary file 18 [file DataSheet_11.zip › other raw data/figure 2a/21.HCC1187-100mg-3.pdf]

# BD FACSDiva 8.0.1

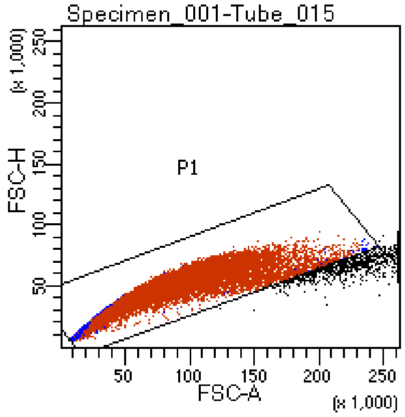


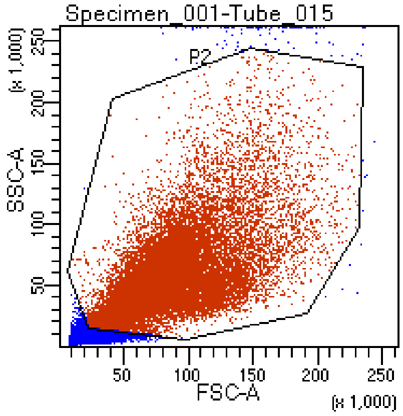


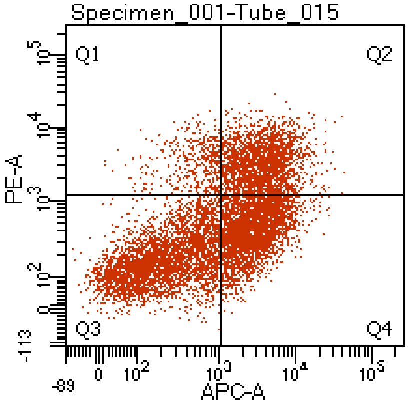


Tube: Tube_015

| Population | #Events | %Parent | %Total |
|---|---|---|---|
| All Events | 29,329 | #### | 100.0 |
| P1 | 27,125 | 92.5 | 92.5 |
| P2 | 20,680 | 76.2 | 70.5 |
| Q1 | 984 | 4.8 | 3.4 |
| Q2 | 4,876 | 23.6 | 16.6 |
| Q3 | 8,174 | 39.5 | 27.9 |
| Q4 | 6,646 | 32.1 | 22.7 |

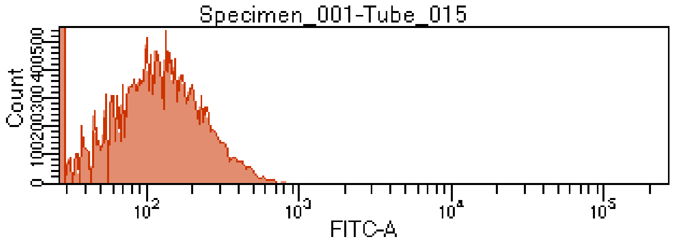


Tube Name: Tube_015
GUID: 6529faee-32df-4fa3-9d93-c4855853d8f2

| Population | #Events | %Parent | PE-A Mean | PE-A %CV | APC-A Mean | APC-A %CV | APC-Cy7-A Mean | APC-Cy7-A %CV |
|---|---|---|---|---|---|---|---|---|
| All Events | 29,329 | #### | 1,146 | 187.2 | 1,842 | 152.1 | 1,149 | 156.8 |
| P1 | 27,125 | 92.5 | 1,147 | 178.1 | 1,874 | 141.9 | 1,170 | 145.9 |
| P2 | 20,680 | 76.2 | 1,375 | 157.7 | 2,270 | 125.5 | 1,420 | 129.2 |
| Q1 | 984 | 4.8 | 3,799 | 61.6 | 613 | 46.4 | 365 | 48.8 |
| Q2 | 4,876 | 23.6 | 4,007 | 67.4 | 4,414 | 78.9 | 2,813 | 80.6 |
| Q3 | 8,174 | 39.5 | 226 | 89.7 | 319 | 89.6 | 177 | 94.6 |
| Q4 | 6,646 | 32.1 | 498 | 58.0 | 3,343 | 77.1 | 2,084 | 79.4 |
