# Supplementary material for: Salvia chinensis Benth Inhibits Triple-Negative Breast Cancer Progression by Inducing the DNA Damage Pathway
Source: Front Oncol. 2022 Aug 10;12:882784. doi: 10.3389/fonc.2022.882784 (PMC9404549; doi:10.3389/fonc.2022.882784)
Supplement: Supplementary file 18 [file DataSheet_11.zip › other raw data/figure 2a/22.HCC1187-200mg-1.pdf]

BD FACSDiva 8.0.1

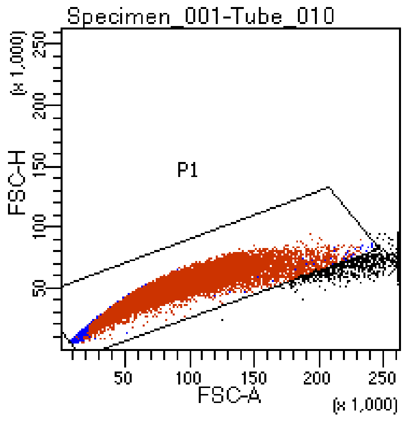

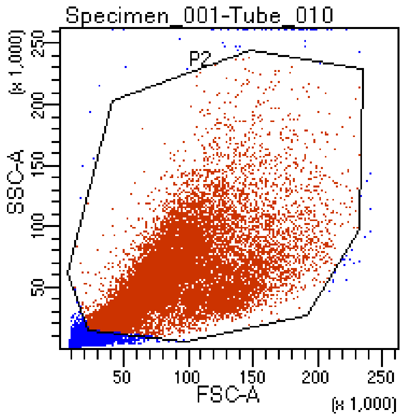

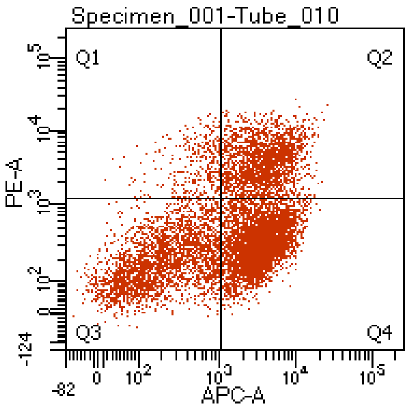

Tube: Tube_010

Population	#Events	%Parent	%Total
All Events	29,906	####	100.0
P1	27,473	91.9	91.9
P2	20,829	75.8	69.6
Q1	696	3.3	2.3
Q2	4,287	20.6	14.3
Q3	5,932	28.5	19.8
Q4	9,914	47.6	33.2

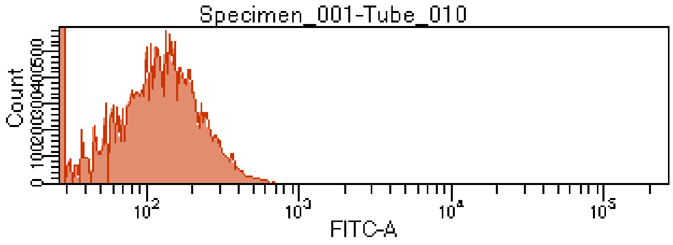

Tube Name: Tube_010
GUID: 8fe0db9e-4669-4e68-9864-c2f7c521cdb9

Population	#Events	%Parent	PE-A Mean	PE-A %CV	APC-A Mean	APC-A %CV	APC-Cy7-A Mean	APC-Cy7-A %CV
All Events	29,906	####	1,096	213.0	2,188	118.1	1,387	122.3
P1	27,473	91.9	1,118	204.8	2,317	111.3	1,470	115.4
P2	20,829	75.8	1,372	181.0	2,840	94.6	1,807	98.1
Q1	696	3.3	4,579	68.6	620	45.5	368	47.8
Q2	4,287	20.6	4,719	71.1	4,660	67.0	2,994	70.8
Q3	5,932	28.5	246	89.4	304	93.8	169	99.3
Q4	9,914	47.6	374	63.1	3,727	56.9	2,374	58.9
