# Supplementary material for: Salvia chinensis Benth Inhibits Triple-Negative Breast Cancer Progression by Inducing the DNA Damage Pathway
Source: Front Oncol. 2022 Aug 10;12:882784. doi: 10.3389/fonc.2022.882784 (PMC9404549; doi:10.3389/fonc.2022.882784)
Supplement: Supplementary file 18 [file DataSheet_11.zip › other raw data/figure 2a/23.HCC1187-200mg-2.pdf]

BD FACSDiva 8.0.1

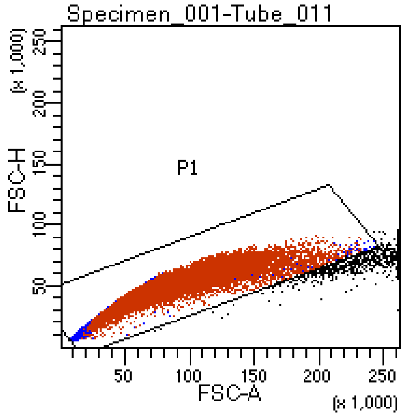

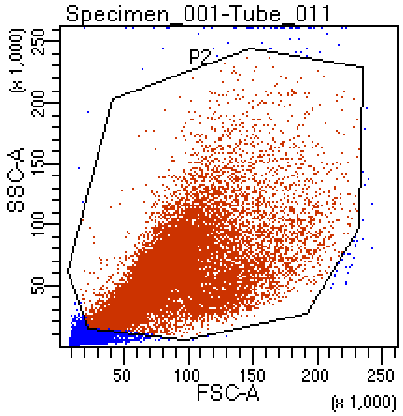

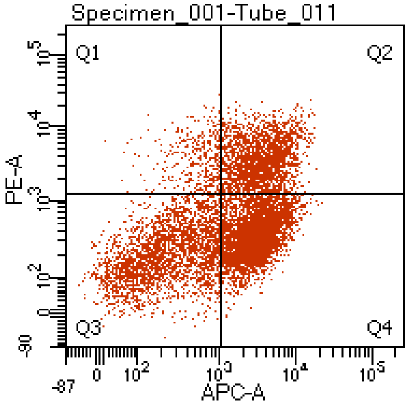

Tube: Tube_011

Population	#Events	%Parent	%Total
All Events	29,463	####	100.0
P1	27,135	92.1	92.1
P2	20,715	76.3	70.3
Q1	820	4.0	2.8
Q2	4,584	22.1	15.6
Q3	5,755	27.8	19.5
Q4	9,556	46.1	32.4

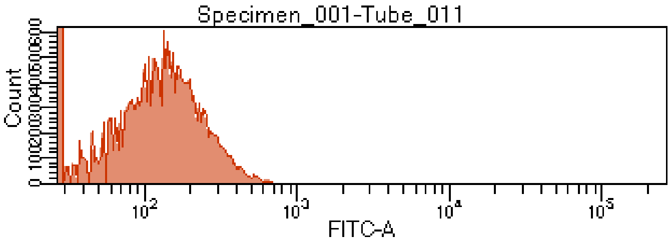

Tube Name: Tube_011
GUID: cb05097f-94ad-460e-b28f-e3892b568daa

Population	#Events	%Parent	PE-A Mean	PE-A %CV	APC-A Mean	APC-A %CV	APC-Cy7-A Mean	APC-Cy7-A %CV
All Events	29,463	####	1,163	202.9	1,997	116.3	1,252	120.4
P1	27,135	92.1	1,182	195.3	2,093	109.7	1,313	113.5
P2	20,715	76.3	1,457	173.2	2,563	92.3	1,612	95.7
Q1	820	4.0	4,617	73.1	625	43.8	366	45.9
Q2	4,584	22.1	4,590	71.7	4,196	64.5	2,661	68.2
Q3	5,755	27.8	259	87.2	343	86.7	191	92.3
Q4	9,556	46.1	405	60.5	3,283	56.8	2,071	58.5
