# Supplementary material for: Salvia chinensis Benth Inhibits Triple-Negative Breast Cancer Progression by Inducing the DNA Damage Pathway
Source: Front Oncol. 2022 Aug 10;12:882784. doi: 10.3389/fonc.2022.882784 (PMC9404549; doi:10.3389/fonc.2022.882784)
Supplement: Supplementary file 18 [file DataSheet_11.zip › other raw data/figure 2a/24.HCC187-200mg-3.pdf]

BD FACSDiva 8.0.1

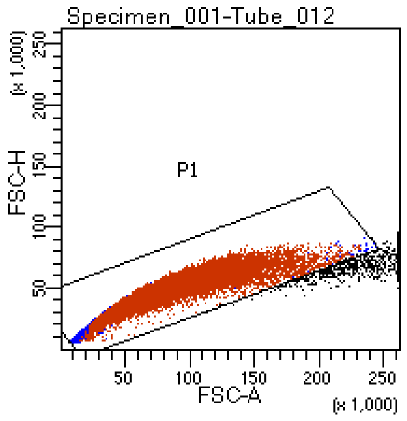

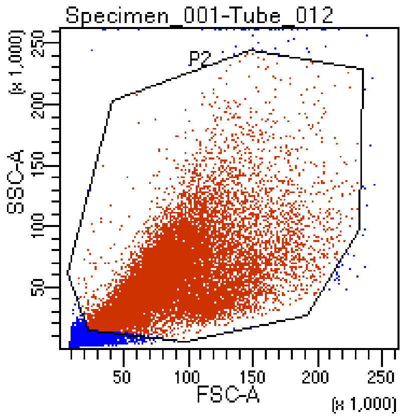

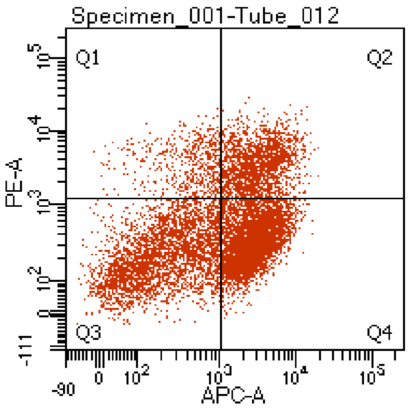

Tube: Tube_012

Population	#Events	%Parent	%Total
All Events	30,458	####	100.0
P1	28,112	92.3	92.3
P2	20,880	74.3	68.6
Q1	1,192	5.7	3.9
Q2	3,777	18.1	12.4
Q3	6,611	31.7	21.7
Q4	9,300	44.5	30.5

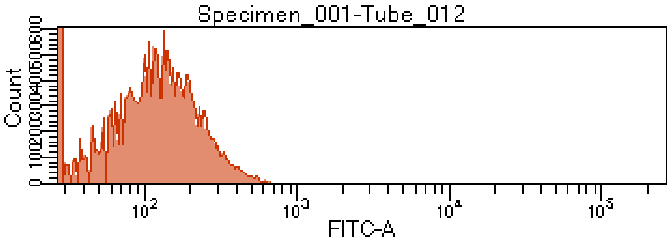

Tube Name: Tube_012
GUID: 8b29e6ce-f2fa-477a-8092-5dbc00e4ff2b

Population	#Events	%Parent	PE-A Mean	PE-A %CV	APC-A Mean	APC-A %CV	APC-Cy7-A Mean	APC-Cy7-A %CV
All Events	30,458	####	1,011	206.9	1,682	127.8	1,038	132.7
P1	28,112	92.3	1,024	196.7	1,755	117.1	1,084	120.9
P2	20,880	74.3	1,271	174.0	2,187	98.4	1,355	101.7
Q1	1,192	5.7	4,272	65.8	539	55.5	316	57.2
Q2	3,777	18.1	4,242	70.5	3,880	66.3	2,424	69.5
Q3	6,611	31.7	279	88.3	305	93.3	170	98.5
Q4	9,300	44.5	384	62.8	3,049	55.6	1,896	57.5
