# Supplementary material for: Salvia chinensis Benth Inhibits Triple-Negative Breast Cancer Progression by Inducing the DNA Damage Pathway
Source: Front Oncol. 2022 Aug 10;12:882784. doi: 10.3389/fonc.2022.882784 (PMC9404549; doi:10.3389/fonc.2022.882784)
Supplement: Supplementary file 18 [file DataSheet_11.zip › other raw data/figure 2a/25.4T1-V1.pdf]

BD FACSDiva 8.0.1

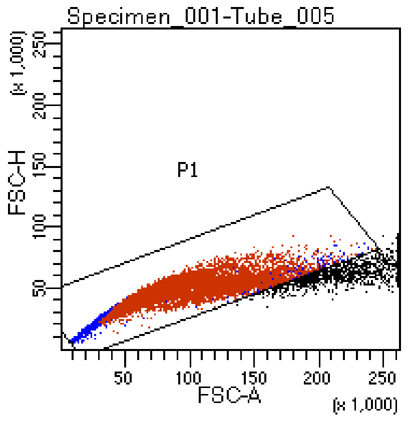

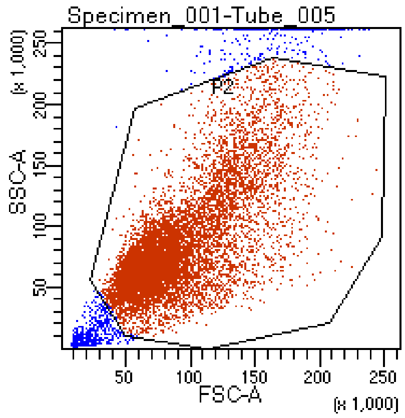

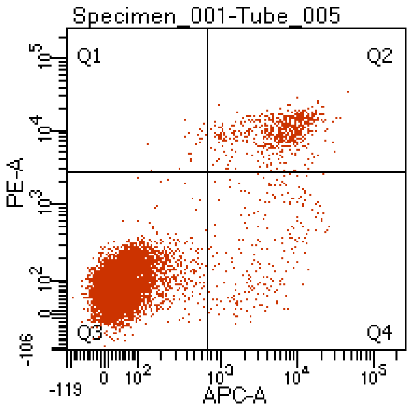

Tube: Tube_005

Population	#Events	%Parent	%Total
All Events	12,365	####	100.0
P1	10,737	86.8	86.8
P2	9,870	91.9	79.8
Q1	26	0.3	0.2
Q2	665	6.7	5.4
Q3	8,945	90.6	72.3
Q4	234	2.4	1.9

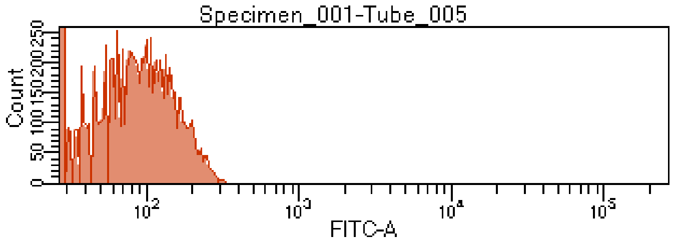

Tube Name: Tube_005

GUID: 9b1f0927-a3bc-45b6-b9a3-f22a6c5bf6e6

Population	#Events	%Parent	PE-A Mean	PE-A %CV	APC-A Mean	APC-A %CV	APC-Cy7-A Mean	APC-Cy7-A %CV
All Events	12,365	####	868	344.0	695	353.4	384	371.1
P1	10,737	86.8	829	348.7	712	358.0	396	374.7
P2	9,870	91.9	827	347.4	680	374.8	376	390.9
Q1	26	0.3	7,046	44.8	464	34.7	261	34.3
Q2	665	6.7	10,581	39.9	7,553	68.8	4,224	73.3
Q3	8,945	90.6	91	85.8	47	139.1	21	196.4
Q4	234	2.4	519	121.5	5,393	94.1	3,053	103.6
