# Supplementary material for: Salvia chinensis Benth Inhibits Triple-Negative Breast Cancer Progression by Inducing the DNA Damage Pathway
Source: Front Oncol. 2022 Aug 10;12:882784. doi: 10.3389/fonc.2022.882784 (PMC9404549; doi:10.3389/fonc.2022.882784)
Supplement: Supplementary file 18 [file DataSheet_11.zip › other raw data/figure 2a/26.4T1-V2.pdf]

BD FACSDiva 8.0.1

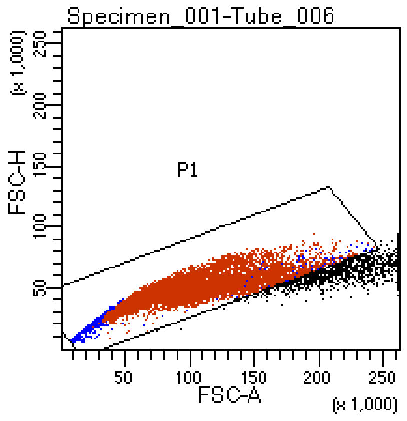

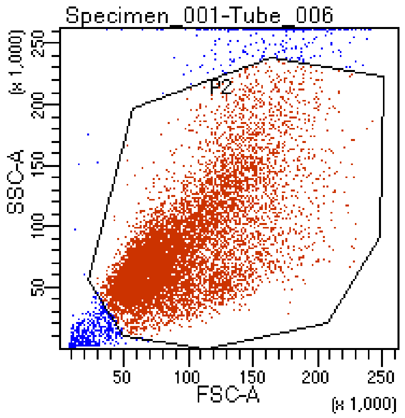

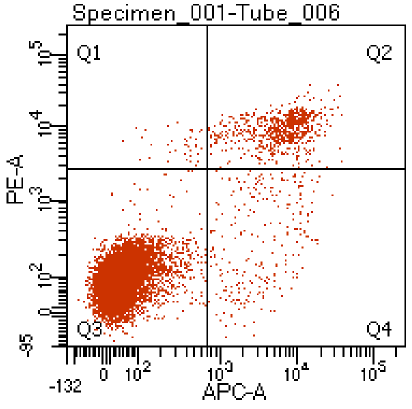

Tube: Tube_006

Population	#Events	%Parent	%Total
All Events	12,479	####	100.0
P1	10,697	85.7	85.7
P2	9,900	92.5	79.3
Q1	38	0.4	0.3
Q2	738	7.5	5.9
Q3	8,874	89.6	71.1
Q4	250	2.5	2.0

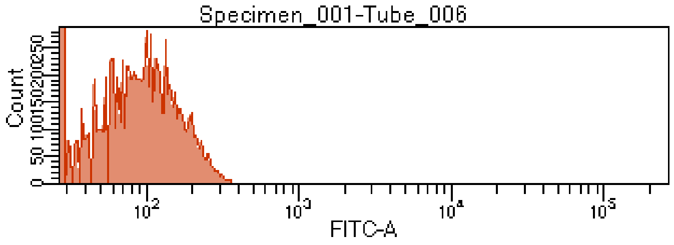

Tube Name: Tube_006
GUID: 10417927-773d-4f6d-86ab-c1d8847db804

Population	#Events	%Parent	PE-A Mean	PE-A %CV	APC-A Mean	APC-A %CV	APC-Cy7-A Mean	APC-Cy7-A %CV
All Events	12,479	####	916	335.8	781	353.7	435	369.5
P1	10,697	85.7	868	331.3	790	347.5	443	361.1
P2	9,900	92.5	888	331.6	760	363.7	425	378.6
Q1	38	0.4	6,089	38.8	351	44.6	208	42.1
Q2	738	7.5	10,183	44.5	7,927	71.7	4,482	76.8
Q3	8,874	89.6	100	99.1	50	137.1	23	179.5
Q4	250	2.5	620	112.1	4,859	93.1	2,725	102.3
