# Supplementary material for: Salvia chinensis Benth Inhibits Triple-Negative Breast Cancer Progression by Inducing the DNA Damage Pathway
Source: Front Oncol. 2022 Aug 10;12:882784. doi: 10.3389/fonc.2022.882784 (PMC9404549; doi:10.3389/fonc.2022.882784)
Supplement: Supplementary file 18 [file DataSheet_11.zip › other raw data/figure 2a/27.4T1-V3.pdf]

BD FACSDiva 8.0.1

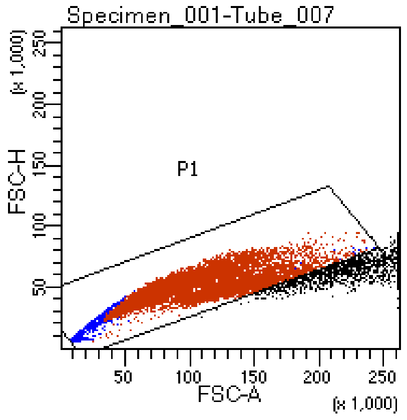

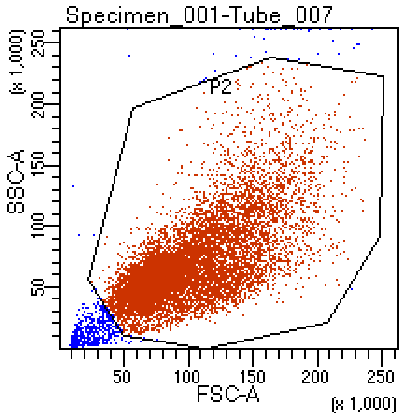

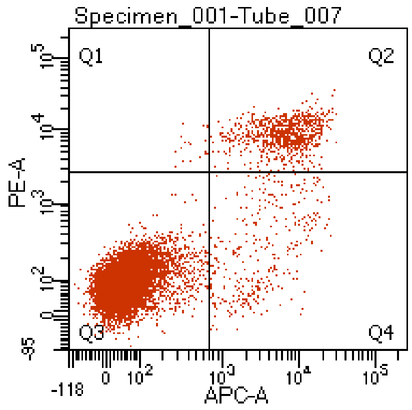

Tube: Tube_007

Population	#Events	%Parent	%Total
All Events	12,602	####	100.0
P1	10,602	84.1	84.1
P2	9,902	93.4	78.6
Q1	18	0.2	0.1
Q2	888	9.0	7.0
Q3	8,668	87.5	68.8
Q4	328	3.3	2.6

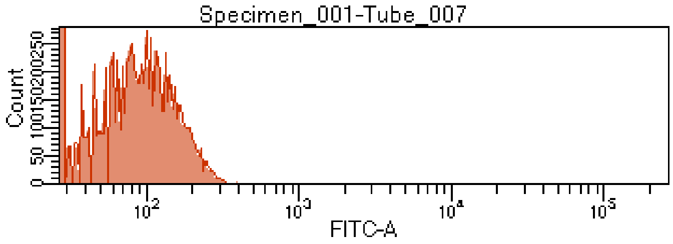

Tube Name: Tube_007
GUID: d01722fe-2650-47a4-9bd9-8dd874f6f28e

Population	#Events	%Parent	PE-A Mean	PE-A %CV	APC-A Mean	APC-A %CV	APC-Cy7-A Mean	APC-Cy7-A %CV
All Events	12,602	####	981	321.6	967	323.9	557	446.4
P1	10,602	84.1	961	309.0	1,012	306.6	572	317.3
P2	9,902	93.4	987	307.0	972	323.7	548	335.7
Q1	18	0.2	6,819	43.6	458	32.0	246	30.4
Q2	888	9.0	9,728	42.5	8,367	65.2	4,763	68.3
Q3	8,668	87.5	97	73.1	58	129.5	26	173.0
Q4	328	3.3	512	123.7	5,158	107.7	2,933	119.8
