# Supplementary material for: Salvia chinensis Benth Inhibits Triple-Negative Breast Cancer Progression by Inducing the DNA Damage Pathway
Source: Front Oncol. 2022 Aug 10;12:882784. doi: 10.3389/fonc.2022.882784 (PMC9404549; doi:10.3389/fonc.2022.882784)
Supplement: Supplementary file 18 [file DataSheet_11.zip › other raw data/figure 2a/28.4T1-50mg-1.pdf]

BD FACSDiva 8.0.1

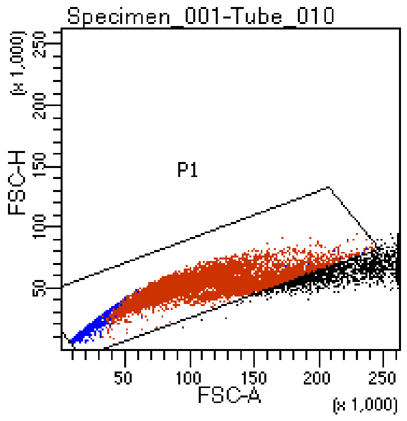

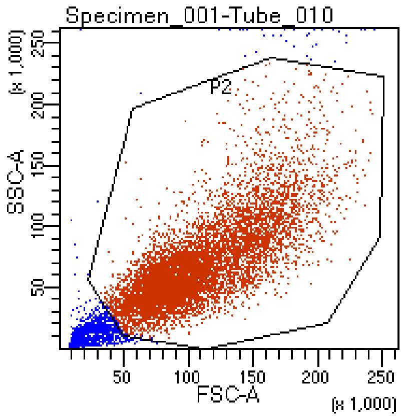

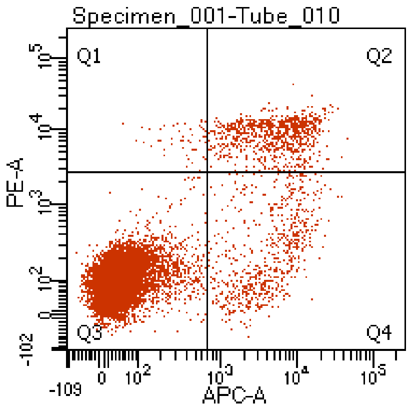

Tube: Tube_010

Population	#Events	%Parent	%Total
All Events	13,148	####	100.0
P1	11,140	84.7	84.7
P2	9,901	88.9	75.3
Q1	52	0.5	0.4
Q2	832	8.4	6.3
Q3	8,343	84.3	63.5
Q4	674	6.8	5.1

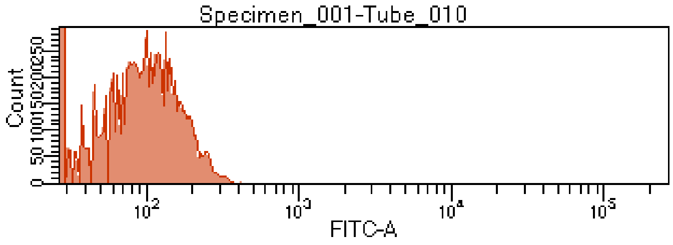

Tube Name: Tube_010
GUID: 6c6217a1-b292-4497-8337-4bc14cfe224d

Population	#Events	%Parent	PE-A Mean	PE-A %CV	APC-A Mean	APC-A %CV	APC-Cy7-A Mean	APC-Cy7-A %CV
All Events	13,148	####	993	333.0	1,080	284.9	623	294.9
P1	11,140	84.7	911	314.9	1,134	273.1	657	282.2
P2	9,901	88.9	969	302.7	1,025	304.6	594	315.1
Q1	52	0.5	7,947	38.6	398	46.5	210	52.6
Q2	832	8.4	9,569	43.0	7,145	80.6	4,178	84.7
Q3	8,343	84.3	110	90.9	49	156.5	24	197.6
Q4	674	6.8	449	131.1	5,602	86.2	3,247	92.3
