# Supplementary material for: Salvia chinensis Benth Inhibits Triple-Negative Breast Cancer Progression by Inducing the DNA Damage Pathway
Source: Front Oncol. 2022 Aug 10;12:882784. doi: 10.3389/fonc.2022.882784 (PMC9404549; doi:10.3389/fonc.2022.882784)
Supplement: Supplementary file 18 [file DataSheet_11.zip › other raw data/figure 2a/29.4T1-50mg-2.pdf]

BD FACSDiva 8.0.1

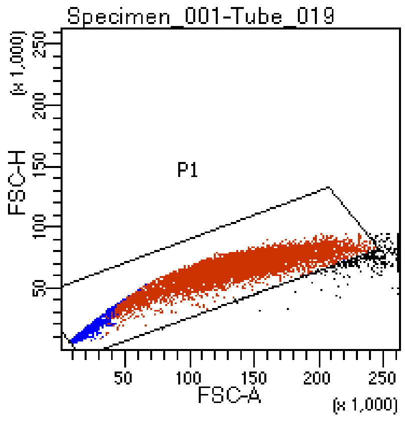

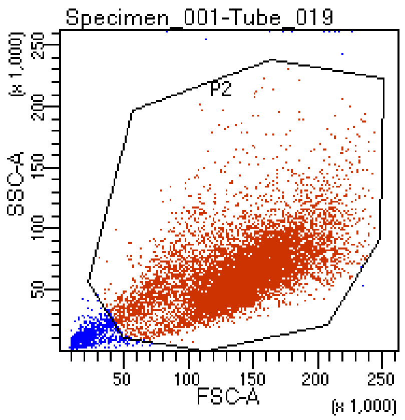

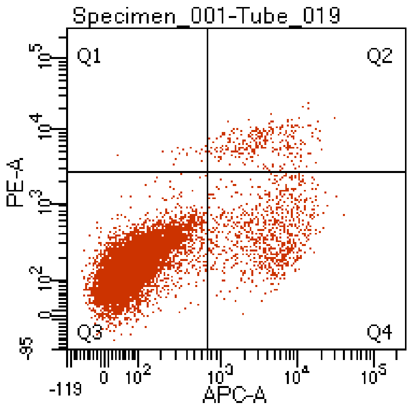

Tube: Tube_019

Population	#Events	%Parent	%Total
All Events	11,798	####	100.0
P1	11,027	93.5	93.5
P2	10,005	90.7	84.8
Q1	19	0.2	0.2
Q2	298	3.0	2.5
Q3	8,855	88.5	75.1
Q4	833	8.3	7.1

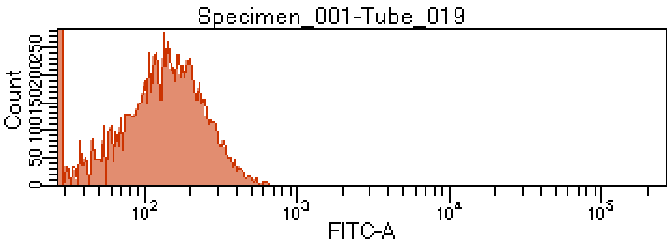

Tube Name: Tube_019
GUID: bdadd843-5b71-4b02-b577-a30b002a2bc4

Population	#Events	%Parent	PE-A Mean	PE-A %CV	APC-A Mean	APC-A %CV	APC-Cy7-A Mean	APC-Cy7-A %CV
All Events	11,798	####	468	363.8	752	319.3	441	332.0
P1	11,027	93.5	426	306.1	721	302.9	423	315.1
P2	10,005	90.7	443	303.2	686	317.9	402	331.3
Q1	19	0.2	4,916	14.4	418	42.3	252	43.9
Q2	298	3.0	7,255	42.8	4,908	85.3	2,981	88.5
Q3	8,855	88.5	191	74.3	99	101.6	50	121.3
Q4	833	8.3	588	88.3	5,421	78.8	3,224	82.5
