# Supplementary material for: Salvia chinensis Benth Inhibits Triple-Negative Breast Cancer Progression by Inducing the DNA Damage Pathway
Source: Front Oncol. 2022 Aug 10;12:882784. doi: 10.3389/fonc.2022.882784 (PMC9404549; doi:10.3389/fonc.2022.882784)
Supplement: Supplementary file 18 [file DataSheet_11.zip › other raw data/figure 2a/3.MDAMB231-V3.pdf]

BD FACSDiva 8.0.1

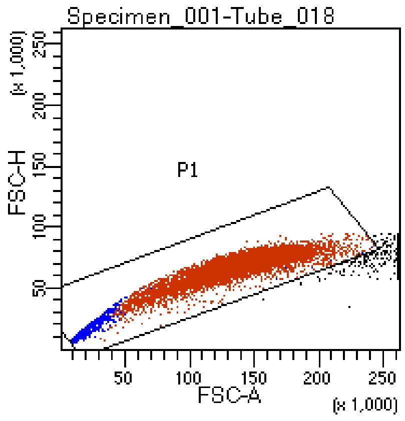

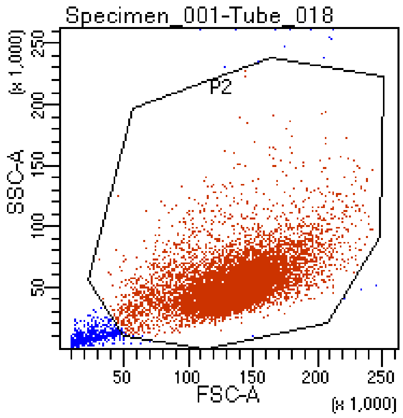

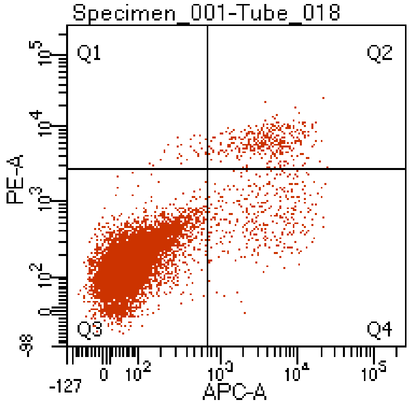

Tube: Tube_018

Population	#Events	%Parent	%Total
All Events	11,446	####	100.0
P1	10,894	95.2	95.2
P2	10,035	92.1	87.7
Q1	29	0.3	0.3
Q2	378	3.8	3.3
Q3	9,263	92.3	80.9
Q4	365	3.6	3.2

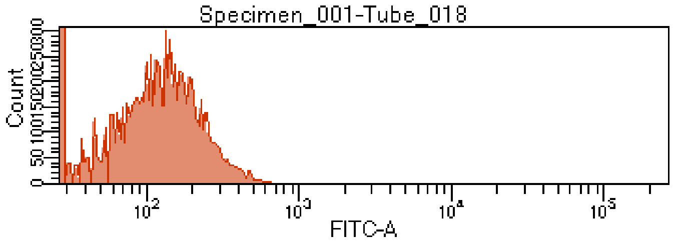

Tube Name: Tube_018
GUID: 7034c6b3-61b6-4ac8-a14e-b6bf2c8f6212

Population	#Events	%Parent	PE-A Mean	PE-A %CV	APC-A Mean	APC-A %CV	APC-Cy7-A Mean	APC-Cy7-A %CV
All Events	11,446	####	468	324.6	477	393.1	278	408.4
P1	10,894	95.2	440	311.7	466	373.8	271	390.1
P2	10,035	92.1	455	306.5	444	382.9	258	399.3
Q1	29	0.3	4,888	29.4	399	41.5	234	45.0
Q2	378	3.8	6,741	41.2	5,025	75.9	2,981	77.7
Q3	9,263	92.3	172	79.4	78	117.7	39	141.8
Q4	365	3.6	774	72.3	4,991	86.4	2,985	90.1
