# Supplementary material for: Salvia chinensis Benth Inhibits Triple-Negative Breast Cancer Progression by Inducing the DNA Damage Pathway
Source: Front Oncol. 2022 Aug 10;12:882784. doi: 10.3389/fonc.2022.882784 (PMC9404549; doi:10.3389/fonc.2022.882784)
Supplement: Supplementary file 18 [file DataSheet_11.zip › other raw data/figure 2a/30.4T1-50mg-3.pdf]

BD FACSDiva 8.0.1

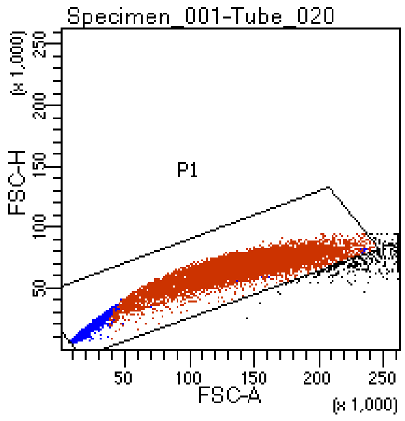

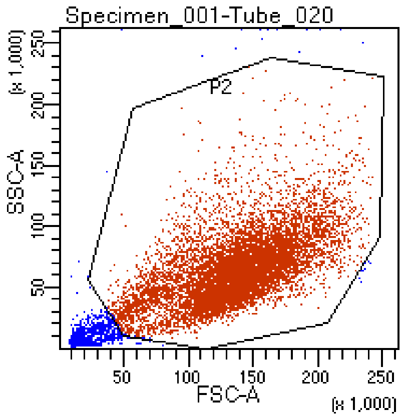

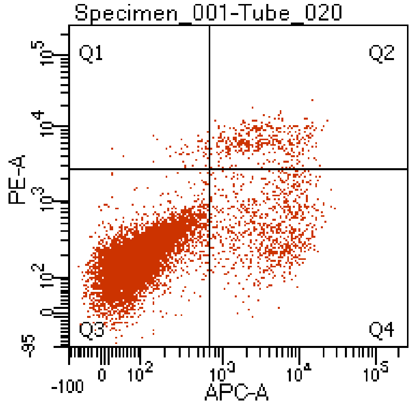

Tube: Tube_020			
Population	#Events	%Parent	%Total
All Events	11,800	####	100.0
P1	11,064	93.8	93.8
P2	10,023	90.6	84.9
Q1	55	0.5	0.5
Q2	400	4.0	3.4
Q3	8,691	86.7	73.7
Q4	877	8.7	7.4

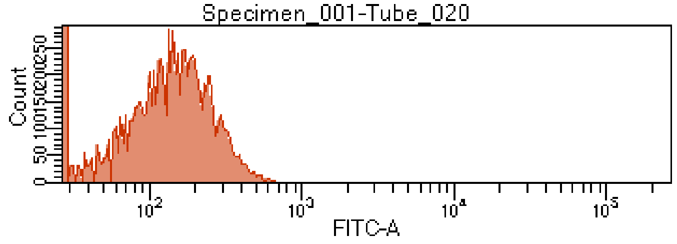

Tube Name: Tube_020

GUID: 3d96f0b6-85fe-47b6-9446-fa320783e658

Population	#Events	%Parent	PE-A Mean	PE-A %CV	APC-A Mean	APC-A %CV	APC-Cy7-A Mean	APC-Cy7-A %CV
All Events	11,800	####	547	301.6	819	308.0	483	320.6
P1	11,064	93.8	517	277.4	795	285.7	468	296.1
P2	10,023	90.6	544	273.0	763	297.7	447	308.7
Q1	55	0.5	5,052	25.5	442	39.3	254	43.0
Q2	400	4.0	6,922	40.3	4,726	85.9	2,863	89.5
Q3	8,691	86.7	212	76.1	106	102.1	55	115.2
Q4	877	8.7	641	91.5	5,478	76.6	3,245	80.1
