# Supplementary material for: Salvia chinensis Benth Inhibits Triple-Negative Breast Cancer Progression by Inducing the DNA Damage Pathway
Source: Front Oncol. 2022 Aug 10;12:882784. doi: 10.3389/fonc.2022.882784 (PMC9404549; doi:10.3389/fonc.2022.882784)
Supplement: Supplementary file 18 [file DataSheet_11.zip › other raw data/figure 2a/31.4T1-100mg-1.pdf]

BD FACSDiva 8.0.1

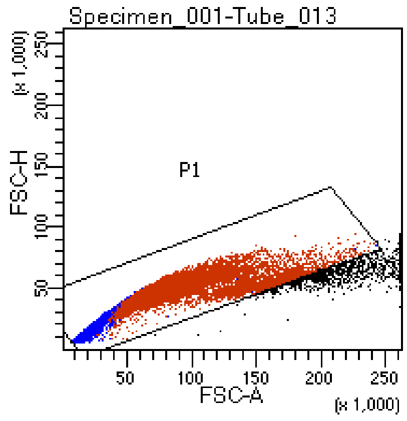

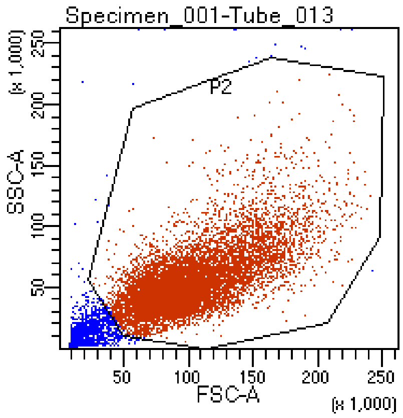

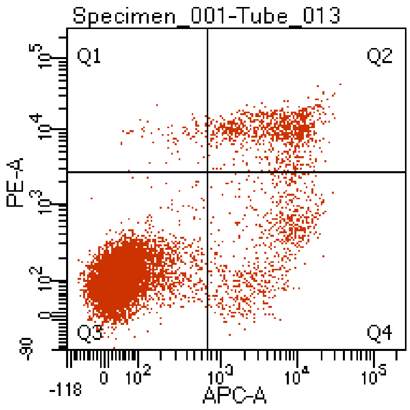

Tube: Tube_013

Population	#Events	%Parent	%Total
All Events	12,892	####	100.0
P1	11,456	88.9	88.9
P2	9,882	86.3	76.7
Q1	74	0.7	0.6
Q2	924	9.4	7.2
Q3	8,137	82.3	63.1
Q4	747	7.6	5.8

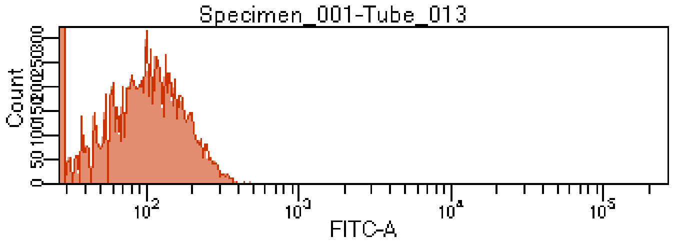

Tube Name: Tube_013
GUID: 16c92611-6278-4da3-8fc5-32b75fa8498c

Population	#Events	%Parent	PE-A Mean	PE-A %CV	APC-A Mean	APC-A %CV	APC-Cy7-A Mean	APC-Cy7-A %CV
All Events	12,892	####	1,117	329.5	1,217	265.7	712	276.5
P1	11,456	88.9	1,105	304.6	1,274	248.2	747	256.4
P2	9,882	86.3	1,226	291.7	1,149	278.8	670	288.7
Q1	74	0.7	9,447	35.8	355	56.4	188	62.3
Q2	924	9.4	10,903	46.1	7,070	75.3	4,149	79.1
Q3	8,137	82.3	118	83.6	48	163.8	22	215.4
Q4	747	7.6	516	117.0	5,897	78.6	3,478	85.2
