# Supplementary material for: Salvia chinensis Benth Inhibits Triple-Negative Breast Cancer Progression by Inducing the DNA Damage Pathway
Source: Front Oncol. 2022 Aug 10;12:882784. doi: 10.3389/fonc.2022.882784 (PMC9404549; doi:10.3389/fonc.2022.882784)
Supplement: Supplementary file 18 [file DataSheet_11.zip › other raw data/figure 2a/32.4T1-100mg-2.pdf]

BD FACSDiva 8.0.1

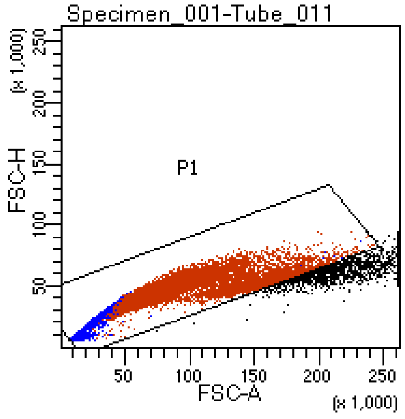

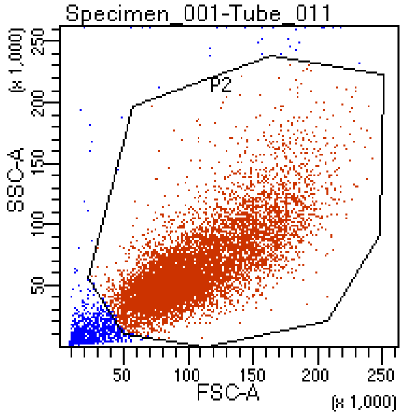

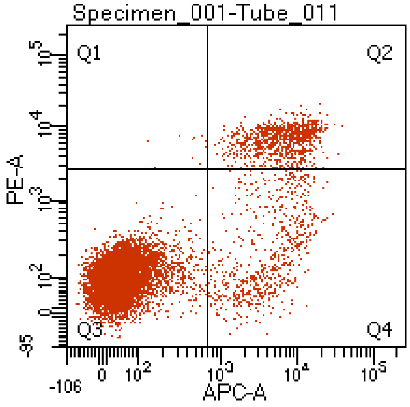

Tube: Tube_011

Population	#Events	%Parent	%Total
All Events	12,937	####	100.0
P1	10,969	84.8	84.8
P2	9,931	90.5	76.8
Q1	13	0.1	0.1
Q2	919	9.3	7.1
Q3	8,336	83.9	64.4
Q4	663	6.7	5.1

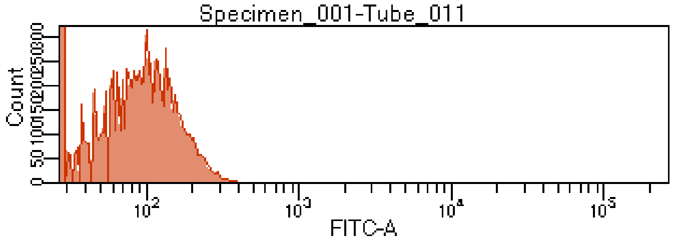

Tube Name: Tube_011
GUID: a11527c7-aab2-403d-96ba-f16f21501e30

Population	#Events	%Parent	PE-A Mean	PE-A %CV	APC-A Mean	APC-A %CV	APC-Cy7-A Mean	APC-Cy7-A %CV
All Events	12,937	####	802	305.8	1,099	279.2	625	288.4
P1	10,969	84.8	759	293.3	1,168	269.1	668	277.3
P2	9,931	90.5	795	283.4	1,119	282.8	639	291.1
Q1	13	0.1	5,503	34.9	464	40.0	267	43.6
Q2	919	9.3	7,290	37.7	7,697	66.8	4,426	69.5
Q3	8,336	83.9	99	69.4	45	160.5	21	216.9
Q4	663	6.7	463	134.4	5,518	79.8	3,171	86.8
