# Supplementary material for: Salvia chinensis Benth Inhibits Triple-Negative Breast Cancer Progression by Inducing the DNA Damage Pathway
Source: Front Oncol. 2022 Aug 10;12:882784. doi: 10.3389/fonc.2022.882784 (PMC9404549; doi:10.3389/fonc.2022.882784)
Supplement: Supplementary file 18 [file DataSheet_11.zip › other raw data/figure 2a/33.4T1-100mg-3.pdf]

BD FACSDiva 8.0.1

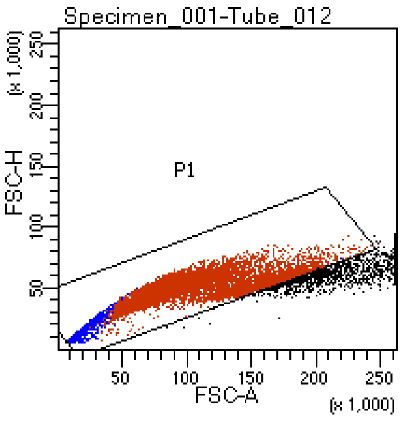

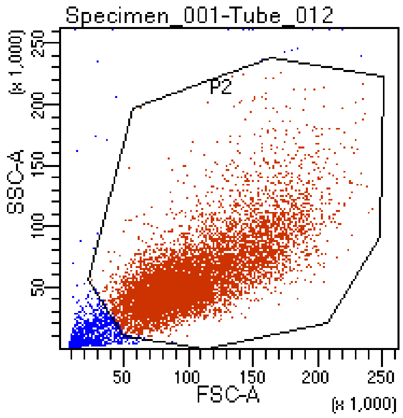

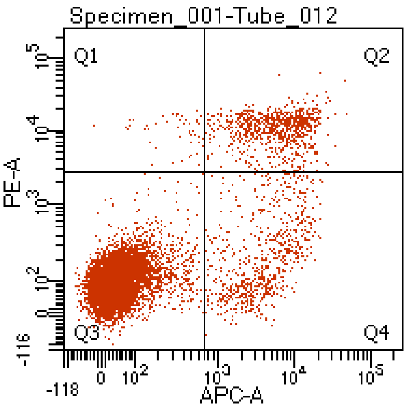

Tube: Tube_012

Population	#Events	%Parent	%Total
All Events	12,902	####	100.0
P1	11,120	86.2	86.2
P2	9,920	89.2	76.9
Q1	50	0.5	0.4
Q2	818	8.2	6.3
Q3	8,346	84.1	64.7
Q4	706	7.1	5.5

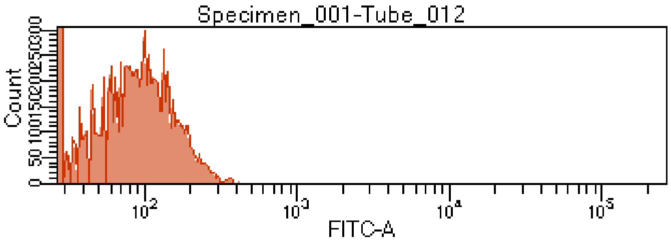

Tube Name: Tube_012
GUID: 9efd47fe-4ef0-493a-9872-9c3025479824

Population	#Events	%Parent	PE-A Mean	PE-A %CV	APC-A Mean	APC-A %CV	APC-Cy7-A Mean	APC-Cy7-A %CV
All Events	12,902	####	1,051	337.1	1,019	302.5	573	313.0
P1	11,120	86.2	1,062	329.2	1,095	285.3	618	295.0
P2	9,920	89.2	1,127	322.1	1,060	300.6	599	310.7
Q1	50	0.5	10,508	40.7	355	51.8	193	49.0
Q2	818	8.2	11,607	48.7	7,841	75.4	4,462	78.9
Q3	8,346	84.1	104	96.6	44	164.9	20	221.6
Q4	706	7.1	409	141.6	5,275	82.7	2,990	90.1
