# Supplementary material for: Salvia chinensis Benth Inhibits Triple-Negative Breast Cancer Progression by Inducing the DNA Damage Pathway
Source: Front Oncol. 2022 Aug 10;12:882784. doi: 10.3389/fonc.2022.882784 (PMC9404549; doi:10.3389/fonc.2022.882784)
Supplement: Supplementary file 18 [file DataSheet_11.zip › other raw data/figure 2a/34.4T1-200mg-1.pdf]

BD FACSDiva 8.0.1

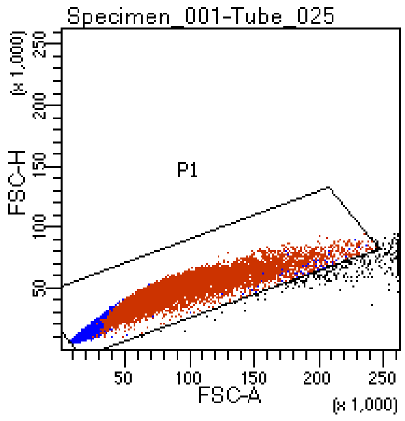

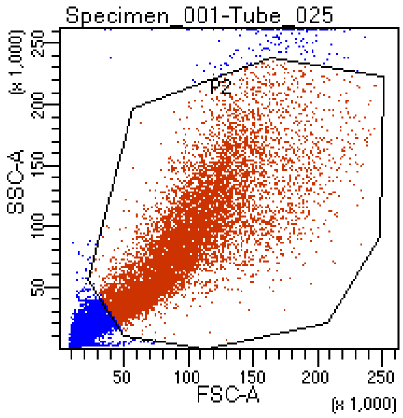

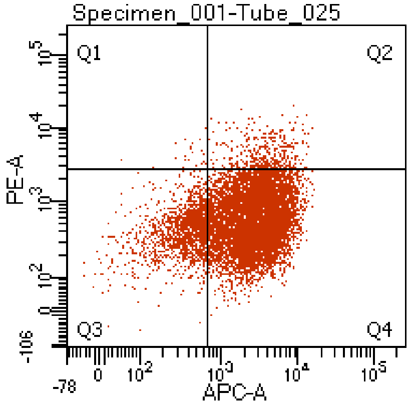

Tube: Tube_025

Population	#Events	%Parent	%Total
All Events	18,099	####	100.0
P1	16,524	91.3	91.3
P2	9,343	56.5	51.6
Q1	26	0.3	0.1
Q2	437	4.7	2.4
Q3	1,499	16.0	8.3
Q4	7,381	79.0	40.8

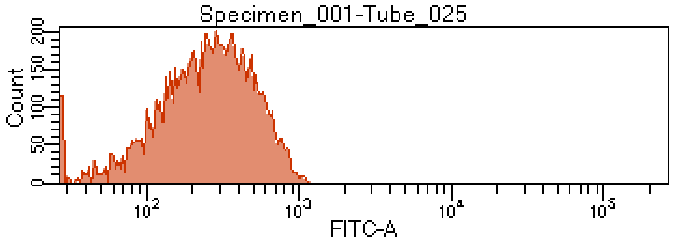

Tube Name: Tube_025
GUID: 5546ce02-5c72-4f1e-be21-939085d2ee10

Population	#Events	%Parent	PE-A Mean	PE-A %CV	APC-A Mean	APC-A %CV	APC-Cy7-A Mean	APC-Cy7-A %CV
All Events	18,099	####	581	178.2	1,814	126.7	1,066	130.4
P1	16,524	91.3	585	169.5	1,865	117.3	1,096	120.9
P2	9,343	56.5	889	128.7	2,839	79.6	1,669	83.0
Q1	26	0.3	4,270	41.6	434	38.9	264	32.6
Q2	437	4.7	4,633	55.6	4,402	60.4	2,698	64.1
Q3	1,499	16.0	495	68.9	382	45.7	215	48.4
Q4	7,381	79.0	735	75.0	3,253	65.0	1,909	68.2
