# Supplementary material for: Salvia chinensis Benth Inhibits Triple-Negative Breast Cancer Progression by Inducing the DNA Damage Pathway
Source: Front Oncol. 2022 Aug 10;12:882784. doi: 10.3389/fonc.2022.882784 (PMC9404549; doi:10.3389/fonc.2022.882784)
Supplement: Supplementary file 18 [file DataSheet_11.zip › other raw data/figure 2a/35.4T1-200mg-2.pdf]

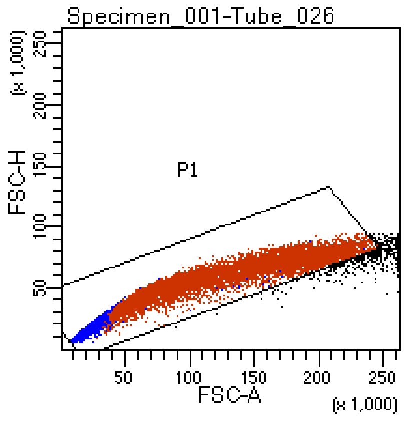

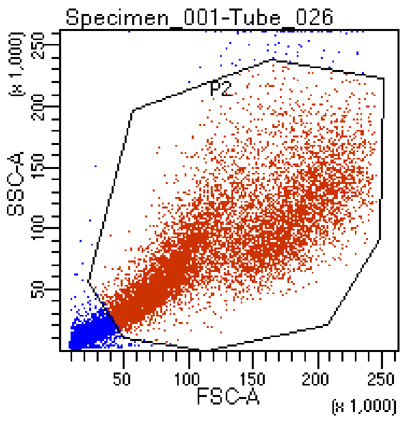

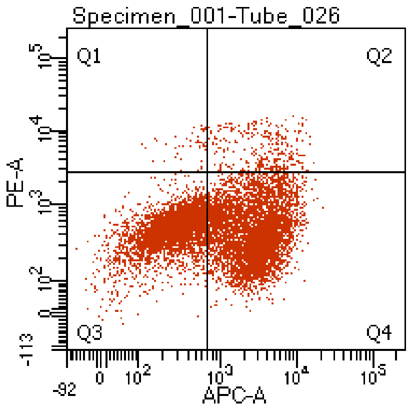

Tube: Tube_026

Population	#Events	%Parent	%Total
All Events	15,393	####	100.0
P1	13,747	89.3	89.3
P2	9,757	71.0	63.4
Q1	50	0.5	0.3
Q2	323	3.3	2.1
Q3	3,938	40.4	25.6
Q4	5,446	55.8	35.4

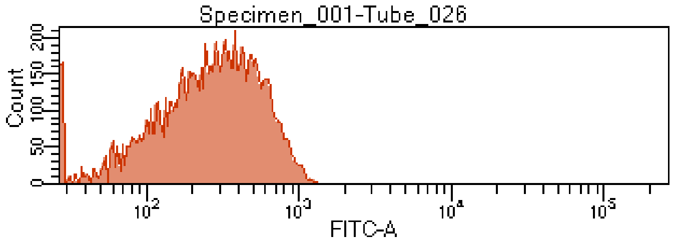

Tube Name: Tube_026
GUID: 98a21ede-b553-4a1c-828d-c68d06876669

Population	#Events	%Parent	PE-A Mean	PE-A %CV	APC-A Mean	APC-A %CV	APC-Cy7-A Mean	APC-Cy7-A %CV
All Events	15,393	####	636	211.4	1,610	144.1	957	148.9
P1	13,747	89.3	609	198.2	1,660	130.2	986	134.6
P2	9,757	71.0	772	168.5	2,071	110.5	1,231	114.6
Q1	50	0.5	6,493	36.8	448	37.9	274	36.9
Q2	323	3.3	6,111	52.5	4,662	67.1	2,921	69.0
Q3	3,938	40.4	525	50.8	297	56.7	166	60.2
Q4	5,446	55.8	581	85.6	3,214	68.0	1,909	71.5
