# Supplementary material for: Salvia chinensis Benth Inhibits Triple-Negative Breast Cancer Progression by Inducing the DNA Damage Pathway
Source: Front Oncol. 2022 Aug 10;12:882784. doi: 10.3389/fonc.2022.882784 (PMC9404549; doi:10.3389/fonc.2022.882784)
Supplement: Supplementary file 18 [file DataSheet_11.zip › other raw data/figure 2a/36.4T1-200mg-3.pdf]

BD FACSDiva 8.0.1

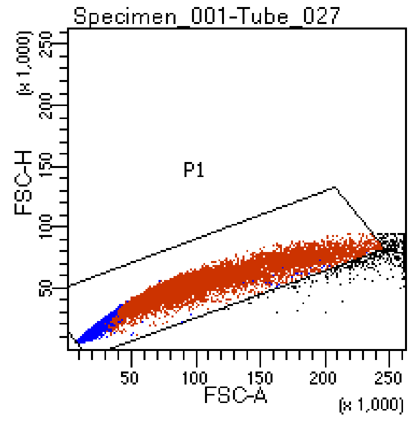

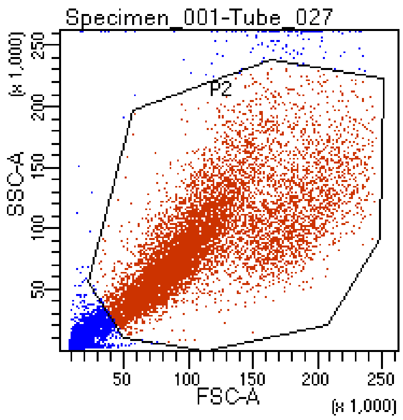

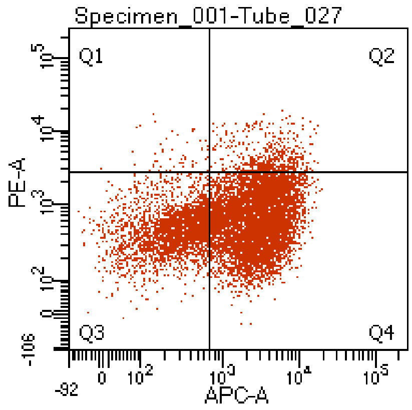

Tube: Tube_027

Population	#Events	%Parent	%Total
All Events	15,830	####	100.0
P1	14,319	90.5	90.5
P2	9,750	68.1	61.6
Q1	57	0.6	0.4
Q2	529	5.4	3.3
Q3	2,725	27.9	17.2
Q4	6,439	66.0	40.7

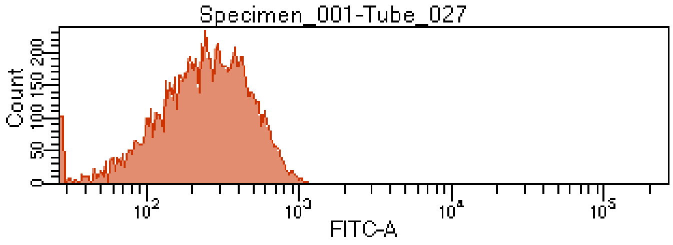

Tube Name: Tube_027
GUID: e41866e3-faaf-4afe-a2b6-0173dab44999

Population	#Events	%Parent	PE-A Mean	PE-A %CV	APC-A Mean	APC-A %CV	APC-Cy7-A Mean	APC-Cy7-A %CV
All Events	15,830	####	730	180.5	1,871	135.7	1,108	139.8
P1	14,319	90.5	731	165.5	1,942	124.8	1,150	128.7
P2	9,750	68.1	967	136.4	2,555	97.0	1,514	100.6
Q1	57	0.6	5,313	57.7	304	56.2	167	66.2
Q2	529	5.4	4,823	57.5	4,759	63.4	2,905	65.7
Q3	2,725	27.9	545	68.3	305	60.8	171	63.6
Q4	6,439	66.0	790	76.9	3,347	68.9	1,980	72.4
