# Supplementary material for: Salvia chinensis Benth Inhibits Triple-Negative Breast Cancer Progression by Inducing the DNA Damage Pathway
Source: Front Oncol. 2022 Aug 10;12:882784. doi: 10.3389/fonc.2022.882784 (PMC9404549; doi:10.3389/fonc.2022.882784)
Supplement: Supplementary file 18 [file DataSheet_11.zip › other raw data/figure 2a/4.MDAMB231-50mg-1.pdf]

BD FACSDiva 8.0.1

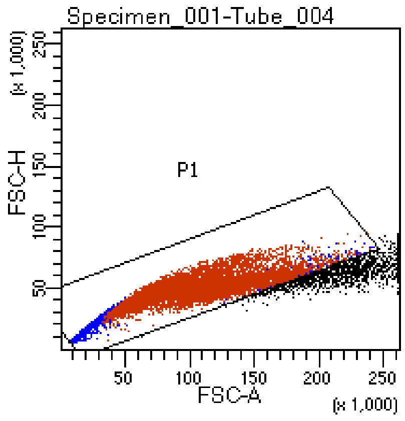

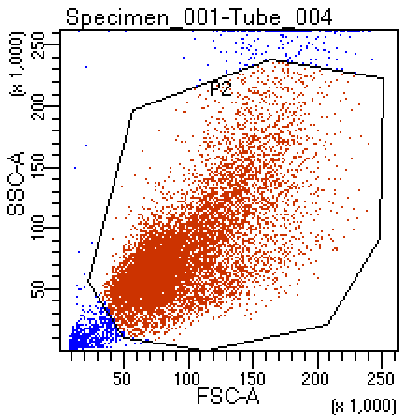

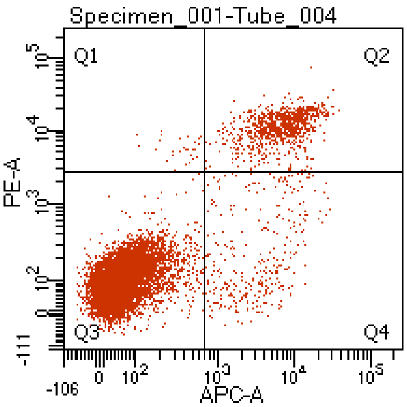

Tube: Tube_004

Population	#Events	%Parent	%Total
All Events	12,967	####	100.0
P1	10,821	83.5	83.5
P2	9,882	91.3	76.2
Q1	30	0.3	0.2
Q2	968	9.8	7.5
Q3	8,561	86.6	66.0
Q4	323	3.3	2.5

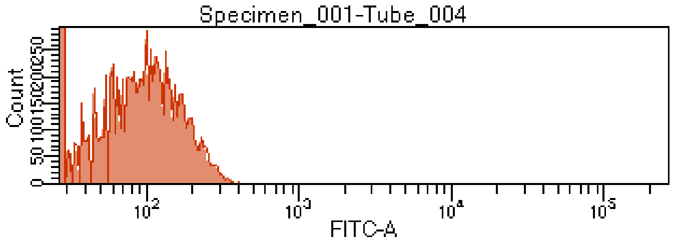

Tube Name: Tube_004
GUID: b9bc6cd3-74d2-4877-b923-276db9d6ae2c

Population	#Events	%Parent	PE-A Mean	PE-A %CV	APC-A Mean	APC-A %CV	APC-Cy7-A Mean	APC-Cy7-A %CV
All Events	12,967	####	1,366	329.2	1,034	301.0	579	314.0
P1	10,821	83.5	1,284	303.6	1,054	298.3	595	308.1
P2	9,882	91.3	1,324	300.9	1,021	310.9	575	321.6
Q1	30	0.3	5,710	34.1	394	40.6	228	44.5
Q2	968	9.8	12,198	44.5	8,235	66.1	4,685	68.6
Q3	8,561	86.6	109	85.8	63	123.5	28	163.5
Q4	323	3.3	516	140.0	4,854	101.7	2,787	114.7
