# Supplementary material for: Salvia chinensis Benth Inhibits Triple-Negative Breast Cancer Progression by Inducing the DNA Damage Pathway
Source: Front Oncol. 2022 Aug 10;12:882784. doi: 10.3389/fonc.2022.882784 (PMC9404549; doi:10.3389/fonc.2022.882784)
Supplement: Supplementary file 18 [file DataSheet_11.zip › other raw data/figure 2a/5.MDAMB231-50mg-2.pdf]

BD FACSDiva 8.0.1

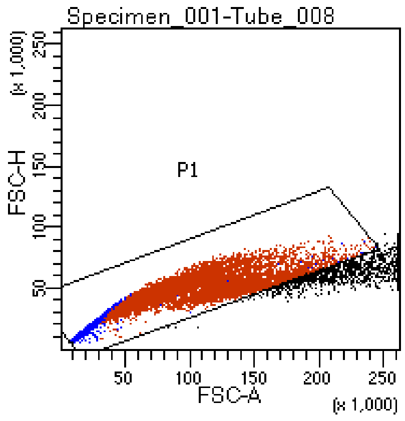

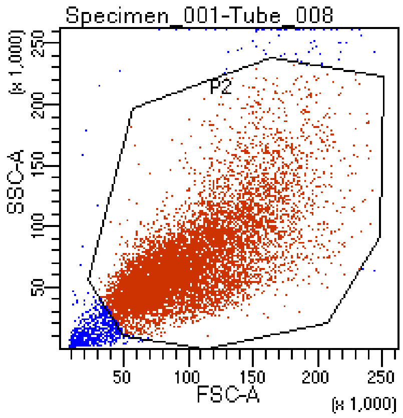

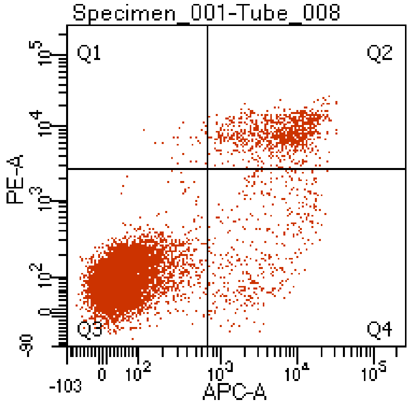

Tube: Tube_008

Population	#Events	%Parent	%Total
All Events	12,714	####	100.0
P1	10,684	84.0	84.0
P2	9,878	92.5	77.7
Q1	25	0.3	0.2
Q2	927	9.4	7.3
Q3	8,512	86.2	66.9
Q4	414	4.2	3.3

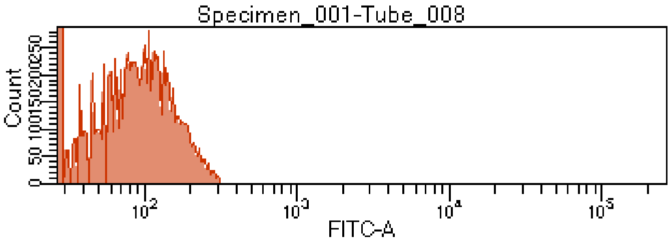

Tube Name: Tube_008

GUID: f60be860-e571-47ad-9c3b-bbf0247cd004

Population	#Events	%Parent	PE-A Mean	PE-A %CV	APC-A Mean	APC-A %CV	APC-Cy7-A Mean	APC-Cy7-A %CV
All Events	12,714	####	998	312.2	1,028	308.8	580	321.0
P1	10,684	84.0	962	299.6	1,072	298.1	607	307.4
P2	9,878	92.5	992	295.7	1,033	312.4	584	322.4
Q1	25	0.3	7,136	42.0	422	39.8	243	44.2
Q2	927	9.4	9,197	42.7	8,053	71.3	4,589	74.6
Q3	8,512	86.2	101	91.9	55	135.0	26	174.7
Q4	414	4.2	559	122.1	5,461	92.2	3,120	99.7
