# Supplementary material for: Salvia chinensis Benth Inhibits Triple-Negative Breast Cancer Progression by Inducing the DNA Damage Pathway
Source: Front Oncol. 2022 Aug 10;12:882784. doi: 10.3389/fonc.2022.882784 (PMC9404549; doi:10.3389/fonc.2022.882784)
Supplement: Supplementary file 18 [file DataSheet_11.zip › other raw data/figure 2a/6.MDAMB231-50mg-3.pdf]

BD FACSDiva 8.0.1

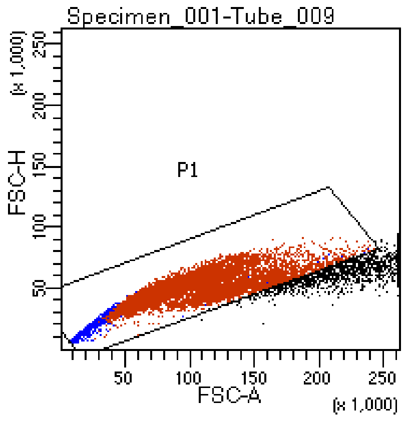

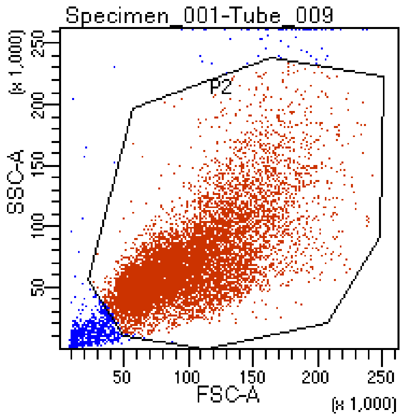

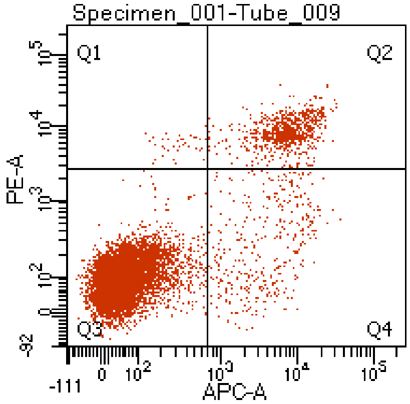

Tube: Tube_009

Population	#Events	%Parent	%Total
All Events	12,711	####	100.0
P1	10,736	84.5	84.5
P2	9,884	92.1	77.8
Q1	36	0.4	0.3
Q2	826	8.4	6.5
Q3	8,652	87.5	68.1
Q4	370	3.7	2.9

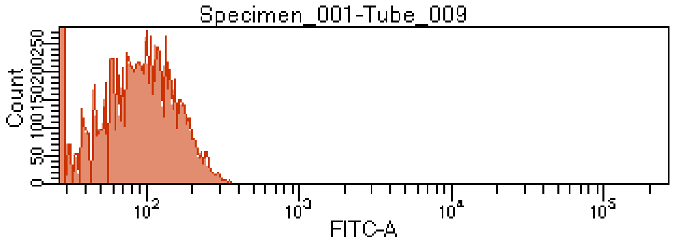

Tube Name: Tube_009
GUID: ead0cb79-7dfb-4123-b609-342b2aa772e2

Population	#Events	%Parent	PE-A Mean	PE-A %CV	APC-A Mean	APC-A %CV	APC-Cy7-A Mean	APC-Cy7-A %CV
All Events	12,711	####	935	323.2	1,004	303.1	567	313.0
P1	10,736	84.5	905	318.3	1,050	298.0	597	306.7
P2	9,884	92.1	937	316.4	997	316.0	565	325.5
Q1	36	0.4	5,918	27.7	332	46.9	183	55.3
Q2	826	8.4	9,656	46.8	8,837	58.9	5,057	62.1
Q3	8,652	87.5	102	86.9	55	139.7	26	179.7
Q4	370	3.7	533	122.5	5,569	90.7	3,167	97.8
