# Supplementary material for: Salvia chinensis Benth Inhibits Triple-Negative Breast Cancer Progression by Inducing the DNA Damage Pathway
Source: Front Oncol. 2022 Aug 10;12:882784. doi: 10.3389/fonc.2022.882784 (PMC9404549; doi:10.3389/fonc.2022.882784)
Supplement: Supplementary file 18 [file DataSheet_11.zip › other raw data/figure 2a/7.MDAMB231-100mg-1.pdf]

BD FACSDiva 8.0.1

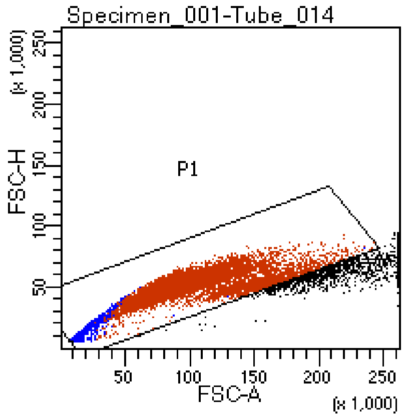

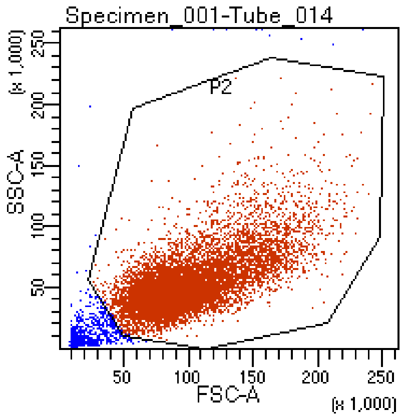

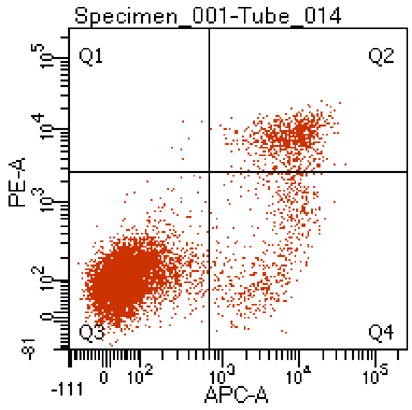

Tube: Tube_014

Population	#Events	%Parent	%Total
All Events	12,175	####	100.0
P1	10,775	88.5	88.5
P2	9,941	92.3	81.7
Q1	7	0.1	0.1
Q2	828	8.3	6.8
Q3	8,495	85.5	69.8
Q4	611	6.1	5.0

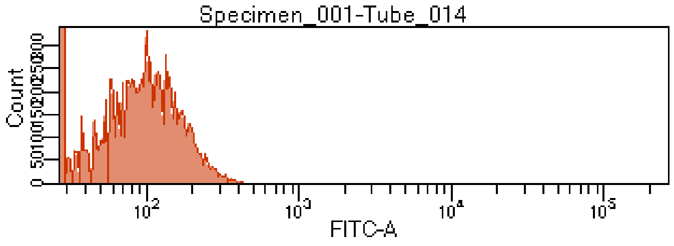

Tube Name: Tube_014
GUID: 6ec7df35-278d-4c20-ad9e-49b85747e5ba

Population	#Events	%Parent	PE-A Mean	PE-A %CV	APC-A Mean	APC-A %CV	APC-Cy7-A Mean	APC-Cy7-A %CV
All Events	12,175	####	820	328.8	1,134	291.8	649	301.0
P1	10,775	88.5	816	307.5	1,192	278.0	683	285.9
P2	9,941	92.3	845	301.7	1,159	290.5	664	298.6
Q1	7	0.1	7,313	57.6	330	35.0	226	34.3
Q2	828	8.3	8,492	42.5	9,185	60.4	5,310	63.1
Q3	8,495	85.5	113	89.5	53	146.3	26	185.2
Q4	611	6.1	591	121.5	5,670	75.2	3,243	82.1
