# Supplementary material for: Salvia chinensis Benth Inhibits Triple-Negative Breast Cancer Progression by Inducing the DNA Damage Pathway
Source: Front Oncol. 2022 Aug 10;12:882784. doi: 10.3389/fonc.2022.882784 (PMC9404549; doi:10.3389/fonc.2022.882784)
Supplement: Supplementary file 18 [file DataSheet_11.zip › other raw data/figure 2a/8.MDAMB231-100mg-2.pdf]

BD FACSDiva 8.0.1

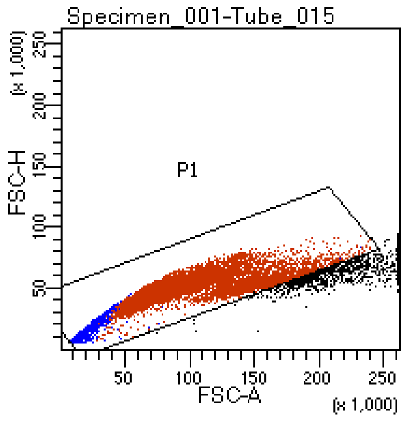

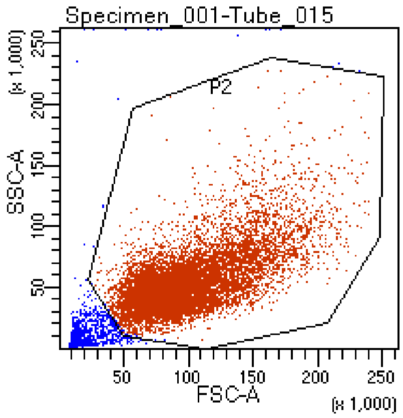

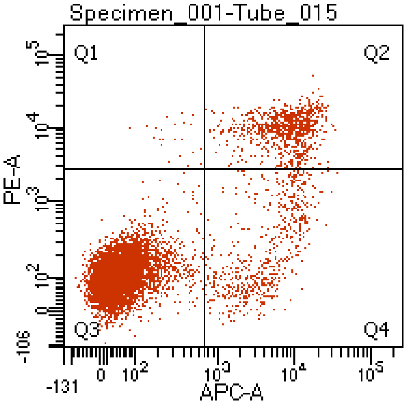

Tube: Tube_015

Population	#Events	%Parent	%Total
All Events	12,156	####	100.0
P1	10,901	89.7	89.7
P2	9,946	91.2	81.8
Q1	23	0.2	0.2
Q2	900	9.0	7.4
Q3	8,421	84.7	69.3
Q4	602	6.1	5.0

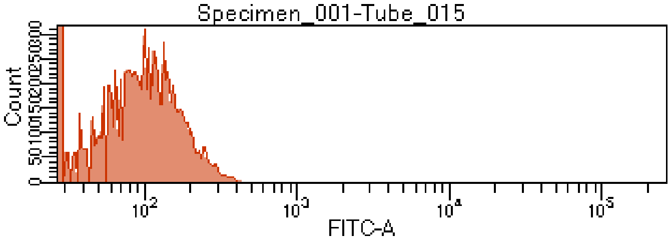

Tube Name: Tube_015
GUID: 39191132-26cd-4397-85c3-9edac4f1d65b

Population	#Events	%Parent	PE-A Mean	PE-A %CV	APC-A Mean	APC-A %CV	APC-Cy7-A Mean	APC-Cy7-A %CV
All Events	12,156	####	1,103	368.0	1,214	289.1	704	299.5
P1	10,901	89.7	1,059	309.2	1,250	277.1	725	286.3
P2	9,946	91.2	1,111	304.5	1,234	287.4	717	296.5
Q1	23	0.2	8,039	50.0	333	45.4	173	47.0
Q2	900	9.0	10,662	45.8	9,337	59.2	5,467	63.1
Q3	8,421	84.7	114	81.5	46	152.3	22	201.4
Q4	602	6.1	516	128.9	5,779	84.0	3,365	91.0
