# Supplementary material for: Salvia chinensis Benth Inhibits Triple-Negative Breast Cancer Progression by Inducing the DNA Damage Pathway
Source: Front Oncol. 2022 Aug 10;12:882784. doi: 10.3389/fonc.2022.882784 (PMC9404549; doi:10.3389/fonc.2022.882784)
Supplement: Supplementary file 18 [file DataSheet_11.zip › other raw data/figure 2a/9.MDAMB231-100mg-3.pdf]

BD FACSDiva 8.0.1

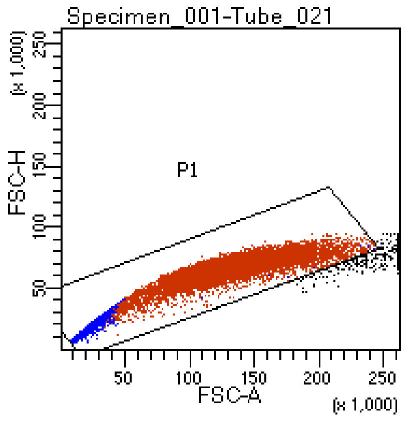

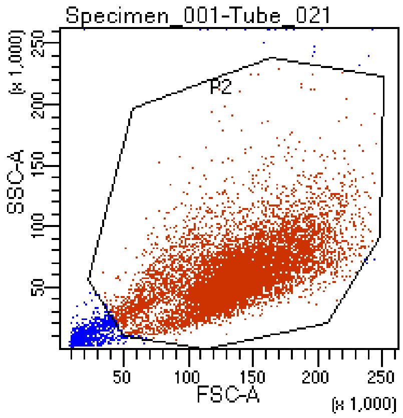

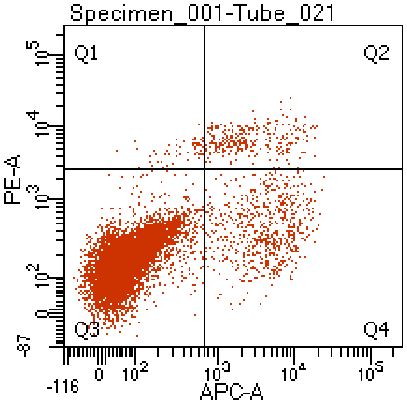

Tube: Tube_021

Population	#Events	%Parent	%Total
All Events	11,695	####	100.0
P1	11,037	94.4	94.4
P2	10,013	90.7	85.6
Q1	58	0.6	0.5
Q2	305	3.0	2.6
Q3	8,970	89.6	76.7
Q4	680	6.8	5.8

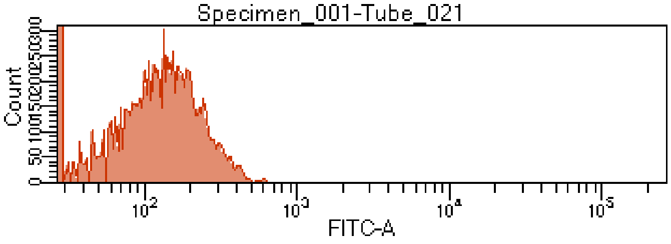

Tube Name: Tube_021
GUID: f5b9535d-e8eb-45f2-b3ea-879f177e0db2

Population	#Events	%Parent	PE-A Mean	PE-A %CV	APC-A Mean	APC-A %CV	APC-Cy7-A Mean	APC-Cy7-A %CV
All Events	11,695	####	486	424.2	604	349.1	355	361.9
P1	11,037	94.4	439	306.5	582	327.2	341	338.3
P2	10,013	90.7	456	302.4	540	348.7	316	360.9
Q1	58	0.6	5,191	28.2	418	44.7	250	47.1
Q2	305	3.0	7,198	41.4	3,884	101.0	2,341	103.8
Q3	8,970	89.6	186	79.0	73	119.5	37	143.9
Q4	680	6.8	596	88.9	5,210	75.7	3,090	78.6
