# Supplementary material for: Salvia chinensis Benth Inhibits Triple-Negative Breast Cancer Progression by Inducing the DNA Damage Pathway
Source: Front Oncol. 2022 Aug 10;12:882784. doi: 10.3389/fonc.2022.882784 (PMC9404549; doi:10.3389/fonc.2022.882784)
Supplement: Supplementary file 18 [file DataSheet_11.zip › other raw data/figure 4a/1.231-V1.pdf]

BD FACSDiva 8.0.1

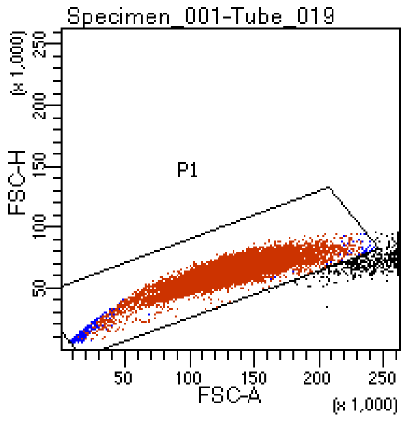

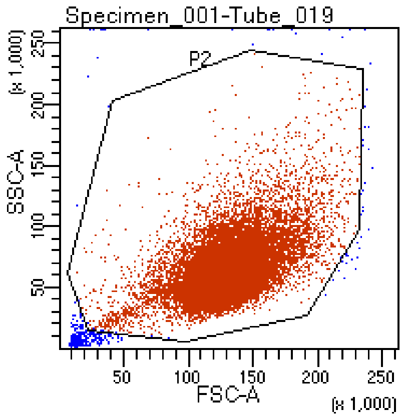

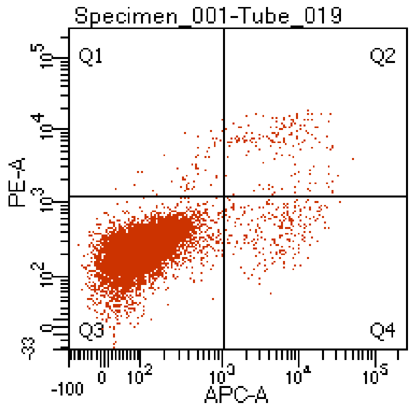

Tube: Tube_019

Population	#Events	%Parent	%Total
All Events	21,532	####	100.0
P1	20,580	95.6	95.6
P2	20,045	97.4	93.1
Q1	98	0.5	0.5
Q2	389	1.9	1.8
Q3	19,049	95.0	88.5
Q4	509	2.5	2.4

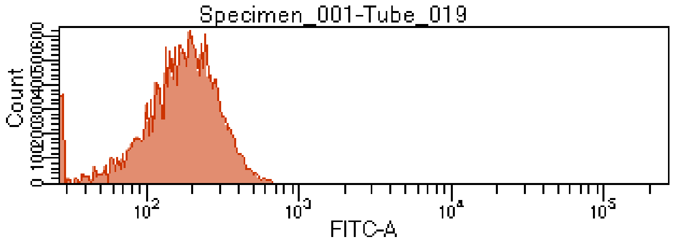

Tube Name: Tube_019
GUID: ee9efc8f-ea84-4b90-be25-44d69abae4ee

Population	#Events	%Parent	PE-A Mean	PE-A %CV	APC-A Mean	APC-A %CV	APC-Cy7-A Mean	APC-Cy7-A %CV
All Events	21,532	####	445	304.6	479	437.5	272	470.5
P1	20,580	95.6	424	297.9	462	441.9	263	476.5
P2	20,045	97.4	423	294.5	451	454.0	256	490.0
Q1	98	0.5	3,732	68.0	519	46.8	294	47.1
Q2	389	1.9	7,794	56.1	7,997	93.6	4,758	100.7
Q3	19,049	95.0	254	51.2	122	92.2	62	106.8
Q4	509	2.5	502	54.2	7,000	80.5	4,105	87.3
