# Supplementary material for: Salvia chinensis Benth Inhibits Triple-Negative Breast Cancer Progression by Inducing the DNA Damage Pathway
Source: Front Oncol. 2022 Aug 10;12:882784. doi: 10.3389/fonc.2022.882784 (PMC9404549; doi:10.3389/fonc.2022.882784)
Supplement: Supplementary file 18 [file DataSheet_11.zip › other raw data/figure 4a/10.231-Combo-1.pdf]

BD FACSDiva 8.0.1

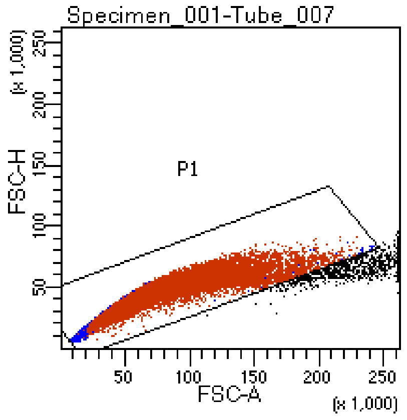

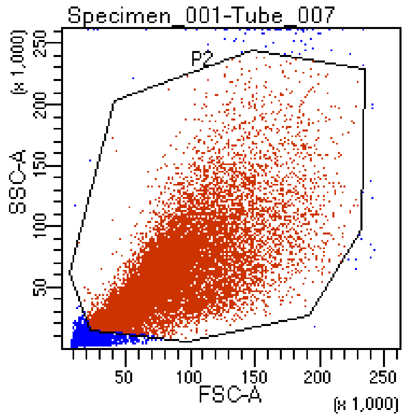

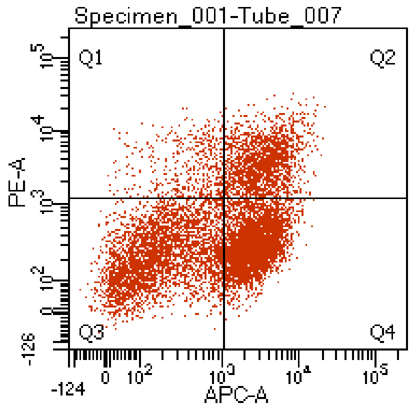

Tube: Tube_007

Population	#Events	%Parent	%Total
All Events	30,943	####	100.0
P1	28,644	92.6	92.6
P2	20,955	73.2	67.7
Q1	981	4.7	3.2
Q2	3,622	17.3	11.7
Q3	7,346	35.1	23.7
Q4	9,006	43.0	29.1

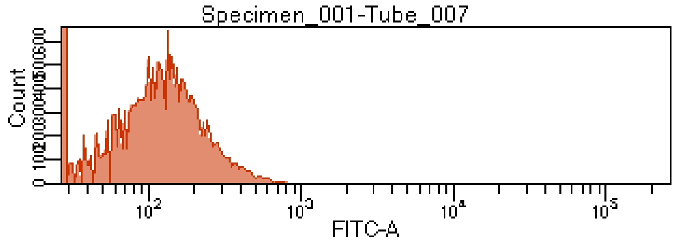

Tube Name: Tube_007
GUID: 6eab69f8-fb4e-4f2b-b511-26d1f722cbba

Population	#Events	%Parent	PE-A Mean	PE-A %CV	APC-A Mean	APC-A %CV	APC-Cy7-A Mean	APC-Cy7-A %CV
All Events	30,943	####	1,005	227.8	1,584	126.6	967	130.3
P1	28,644	92.6	1,014	222.5	1,654	121.3	1,011	124.7
P2	20,955	73.2	1,277	193.1	2,068	102.8	1,266	105.7
Q1	981	4.7	4,525	79.7	527	57.0	306	58.4
Q2	3,622	17.3	4,697	75.9	4,082	64.0	2,530	67.1
Q3	7,346	35.1	291	86.2	278	100.3	157	106.6
Q4	9,006	43.0	351	65.2	2,887	55.1	1,768	56.2
