# Supplementary material for: Salvia chinensis Benth Inhibits Triple-Negative Breast Cancer Progression by Inducing the DNA Damage Pathway
Source: Front Oncol. 2022 Aug 10;12:882784. doi: 10.3389/fonc.2022.882784 (PMC9404549; doi:10.3389/fonc.2022.882784)
Supplement: Supplementary file 18 [file DataSheet_11.zip › other raw data/figure 4a/11.231-Combo-2.pdf]

BD FACSDiva 8.0.1

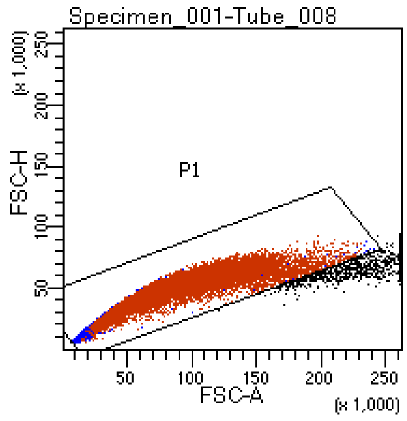

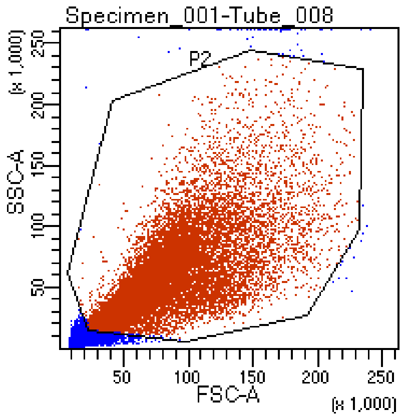

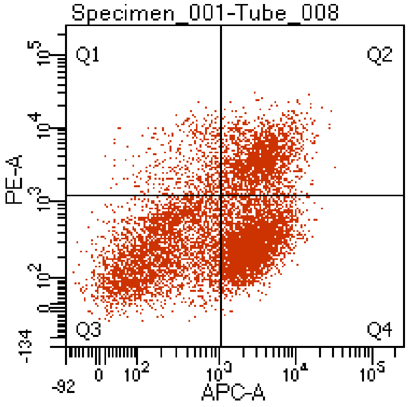

Tube: Tube_008

Population	#Events	%Parent	%Total
All Events	30,328	####	100.0
P1	28,096	92.6	92.6
P2	20,909	74.4	68.9
Q1	986	4.7	3.3
Q2	3,723	17.8	12.3
Q3	6,909	33.0	22.8
Q4	9,291	44.4	30.6

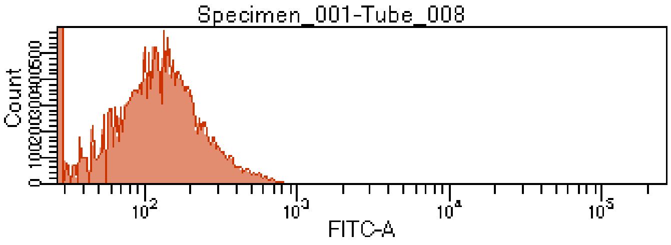

Tube Name: Tube_008
GUID: 3f98551d-f47a-4182-a1b5-26ea2403808b

Population	#Events	%Parent	PE-A Mean	PE-A %CV	APC-A Mean	APC-A %CV	APC-Cy7-A Mean	APC-Cy7-A %CV
All Events	30,328	####	1,025	226.2	1,705	130.4	1,055	134.0
P1	28,096	92.6	1,027	215.6	1,775	122.7	1,100	126.1
P2	20,909	74.4	1,276	190.5	2,210	104.7	1,373	107.6
Q1	986	4.7	4,319	81.7	552	52.5	329	53.8
Q2	3,723	17.8	4,589	76.4	4,136	70.8	2,605	74.0
Q3	6,909	33.0	305	86.0	301	92.3	170	98.4
Q4	9,291	44.4	347	66.3	3,035	60.5	1,884	61.3
