# Supplementary material for: Salvia chinensis Benth Inhibits Triple-Negative Breast Cancer Progression by Inducing the DNA Damage Pathway
Source: Front Oncol. 2022 Aug 10;12:882784. doi: 10.3389/fonc.2022.882784 (PMC9404549; doi:10.3389/fonc.2022.882784)
Supplement: Supplementary file 18 [file DataSheet_11.zip › other raw data/figure 4a/12.231-Combo-3.pdf]

BD FACSDiva 8.0.1

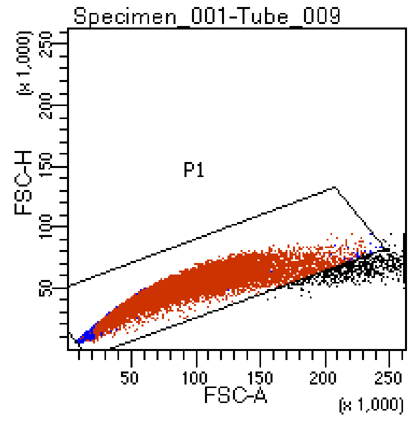

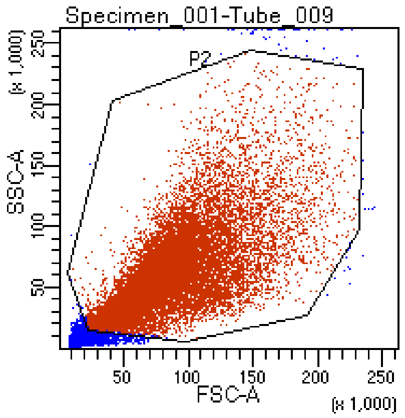

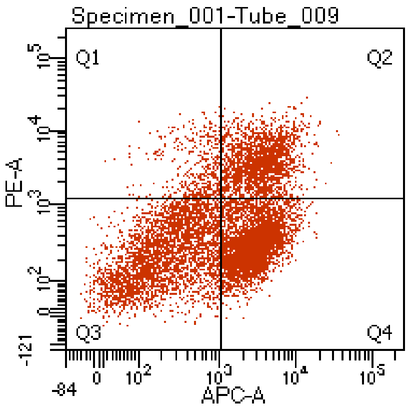

Tube: Tube_009

Population	#Events	%Parent	%Total
All Events	31,058	####	100.0
P1	28,783	92.7	92.7
P2	20,939	72.7	67.4
Q1	875	4.2	2.8
Q2	3,988	19.0	12.8
Q3	6,774	32.4	21.8
Q4	9,302	44.4	30.0

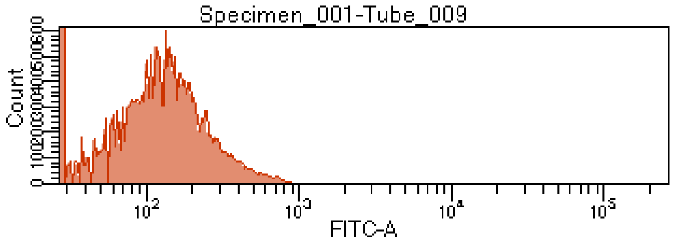

Tube Name: Tube_009

GUID: ff703edb-0f15-446e-aeba-8e796ecdaab8

Population	#Events	%Parent	PE-A Mean	PE-A %CV	APC-A Mean	APC-A %CV	APC-Cy7-A Mean	APC-Cy7-A %CV
All Events	31,058	####	1,028	213.6	1,724	126.4	1,088	130.6
P1	28,783	92.7	1,044	208.5	1,800	120.8	1,137	124.8
P2	20,939	72.7	1,315	181.8	2,274	101.8	1,440	105.3
Q1	875	4.2	4,360	83.1	587	46.1	347	48.0
Q2	3,988	19.0	4,581	70.3	4,159	70.9	2,670	74.7
Q3	6,774	32.4	316	87.9	310	88.7	176	94.8
Q4	9,302	44.4	357	65.7	3,056	57.3	1,937	58.4
