# Supplementary material for: Salvia chinensis Benth Inhibits Triple-Negative Breast Cancer Progression by Inducing the DNA Damage Pathway
Source: Front Oncol. 2022 Aug 10;12:882784. doi: 10.3389/fonc.2022.882784 (PMC9404549; doi:10.3389/fonc.2022.882784)
Supplement: Supplementary file 18 [file DataSheet_11.zip › other raw data/figure 4a/13.HCC1187-V1.pdf]

BD FACSDiva 8.0.1

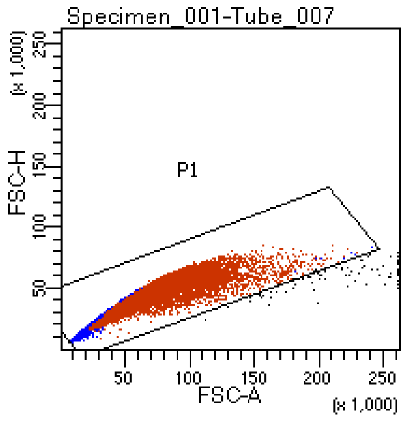

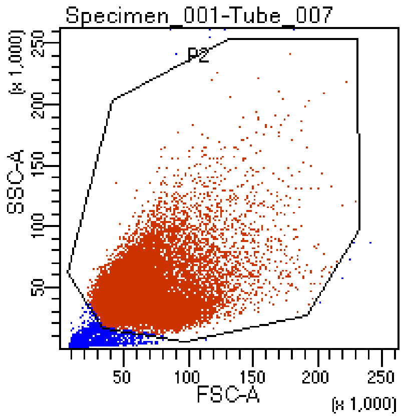

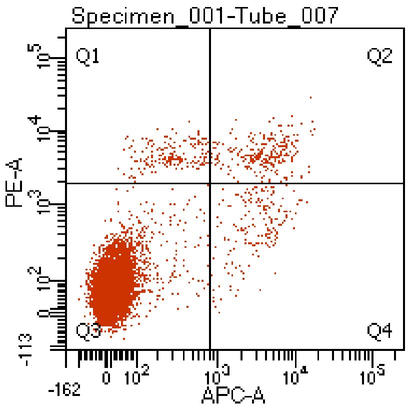

Tube: Tube_007

Population	#Events	%Parent	%Total
All Events	22,457	####	100.0
P1	22,256	99.1	99.1
P2	20,001	89.9	89.1
Q1	338	1.7	1.5
Q2	543	2.7	2.4
Q3	18,752	93.8	83.5
Q4	368	1.8	1.6

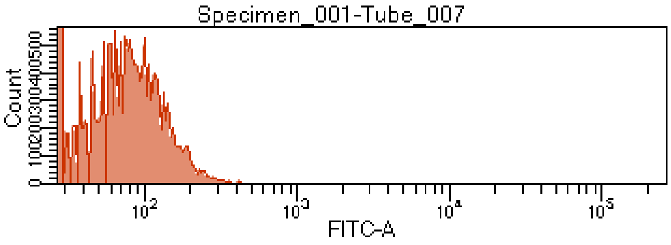

Tube Name: Tube_007
GUID: f8dfb1b7-a0e8-4c64-9e5b-091913bce19a

Population	#Events	%Parent	PE-A Mean	PE-A %CV	APC-A Mean	APC-A %CV	APC-Cy7-A Mean	APC-Cy7-A %CV
All Events	22,457	####	306	398.7	238	479.2	143	494.7
P1	22,256	99.1	297	377.0	231	459.9	139	472.7
P2	20,001	89.9	312	365.3	209	510.2	126	524.4
Q1	338	1.7	4,721	40.5	315	59.7	194	61.4
Q2	543	2.7	5,257	49.2	4,639	66.6	2,832	68.7
Q3	18,752	93.8	82	107.9	12	406.3	6	624.7
Q4	368	1.8	696	67.7	3,612	63.5	2,181	67.0
