# Supplementary material for: Salvia chinensis Benth Inhibits Triple-Negative Breast Cancer Progression by Inducing the DNA Damage Pathway
Source: Front Oncol. 2022 Aug 10;12:882784. doi: 10.3389/fonc.2022.882784 (PMC9404549; doi:10.3389/fonc.2022.882784)
Supplement: Supplementary file 18 [file DataSheet_11.zip › other raw data/figure 4a/14.HCC1187-V2.pdf]

BD FACSDiva 8.0.1

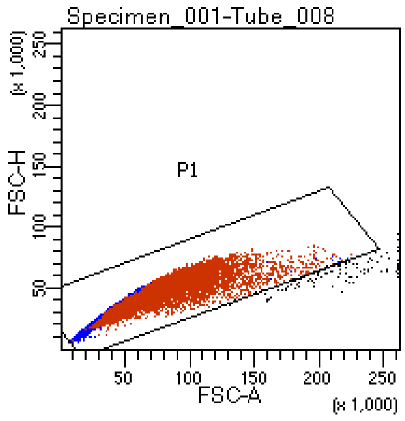

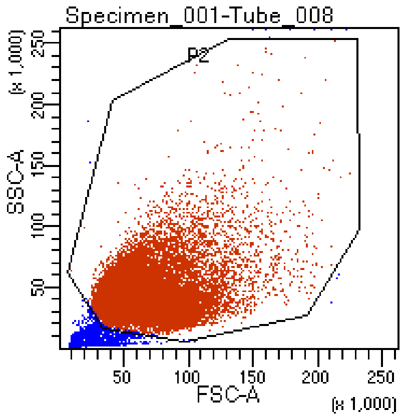

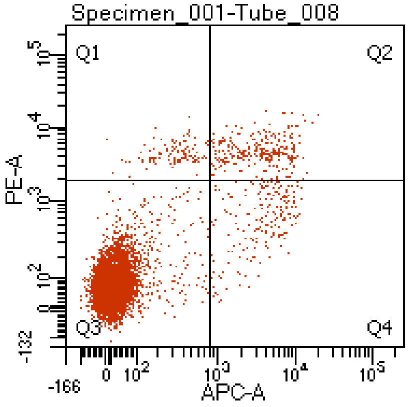

Tube: Tube_008

Population	#Events	%Parent	%Total
All Events	22,393	####	100.0
P1	22,147	98.9	98.9
P2	20,006	90.3	89.3
Q1	240	1.2	1.1
Q2	607	3.0	2.7
Q3	18,761	93.8	83.8
Q4	398	2.0	1.8

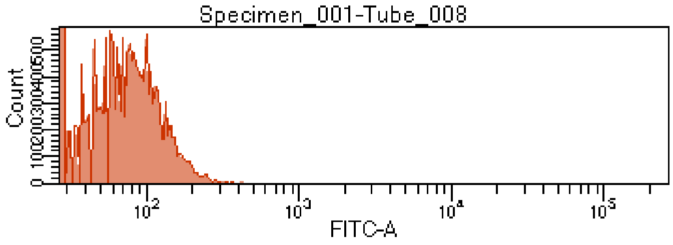

Tube Name: Tube_008

GUID: 70e40bbf-f84a-48fa-b091-31da2c89a595

Population	#Events	%Parent	PE-A Mean	PE-A %CV	APC-A Mean	APC-A %CV	APC-Cy7-A Mean	APC-Cy7-A %CV
All Events	22,393	####	291	388.4	264	459.9	158	472.5
P1	22,147	98.9	284	378.9	257	450.7	154	462.8
P2	20,006	90.3	297	374.6	230	500.8	138	514.7
Q1	240	1.2	4,698	37.2	350	51.4	224	52.6
Q2	607	3.0	5,178	47.6	4,348	71.2	2,659	73.6
Q3	18,761	93.8	74	115.2	11	461.3	4	834.0
Q4	398	2.0	703	70.1	4,182	67.7	2,525	69.4
