# Supplementary material for: Salvia chinensis Benth Inhibits Triple-Negative Breast Cancer Progression by Inducing the DNA Damage Pathway
Source: Front Oncol. 2022 Aug 10;12:882784. doi: 10.3389/fonc.2022.882784 (PMC9404549; doi:10.3389/fonc.2022.882784)
Supplement: Supplementary file 18 [file DataSheet_11.zip › other raw data/figure 4a/15.HCC1187-V3.pdf]

BD FACSDiva 8.0.1

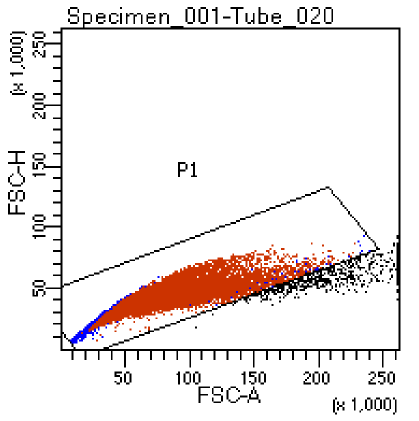

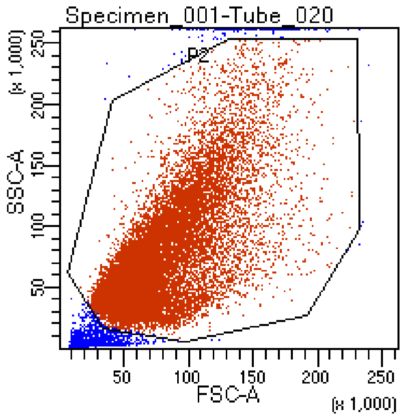

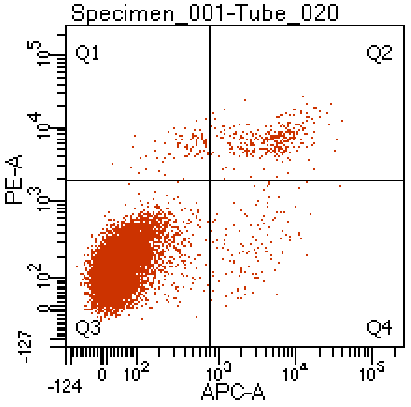

Tube: Tube_020

Population	#Events	%Parent	%Total
All Events	22,854	####	100.0
P1	22,043	96.5	96.5
P2	20,053	91.0	87.7
Q1	172	0.9	0.8
Q2	662	3.3	2.9
Q3	18,965	94.6	83.0
Q4	254	1.3	1.1

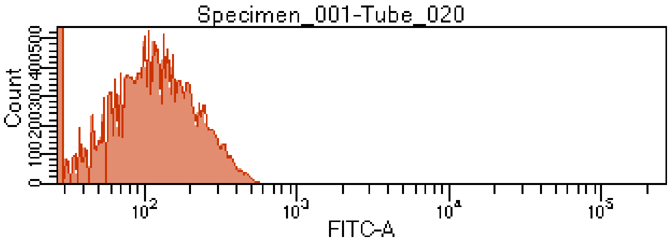

Tube Name: Tube_020

GUID: 162bb222-4af4-431b-8606-78146b5a0260

Population	#Events	%Parent	PE-A Mean	PE-A %CV	APC-A Mean	APC-A %CV	APC-Cy7-A Mean	APC-Cy7-A %CV
All Events	22,854	####	485	359.5	350	545.0	210	579.7
P1	22,043	96.5	448	342.7	325	483.9	194	509.4
P2	20,053	91.0	466	339.7	310	512.2	184	540.4
Q1	172	0.9	6,044	39.9	444	42.6	275	45.1
Q2	662	3.3	7,556	47.7	6,378	81.1	3,900	85.8
Q3	18,965	94.6	169	77.6	48	137.4	24	176.5
Q4	254	1.3	402	97.3	3,964	98.3	2,373	105.6
