# Supplementary material for: Salvia chinensis Benth Inhibits Triple-Negative Breast Cancer Progression by Inducing the DNA Damage Pathway
Source: Front Oncol. 2022 Aug 10;12:882784. doi: 10.3389/fonc.2022.882784 (PMC9404549; doi:10.3389/fonc.2022.882784)
Supplement: Supplementary file 18 [file DataSheet_11.zip › other raw data/figure 4a/16.HCC1187-Q(50uM)-1.pdf]

BD FACSDiva 8.0.1

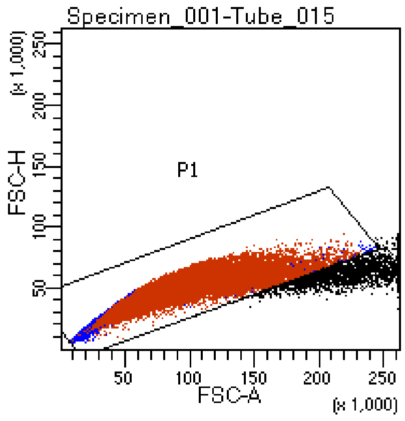

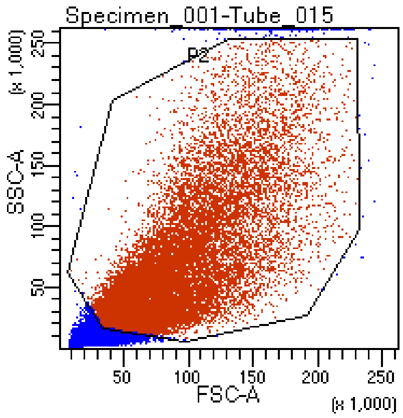

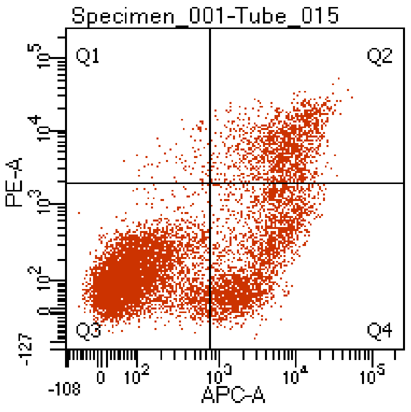

Tube: Tube_015

Population	#Events	%Parent	%Total
All Events	35,995	####	100.0
P1	31,062	86.3	86.3
P2	20,090	64.7	55.8
Q1	153	0.8	0.4
Q2	2,838	14.1	7.9
Q3	12,208	60.8	33.9
Q4	4,891	24.3	13.6

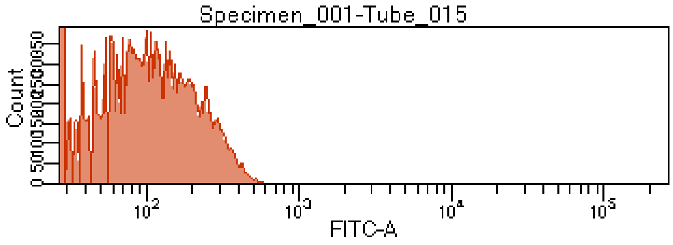

Tube Name: Tube_015

GUID: 69c40bb0-b7f2-4ad1-a055-fb57c9e06c3f

Population	#Events	%Parent	PE-A Mean	PE-A %CV	APC-A Mean	APC-A %CV	APC-Cy7-A Mean	APC-Cy7-A %CV
All Events	35,995	####	1,124	317.0	1,784	207.6	1,084	216.2
P1	31,062	86.3	1,017	302.9	1,888	194.4	1,152	202.5
P2	20,090	64.7	1,383	263.4	2,317	185.3	1,421	192.1
Q1	153	0.8	5,772	65.5	428	44.7	248	49.4
Q2	2,838	14.1	8,229	73.4	8,844	71.5	5,587	73.5
Q3	12,208	60.8	140	103.5	120	127.1	62	135.3
Q4	4,891	24.3	376	118.8	4,073	86.9	2,433	92.7
