# Supplementary material for: Salvia chinensis Benth Inhibits Triple-Negative Breast Cancer Progression by Inducing the DNA Damage Pathway
Source: Front Oncol. 2022 Aug 10;12:882784. doi: 10.3389/fonc.2022.882784 (PMC9404549; doi:10.3389/fonc.2022.882784)
Supplement: Supplementary file 18 [file DataSheet_11.zip › other raw data/figure 4a/17.HCC1187-Q(50uM)-2.pdf]

BD FACSDiva 8.0.1

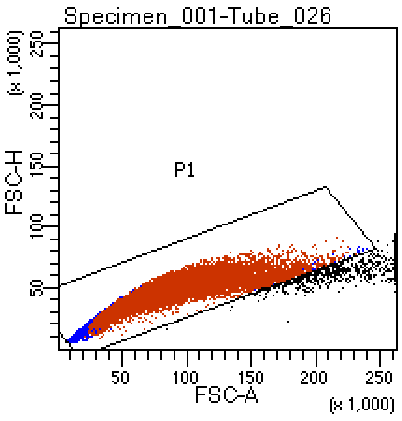

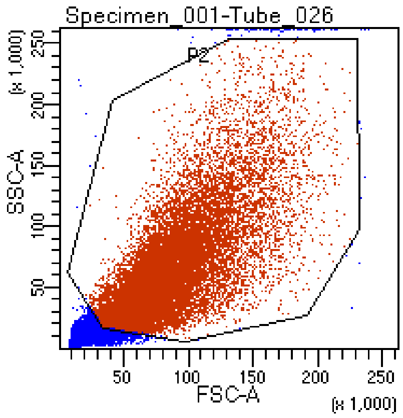

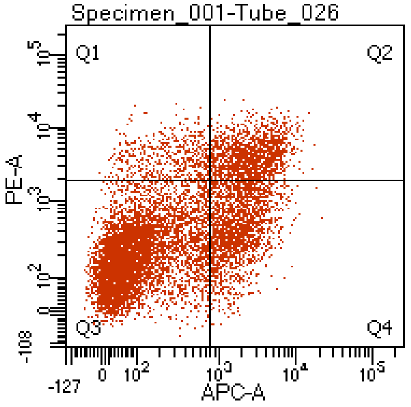

Tube: Tube_026

Population	#Events	%Parent	%Total
All Events	30,835	####	100.0
P1	29,213	94.7	94.7
P2	20,025	68.5	64.9
Q1	1,118	5.6	3.6
Q2	3,037	15.2	9.8
Q3	11,995	59.9	38.9
Q4	3,875	19.4	12.6

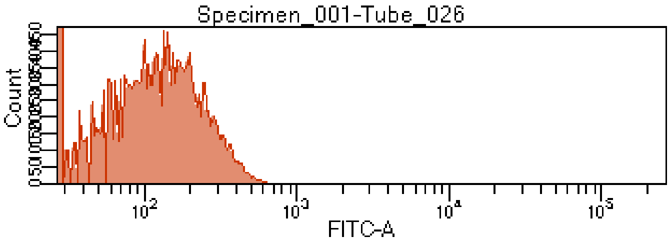

Tube Name: Tube_026
GUID: 88daf8b8-e4a0-4c7b-a56e-70bc39338483

Population	#Events	%Parent	PE-A Mean	PE-A %CV	APC-A Mean	APC-A %CV	APC-Cy7-A Mean	APC-Cy7-A %CV
All Events	30,835	####	1,006	208.1	773	182.9	458	190.3
P1	29,213	94.7	983	199.6	783	171.4	464	176.7
P2	20,025	68.5	1,282	173.2	950	160.6	565	165.0
Q1	1,118	5.6	4,658	59.7	337	65.6	201	67.8
Q2	3,037	15.2	4,817	58.2	3,060	68.4	1,857	70.5
Q3	11,995	59.9	286	108.7	135	130.2	75	139.5
Q4	3,875	19.4	622	76.3	1,996	66.2	1,175	68.9
