# Supplementary material for: Salvia chinensis Benth Inhibits Triple-Negative Breast Cancer Progression by Inducing the DNA Damage Pathway
Source: Front Oncol. 2022 Aug 10;12:882784. doi: 10.3389/fonc.2022.882784 (PMC9404549; doi:10.3389/fonc.2022.882784)
Supplement: Supplementary file 18 [file DataSheet_11.zip › other raw data/figure 4a/18.HCC1187-Q(50uM)-3.pdf]

BD FACSDiva 8.0.1

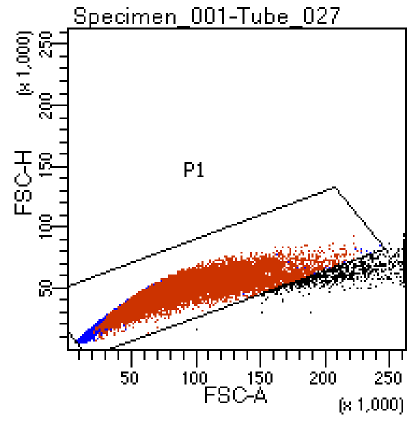

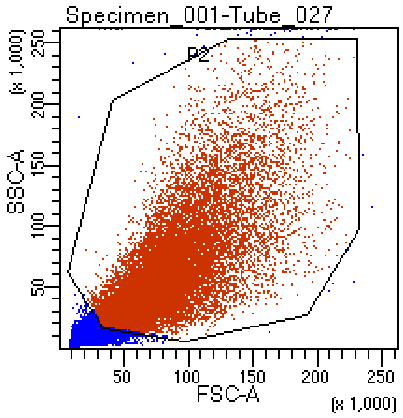

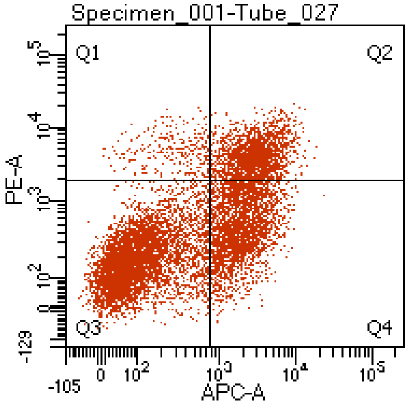

Tube: Tube_027

Population	#Events	%Parent	%Total
All Events	29,996	####	100.0
P1	28,418	94.7	94.7
P2	20,038	70.5	66.8
Q1	628	3.1	2.1
Q2	3,771	18.8	12.6
Q3	10,313	51.5	34.4
Q4	5,326	26.6	17.8

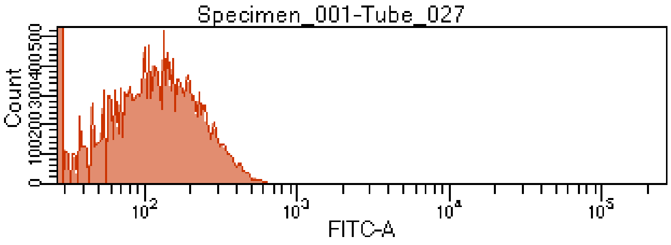

Tube Name: Tube_027
GUID: 10c1b22a-531f-42fd-940b-899d2bac48c0

Population	#Events	%Parent	PE-A Mean	PE-A %CV	APC-A Mean	APC-A %CV	APC-Cy7-A Mean	APC-Cy7-A %CV
All Events	29,996	####	1,071	207.0	1,090	150.8	668	155.9
P1	28,418	94.7	1,030	194.6	1,099	144.8	673	149.5
P2	20,038	70.5	1,332	169.5	1,319	134.7	810	138.8
Q1	628	3.1	5,219	57.5	362	62.0	218	63.9
Q2	3,771	18.8	4,680	58.0	3,422	61.4	2,143	63.6
Q3	10,313	51.5	237	105.6	148	118.9	82	127.5
Q4	5,326	26.6	621	76.6	2,212	64.0	1,344	66.5
