# Supplementary material for: Salvia chinensis Benth Inhibits Triple-Negative Breast Cancer Progression by Inducing the DNA Damage Pathway
Source: Front Oncol. 2022 Aug 10;12:882784. doi: 10.3389/fonc.2022.882784 (PMC9404549; doi:10.3389/fonc.2022.882784)
Supplement: Supplementary file 18 [file DataSheet_11.zip › other raw data/figure 4a/19.HCC1187-B(50uM)-1.pdf]

BD FACSDiva 8.0.1

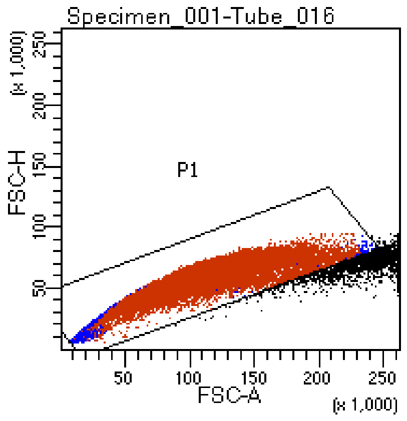

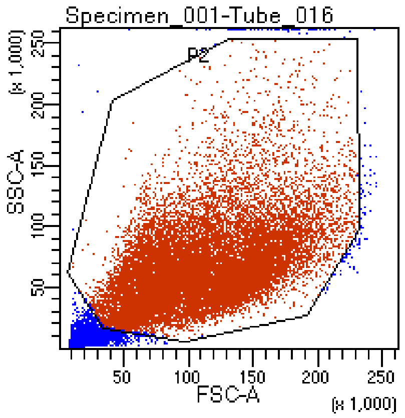

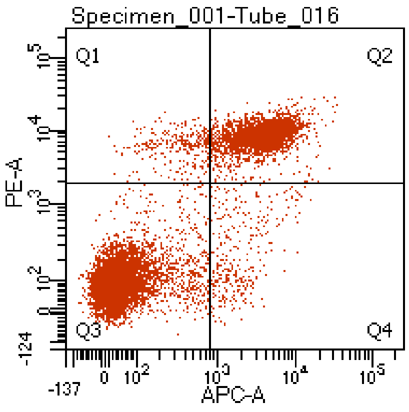

Tube: Tube_016

Population	#Events	%Parent	%Total
All Events	31,374	####	100.0
P1	23,449	74.7	74.7
P2	20,016	85.4	63.8
Q1	680	3.4	2.2
Q2	5,611	28.0	17.9
Q3	13,080	65.3	41.7
Q4	645	3.2	2.1

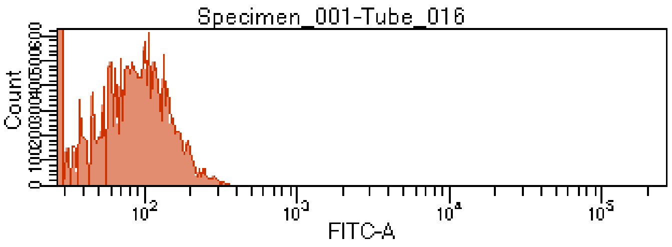

Tube Name: Tube_016
GUID: 334285e4-f955-4ef9-8271-daf83d34c01a

Population	#Events	%Parent	PE-A Mean	PE-A %CV	APC-A Mean	APC-A %CV	APC-Cy7-A Mean	APC-Cy7-A %CV
All Events	31,374	####	2,248	193.8	1,091	246.2	637	258.7
P1	23,449	74.7	2,451	168.1	1,223	198.2	717	205.3
P2	20,016	85.4	2,776	155.8	1,321	192.5	773	199.4
Q1	680	3.4	6,830	41.8	406	50.4	228	53.2
Q2	5,611	28.0	8,767	37.2	4,247	73.2	2,501	77.5
Q3	13,080	65.3	111	117.1	53	199.0	26	232.2
Q4	645	3.2	440	111.7	2,542	103.7	1,456	116.5
