# Supplementary material for: Salvia chinensis Benth Inhibits Triple-Negative Breast Cancer Progression by Inducing the DNA Damage Pathway
Source: Front Oncol. 2022 Aug 10;12:882784. doi: 10.3389/fonc.2022.882784 (PMC9404549; doi:10.3389/fonc.2022.882784)
Supplement: Supplementary file 18 [file DataSheet_11.zip › other raw data/figure 4a/2.231-V2.pdf]

BD FACSDiva 8.0.1

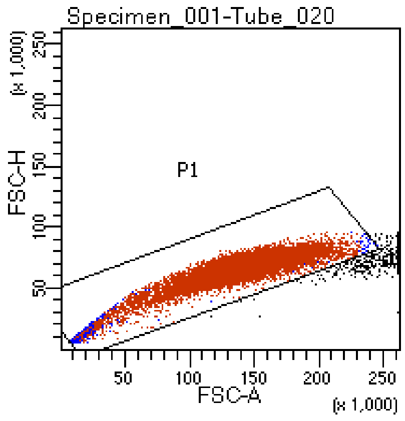

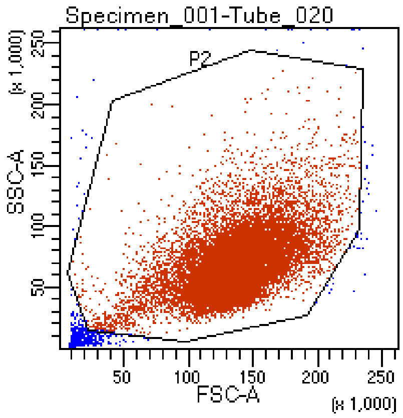

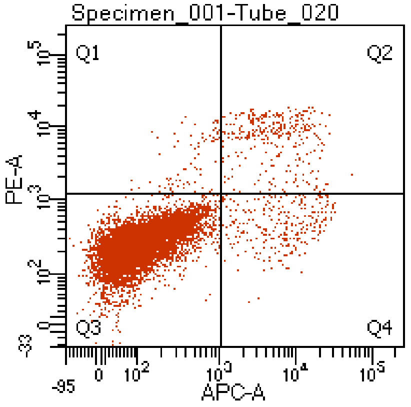

Tube: Tube_020

Population	#Events	%Parent	%Total
All Events	21,722	####	100.0
P1	20,847	96.0	96.0
P2	20,071	96.3	92.4
Q1	149	0.7	0.7
Q2	588	2.9	2.7
Q3	18,746	93.4	86.3
Q4	588	2.9	2.7

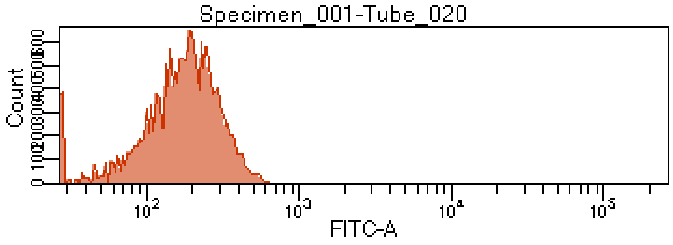

Tube Name: Tube_020
GUID: e93c7b37-fe30-471b-bdc6-38b9fa0a2e0a

Population	#Events	%Parent	PE-A Mean	PE-A %CV	APC-A Mean	APC-A %CV	APC-Cy7-A Mean	APC-Cy7-A %CV
All Events	21,722	####	576	303.4	660	423.9	384	453.3
P1	20,847	96.0	564	302.7	636	401.0	370	428.9
P2	20,071	96.3	572	297.3	627	409.6	365	438.4
Q1	149	0.7	4,798	71.1	533	56.0	297	58.0
Q2	588	2.9	8,673	54.4	7,373	89.4	4,373	94.2
Q3	18,746	93.4	285	50.2	152	90.3	78	101.1
Q4	588	2.9	534	55.1	9,040	84.0	5,510	90.8
