# Supplementary material for: Salvia chinensis Benth Inhibits Triple-Negative Breast Cancer Progression by Inducing the DNA Damage Pathway
Source: Front Oncol. 2022 Aug 10;12:882784. doi: 10.3389/fonc.2022.882784 (PMC9404549; doi:10.3389/fonc.2022.882784)
Supplement: Supplementary file 18 [file DataSheet_11.zip › other raw data/figure 4a/20.HCC1187-B(50uM)-2.pdf]

BD FACSDiva 8.0.1

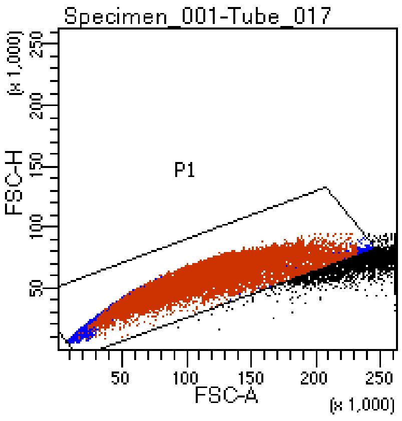

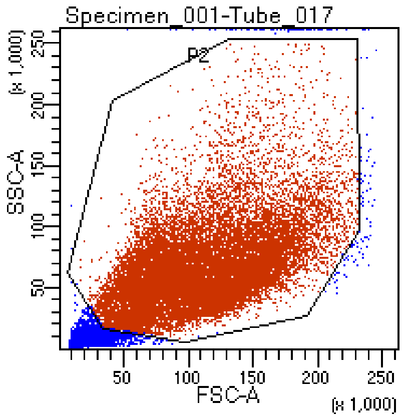

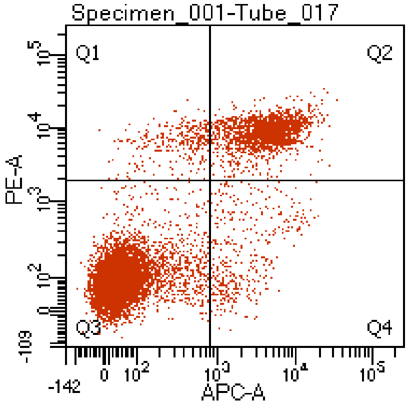

Tube: Tube_017

Population	#Events	%Parent	%Total
All Events	32,099	####	100.0
P1	23,390	72.9	72.9
P2	20,016	85.6	62.4
Q1	619	3.1	1.9
Q2	4,128	20.6	12.9
Q3	14,491	72.4	45.1
Q4	778	3.9	2.4

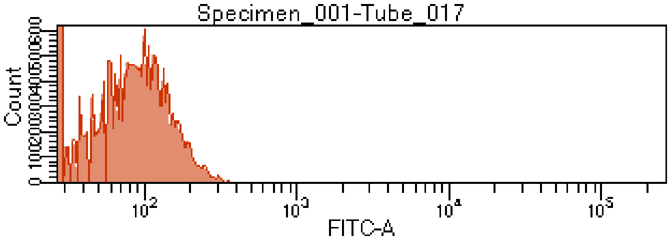

Tube Name: Tube_017
GUID: e017137c-790f-4419-a146-c006abdecf20

Population	#Events	%Parent	PE-A Mean	PE-A %CV	APC-A Mean	APC-A %CV	APC-Cy7-A Mean	APC-Cy7-A %CV
All Events	32,099	####	1,829	227.4	986	265.6	574	279.0
P1	23,390	72.9	1,969	200.3	1,140	226.8	666	234.3
P2	20,016	85.6	2,212	187.5	1,222	222.6	713	229.8
Q1	619	3.1	7,096	47.2	385	53.7	218	56.7
Q2	4,128	20.6	9,217	38.8	5,133	71.1	3,016	75.1
Q3	14,491	72.4	107	115.8	50	201.2	25	236.2
Q4	778	3.9	367	115.0	2,972	103.3	1,716	111.8
