# Supplementary material for: Salvia chinensis Benth Inhibits Triple-Negative Breast Cancer Progression by Inducing the DNA Damage Pathway
Source: Front Oncol. 2022 Aug 10;12:882784. doi: 10.3389/fonc.2022.882784 (PMC9404549; doi:10.3389/fonc.2022.882784)
Supplement: Supplementary file 18 [file DataSheet_11.zip › other raw data/figure 4a/21.HCC1187-B(50uM)-3.pdf]

BD FACSDiva 8.0.1

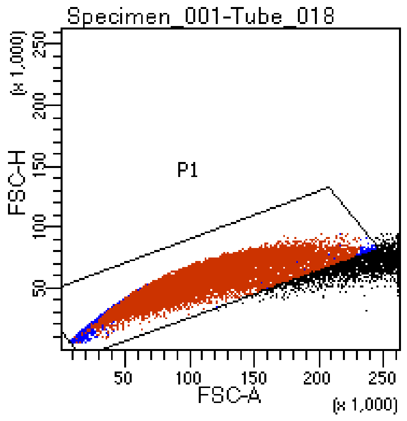

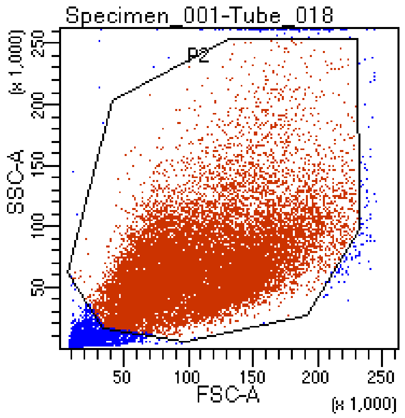

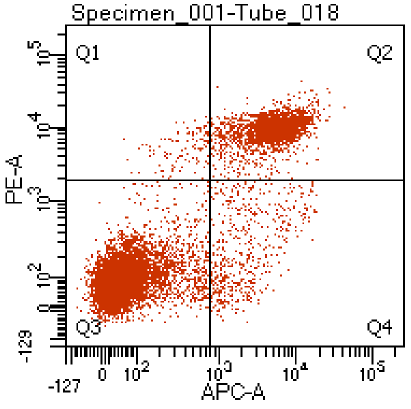

Tube: Tube_018

Population	#Events	%Parent	%Total
All Events	32,369	####	100.0
P1	23,238	71.8	71.8
P2	20,023	86.2	61.9
Q1	193	1.0	0.6
Q2	4,758	23.8	14.7
Q3	14,031	70.1	43.3
Q4	1,041	5.2	3.2

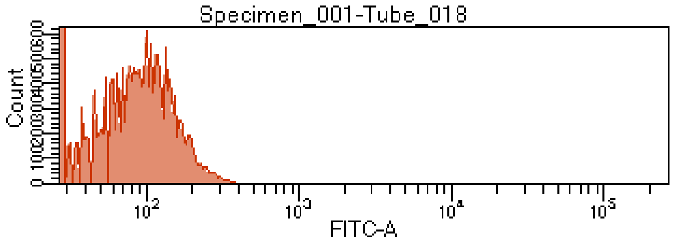

Tube Name: Tube_018
GUID: 25218b8c-40eb-415b-997d-40e7da77d0f9

Population	#Events	%Parent	PE-A Mean	PE-A %CV	APC-A Mean	APC-A %CV	APC-Cy7-A Mean	APC-Cy7-A %CV
All Events	32,369	####	2,007	224.8	1,322	236.5	774	246.0
P1	23,238	71.8	2,236	196.4	1,564	204.1	922	210.0
P2	20,023	86.2	2,506	183.3	1,692	198.3	997	204.0
Q1	193	1.0	6,070	60.1	438	47.3	259	49.8
Q2	4,758	23.8	9,890	39.4	6,218	64.9	3,689	68.3
Q3	14,031	70.1	109	112.9	71	162.9	35	185.0
Q4	1,041	5.2	404	111.5	3,087	104.5	1,788	114.5
