# Supplementary material for: Salvia chinensis Benth Inhibits Triple-Negative Breast Cancer Progression by Inducing the DNA Damage Pathway
Source: Front Oncol. 2022 Aug 10;12:882784. doi: 10.3389/fonc.2022.882784 (PMC9404549; doi:10.3389/fonc.2022.882784)
Supplement: Supplementary file 18 [file DataSheet_11.zip › other raw data/figure 4a/22.HCC1187-Combo-1.pdf]

BD FACSDiva 8.0.1

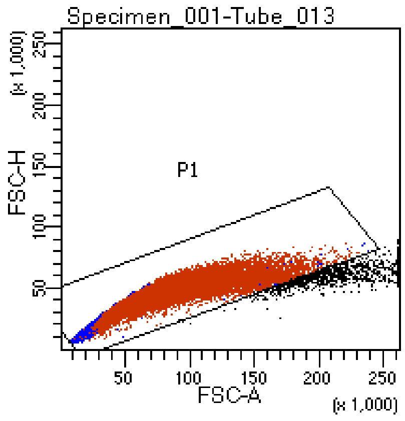

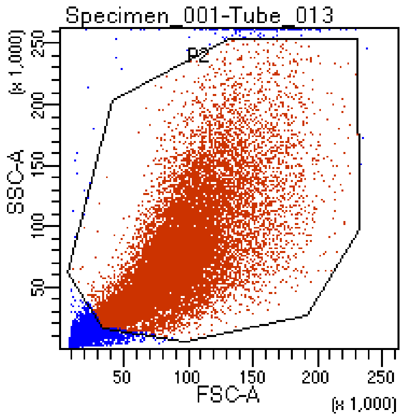

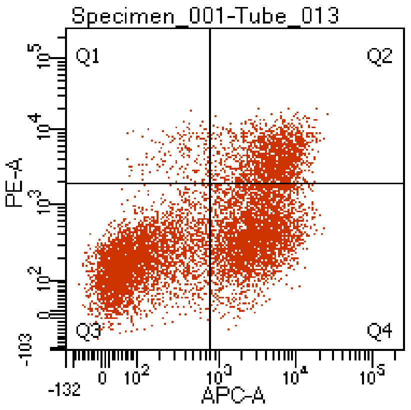

Tube: Tube_013

Population	#Events	%Parent	%Total
All Events	27,139	####	100.0
P1	25,462	93.8	93.8
P2	20,053	78.8	73.9
Q1	347	1.7	1.3
Q2	3,431	17.1	12.6
Q3	8,906	44.4	32.8
Q4	7,369	36.7	27.2

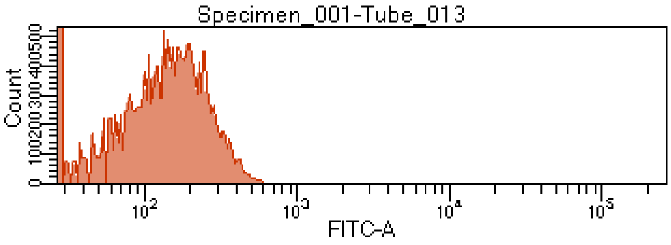

Tube Name: Tube_013
GUID: 300bf66f-51e3-42b3-ac8f-b66e26ba05fe

Population	#Events	%Parent	PE-A Mean	PE-A %CV	APC-A Mean	APC-A %CV	APC-Cy7-A Mean	APC-Cy7-A %CV
All Events	27,139	####	1,081	210.6	2,005	145.2	1,217	148.9
P1	25,462	93.8	1,069	201.9	2,064	139.8	1,252	143.3
P2	20,053	78.8	1,248	181.9	2,360	130.7	1,433	134.0
Q1	347	1.7	5,549	56.8	398	50.0	246	51.6
Q2	3,431	17.1	4,999	59.5	5,680	62.1	3,522	64.0
Q3	8,906	44.4	223	95.5	133	127.4	73	137.8
Q4	7,369	36.7	537	80.4	3,599	74.7	2,158	77.4
