# Supplementary material for: Salvia chinensis Benth Inhibits Triple-Negative Breast Cancer Progression by Inducing the DNA Damage Pathway
Source: Front Oncol. 2022 Aug 10;12:882784. doi: 10.3389/fonc.2022.882784 (PMC9404549; doi:10.3389/fonc.2022.882784)
Supplement: Supplementary file 18 [file DataSheet_11.zip › other raw data/figure 4a/23.HCC1187-Combo-2.pdf]

# BD FACSDiva 8.0.1

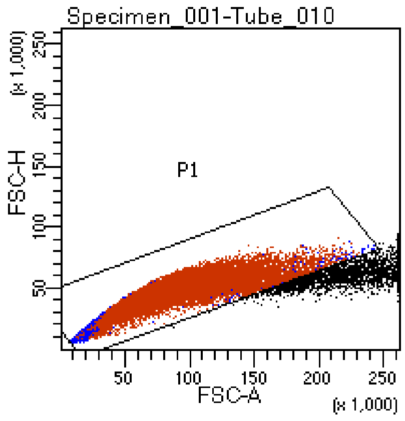


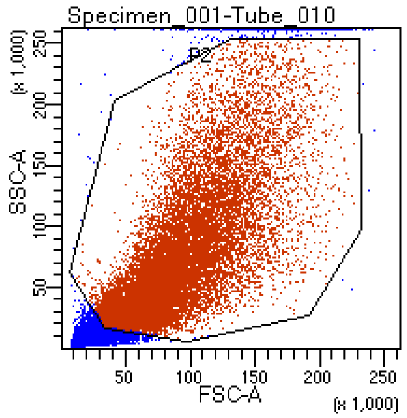


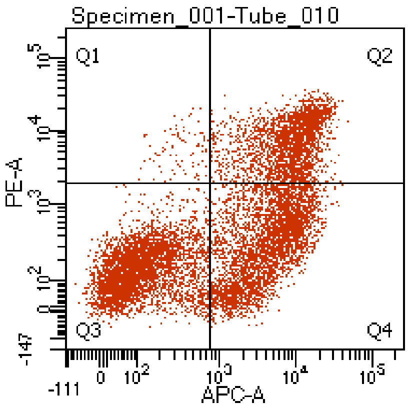


Tube: Tube_010

| Population | #Events | %Parent | %Total |
|---|---|---|---|
| All Events | 34,050 | #### | 100.0 |
| P1 | 29,338 | 86.2 | 86.2 |
| P2 | 20,124 | 68.6 | 59.1 |
| Q1 | 178 | 0.9 | 0.5 |
| Q2 | 4,836 | 24.0 | 14.2 |
| Q3 | 7,731 | 38.4 | 22.7 |
| Q4 | 7,379 | 36.7 | 21.7 |

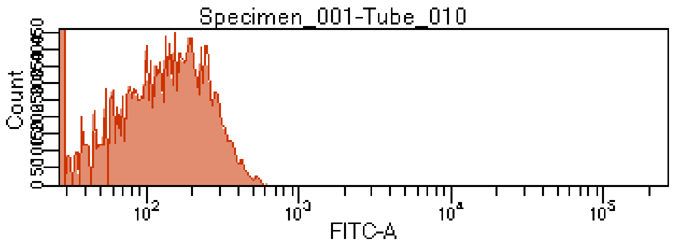


Tube Name: Tube_010
GUID: 77333fce-d5ce-400c-ad2a-6b5201bd811f

| Population | #Events | %Parent | PE-A Mean | PE-A %CV | APC-A Mean | APC-A %CV | APC-Cy7-A Mean | APC-Cy7-A %CV |
|---|---|---|---|---|---|---|---|---|
| All Events | 34,050 | #### | 1,883 | 248.2 | 3,361 | 160.9 | 2,015 | 166.8 |
| P1 | 29,338 | 86.2 | 1,840 | 233.4 | 3,591 | 147.2 | 2,158 | 152.5 |
| P2 | 20,124 | 68.6 | 2,459 | 197.8 | 4,589 | 127.5 | 2,765 | 132.2 |
| Q1 | 178 | 0.9 | 6,088 | 66.3 | 426 | 48.2 | 236 | 49.7 |
| Q2 | 4,836 | 24.0 | 9,010 | 69.9 | 10,497 | 63.8 | 6,478 | 66.1 |
| Q3 | 7,731 | 38.4 | 183 | 98.2 | 157 | 109.4 | 78 | 116.3 |
| Q4 | 7,379 | 36.7 | 462 | 93.5 | 5,461 | 80.5 | 3,207 | 85.1 |
