# Supplementary material for: Salvia chinensis Benth Inhibits Triple-Negative Breast Cancer Progression by Inducing the DNA Damage Pathway
Source: Front Oncol. 2022 Aug 10;12:882784. doi: 10.3389/fonc.2022.882784 (PMC9404549; doi:10.3389/fonc.2022.882784)
Supplement: Supplementary file 18 [file DataSheet_11.zip › other raw data/figure 4a/24.HCC1187-Combo-3.pdf]

BD FACSDiva 8.0.1

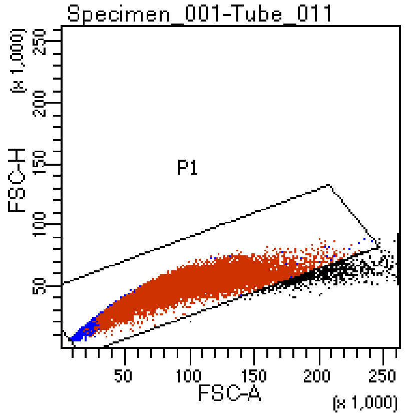


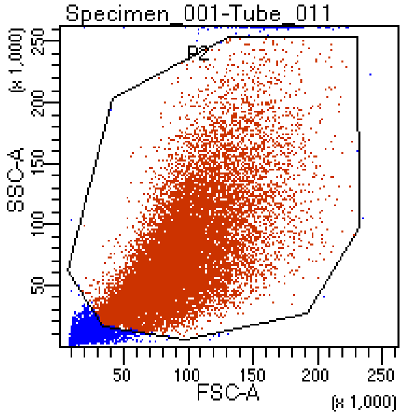


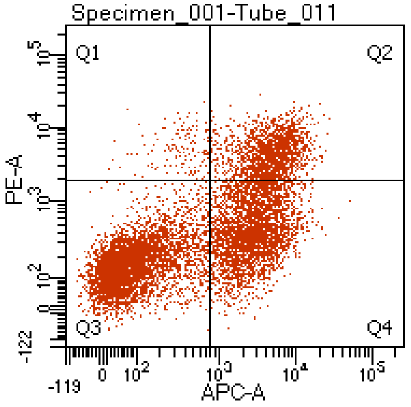


Tube: Tube_011

| Population | #Events | %Parent | %Total |
|---|---|---|---|
| All Events | 26,336 | #### | 100.0 |
| P1 | 25,018 | 95.0 | 95.0 |
| P2 | 20,049 | 80.1 | 76.1 |
| Q1 | 281 | 1.4 | 1.1 |
| Q2 | 3,520 | 17.6 | 13.4 |
| Q3 | 9,625 | 48.0 | 36.5 |
| Q4 | 6,623 | 33.0 | 25.1 |

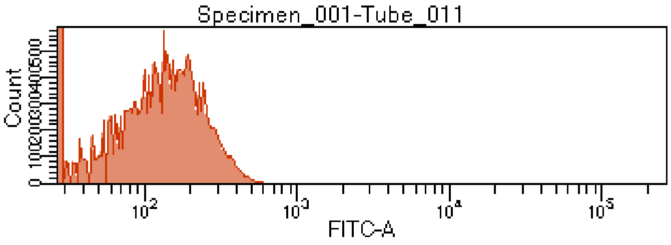


Tube Name: Tube_011
GUID: ab0c0845-aeae-4ba9-aac5-b09bdca74bec

| Population | #Events | %Parent | PE-A Mean | PE-A %CV | APC-A Mean | APC-A %CV | APC-Cy7-A Mean | APC-Cy7-A %CV |
|---|---|---|---|---|---|---|---|---|
| All Events | 26,336 | #### | 1,105 | 212.8 | 1,889 | 154.1 | 1,144 | 157.5 |
| P1 | 25,018 | 95.0 | 1,101 | 208.4 | 1,915 | 148.6 | 1,160 | 152.0 |
| P2 | 20,049 | 80.1 | 1,251 | 188.0 | 2,139 | 141.4 | 1,296 | 144.5 |
| Q1 | 281 | 1.4 | 5,862 | 63.1 | 393 | 51.1 | 237 | 53.7 |
| Q2 | 3,520 | 17.6 | 5,103 | 60.2 | 5,562 | 65.5 | 3,443 | 66.8 |
| Q3 | 9,625 | 48.0 | 196 | 99.9 | 120 | 133.0 | 66 | 142.5 |
| Q4 | 6,623 | 33.0 | 541 | 79.2 | 3,328 | 79.0 | 1,987 | 81.5 |
