# Supplementary material for: Salvia chinensis Benth Inhibits Triple-Negative Breast Cancer Progression by Inducing the DNA Damage Pathway
Source: Front Oncol. 2022 Aug 10;12:882784. doi: 10.3389/fonc.2022.882784 (PMC9404549; doi:10.3389/fonc.2022.882784)
Supplement: Supplementary file 18 [file DataSheet_11.zip › other raw data/figure 4a/25.4T1-V1.pdf]

BD FACSDiva 8.0.1

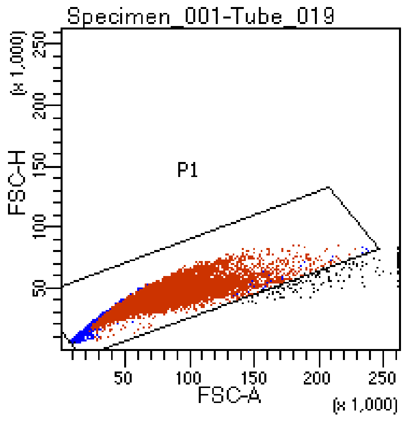

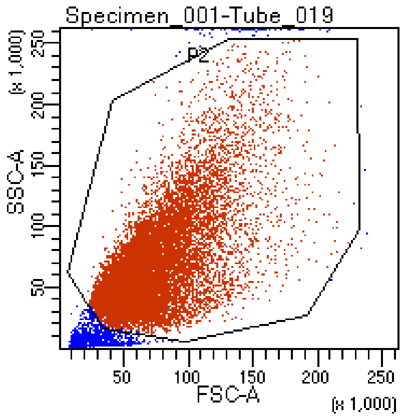

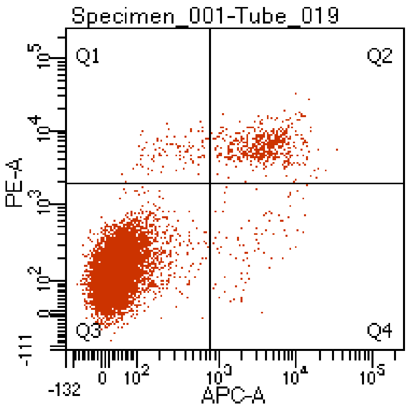

Tube: Tube_019

Population	#Events	%Parent	%Total
All Events	21,934	####	100.0
P1	21,669	98.8	98.8
P2	20,023	92.4	91.3
Q1	203	1.0	0.9
Q2	1,083	5.4	4.9
Q3	18,555	92.7	84.6
Q4	182	0.9	0.8

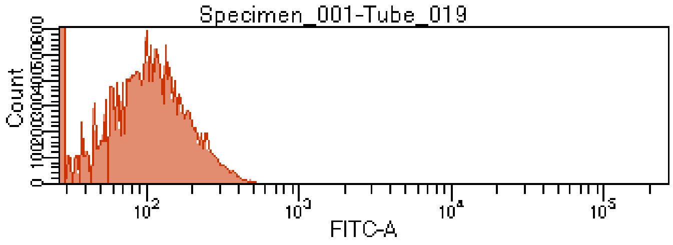

Tube Name: Tube_019

GUID: 69faa2b7-fca8-40cb-ba13-26e7fbd2e8d0

Population	#Events	%Parent	PE-A Mean	PE-A %CV	APC-A Mean	APC-A %CV	APC-Cy7-A Mean	APC-Cy7-A %CV
All Events	21,934	####	562	330.7	348	457.1	211	480.0
P1	21,669	98.8	543	314.6	337	420.2	204	437.7
P2	20,023	92.4	560	308.6	321	436.1	193	454.4
Q1	203	1.0	5,667	41.8	339	55.7	200	60.3
Q2	1,083	5.4	6,618	46.4	4,707	72.7	2,877	77.4
Q3	18,555	92.7	151	78.4	32	187.8	16	248.6
Q4	182	0.9	490	98.1	3,711	90.0	2,275	96.3
