# Supplementary material for: Salvia chinensis Benth Inhibits Triple-Negative Breast Cancer Progression by Inducing the DNA Damage Pathway
Source: Front Oncol. 2022 Aug 10;12:882784. doi: 10.3389/fonc.2022.882784 (PMC9404549; doi:10.3389/fonc.2022.882784)
Supplement: Supplementary file 18 [file DataSheet_11.zip › other raw data/figure 4a/26.4T1-V2.pdf]

BD FACSDiva 8.0.1

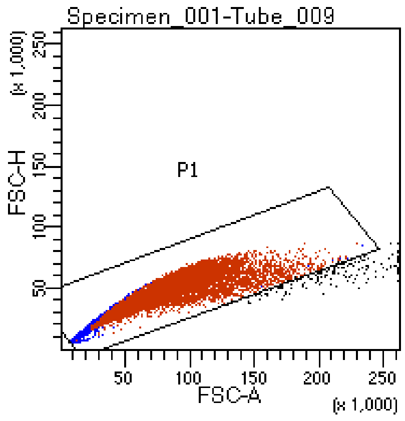

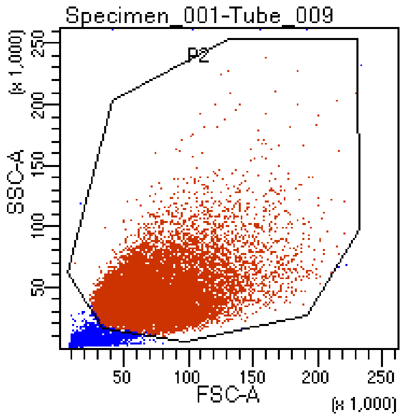

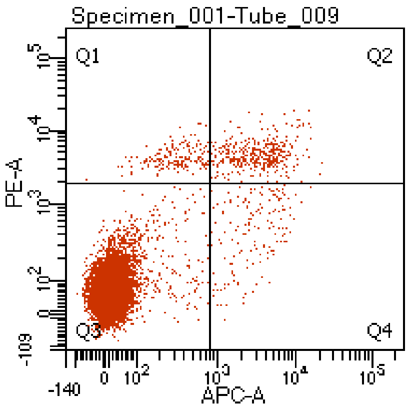

Tube: Tube_009

Population	#Events	%Parent	%Total
All Events	21,965	####	100.0
P1	21,712	98.8	98.8
P2	20,000	92.1	91.1
Q1	341	1.7	1.6
Q2	729	3.6	3.3
Q3	18,620	93.1	84.8
Q4	310	1.6	1.4

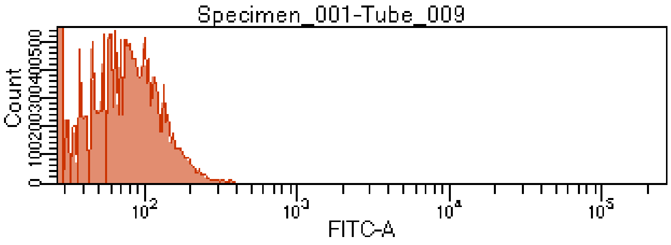

Tube Name: Tube_009
GUID: a0ce9e1c-b473-4781-a19f-39213b5f3391

Population	#Events	%Parent	PE-A Mean	PE-A %CV	APC-A Mean	APC-A %CV	APC-Cy7-A Mean	APC-Cy7-A %CV
All Events	21,965	####	340	366.6	256	446.3	152	458.6
P1	21,712	98.8	332	352.9	250	444.3	149	456.1
P2	20,000	92.1	343	351.8	221	494.0	131	507.3
Q1	341	1.7	4,497	40.1	361	54.1	225	55.3
Q2	729	3.6	5,051	48.4	4,093	74.7	2,466	77.4
Q3	18,620	93.1	77	122.4	12	460.0	6	672.7
Q4	310	1.6	643	77.4	3,494	70.2	2,070	73.6
