# Supplementary material for: Salvia chinensis Benth Inhibits Triple-Negative Breast Cancer Progression by Inducing the DNA Damage Pathway
Source: Front Oncol. 2022 Aug 10;12:882784. doi: 10.3389/fonc.2022.882784 (PMC9404549; doi:10.3389/fonc.2022.882784)
Supplement: Supplementary file 18 [file DataSheet_11.zip › other raw data/figure 4a/27.4T1-V3.pdf]

BD FACSDiva 8.0.1

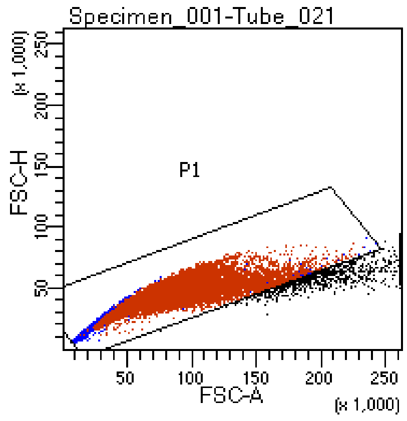

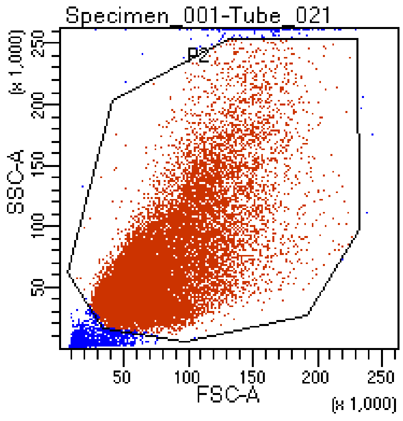

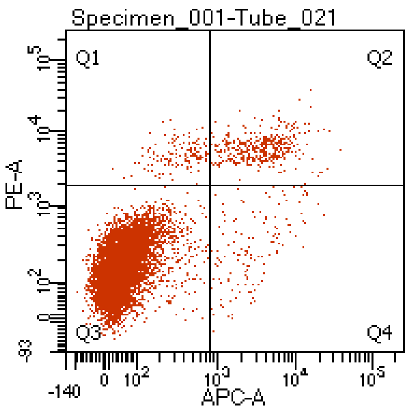

Tube: Tube_021

Population	#Events	%Parent	%Total
All Events	22,841	####	100.0
P1	21,846	95.6	95.6
P2	20,063	91.8	87.8
Q1	298	1.5	1.3
Q2	830	4.1	3.6
Q3	18,709	93.3	81.9
Q4	226	1.1	1.0

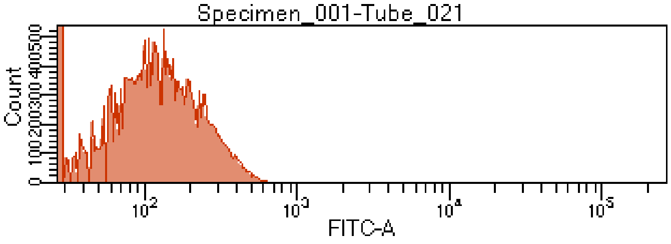

Tube Name: Tube_021
GUID: 69372b86-c68d-425c-b2ca-dac3421ffbd1

Population	#Events	%Parent	PE-A Mean	PE-A %CV	APC-A Mean	APC-A %CV	APC-Cy7-A Mean	APC-Cy7-A %CV
All Events	22,841	####	562	291.4	313	442.8	184	466.0
P1	21,846	95.6	526	294.6	304	441.0	179	463.0
P2	20,063	91.8	538	295.1	282	468.6	165	492.6
Q1	298	1.5	5,407	41.9	398	49.8	239	52.2
Q2	830	4.1	6,670	46.6	4,674	82.5	2,787	87.9
Q3	18,709	93.3	189	78.8	44	148.3	23	187.0
Q4	226	1.1	474	95.0	3,636	98.9	2,182	105.1
