# Supplementary material for: Salvia chinensis Benth Inhibits Triple-Negative Breast Cancer Progression by Inducing the DNA Damage Pathway
Source: Front Oncol. 2022 Aug 10;12:882784. doi: 10.3389/fonc.2022.882784 (PMC9404549; doi:10.3389/fonc.2022.882784)
Supplement: Supplementary file 18 [file DataSheet_11.zip › other raw data/figure 4a/28.4T1-Q(50uM)-1.pdf]

BD FACSDiva 8.0.1

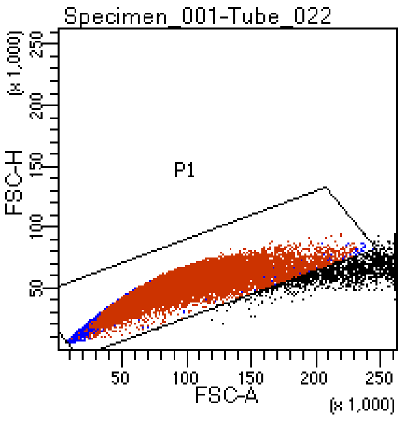

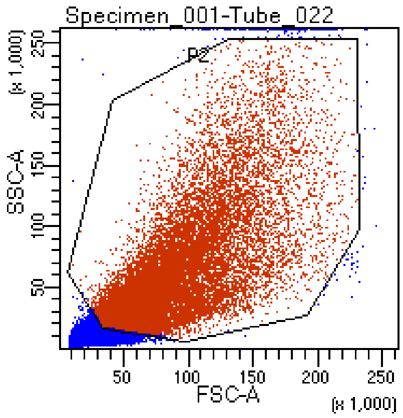

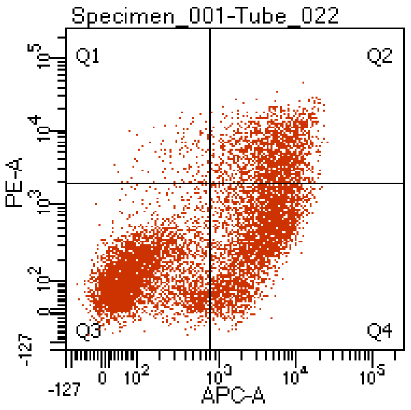

Tube: Tube_022

Population	#Events	%Parent	%Total
All Events	40,016	####	100.0
P1	35,814	89.5	89.5
P2	20,061	56.0	50.1
Q1	234	1.2	0.6
Q2	3,660	18.2	9.1
Q3	8,818	44.0	22.0
Q4	7,349	36.6	18.4

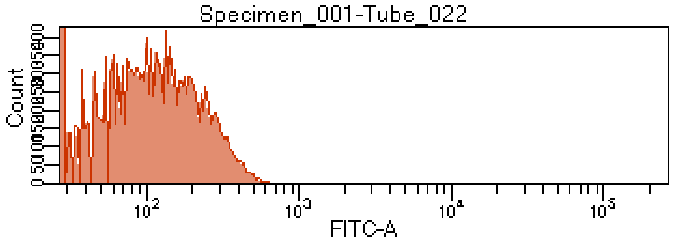

Tube Name: Tube_022

GUID: 3fbf61fb-41d4-4006-b38d-c6e86a268173

Population	#Events	%Parent	PE-A Mean	PE-A %CV	APC-A Mean	APC-A %CV	APC-Cy7-A Mean	APC-Cy7-A %CV
All Events	40,016	####	1,051	279.3	1,852	163.0	1,133	169.5
P1	35,814	89.5	979	263.3	1,928	151.3	1,182	157.4
P2	20,061	56.0	1,516	213.1	2,714	128.2	1,673	133.0
Q1	234	1.2	5,711	69.5	418	46.5	239	49.0
Q2	3,660	18.2	6,458	75.6	6,563	62.9	4,150	66.0
Q3	8,818	44.0	158	125.7	147	123.6	76	130.9
Q4	7,349	36.6	551	84.9	3,949	69.4	2,400	72.8
