# Supplementary material for: Salvia chinensis Benth Inhibits Triple-Negative Breast Cancer Progression by Inducing the DNA Damage Pathway
Source: Front Oncol. 2022 Aug 10;12:882784. doi: 10.3389/fonc.2022.882784 (PMC9404549; doi:10.3389/fonc.2022.882784)
Supplement: Supplementary file 18 [file DataSheet_11.zip › other raw data/figure 4a/29.4T1-Q(50uM)-2.pdf]

BD FACSDiva 8.0.1

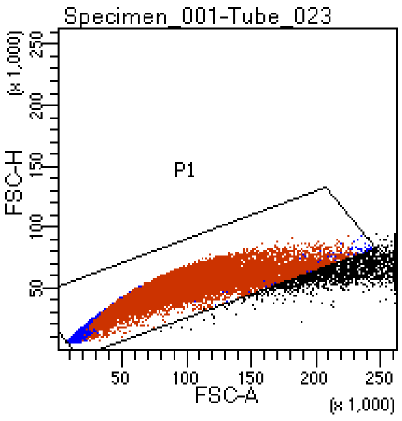

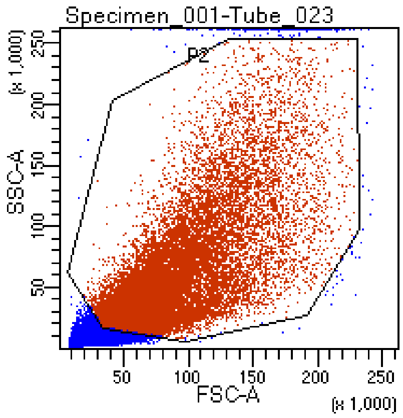

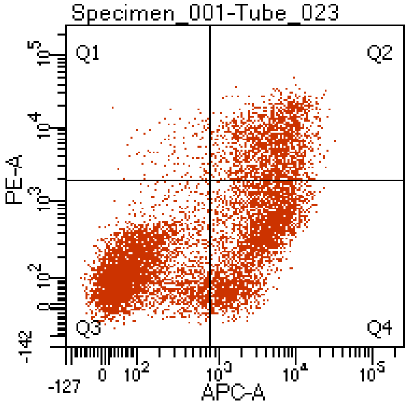

Tube: Tube_023

Population	#Events	%Parent	%Total
All Events	40,703	####	100.0
P1	36,129	88.8	88.8
P2	20,058	55.5	49.3
Q1	235	1.2	0.6
Q2	3,552	17.7	8.7
Q3	10,007	49.9	24.6
Q4	6,264	31.2	15.4

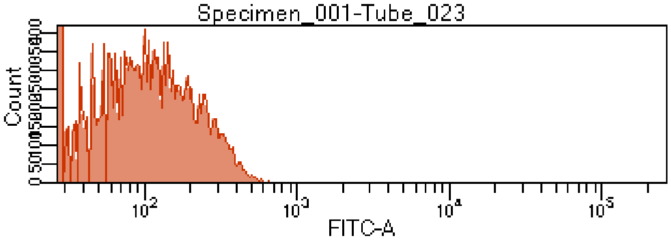

Tube Name: Tube_023
GUID: d491846c-57b8-448c-bda1-2d71bc414ac4

Population	#Events	%Parent	PE-A Mean	PE-A %CV	APC-A Mean	APC-A %CV	APC-Cy7-A Mean	APC-Cy7-A %CV
All Events	40,703	####	1,214	300.0	1,663	185.6	1,018	193.7
P1	36,129	88.8	1,115	288.4	1,712	165.3	1,050	171.6
P2	20,058	55.5	1,757	232.9	2,393	144.0	1,474	148.9
Q1	235	1.2	6,157	65.9	418	50.9	242	52.9
Q2	3,552	17.7	8,161	78.3	6,242	69.4	3,919	72.3
Q3	10,007	49.9	155	121.3	125	137.2	65	146.1
Q4	6,264	31.2	521	89.4	3,906	73.7	2,384	77.5
