# Supplementary material for: Salvia chinensis Benth Inhibits Triple-Negative Breast Cancer Progression by Inducing the DNA Damage Pathway
Source: Front Oncol. 2022 Aug 10;12:882784. doi: 10.3389/fonc.2022.882784 (PMC9404549; doi:10.3389/fonc.2022.882784)
Supplement: Supplementary file 18 [file DataSheet_11.zip › other raw data/figure 4a/3.231-V3.pdf]

BD FACSDiva 8.0.1

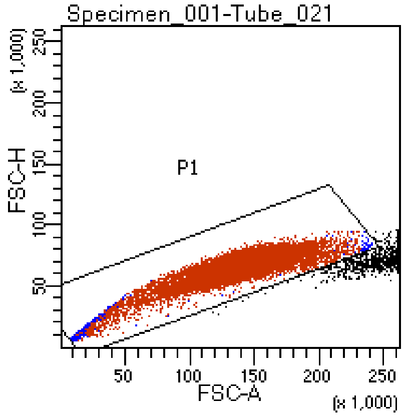

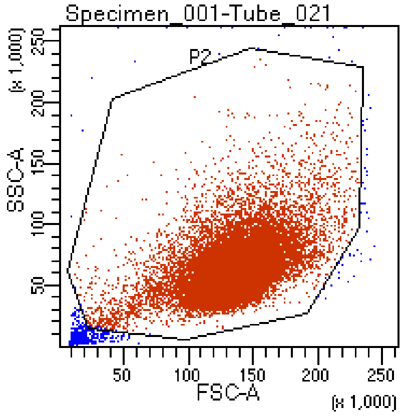

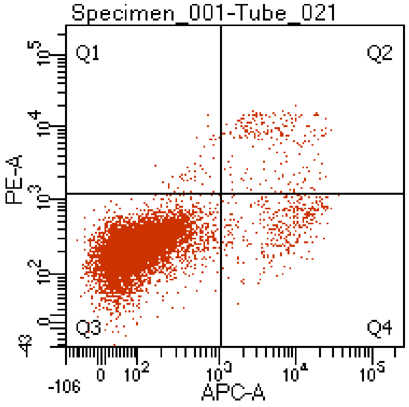

Tube: Tube_021

Population	#Events	%Parent	%Total
All Events	22,456	####	100.0
P1	20,747	92.4	92.4
P2	20,072	96.7	89.4
Q1	97	0.5	0.4
Q2	449	2.2	2.0
Q3	18,810	93.7	83.8
Q4	716	3.6	3.2

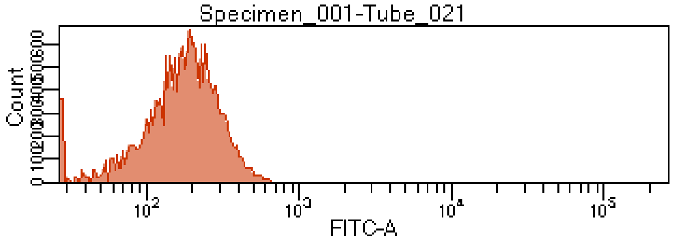

Tube Name: Tube_021
GUID: 7ee11e60-ed6f-46a4-a81a-2eed86406764

Population	#Events	%Parent	PE-A Mean	PE-A %CV	APC-A Mean	APC-A %CV	APC-Cy7-A Mean	APC-Cy7-A %CV
All Events	22,456	####	477	307.9	619	414.0	348	435.8
P1	20,747	92.4	450	320.2	594	409.2	334	427.7
P2	20,072	96.7	451	313.9	582	415.0	327	433.2
Q1	97	0.5	3,628	78.8	527	41.3	293	44.9
Q2	449	2.2	8,186	59.0	7,628	91.3	4,480	95.3
Q3	18,810	93.7	248	51.4	118	96.7	60	111.7
Q4	716	3.6	514	55.2	8,347	71.7	4,746	74.8
