# Supplementary material for: Salvia chinensis Benth Inhibits Triple-Negative Breast Cancer Progression by Inducing the DNA Damage Pathway
Source: Front Oncol. 2022 Aug 10;12:882784. doi: 10.3389/fonc.2022.882784 (PMC9404549; doi:10.3389/fonc.2022.882784)
Supplement: Supplementary file 18 [file DataSheet_11.zip › other raw data/figure 4a/30.4T1-Q(50uM)-3.pdf]

BD FACSDiva 8.0.1

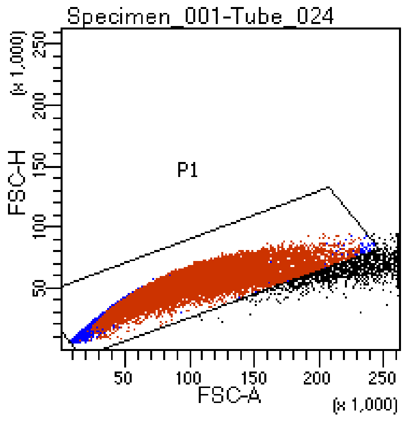

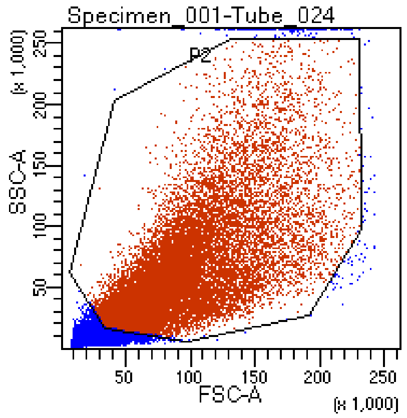

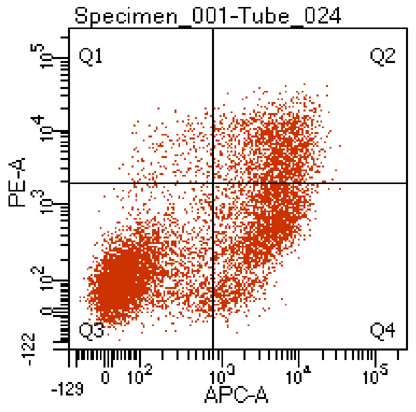

Tube: Tube_024

Population	#Events	%Parent	%Total
All Events	35,534	####	100.0
P1	31,835	89.6	89.6
P2	20,056	63.0	56.4
Q1	389	1.9	1.1
Q2	3,495	17.4	9.8
Q3	10,916	54.4	30.7
Q4	5,256	26.2	14.8

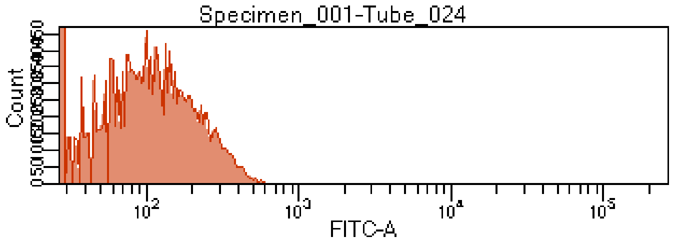

Tube Name: Tube_024
GUID: dee5aa3c-23f2-476e-b47c-bb2fa5ebf495

Population	#Events	%Parent	PE-A Mean	PE-A %CV	APC-A Mean	APC-A %CV	APC-Cy7-A Mean	APC-Cy7-A %CV
All Events	35,534	####	1,223	282.1	1,639	185.7	997	192.3
P1	31,835	89.6	1,184	266.6	1,709	172.2	1,040	178.3
P2	20,056	63.0	1,673	225.4	2,221	154.1	1,357	159.0
Q1	389	1.9	6,439	64.5	355	56.2	201	61.5
Q2	3,495	17.4	7,567	75.8	6,382	68.0	3,987	71.0
Q3	10,916	54.4	144	138.2	96	159.3	50	171.9
Q4	5,256	26.2	577	79.1	4,005	69.9	2,409	72.7
