# Supplementary material for: Salvia chinensis Benth Inhibits Triple-Negative Breast Cancer Progression by Inducing the DNA Damage Pathway
Source: Front Oncol. 2022 Aug 10;12:882784. doi: 10.3389/fonc.2022.882784 (PMC9404549; doi:10.3389/fonc.2022.882784)
Supplement: Supplementary file 18 [file DataSheet_11.zip › other raw data/figure 4a/31.4T1-B(50uM)-1.pdf]

BD FACSDiva 8.0.1

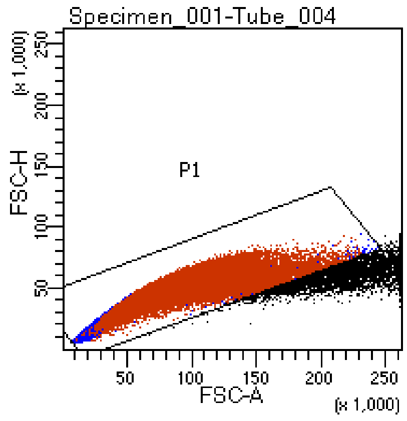

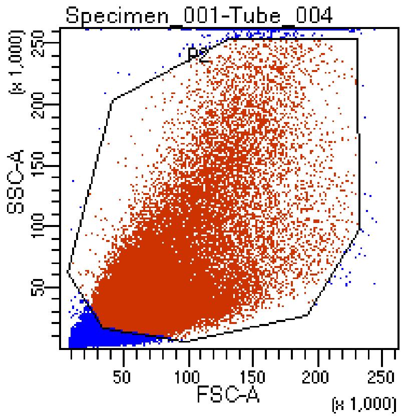

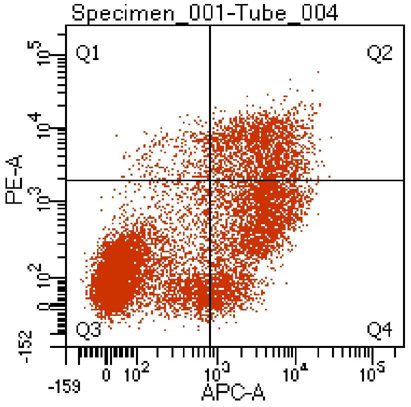

Tube: Tube_004

Population	#Events	%Parent	%Total
All Events	59,342	####	100.0
P1	49,999	84.3	84.3
P2	30,161	60.3	50.8
Q1	618	2.0	1.0
Q2	4,374	14.5	7.4
Q3	18,267	60.6	30.8
Q4	6,902	22.9	11.6

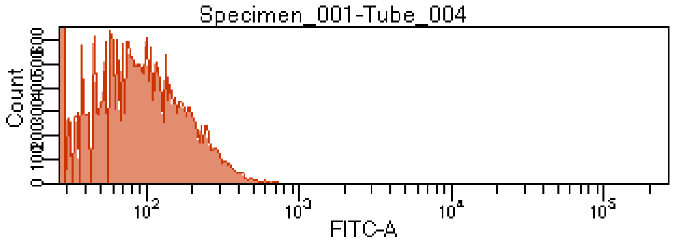

Tube Name: Tube_004
GUID: 46e8c4b2-0c89-401d-9e36-1fa7c65ba7f6

Population	#Events	%Parent	PE-A Mean	PE-A %CV	APC-A Mean	APC-A %CV	APC-Cy7-A Mean	APC-Cy7-A %CV
All Events	59,342	####	1,009	278.9	1,166	202.2	696	211.1
P1	49,999	84.3	876	256.3	1,237	181.2	740	188.8
P2	30,161	60.3	1,223	222.8	1,541	173.7	926	180.3
Q1	618	2.0	4,981	54.3	433	48.6	237	49.2
Q2	4,374	14.5	6,222	66.4	4,706	77.3	2,873	81.2
Q3	18,267	60.6	140	140.2	90	188.3	45	201.9
Q4	6,902	22.9	584	84.0	3,475	76.7	2,085	81.1
