# Supplementary material for: Salvia chinensis Benth Inhibits Triple-Negative Breast Cancer Progression by Inducing the DNA Damage Pathway
Source: Front Oncol. 2022 Aug 10;12:882784. doi: 10.3389/fonc.2022.882784 (PMC9404549; doi:10.3389/fonc.2022.882784)
Supplement: Supplementary file 18 [file DataSheet_11.zip › other raw data/figure 4a/32.4T1-B(50uM)-2.pdf]

BD FACSDiva 8.0.1

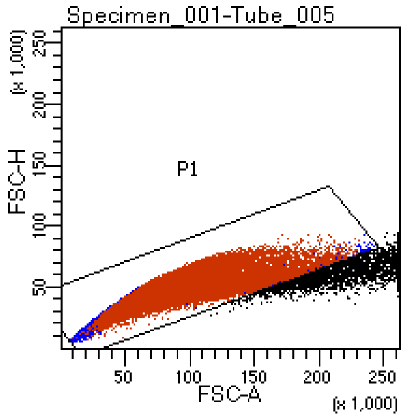

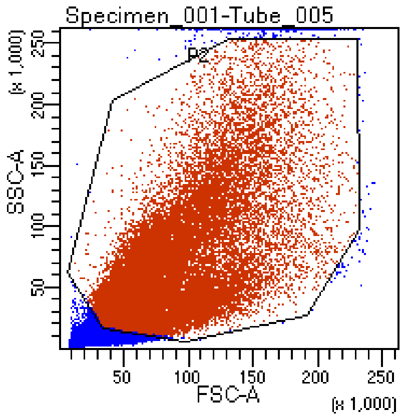

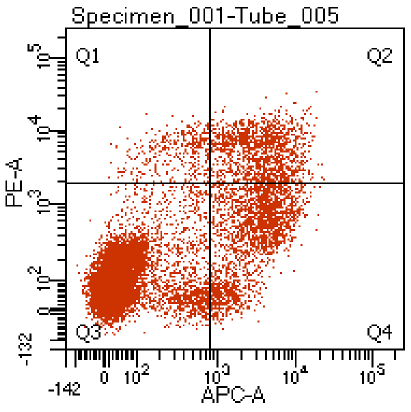

Tube: Tube_005

Population	#Events	%Parent	%Total
All Events	49,470	####	100.0
P1	43,824	88.6	88.6
P2	30,127	68.7	60.9
Q1	720	2.4	1.5
Q2	2,867	9.5	5.8
Q3	22,125	73.4	44.7
Q4	4,415	14.7	8.9

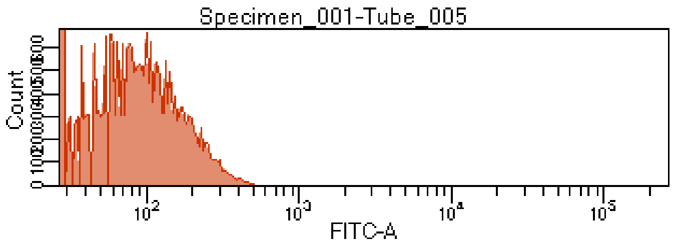

Tube Name: Tube_005
GUID: 90f681c2-19f4-4b73-acd8-ff4b41308929

Population	#Events	%Parent	PE-A Mean	PE-A %CV	APC-A Mean	APC-A %CV	APC-Cy7-A Mean	APC-Cy7-A %CV
All Events	49,470	####	779	296.4	857	233.6	510	243.4
P1	43,824	88.6	727	278.4	898	214.6	534	222.6
P2	30,127	68.7	909	257.3	982	223.6	587	230.6
Q1	720	2.4	5,840	49.7	375	57.0	212	59.9
Q2	2,867	9.5	6,221	63.4	4,540	76.7	2,773	80.0
Q3	22,125	73.4	122	135.0	58	226.4	30	246.8
Q4	4,415	14.7	602	82.1	3,398	75.2	2,024	78.8
