# Supplementary material for: Salvia chinensis Benth Inhibits Triple-Negative Breast Cancer Progression by Inducing the DNA Damage Pathway
Source: Front Oncol. 2022 Aug 10;12:882784. doi: 10.3389/fonc.2022.882784 (PMC9404549; doi:10.3389/fonc.2022.882784)
Supplement: Supplementary file 18 [file DataSheet_11.zip › other raw data/figure 4a/33.4T1-B(50uM)-3.pdf]

BD FACSDiva 8.0.1

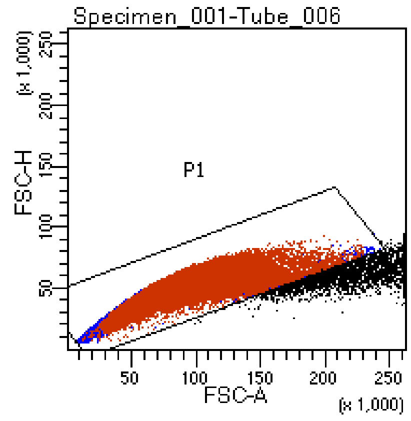

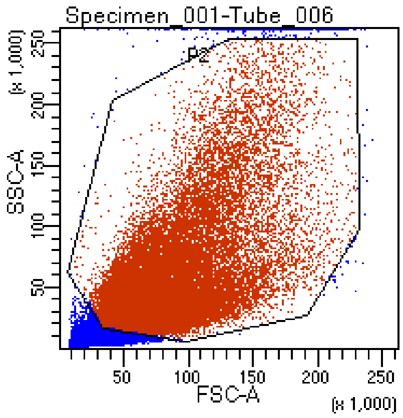

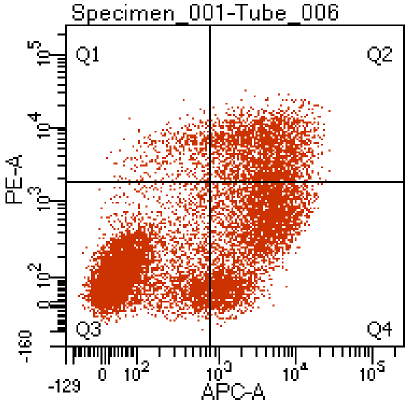

Tube: Tube_006

Population	#Events	%Parent	%Total
All Events	61,173	####	100.0
P1	52,587	86.0	86.0
P2	30,094	57.2	49.2
Q1	855	2.8	1.4
Q2	4,976	16.5	8.1
Q3	15,699	52.2	25.7
Q4	8,564	28.5	14.0

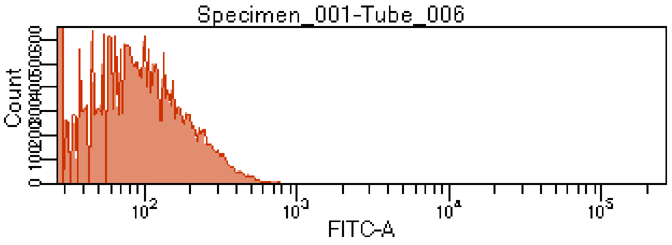

Tube Name: Tube_006
GUID: 1834b8d6-cca9-446b-ae8b-46ee4f6fd81a

Population	#Events	%Parent	PE-A Mean	PE-A %CV	APC-A Mean	APC-A %CV	APC-Cy7-A Mean	APC-Cy7-A %CV
All Events	61,173	####	1,084	288.1	1,383	206.3	830	216.4
P1	52,587	86.0	962	244.1	1,458	174.0	875	181.5
P2	30,094	57.2	1,425	204.4	1,958	156.3	1,184	162.1
Q1	855	2.8	5,649	51.5	411	51.7	237	53.8
Q2	4,976	16.5	6,232	65.5	5,093	78.6	3,151	81.9
Q3	15,699	52.2	150	140.5	112	164.2	56	173.3
Q4	8,564	28.5	547	88.1	3,675	78.8	2,201	83.1
