# Supplementary material for: Salvia chinensis Benth Inhibits Triple-Negative Breast Cancer Progression by Inducing the DNA Damage Pathway
Source: Front Oncol. 2022 Aug 10;12:882784. doi: 10.3389/fonc.2022.882784 (PMC9404549; doi:10.3389/fonc.2022.882784)
Supplement: Supplementary file 18 [file DataSheet_11.zip › other raw data/figure 4a/34.4T1-Combo-1.pdf]

BD FACSDiva 8.0.1

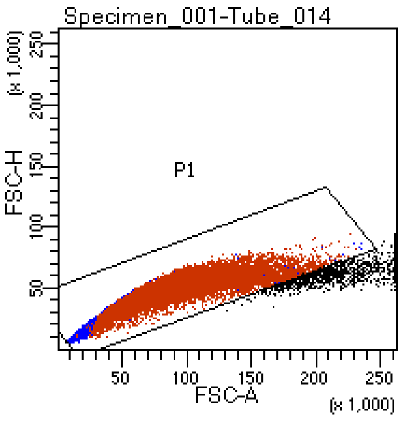

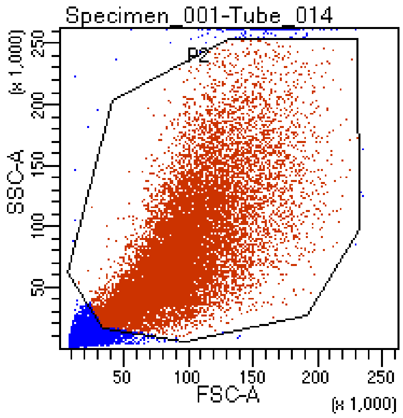

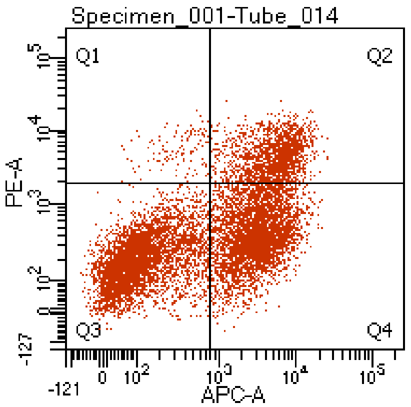

Tube: Tube_014

Population	#Events	%Parent	%Total
All Events	27,632	####	100.0
P1	25,926	93.8	93.8
P2	20,061	77.4	72.6
Q1	251	1.3	0.9
Q2	3,412	17.0	12.3
Q3	8,714	43.4	31.5
Q4	7,684	38.3	27.8

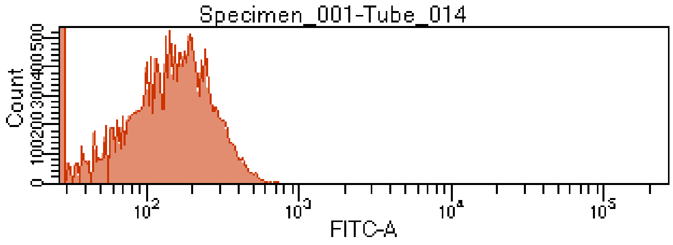

Tube Name: Tube_014
GUID: 6395339e-0e85-4bff-81b0-fe2da01ff5fb

Population	#Events	%Parent	PE-A Mean	PE-A %CV	APC-A Mean	APC-A %CV	APC-Cy7-A Mean	APC-Cy7-A %CV
All Events	27,632	####	1,052	208.9	2,016	140.2	1,235	144.3
P1	25,926	93.8	1,053	204.9	2,086	135.7	1,279	139.7
P2	20,061	77.4	1,234	183.3	2,410	125.7	1,478	129.4
Q1	251	1.3	5,825	54.7	388	49.2	239	53.3
Q2	3,412	17.0	5,053	59.3	5,855	58.8	3,677	61.1
Q3	8,714	43.4	240	90.0	148	114.3	83	122.4
Q4	7,684	38.3	515	79.4	3,512	71.2	2,125	74.1
