# Supplementary material for: Salvia chinensis Benth Inhibits Triple-Negative Breast Cancer Progression by Inducing the DNA Damage Pathway
Source: Front Oncol. 2022 Aug 10;12:882784. doi: 10.3389/fonc.2022.882784 (PMC9404549; doi:10.3389/fonc.2022.882784)
Supplement: Supplementary file 18 [file DataSheet_11.zip › other raw data/figure 4a/35.4T1-Combo-2.pdf]

BD FACSDiva 8.0.1

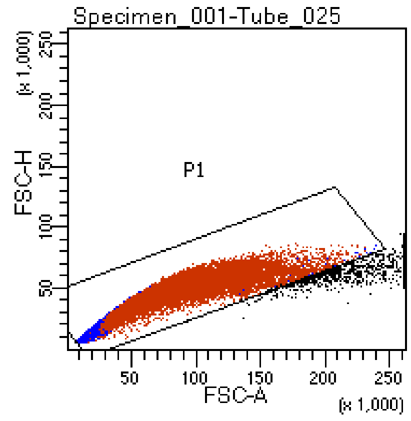

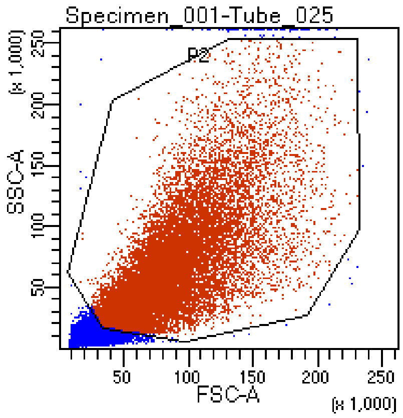

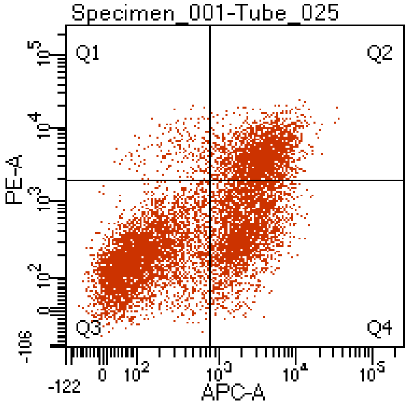

Tube: Tube_025

Population	#Events	%Parent	%Total
All Events	30,386	####	100.0
P1	28,690	94.4	94.4
P2	20,044	69.9	66.0
Q1	447	2.2	1.5
Q2	4,297	21.4	14.1
Q3	9,589	47.8	31.6
Q4	5,711	28.5	18.8

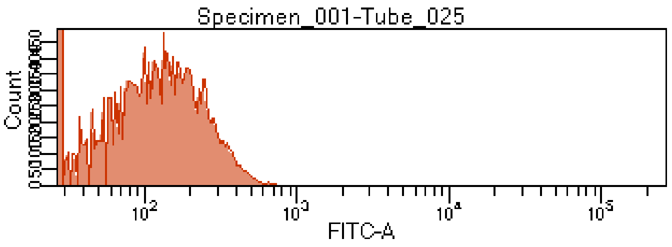

Tube Name: Tube_025
GUID: 14f27bde-0dee-40c8-bc02-0c6d42dbc050

Population	#Events	%Parent	PE-A Mean	PE-A %CV	APC-A Mean	APC-A %CV	APC-Cy7-A Mean	APC-Cy7-A %CV
All Events	30,386	####	1,207	212.0	1,396	150.4	865	154.8
P1	28,690	94.4	1,142	194.5	1,413	146.1	876	150.2
P2	20,044	69.9	1,493	168.8	1,734	134.1	1,077	137.6
Q1	447	2.2	5,317	59.1	388	52.9	233	56.0
Q2	4,297	21.4	5,069	60.2	4,263	66.3	2,702	67.5
Q3	9,589	47.8	246	104.7	156	110.6	86	117.9
Q4	5,711	28.5	597	78.5	2,585	67.4	1,584	70.2
