# Supplementary material for: Salvia chinensis Benth Inhibits Triple-Negative Breast Cancer Progression by Inducing the DNA Damage Pathway
Source: Front Oncol. 2022 Aug 10;12:882784. doi: 10.3389/fonc.2022.882784 (PMC9404549; doi:10.3389/fonc.2022.882784)
Supplement: Supplementary file 18 [file DataSheet_11.zip › other raw data/figure 4a/36.4T1-Combo-3.pdf]

BD FACSDiva 8.0.1

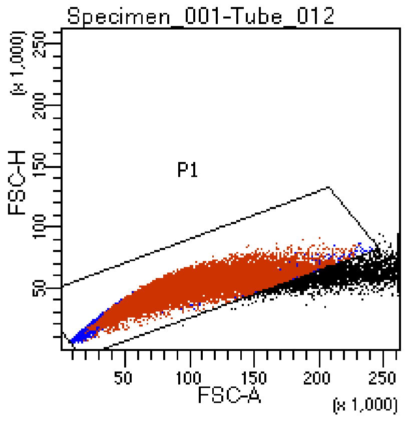

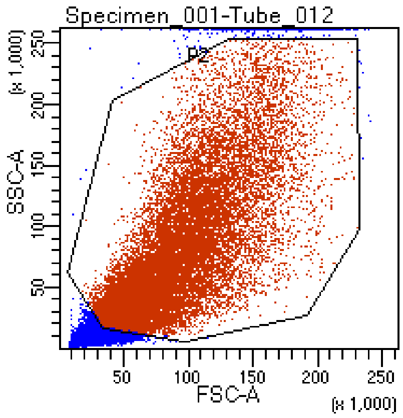

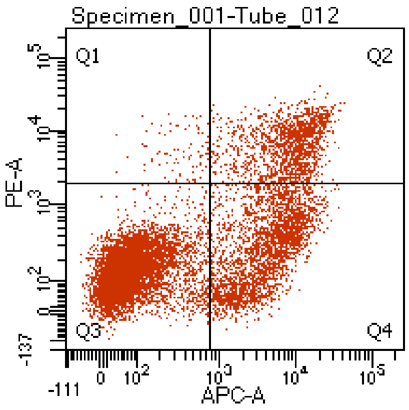

Tube: Tube_012

Population	#Events	%Parent	%Total
All Events	32,259	####	100.0
P1	27,484	85.2	85.2
P2	20,142	73.3	62.4
Q1	197	1.0	0.6
Q2	3,079	15.3	9.5
Q3	11,572	57.5	35.9
Q4	5,294	26.3	16.4

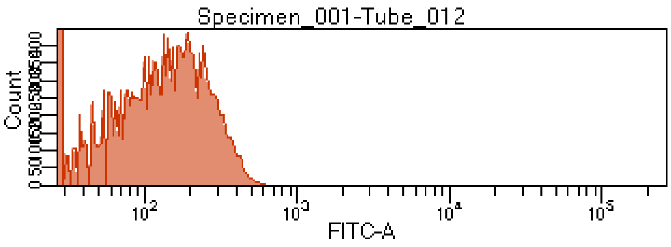

Tube Name: Tube_012
GUID: 8600bd5d-6a28-48e3-8245-93033bc326aa

Population	#Events	%Parent	PE-A Mean	PE-A %CV	APC-A Mean	APC-A %CV	APC-Cy7-A Mean	APC-Cy7-A %CV
All Events	32,259	####	1,239	277.2	2,315	197.7	1,370	206.0
P1	27,484	85.2	1,183	267.4	2,495	183.2	1,480	190.7
P2	20,142	73.3	1,481	235.9	2,997	168.7	1,785	175.1
Q1	197	1.0	5,875	65.9	392	55.8	224	57.1
Q2	3,079	15.3	7,925	66.1	9,896	67.2	6,087	70.3
Q3	11,572	57.5	169	90.8	109	130.1	55	141.1
Q4	5,294	26.3	439	96.1	5,393	82.0	3,123	86.6
