# Supplementary material for: Salvia chinensis Benth Inhibits Triple-Negative Breast Cancer Progression by Inducing the DNA Damage Pathway
Source: Front Oncol. 2022 Aug 10;12:882784. doi: 10.3389/fonc.2022.882784 (PMC9404549; doi:10.3389/fonc.2022.882784)
Supplement: Supplementary file 18 [file DataSheet_11.zip › other raw data/figure 4a/4.231-Q(50uM)-1.pdf]

BD FACSDiva 8.0.1

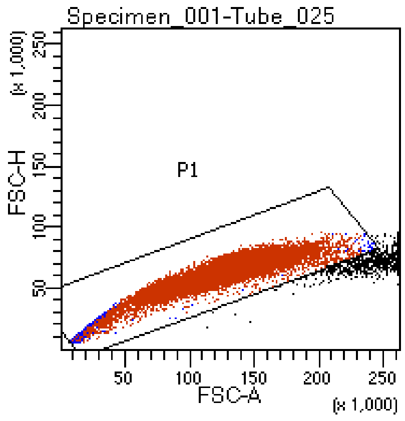

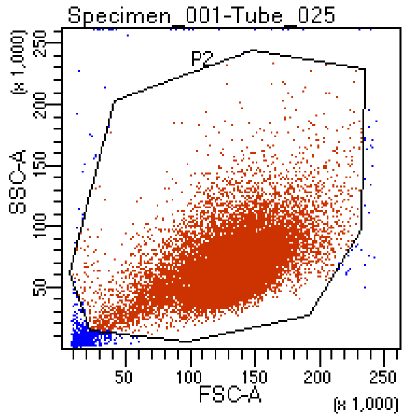

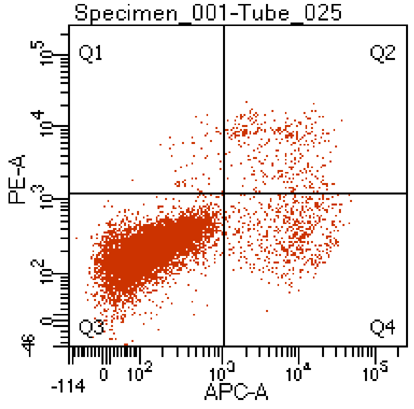

Tube: Tube_025

Population	#Events	%Parent	%Total
All Events	22,754	####	100.0
P1	21,031	92.4	92.4
P2	20,092	95.5	88.3
Q1	133	0.7	0.6
Q2	713	3.5	3.1
Q3	18,006	89.6	79.1
Q4	1,240	6.2	5.4

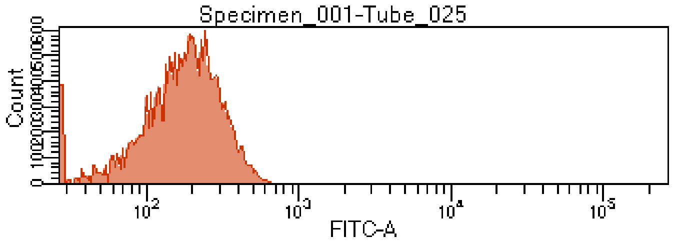

Tube Name: Tube_025
GUID: 7016f021-789a-4946-9376-b3c84627aac5

Population	#Events	%Parent	PE-A Mean	PE-A %CV	APC-A Mean	APC-A %CV	APC-Cy7-A Mean	APC-Cy7-A %CV
All Events	22,754	####	529	303.6	1,074	335.1	644	353.6
P1	21,031	92.4	508	304.2	1,077	336.3	648	354.2
P2	20,092	95.5	518	297.8	1,075	338.9	647	356.6
Q1	133	0.7	4,786	69.3	501	52.2	281	56.6
Q2	713	3.5	6,890	62.5	8,120	84.7	4,893	89.4
Q3	18,006	89.6	240	54.1	175	88.3	93	96.5
Q4	1,240	6.2	432	66.2	10,153	79.3	6,291	84.9
