# Supplementary material for: Salvia chinensis Benth Inhibits Triple-Negative Breast Cancer Progression by Inducing the DNA Damage Pathway
Source: Front Oncol. 2022 Aug 10;12:882784. doi: 10.3389/fonc.2022.882784 (PMC9404549; doi:10.3389/fonc.2022.882784)
Supplement: Supplementary file 18 [file DataSheet_11.zip › other raw data/figure 4a/5.231-Q(50uM)-2.pdf]

BD FACSDiva 8.0.1

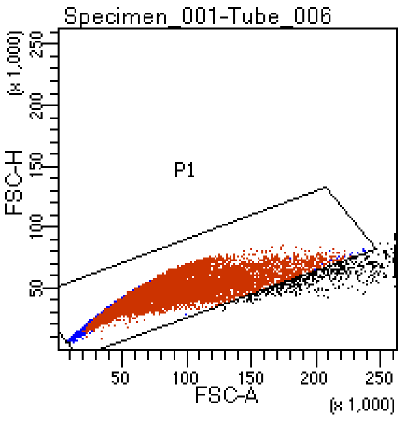

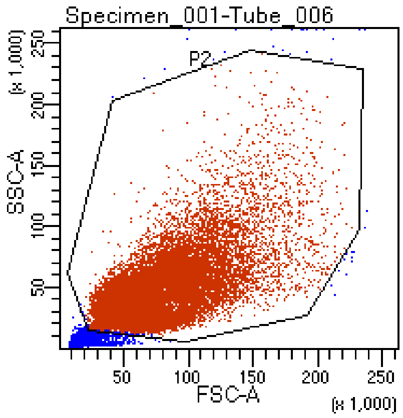

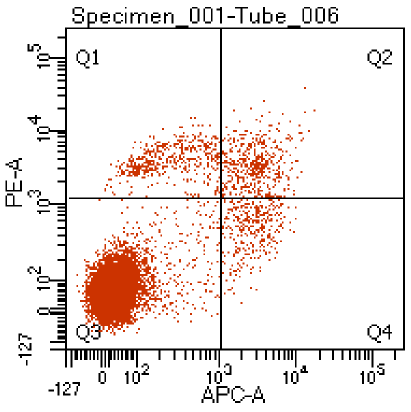

Tube: Tube_006

Population	#Events	%Parent	%Total
All Events	23,455	####	100.0
P1	22,646	96.6	96.6
P2	20,425	90.2	87.1
Q1	949	4.6	4.0
Q2	990	4.8	4.2
Q3	17,803	87.2	75.9
Q4	683	3.3	2.9

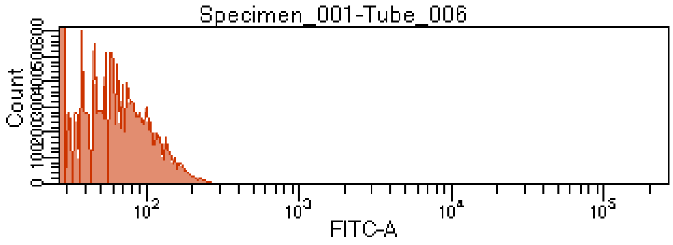

Tube Name: Tube_006
GUID: b368bd4c-b3c7-45b6-9d7e-0cc92f857ecf

Population	#Events	%Parent	PE-A Mean	PE-A %CV	APC-A Mean	APC-A %CV	APC-Cy7-A Mean	APC-Cy7-A %CV
All Events	23,455	####	479	327.7	326	333.8	198	348.6
P1	22,646	96.6	461	321.8	323	328.5	196	342.5
P2	20,425	90.2	482	307.9	306	345.2	186	360.2
Q1	949	4.6	4,194	46.3	335	82.8	196	82.6
Q2	990	4.8	4,307	76.1	3,425	67.6	2,145	71.0
Q3	17,803	87.2	69	120.1	31	281.5	14	370.9
Q4	683	3.3	558	53.7	2,917	53.5	1,806	55.3
