# Supplementary material for: Salvia chinensis Benth Inhibits Triple-Negative Breast Cancer Progression by Inducing the DNA Damage Pathway
Source: Front Oncol. 2022 Aug 10;12:882784. doi: 10.3389/fonc.2022.882784 (PMC9404549; doi:10.3389/fonc.2022.882784)
Supplement: Supplementary file 18 [file DataSheet_11.zip › other raw data/figure 4a/6.231-Q(50uM)-3.pdf]

BD FACSDiva 8.0.1

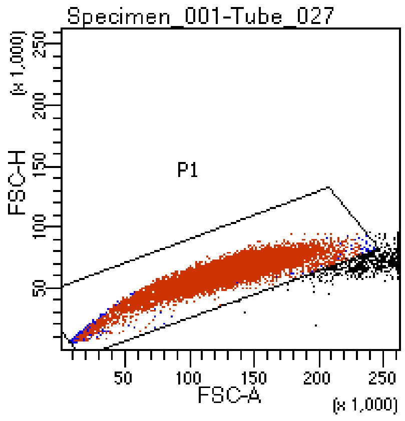

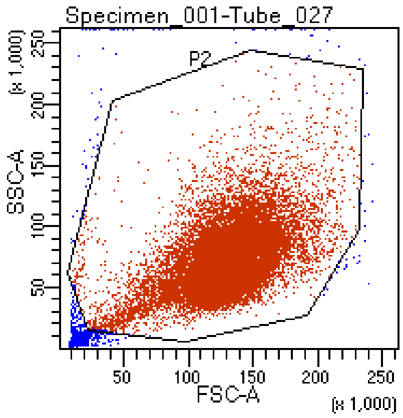

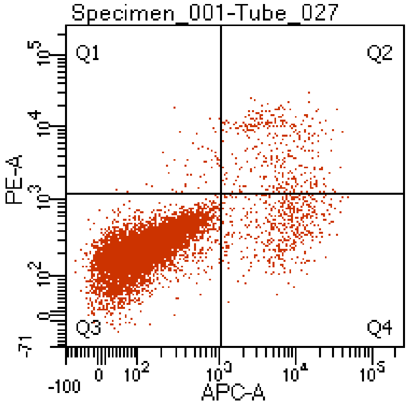

Tube: Tube_027

Population	#Events	%Parent	%Total
All Events	22,401	####	100.0
P1	21,105	94.2	94.2
P2	20,099	95.2	89.7
Q1	97	0.5	0.4
Q2	712	3.5	3.2
Q3	18,265	90.9	81.5
Q4	1,025	5.1	4.6

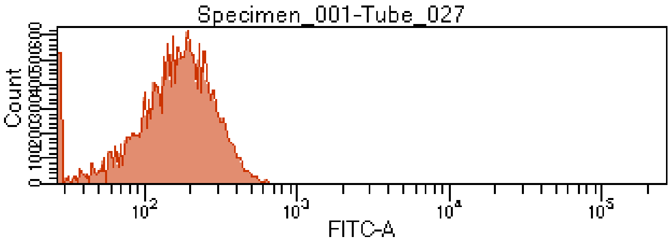

Tube Name: Tube_027
GUID: 5e282b07-9df9-4d3d-b78a-237e5907d301

Population	#Events	%Parent	PE-A Mean	PE-A %CV	APC-A Mean	APC-A %CV	APC-Cy7-A Mean	APC-Cy7-A %CV
All Events	22,401	####	548	323.6	881	343.3	498	360.4
P1	21,105	94.2	529	328.4	869	347.0	492	363.8
P2	20,099	95.2	539	320.1	877	349.3	497	366.0
Q1	97	0.5	4,566	87.7	541	54.1	300	53.8
Q2	712	3.5	7,347	75.3	8,067	85.1	4,664	90.0
Q3	18,265	90.9	253	55.5	160	93.8	82	104.0
Q4	1,025	5.1	527	58.0	8,701	76.9	5,011	82.3
