# Supplementary material for: Salvia chinensis Benth Inhibits Triple-Negative Breast Cancer Progression by Inducing the DNA Damage Pathway
Source: Front Oncol. 2022 Aug 10;12:882784. doi: 10.3389/fonc.2022.882784 (PMC9404549; doi:10.3389/fonc.2022.882784)
Supplement: Supplementary file 18 [file DataSheet_11.zip › other raw data/figure 4a/8.231-B(50uM)-2.pdf]

BD FACSDiva 8.0.1

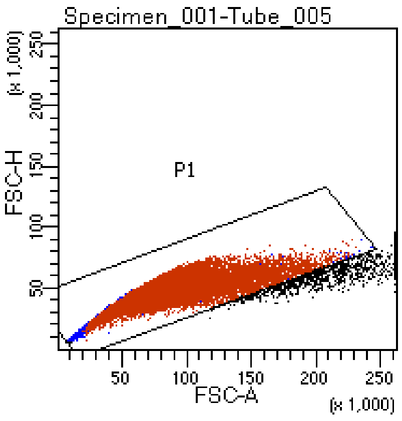

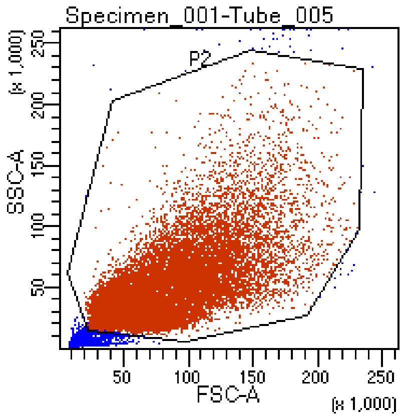

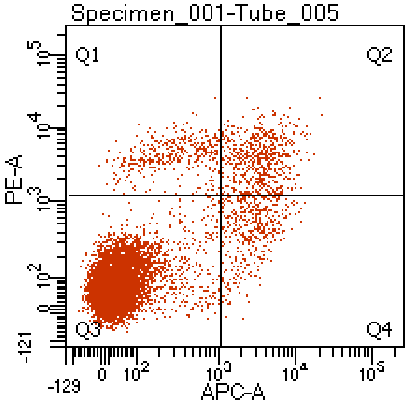

Tube: Tube_005

Population	#Events	%Parent	%Total
All Events	23,542	####	100.0
P1	22,148	94.1	94.1
P2	20,440	92.3	86.8
Q1	870	4.3	3.7
Q2	1,209	5.9	5.1
Q3	17,464	85.4	74.2
Q4	897	4.4	3.8

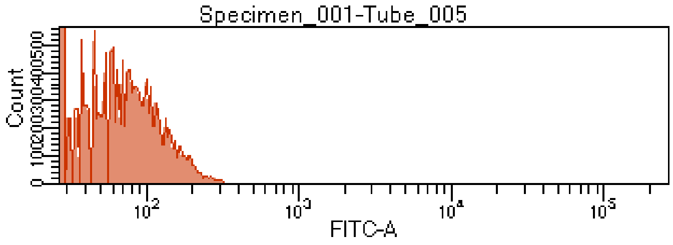

Tube Name: Tube_005
GUID: 1abb6026-7012-46e4-932f-f2247f1505e3

Population	#Events	%Parent	PE-A Mean	PE-A %CV	APC-A Mean	APC-A %CV	APC-Cy7-A Mean	APC-Cy7-A %CV
All Events	23,542	####	579	312.6	450	339.0	277	357.1
P1	22,148	94.1	556	300.7	448	304.1	276	317.7
P2	20,440	92.3	569	296.9	428	319.6	263	333.7
Q1	870	4.3	4,530	51.7	368	77.1	218	76.7
Q2	1,209	5.9	4,713	74.1	4,004	71.2	2,539	74.5
Q3	17,464	85.4	85	108.7	46	230.5	22	278.0
Q4	897	4.4	553	61.6	3,102	65.2	1,934	69.0
