# Supplementary material for: Salvia chinensis Benth Inhibits Triple-Negative Breast Cancer Progression by Inducing the DNA Damage Pathway
Source: Front Oncol. 2022 Aug 10;12:882784. doi: 10.3389/fonc.2022.882784 (PMC9404549; doi:10.3389/fonc.2022.882784)
Supplement: Supplementary file 18 [file DataSheet_11.zip › other raw data/figure 4a/9.231-B(50uM)-3.pdf]

BD FACSDiva 8.0.1

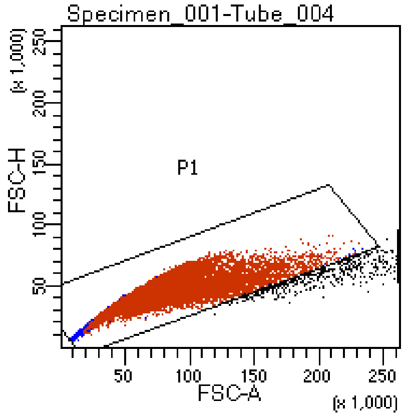

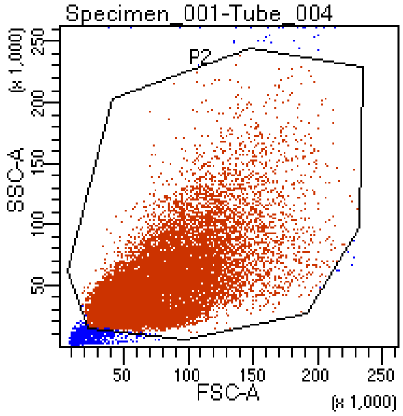

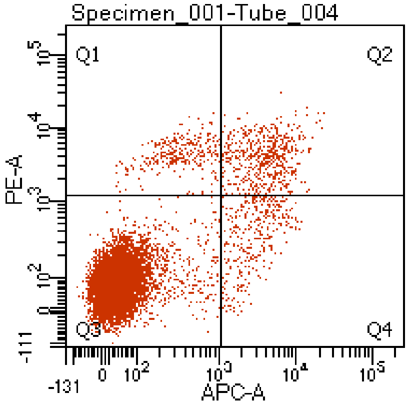

Tube: Tube_004

Population	#Events	%Parent	%Total
All Events	22,394	####	100.0
P1	21,690	96.9	96.9
P2	20,426	94.2	91.2
Q1	674	3.3	3.0
Q2	1,036	5.1	4.6
Q3	18,020	88.2	80.5
Q4	696	3.4	3.1

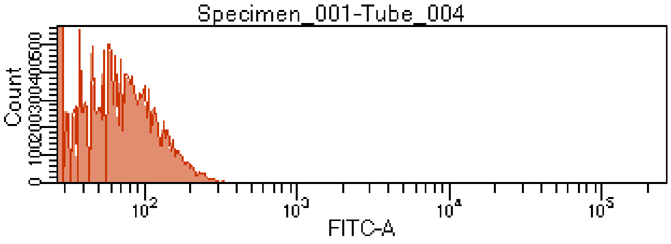

Tube Name: Tube_004
GUID: e7b33e9a-dd28-4cae-ae21-62f0de3eb5bc

Population	#Events	%Parent	PE-A Mean	PE-A %CV	APC-A Mean	APC-A %CV	APC-Cy7-A Mean	APC-Cy7-A %CV
All Events	22,394	####	507	317.8	434	339.7	267	355.3
P1	21,690	96.9	484	313.4	431	337.3	266	353.0
P2	20,426	94.2	485	311.9	405	358.6	249	375.2
Q1	674	3.3	4,660	46.3	414	62.9	250	62.1
Q2	1,036	5.1	4,795	63.0	4,697	71.0	2,977	75.2
Q3	18,020	88.2	79	106.8	40	228.3	20	278.7
Q4	696	3.4	529	66.7	3,441	64.3	2,141	68.0
