# Supplementary figures and images for: Salvia chinensis Benth Inhibits Triple-Negative Breast Cancer Progression by Inducing the DNA Damage Pathway (part 2 of 2)
Source: Front Oncol. 2022 Aug 10;12:882784. doi: 10.3389/fonc.2022.882784 (PMC9404549; doi:10.3389/fonc.2022.882784)

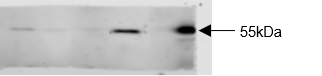

Supplement: Supplementary file 18 [file DataSheet_11.zip › other raw data/figure 7b/fig.7b.p-CHK1.tif]

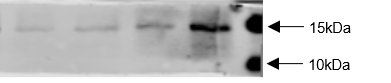

Supplement: Supplementary file 18 [file DataSheet_11.zip › other raw data/figure 7b/fig.7b.p-H2AX.tif]

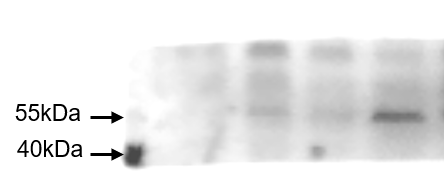

Supplement: Supplementary file 18 [file DataSheet_11.zip › other raw data/figure 7b/fig.7b.p-P53.tif]

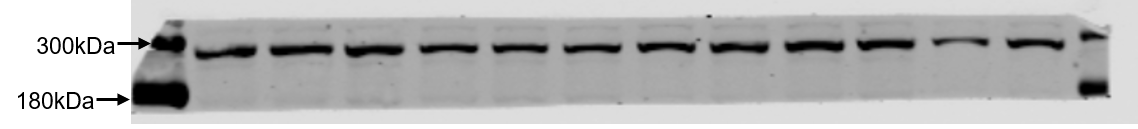

Supplement: Supplementary file 18 [file DataSheet_11.zip › other raw data/figure 8c/fig.8c.ATM.tif]

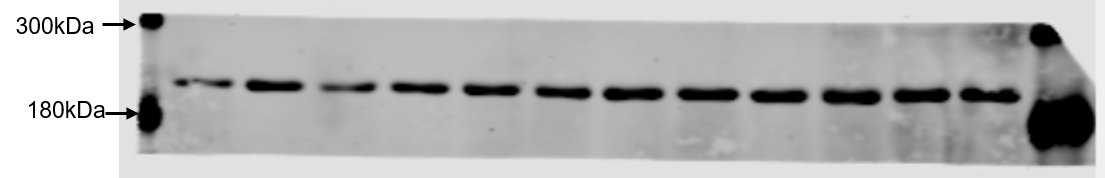

Supplement: Supplementary file 18 [file DataSheet_11.zip › other raw data/figure 8c/fig.8c.ATR.tif]

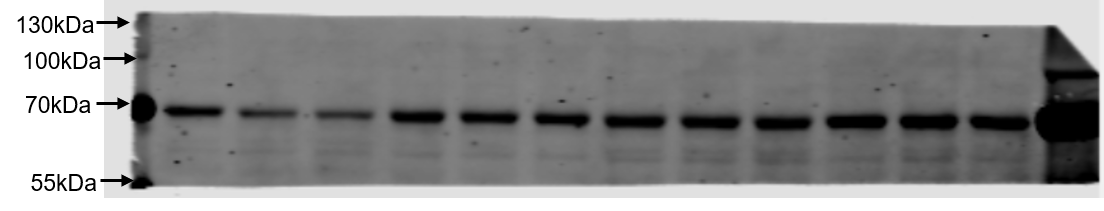

Supplement: Supplementary file 18 [file DataSheet_11.zip › other raw data/figure 8c/fig.8c.CHK1.tif]

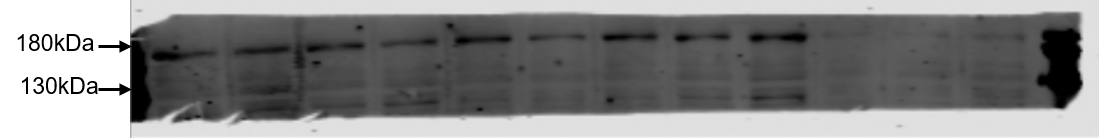

Supplement: Supplementary file 18 [file DataSheet_11.zip › other raw data/figure 8c/fig.8c.FANCD2.tif]

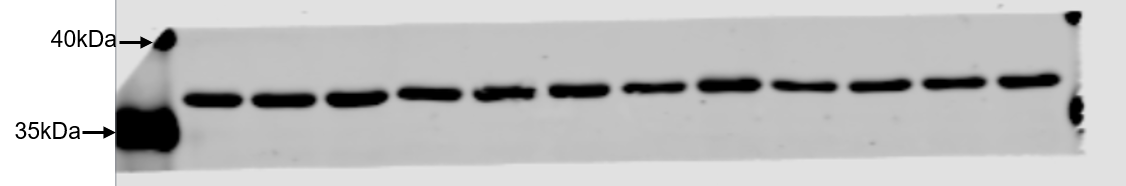

Supplement: Supplementary file 18 [file DataSheet_11.zip › other raw data/figure 8c/fig.8c.GAPDH.tif]

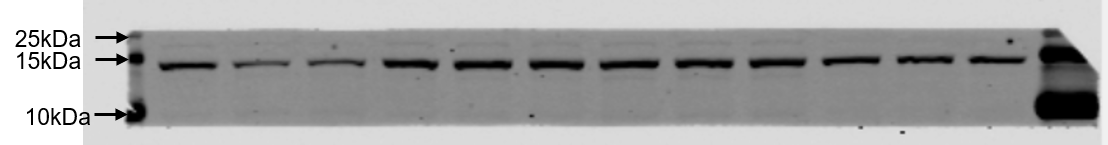

Supplement: Supplementary file 18 [file DataSheet_11.zip › other raw data/figure 8c/fig.8c.H2AX.tif]

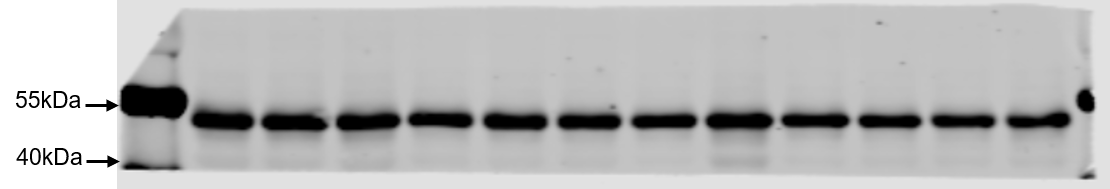

Supplement: Supplementary file 18 [file DataSheet_11.zip › other raw data/figure 8c/fig.8c.P53.tif]

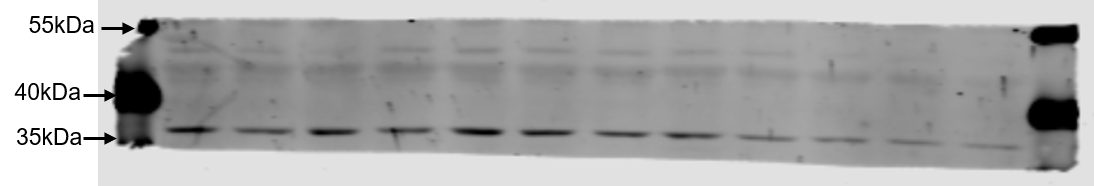

Supplement: Supplementary file 18 [file DataSheet_11.zip › other raw data/figure 8c/fig.8c.RAD51.tif]

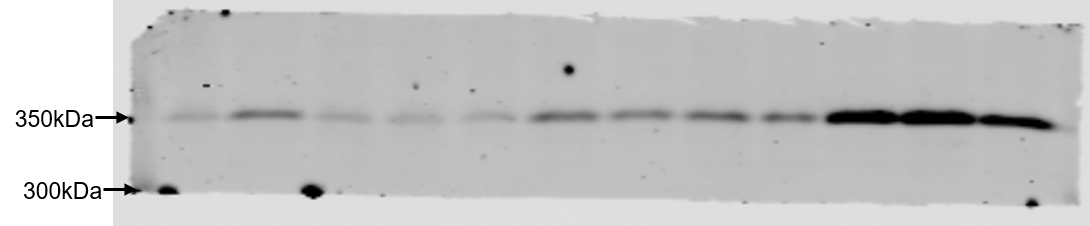

Supplement: Supplementary file 18 [file DataSheet_11.zip › other raw data/figure 8c/fig.8c.p-ATM.tif]

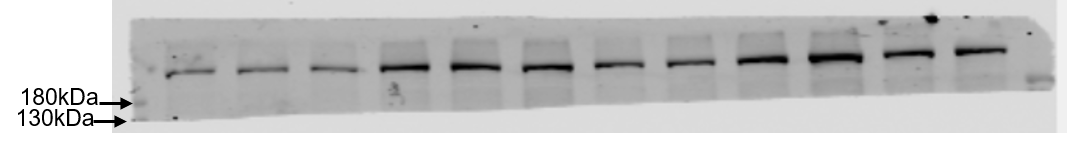

Supplement: Supplementary file 18 [file DataSheet_11.zip › other raw data/figure 8c/fig.8c.p-ATR.tif]

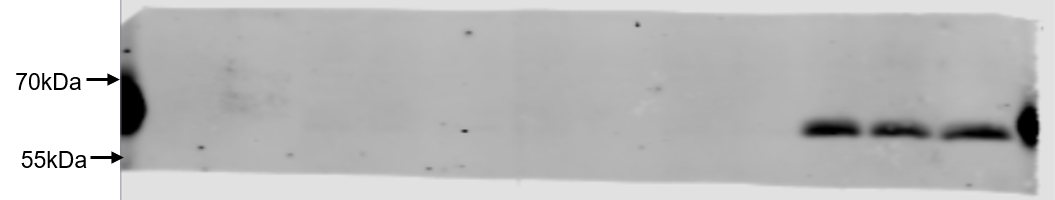

Supplement: Supplementary file 18 [file DataSheet_11.zip › other raw data/figure 8c/fig.8c.p-CHK1.tif]

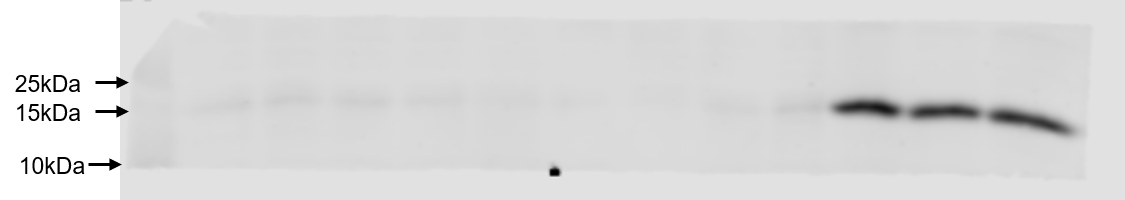

Supplement: Supplementary file 18 [file DataSheet_11.zip › other raw data/figure 8c/fig.8c.p-H2AX.tif]

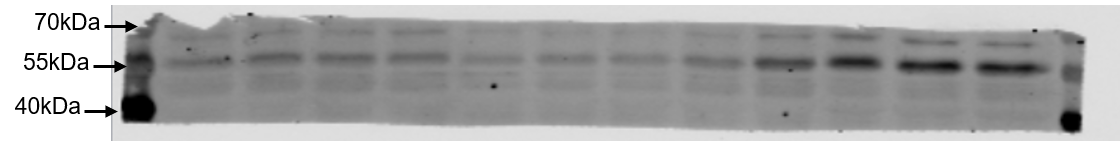

Supplement: Supplementary file 18 [file DataSheet_11.zip › other raw data/figure 8c/fig.8c.p-P53.tif]
